# Supplementary figures and images for: circ-EGFR is a predictor of response to Cetuximab and a potential target in colorectal cancer (part 1 of 2)
Source: EMBO Mol Med. 2025 Nov 10;17(12):3525–54. doi: 10.1038/s44321-025-00333-0 (PMC12686431; doi:10.1038/s44321-025-00333-0)

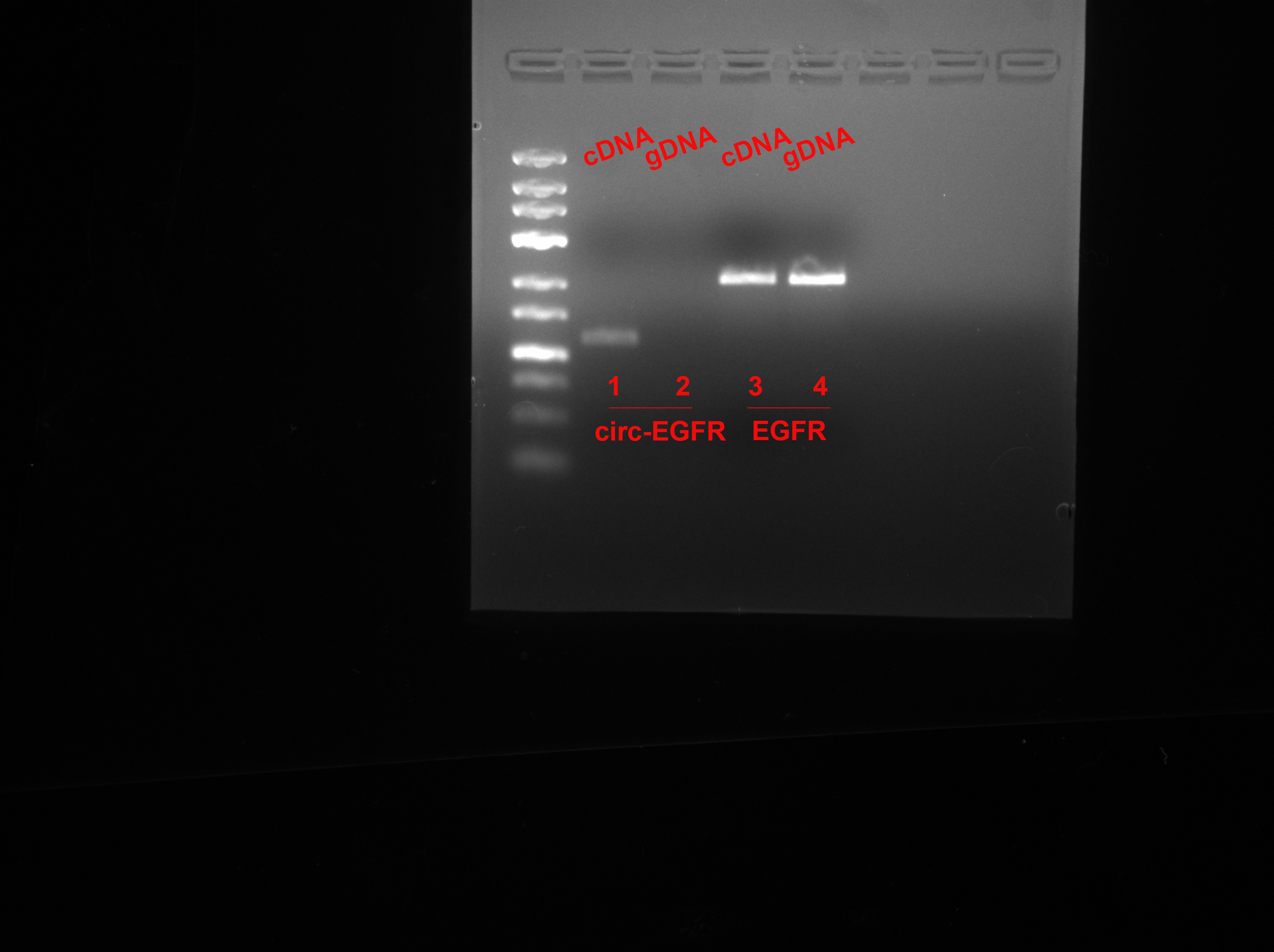

Supplement: Supplementary file 6 — Source data Fig. 1 [file 44321_2025_333_MOESM6_ESM.zip › Figure 1/1C/DiFi/DiFi_gel.tif]

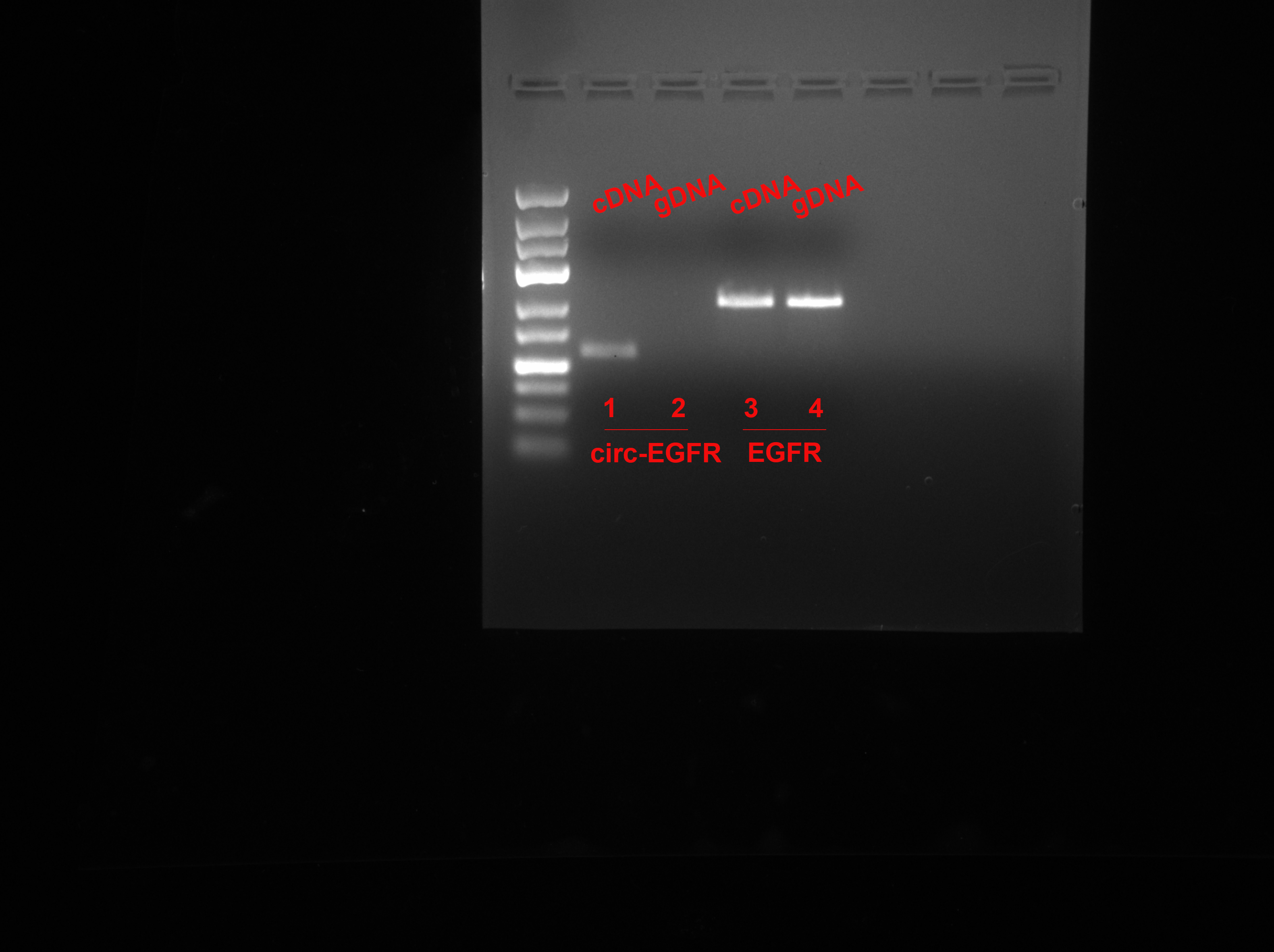

Supplement: Supplementary file 6 — Source data Fig. 1 [file 44321_2025_333_MOESM6_ESM.zip › Figure 1/1C/SW48/SW48_gel.tif]

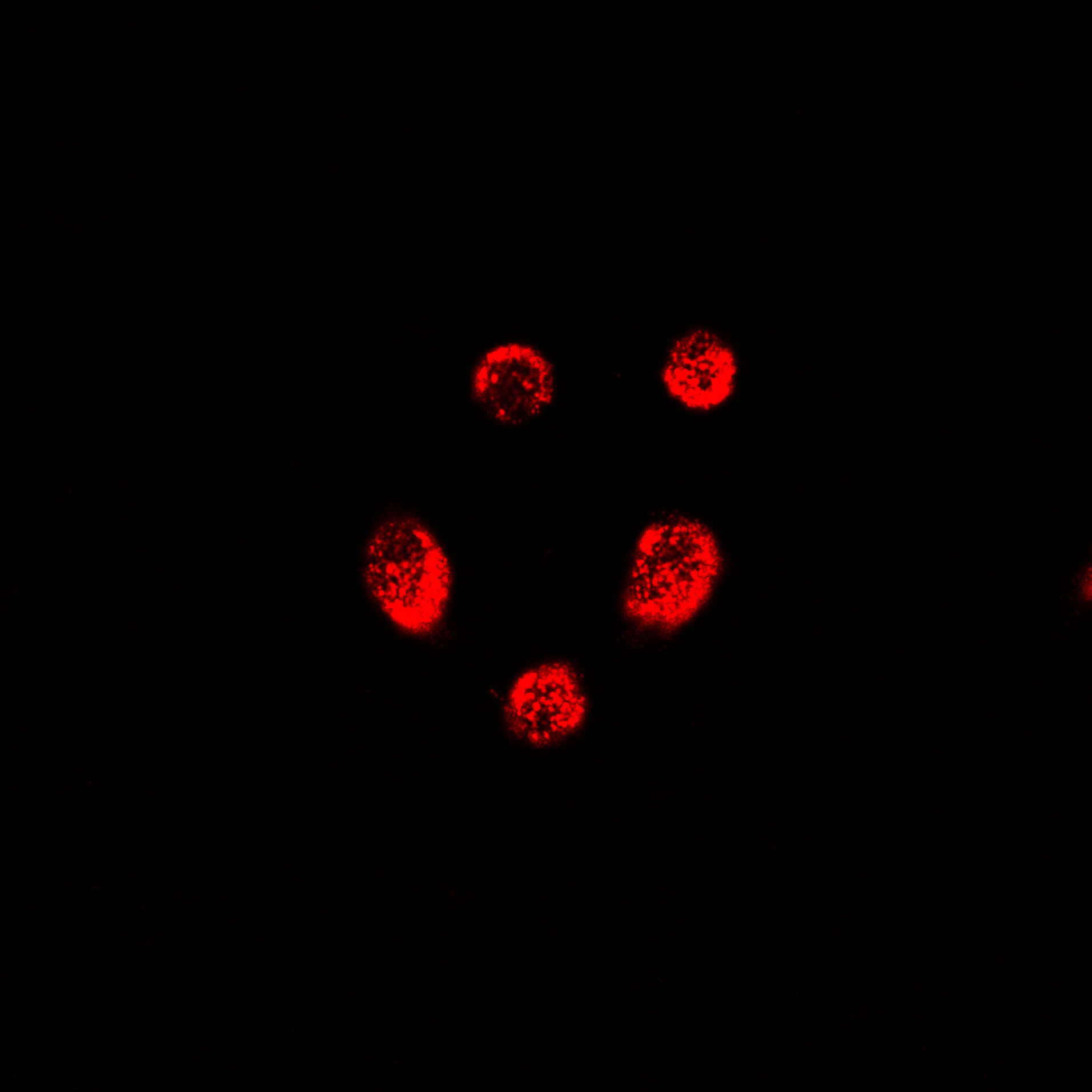

Supplement: Supplementary file 6 — Source data Fig. 1 [file 44321_2025_333_MOESM6_ESM.zip › Figure 1/1E/DiFi/circEGFR.tiff]

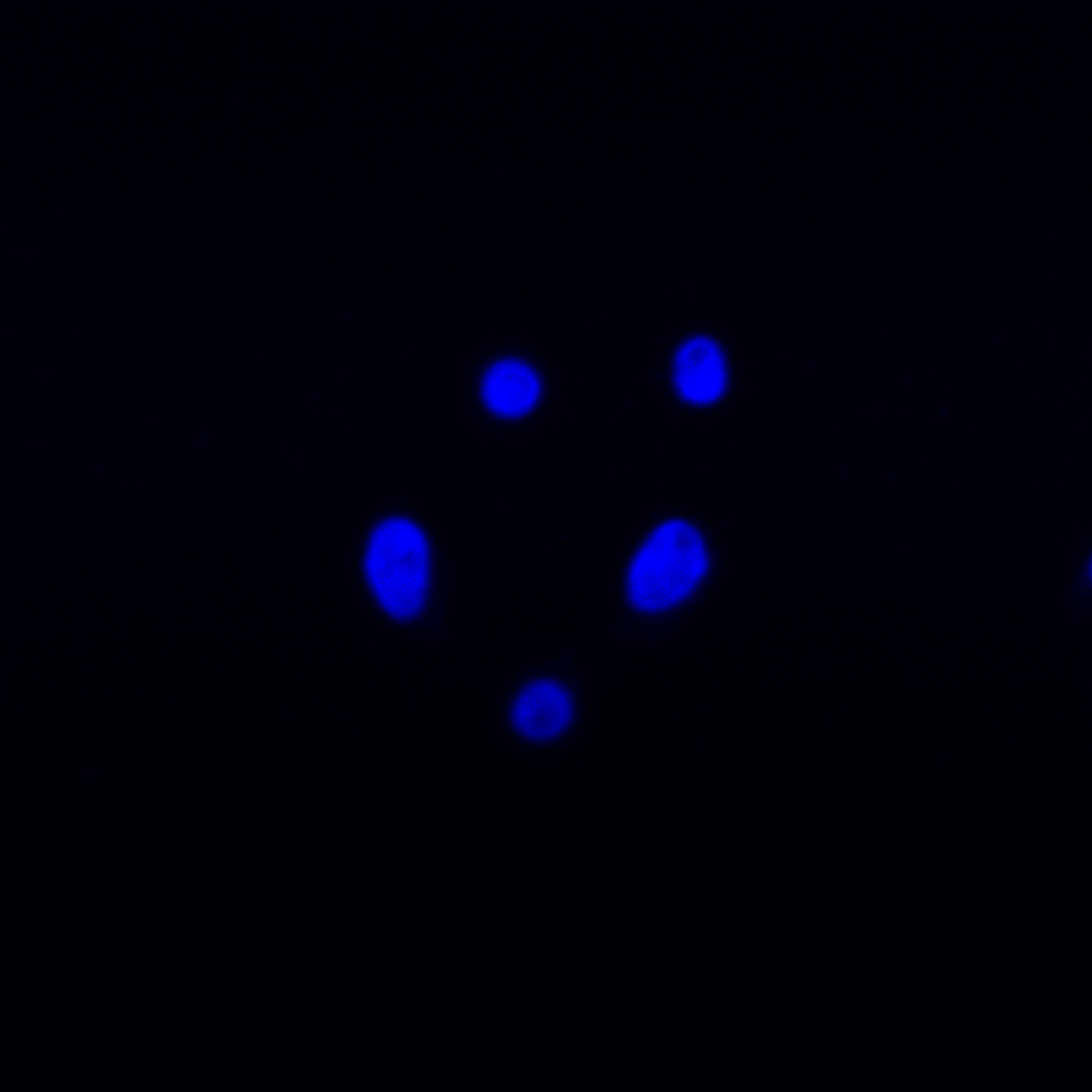

Supplement: Supplementary file 6 — Source data Fig. 1 [file 44321_2025_333_MOESM6_ESM.zip › Figure 1/1E/DiFi/DAPI.tiff]

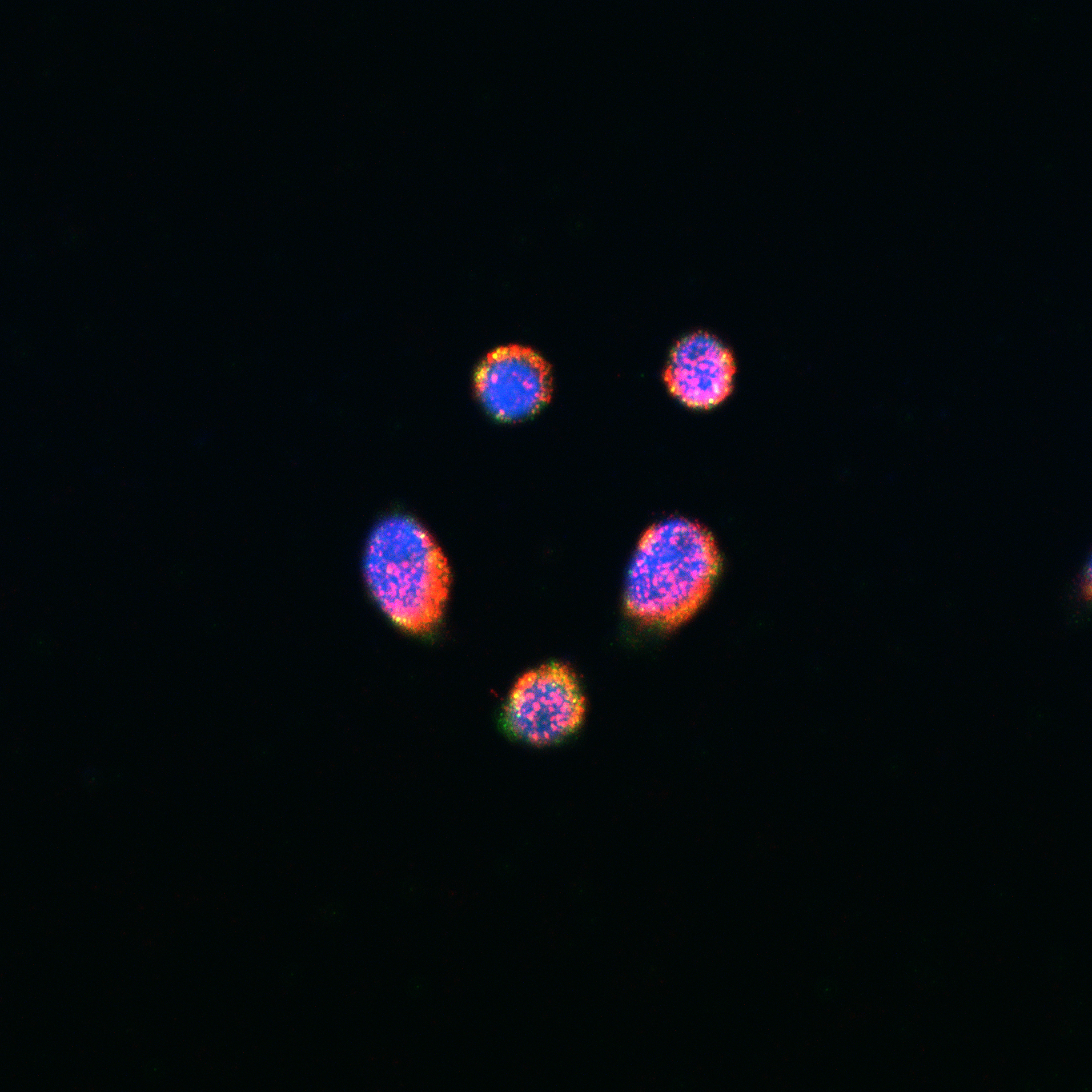

Supplement: Supplementary file 6 — Source data Fig. 1 [file 44321_2025_333_MOESM6_ESM.zip › Figure 1/1E/DiFi/Merge.tiff]

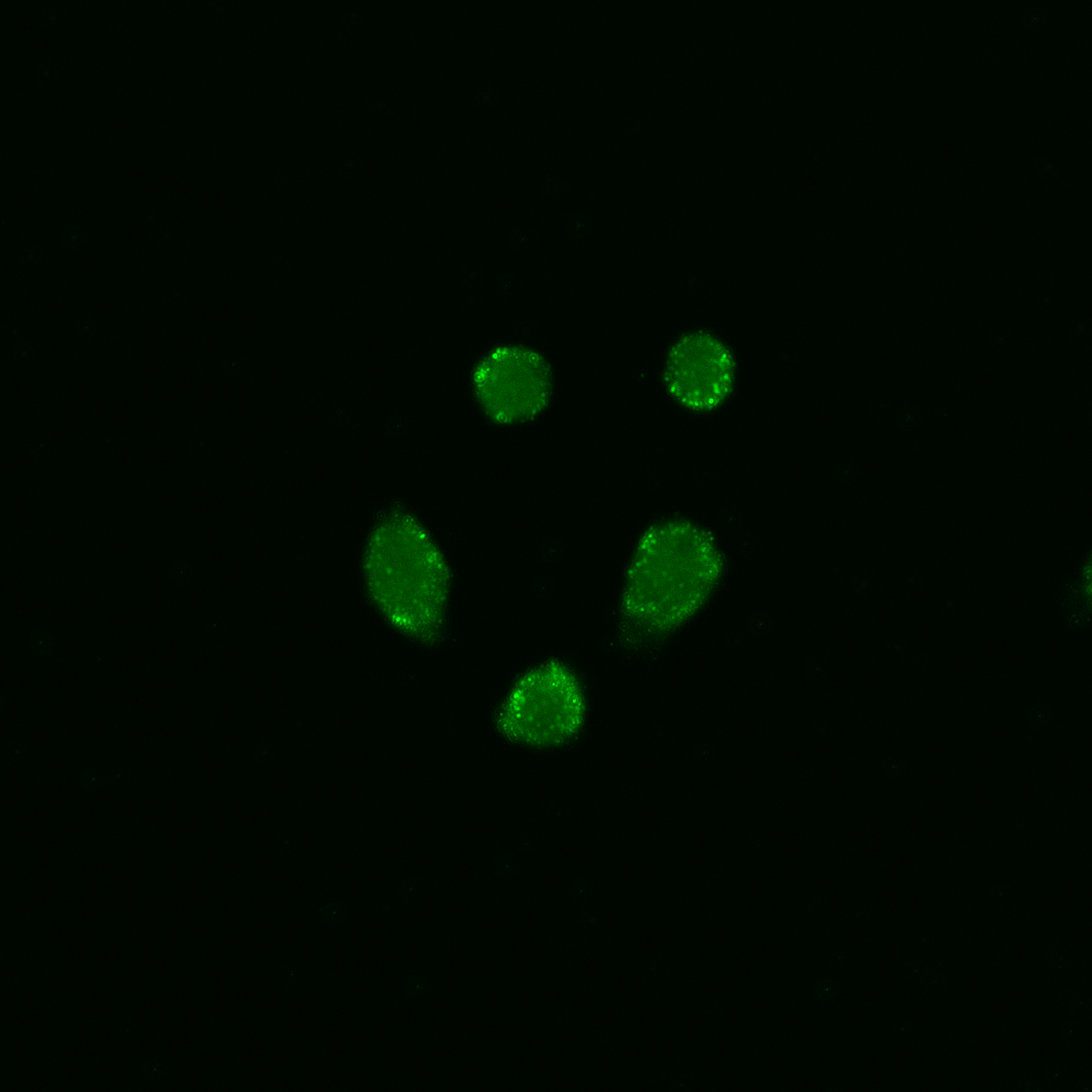

Supplement: Supplementary file 6 — Source data Fig. 1 [file 44321_2025_333_MOESM6_ESM.zip › Figure 1/1E/DiFi/miR-942-3p.tiff]

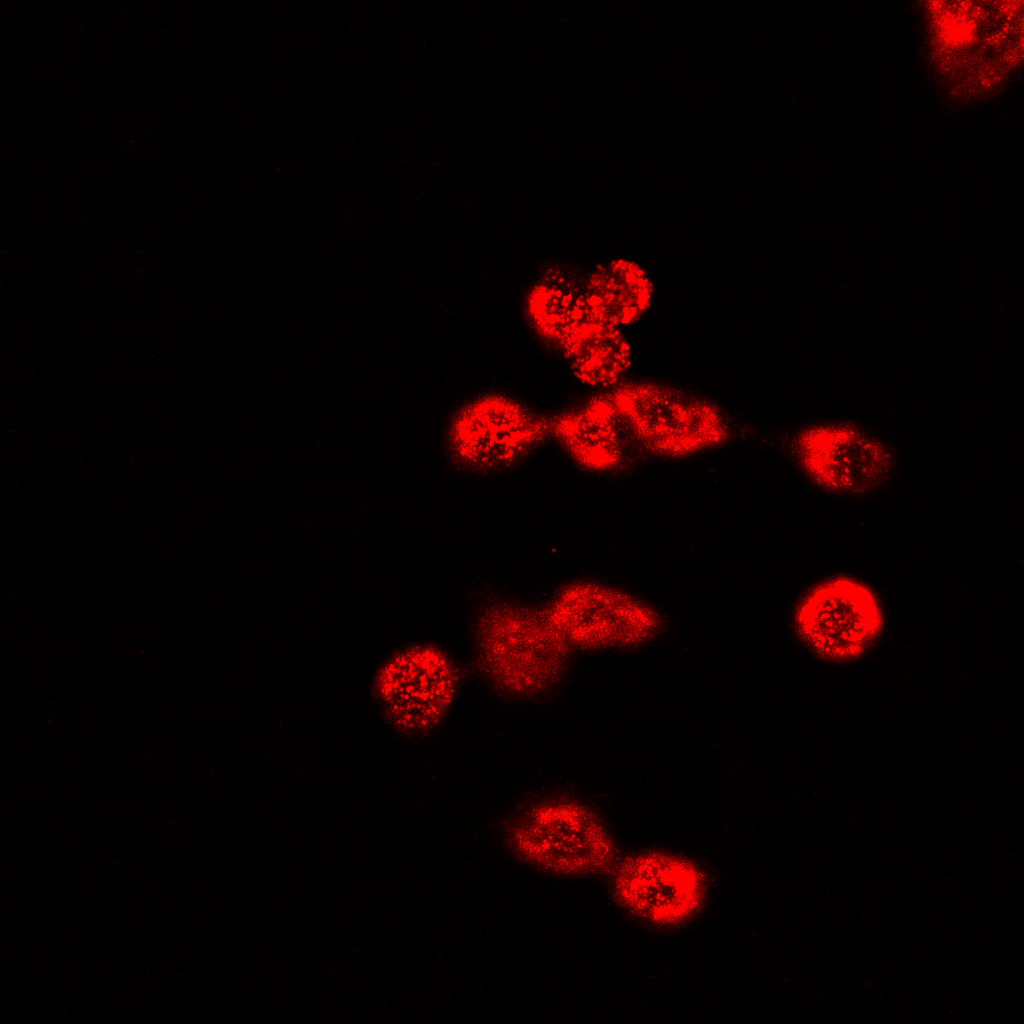

Supplement: Supplementary file 6 — Source data Fig. 1 [file 44321_2025_333_MOESM6_ESM.zip › Figure 1/1E/SW48/circEGFR.tiff]

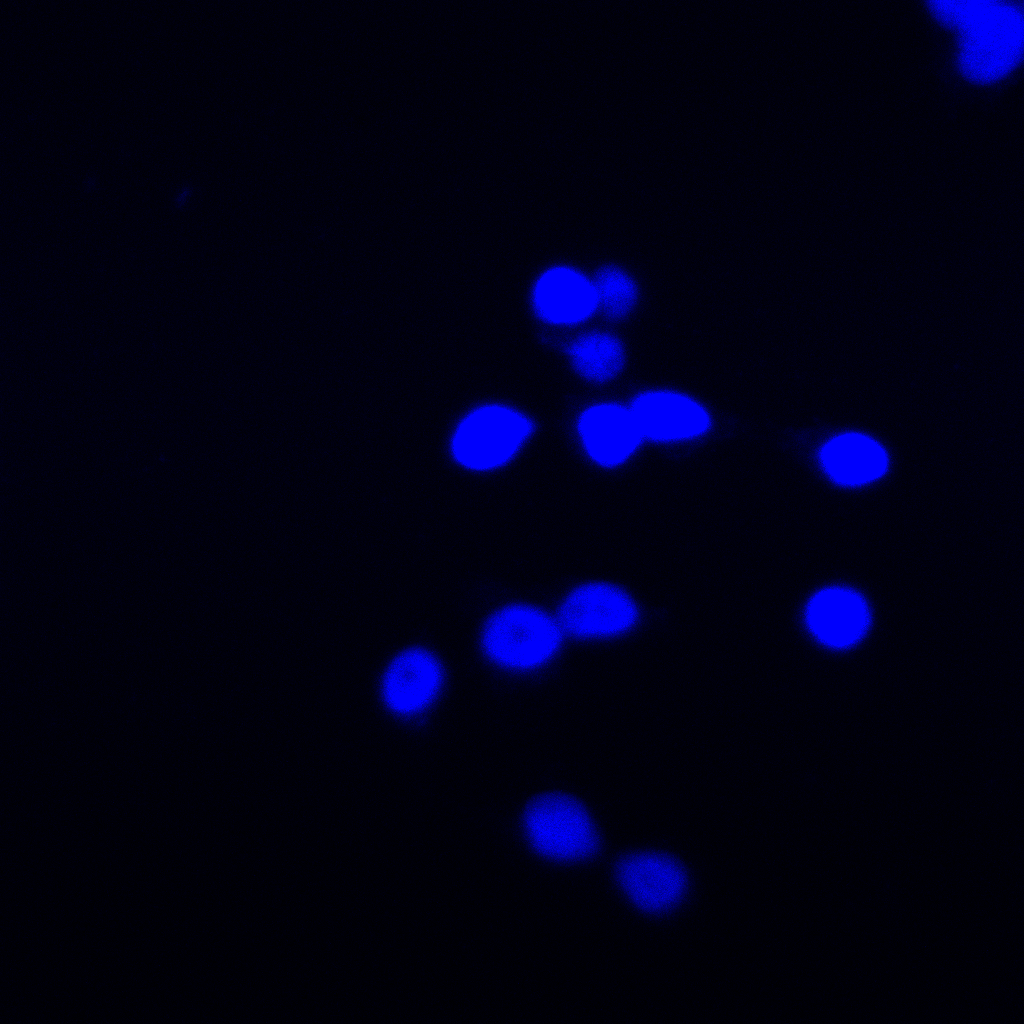

Supplement: Supplementary file 6 — Source data Fig. 1 [file 44321_2025_333_MOESM6_ESM.zip › Figure 1/1E/SW48/DAPI.tiff]

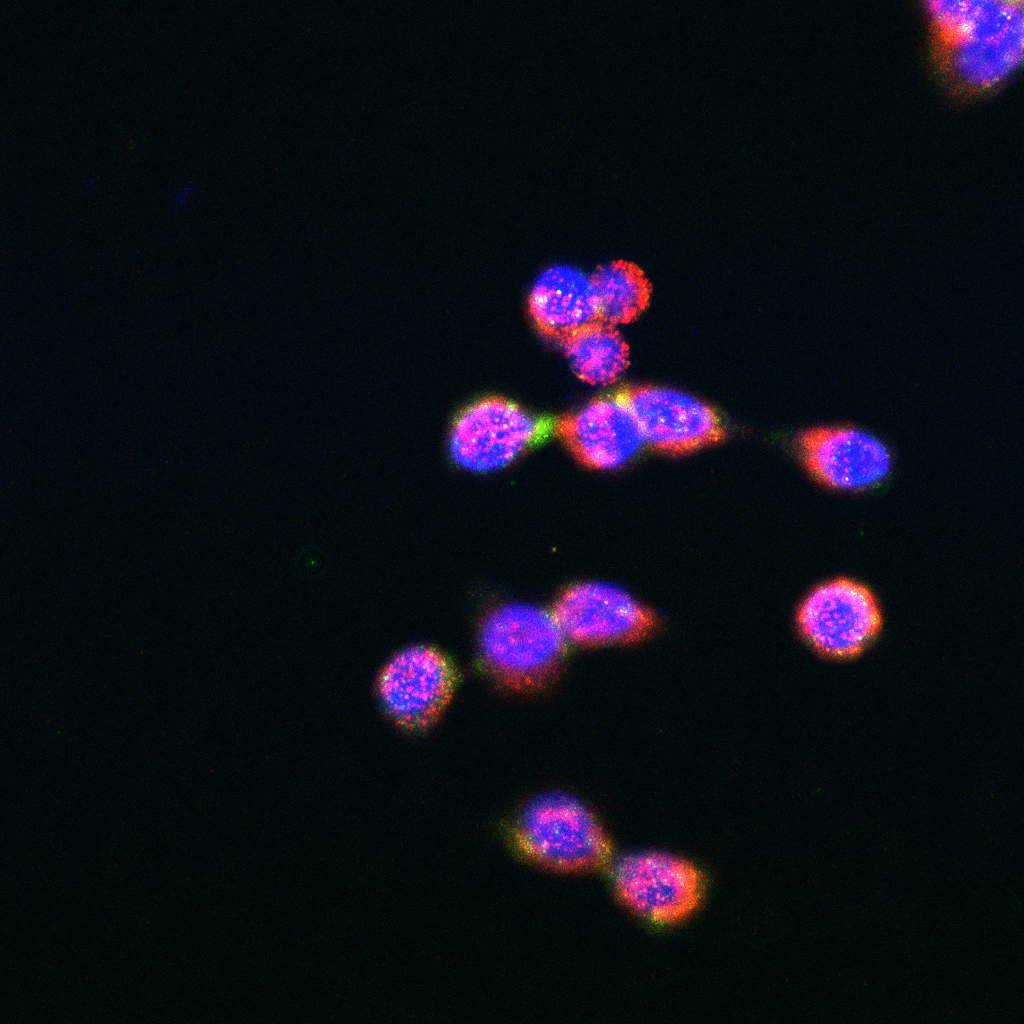

Supplement: Supplementary file 6 — Source data Fig. 1 [file 44321_2025_333_MOESM6_ESM.zip › Figure 1/1E/SW48/Merge.tiff]

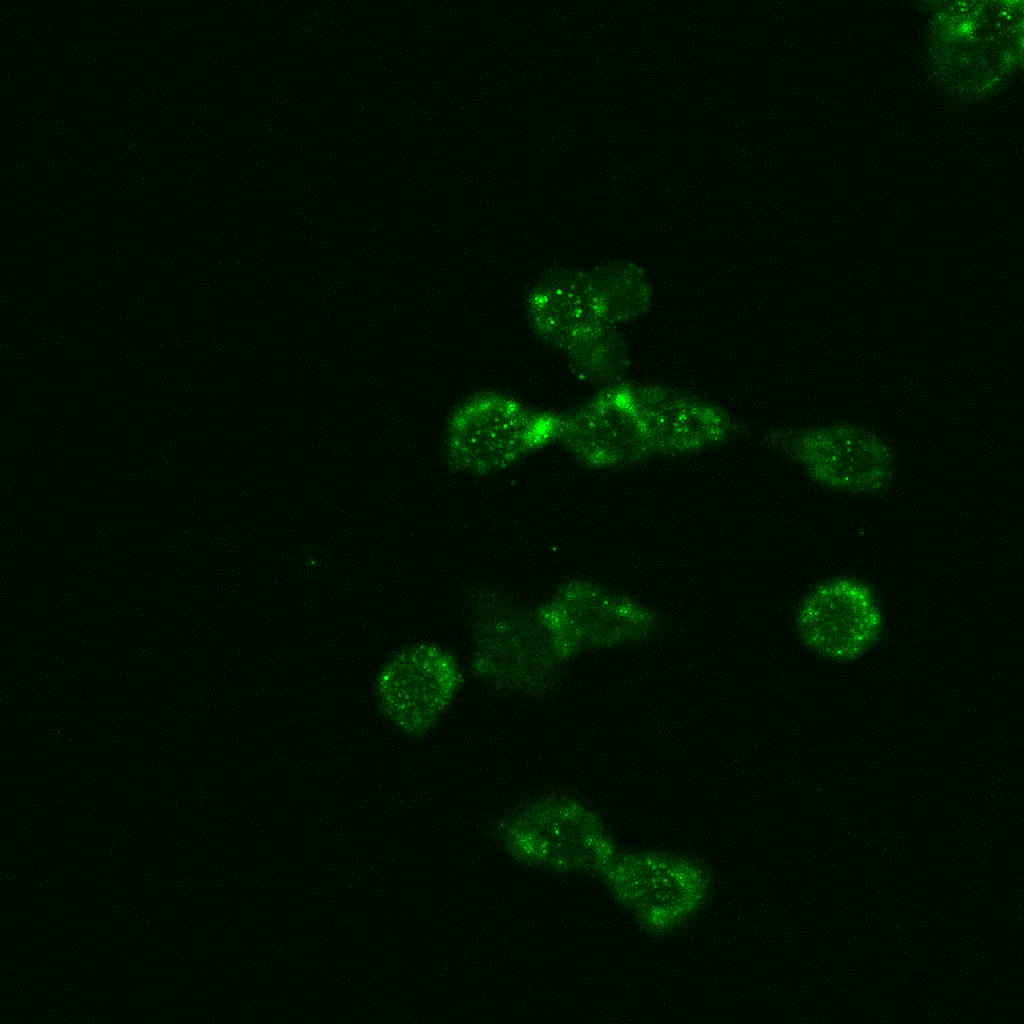

Supplement: Supplementary file 6 — Source data Fig. 1 [file 44321_2025_333_MOESM6_ESM.zip › Figure 1/1E/SW48/miR-942-3p.tiff]

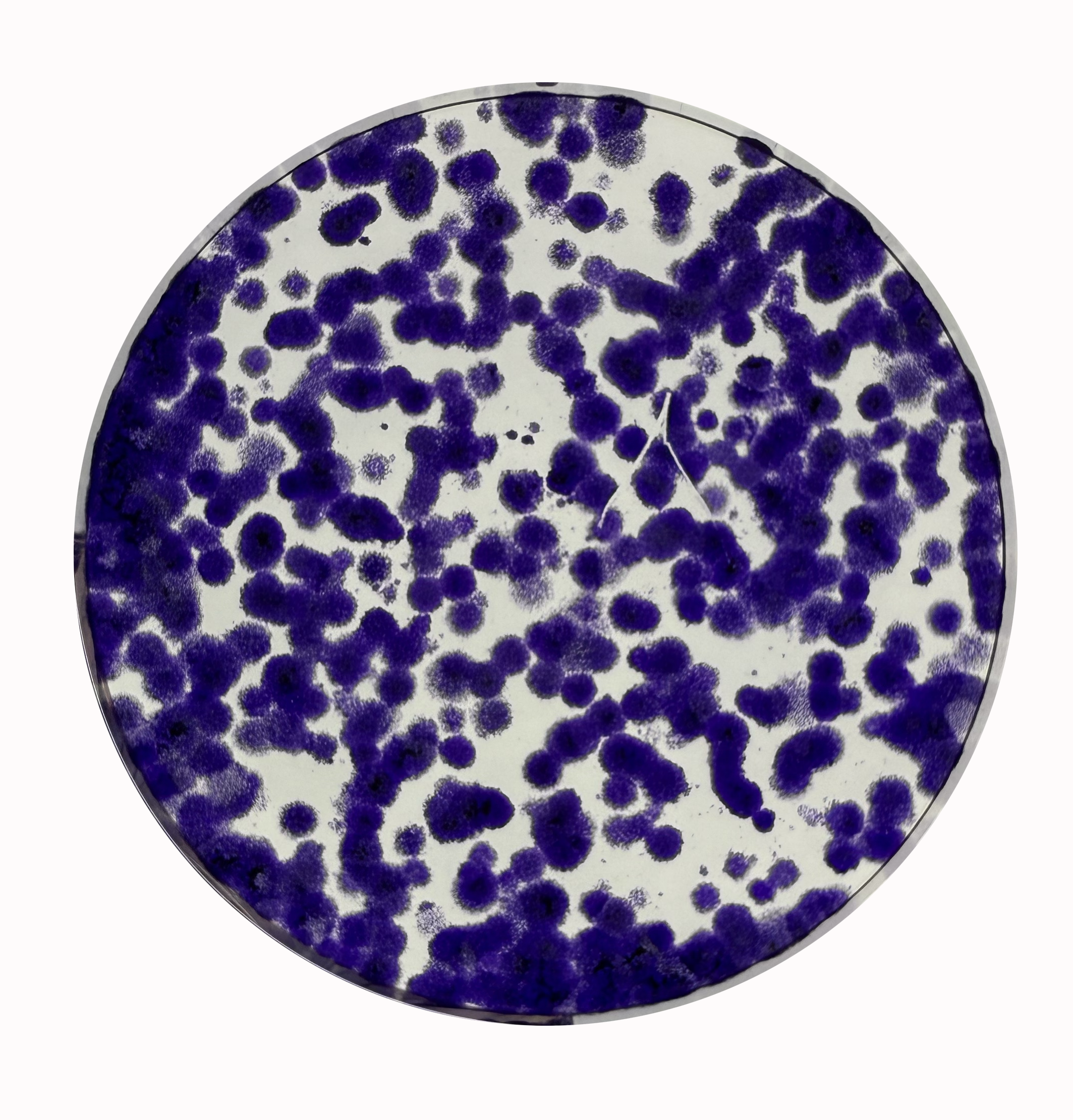

Supplement: Supplementary file 7 — Source data Fig. 2 [file 44321_2025_333_MOESM7_ESM.zip › Figure 2/2C/CaCO2/Rep 1/1_NC.tif]

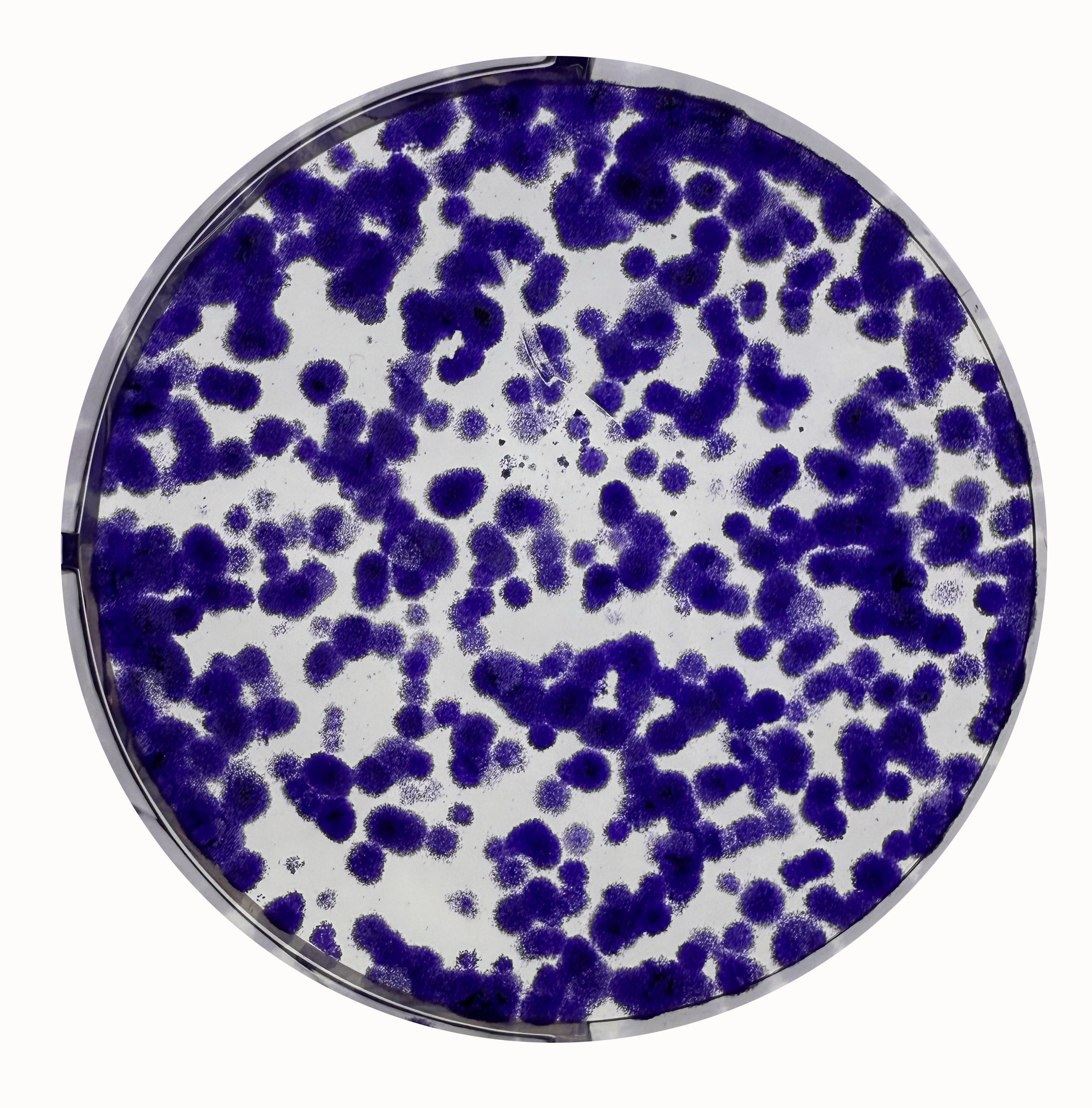

Supplement: Supplementary file 7 — Source data Fig. 2 [file 44321_2025_333_MOESM7_ESM.zip › Figure 2/2C/CaCO2/Rep 1/2_OE circ.tif]

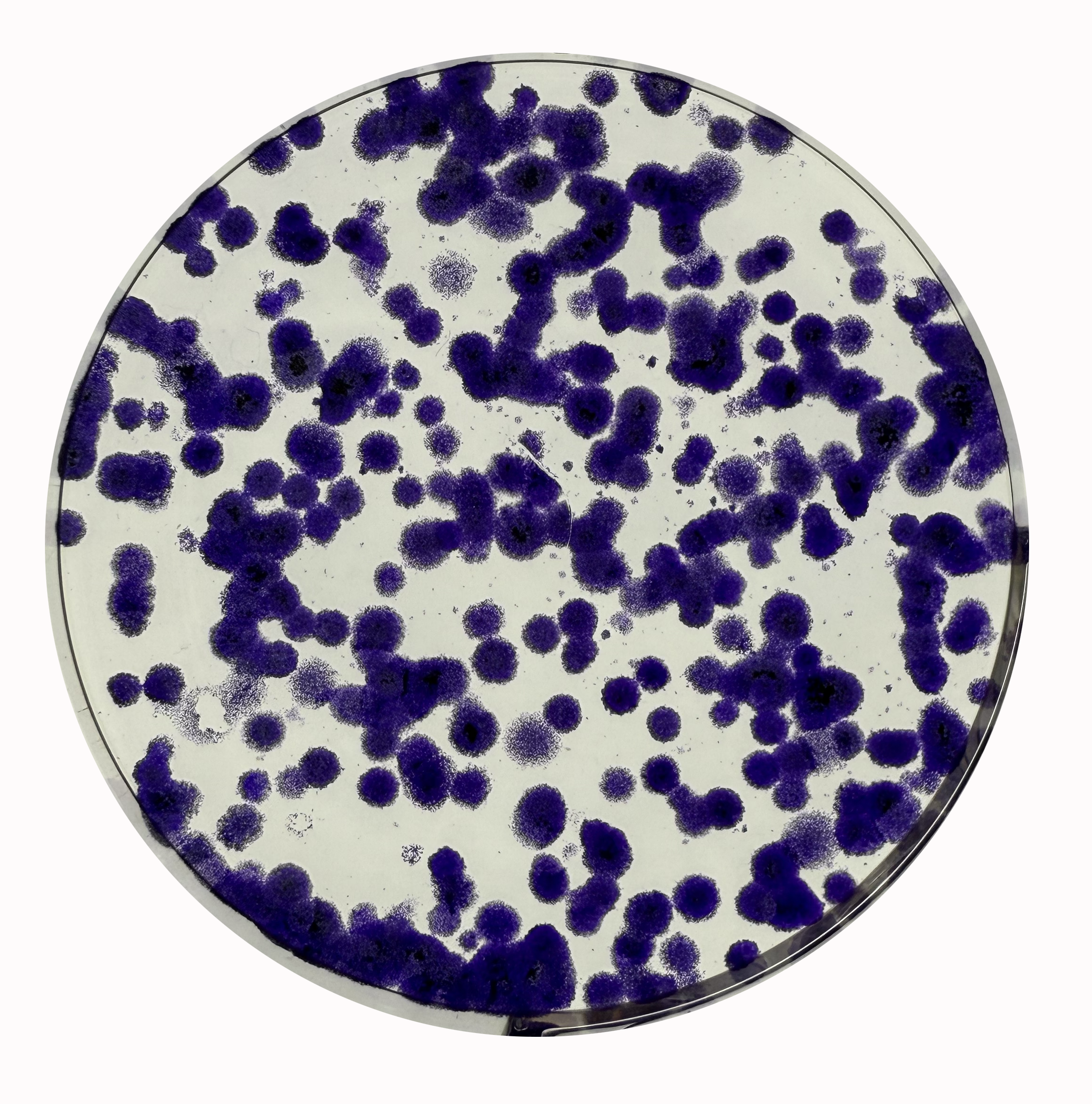

Supplement: Supplementary file 7 — Source data Fig. 2 [file 44321_2025_333_MOESM7_ESM.zip › Figure 2/2C/CaCO2/Rep 1/3_OE+NC.tif]

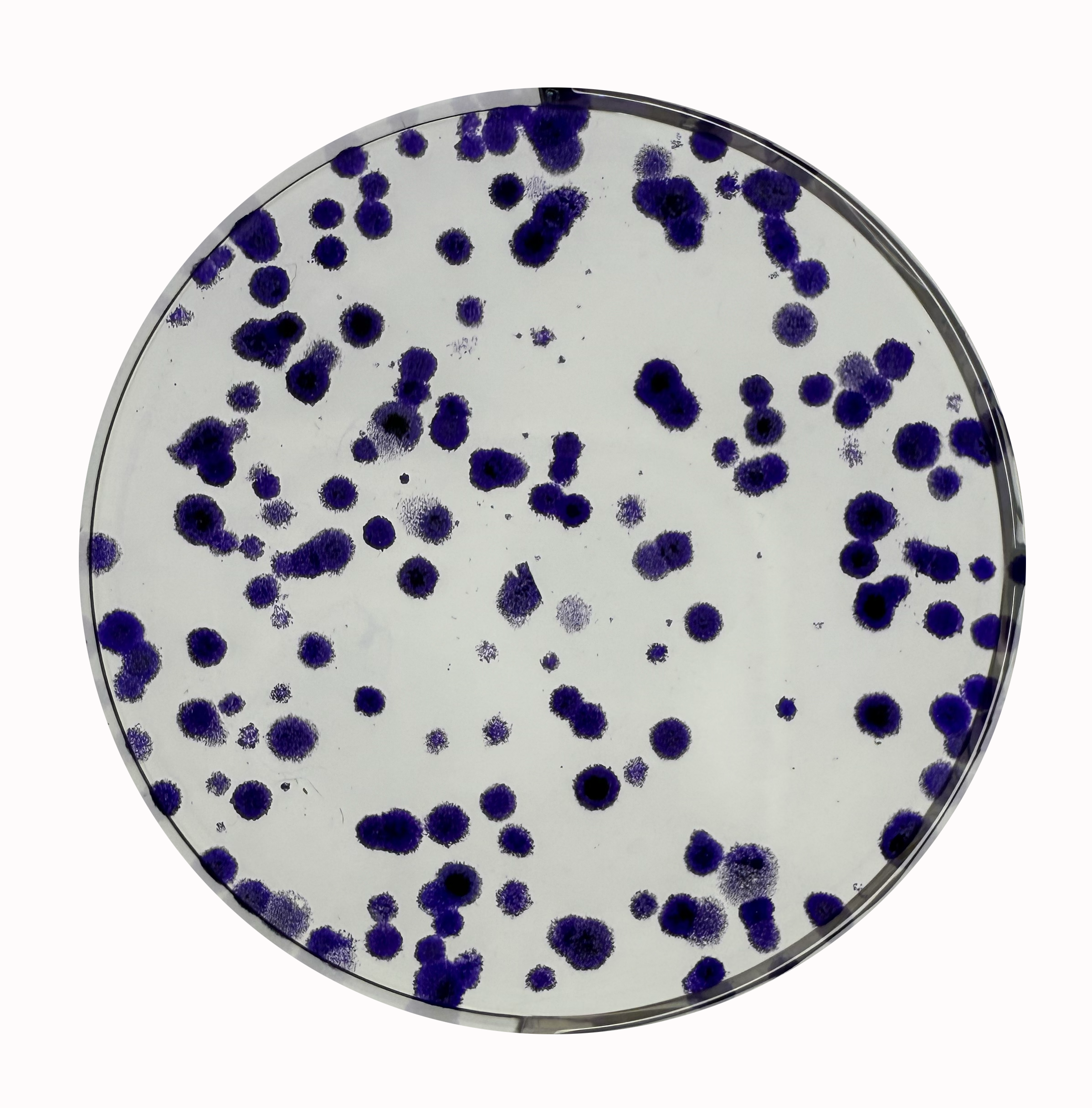

Supplement: Supplementary file 7 — Source data Fig. 2 [file 44321_2025_333_MOESM7_ESM.zip › Figure 2/2C/CaCO2/Rep 1/4_OE+OE.tif]

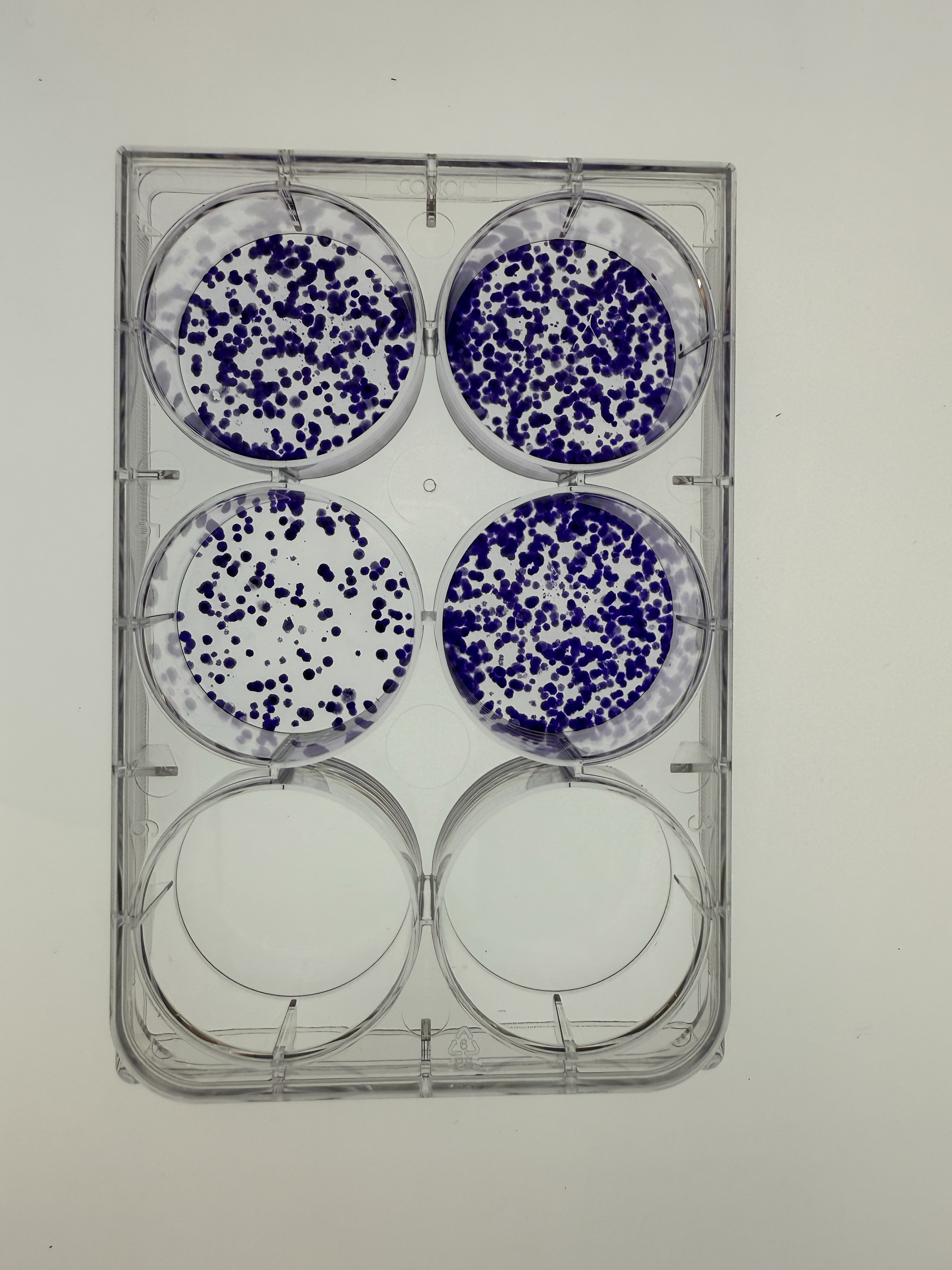

Supplement: Supplementary file 7 — Source data Fig. 2 [file 44321_2025_333_MOESM7_ESM.zip › Figure 2/2C/CaCO2/Rep 1/overall.tif]

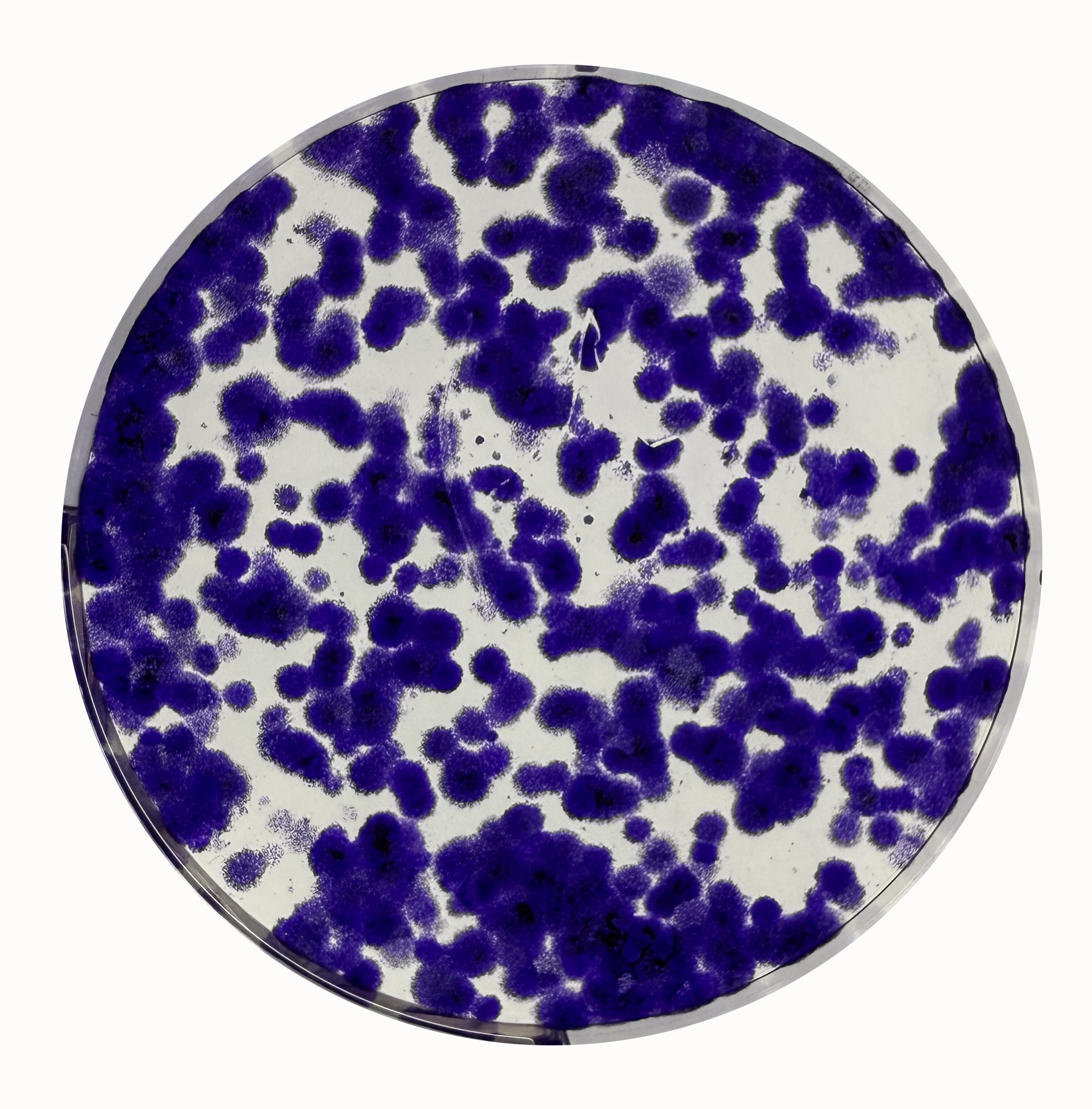

Supplement: Supplementary file 7 — Source data Fig. 2 [file 44321_2025_333_MOESM7_ESM.zip › Figure 2/2C/CaCO2/Rep 2/1_NC.tif]

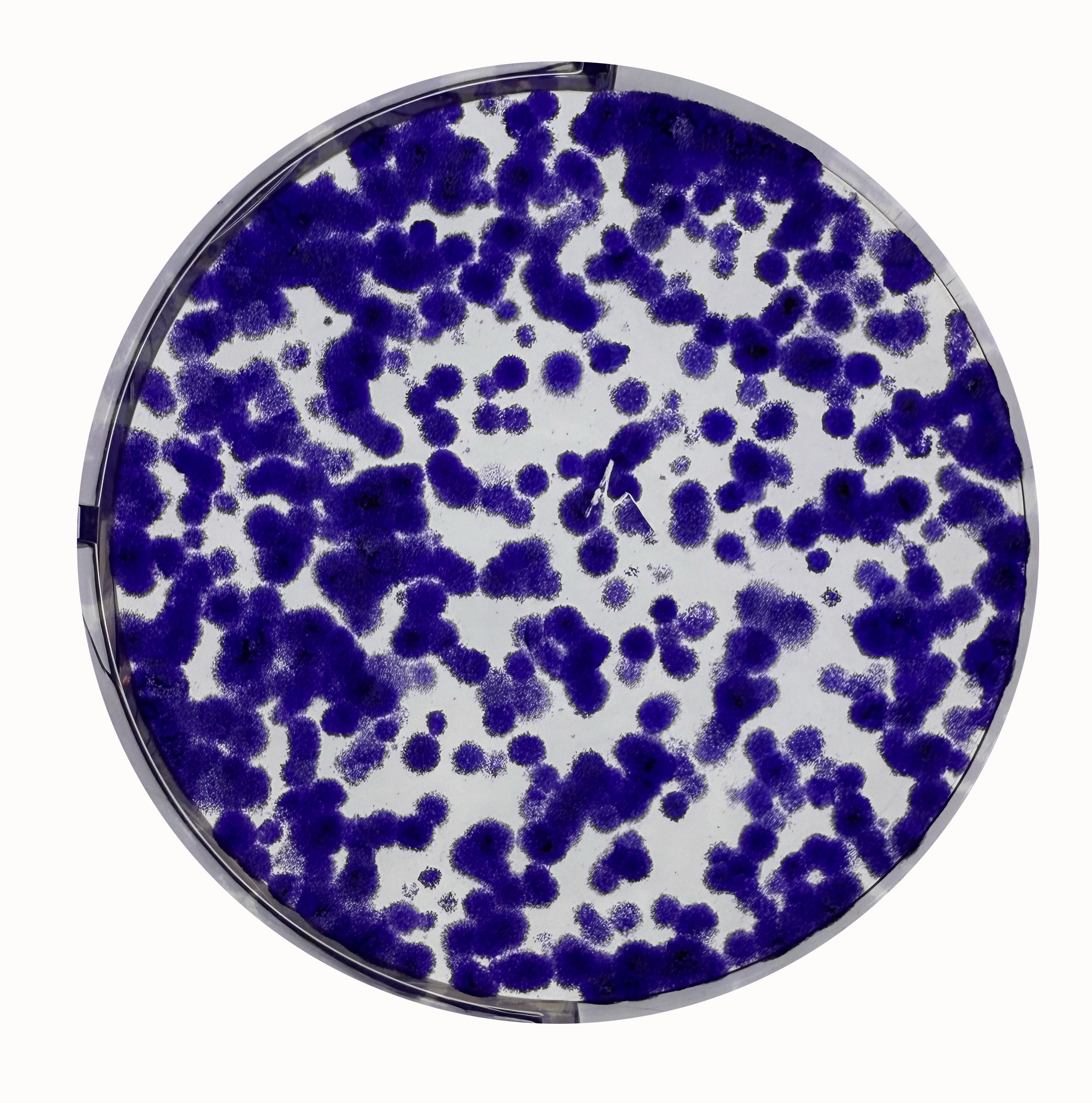

Supplement: Supplementary file 7 — Source data Fig. 2 [file 44321_2025_333_MOESM7_ESM.zip › Figure 2/2C/CaCO2/Rep 2/2_OE circ.tif]

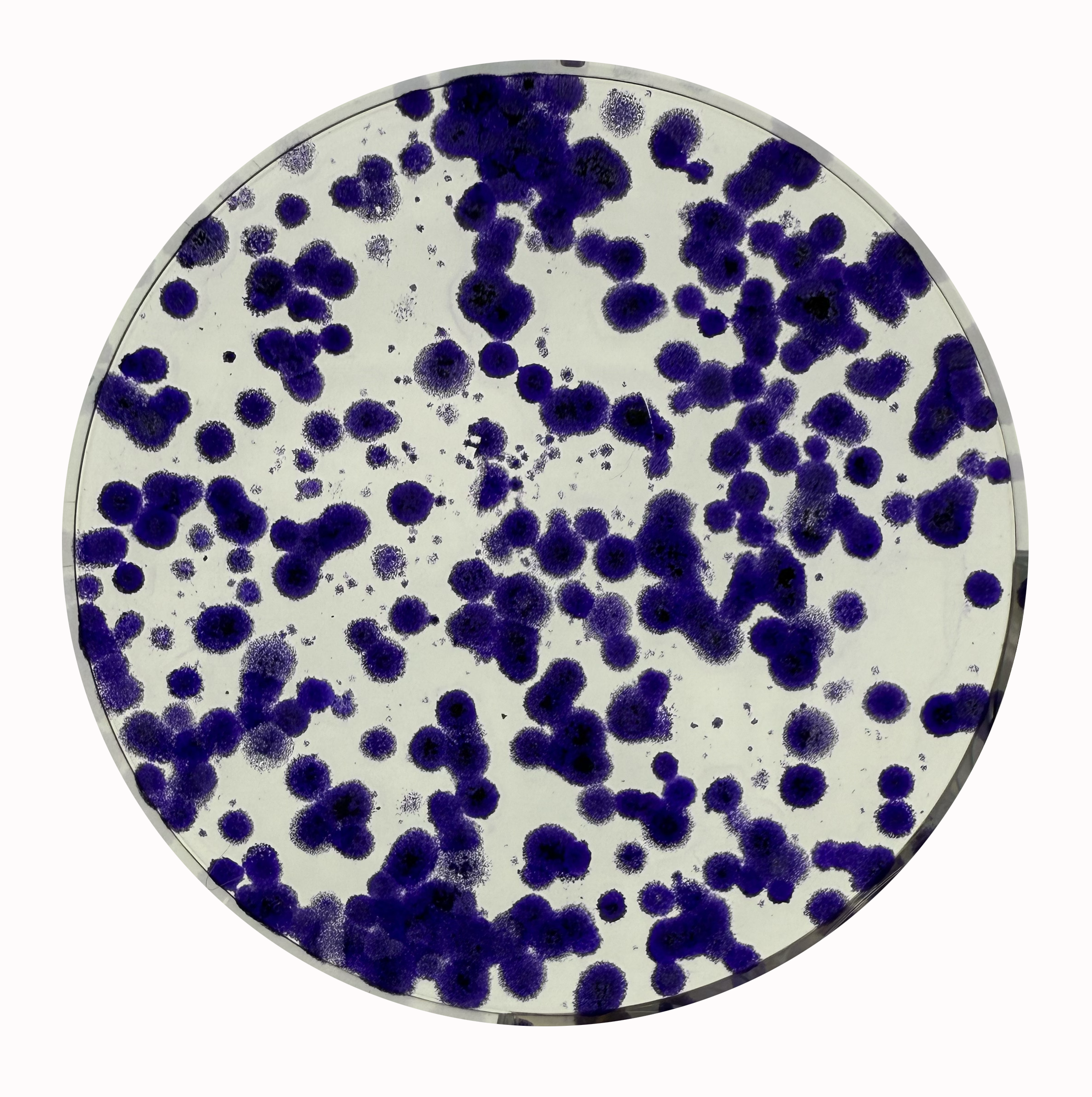

Supplement: Supplementary file 7 — Source data Fig. 2 [file 44321_2025_333_MOESM7_ESM.zip › Figure 2/2C/CaCO2/Rep 2/3_OE+NC.tif]

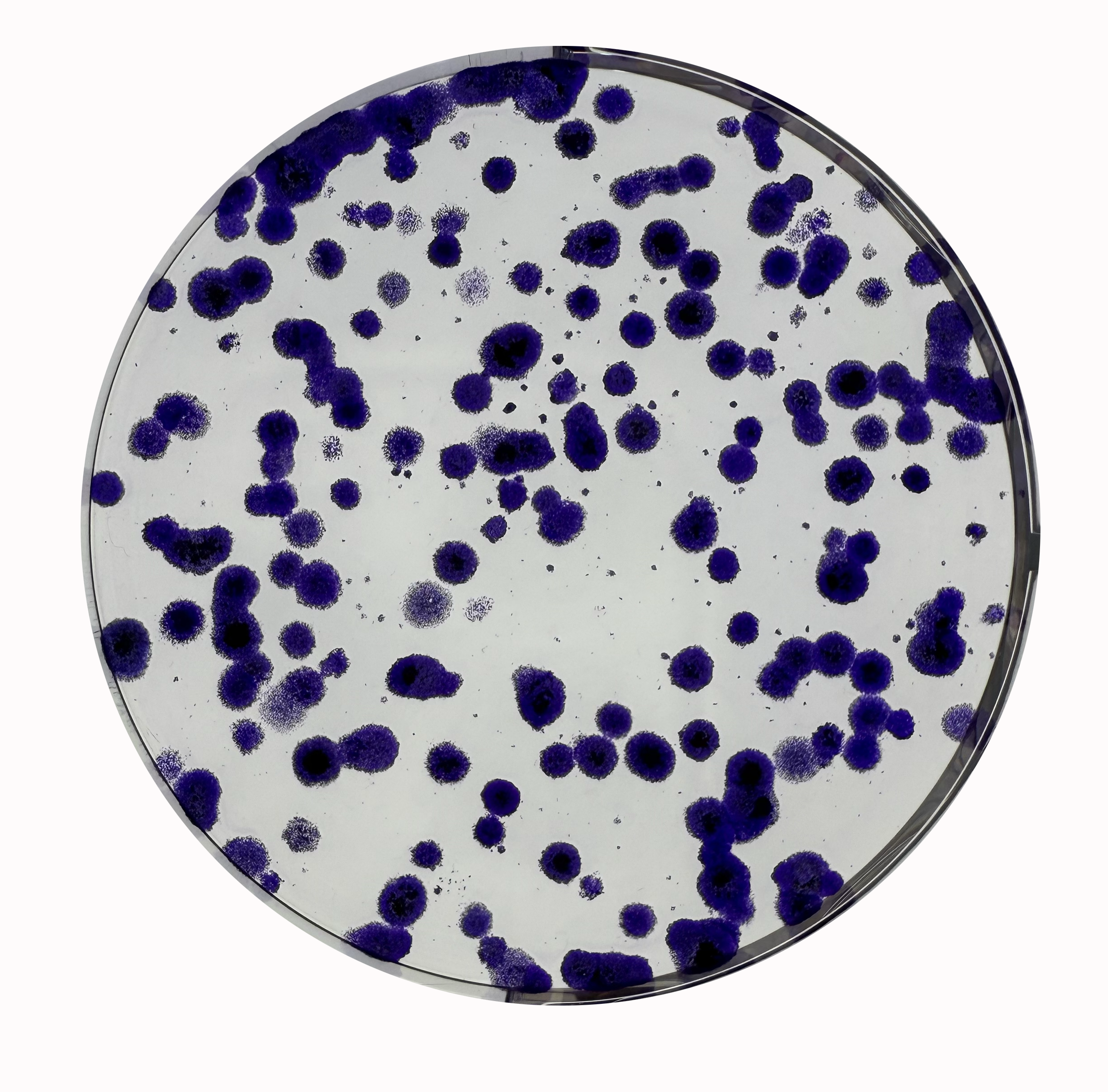

Supplement: Supplementary file 7 — Source data Fig. 2 [file 44321_2025_333_MOESM7_ESM.zip › Figure 2/2C/CaCO2/Rep 2/4_OE+OE.tif]

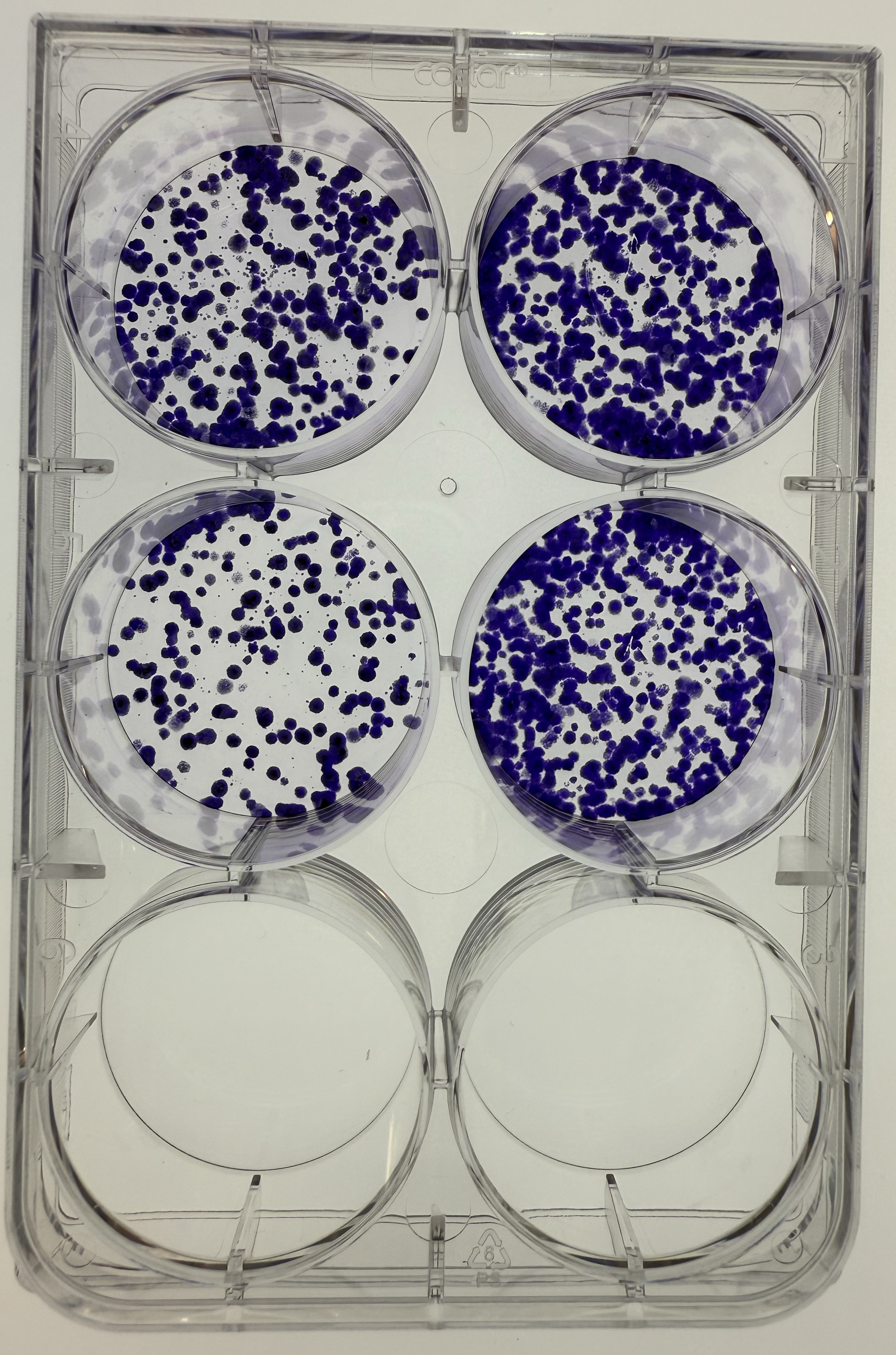

Supplement: Supplementary file 7 — Source data Fig. 2 [file 44321_2025_333_MOESM7_ESM.zip › Figure 2/2C/CaCO2/Rep 2/overall.tif]

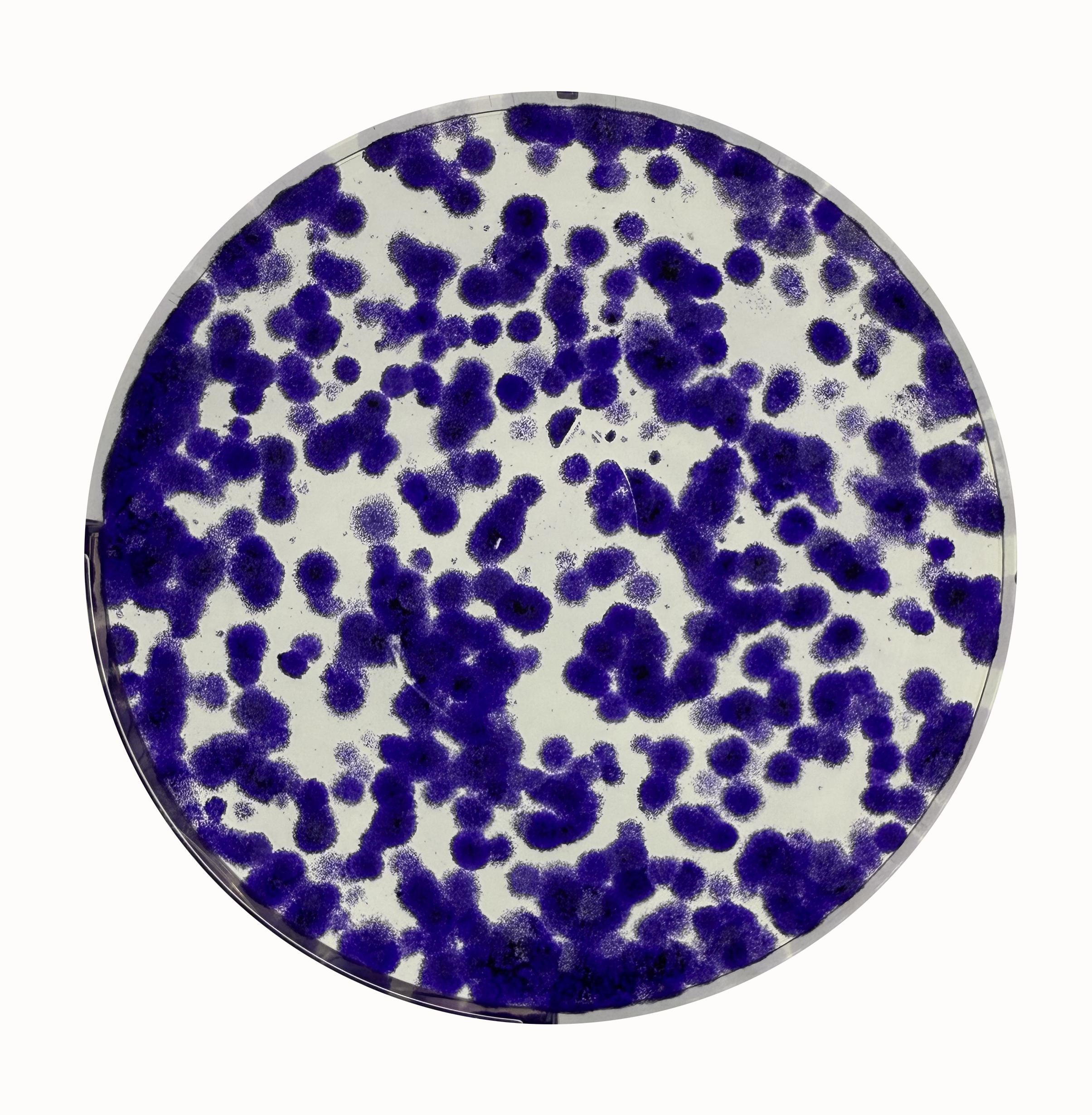

Supplement: Supplementary file 7 — Source data Fig. 2 [file 44321_2025_333_MOESM7_ESM.zip › Figure 2/2C/CaCO2/Rep 3/1_NC.tif]

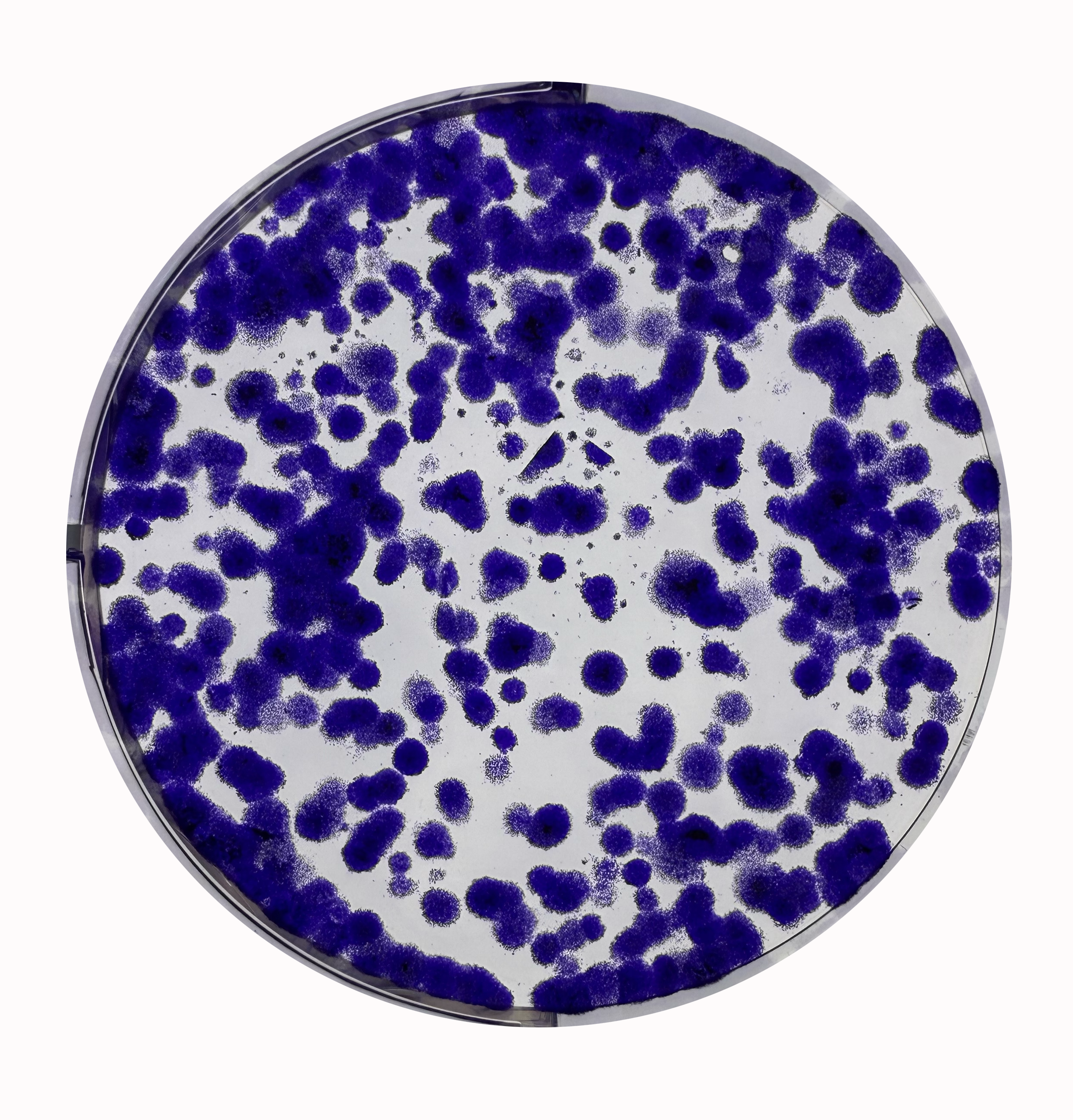

Supplement: Supplementary file 7 — Source data Fig. 2 [file 44321_2025_333_MOESM7_ESM.zip › Figure 2/2C/CaCO2/Rep 3/2_OE circ.tif]

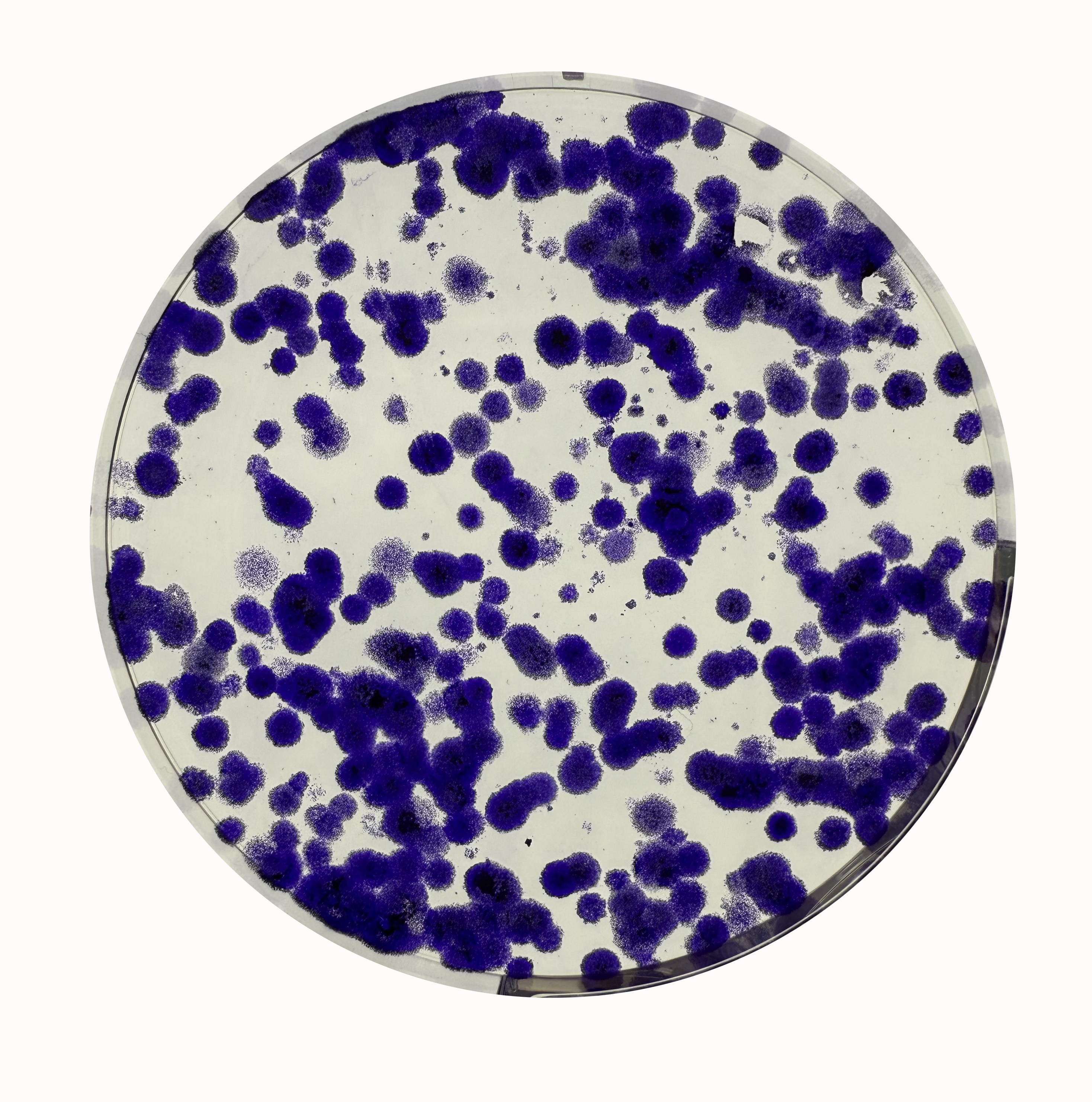

Supplement: Supplementary file 7 — Source data Fig. 2 [file 44321_2025_333_MOESM7_ESM.zip › Figure 2/2C/CaCO2/Rep 3/3_OE+NC.tif]

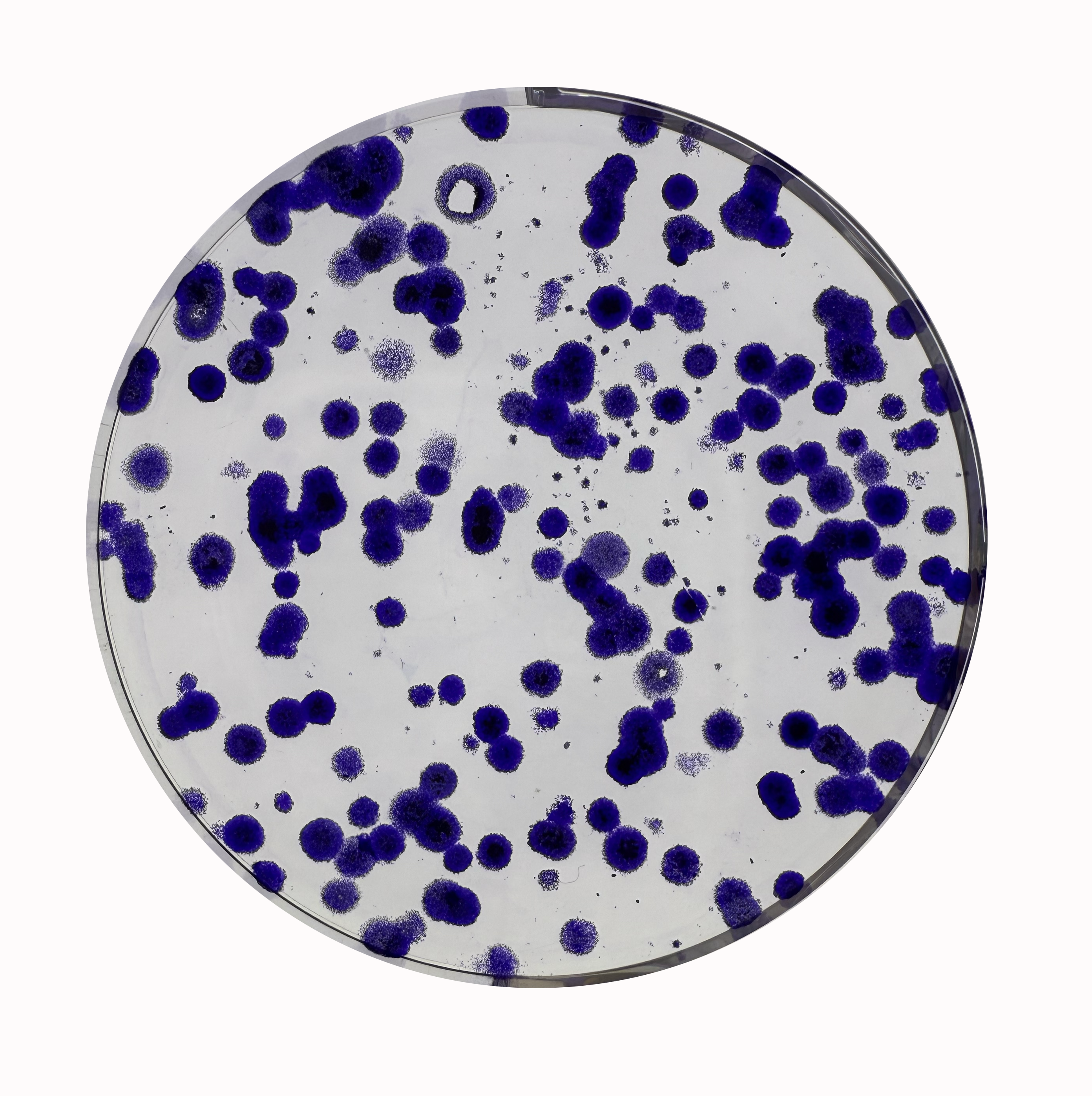

Supplement: Supplementary file 7 — Source data Fig. 2 [file 44321_2025_333_MOESM7_ESM.zip › Figure 2/2C/CaCO2/Rep 3/4_OE+OE.tif]

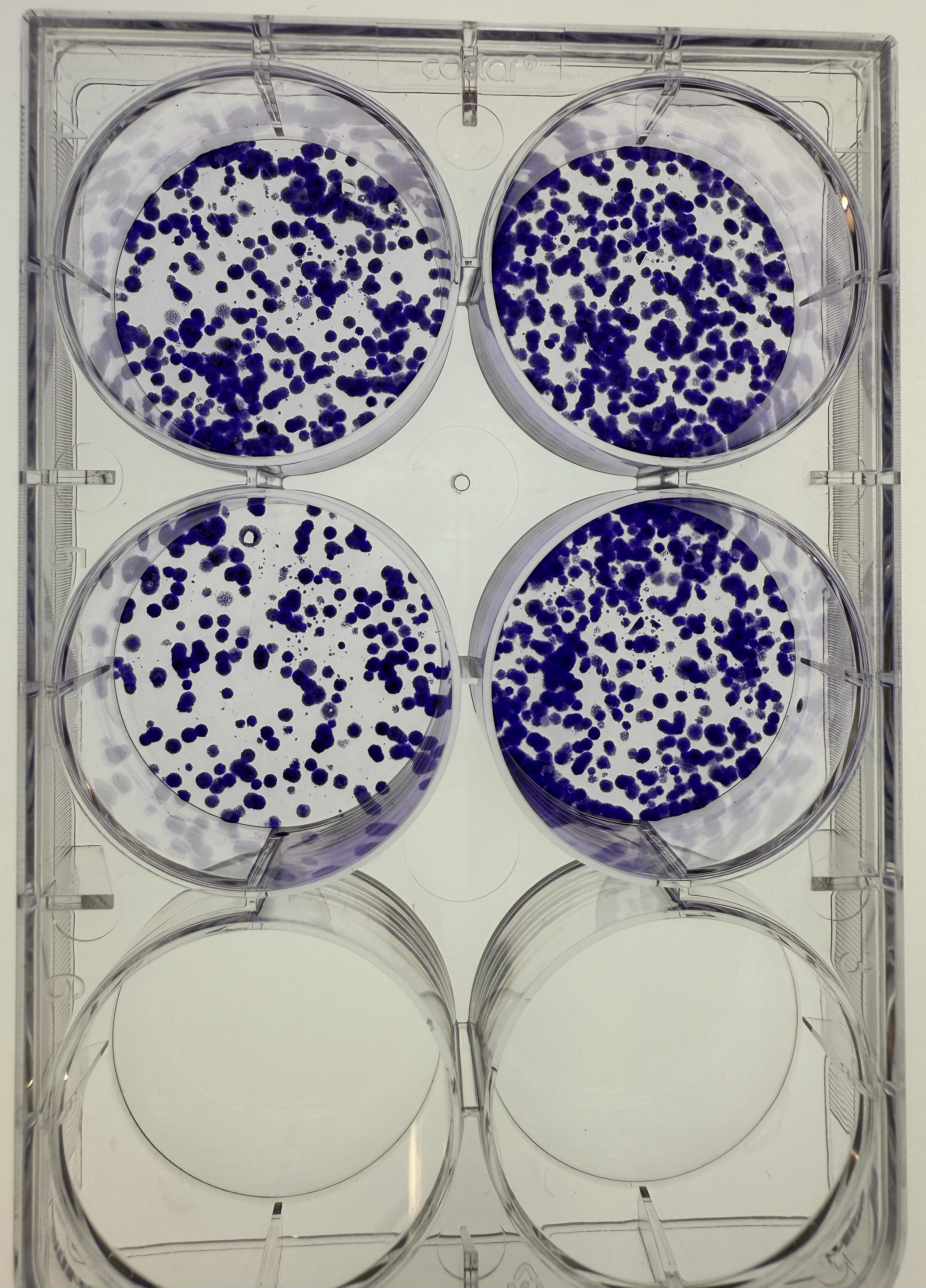

Supplement: Supplementary file 7 — Source data Fig. 2 [file 44321_2025_333_MOESM7_ESM.zip › Figure 2/2C/CaCO2/Rep 3/overall.tif]

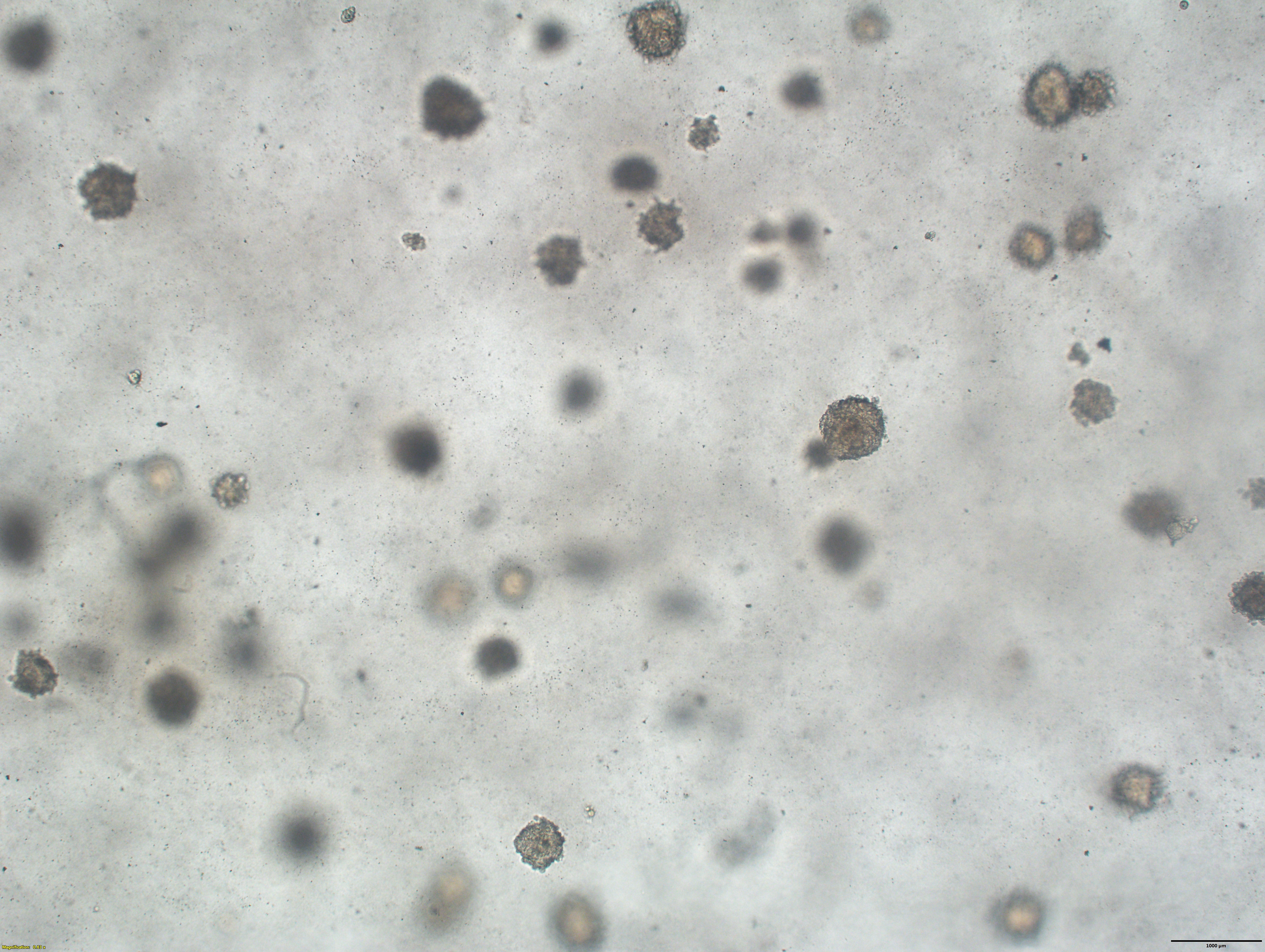

Supplement: Supplementary file 7 — Source data Fig. 2 [file 44321_2025_333_MOESM7_ESM.zip › Figure 2/2C/SNU-C1/Rep 1/1_EV_no drug.jpg]

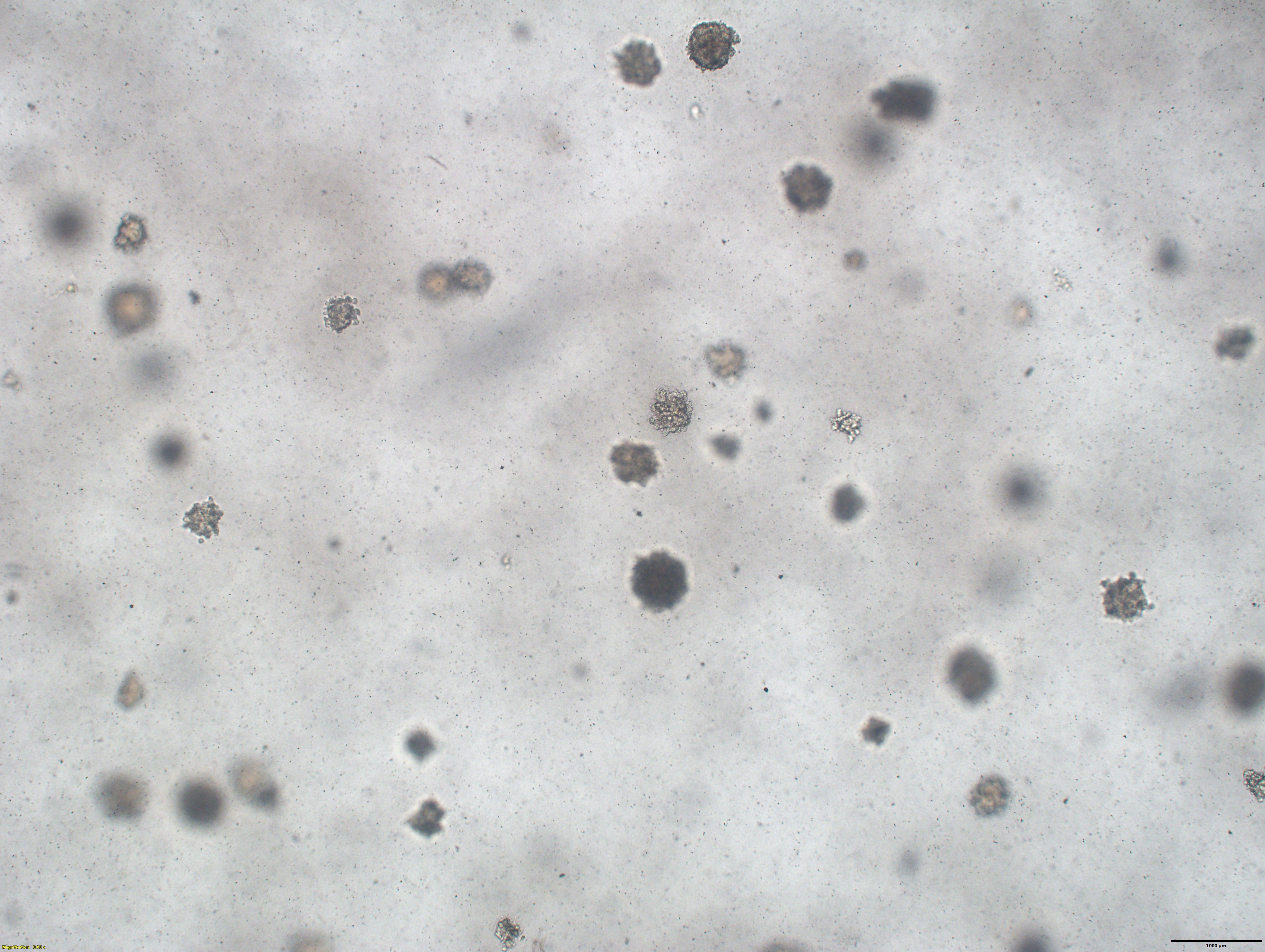

Supplement: Supplementary file 7 — Source data Fig. 2 [file 44321_2025_333_MOESM7_ESM.zip › Figure 2/2C/SNU-C1/Rep 1/2_OE_no drug.jpg]

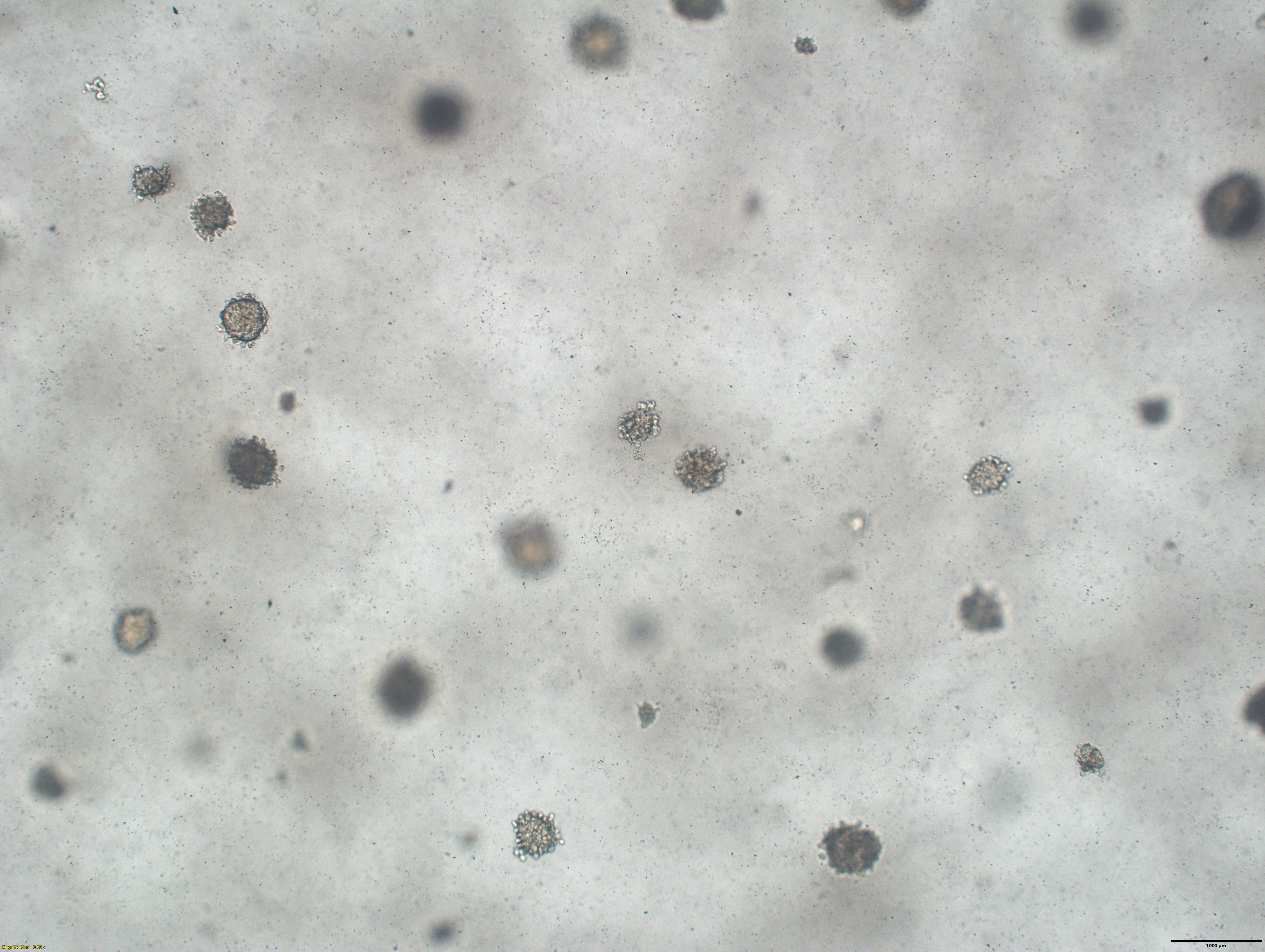

Supplement: Supplementary file 7 — Source data Fig. 2 [file 44321_2025_333_MOESM7_ESM.zip › Figure 2/2C/SNU-C1/Rep 1/3_EV_cetuximab.jpg]

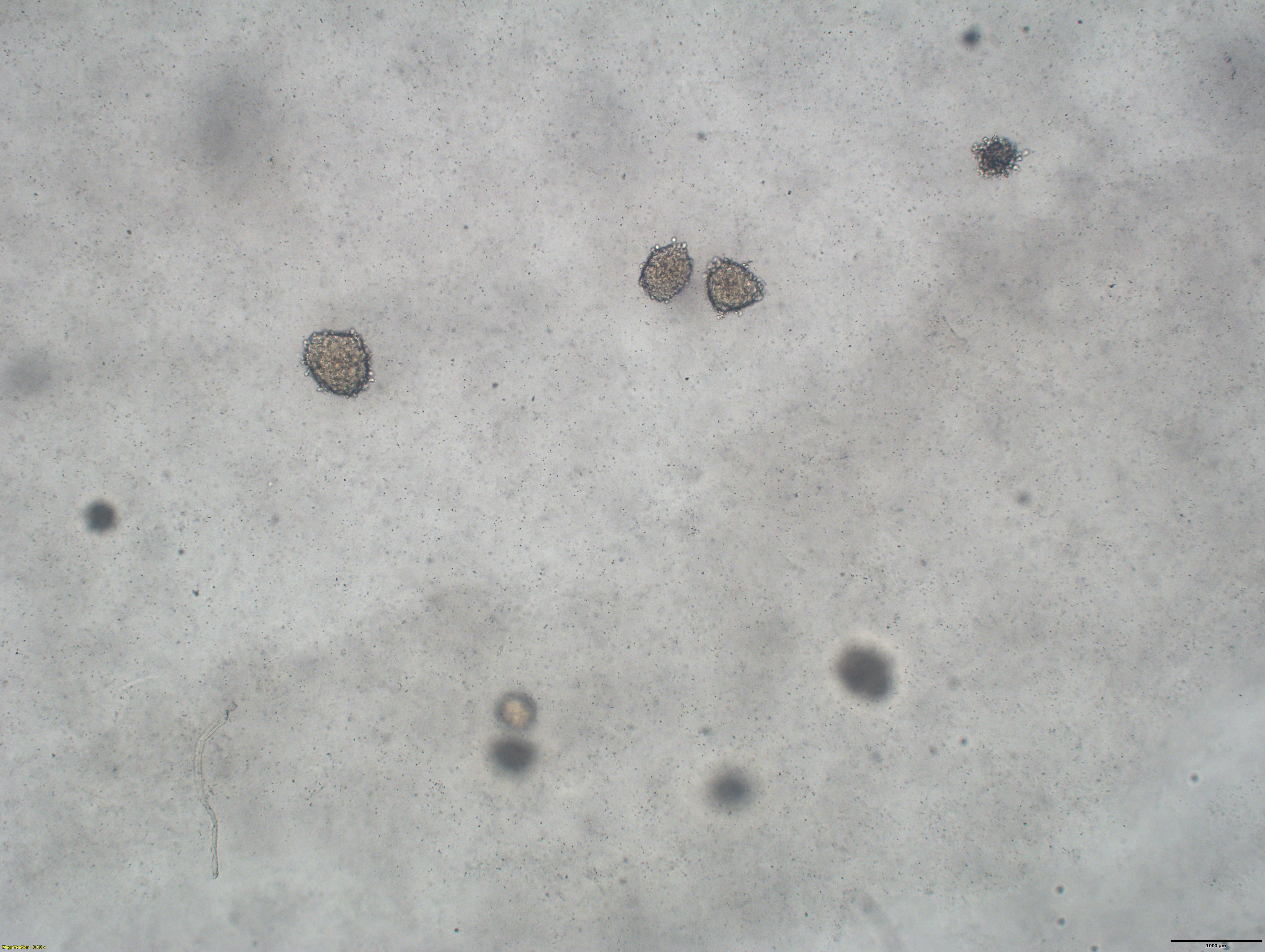

Supplement: Supplementary file 7 — Source data Fig. 2 [file 44321_2025_333_MOESM7_ESM.zip › Figure 2/2C/SNU-C1/Rep 1/4_OE_cetuximab.jpg]

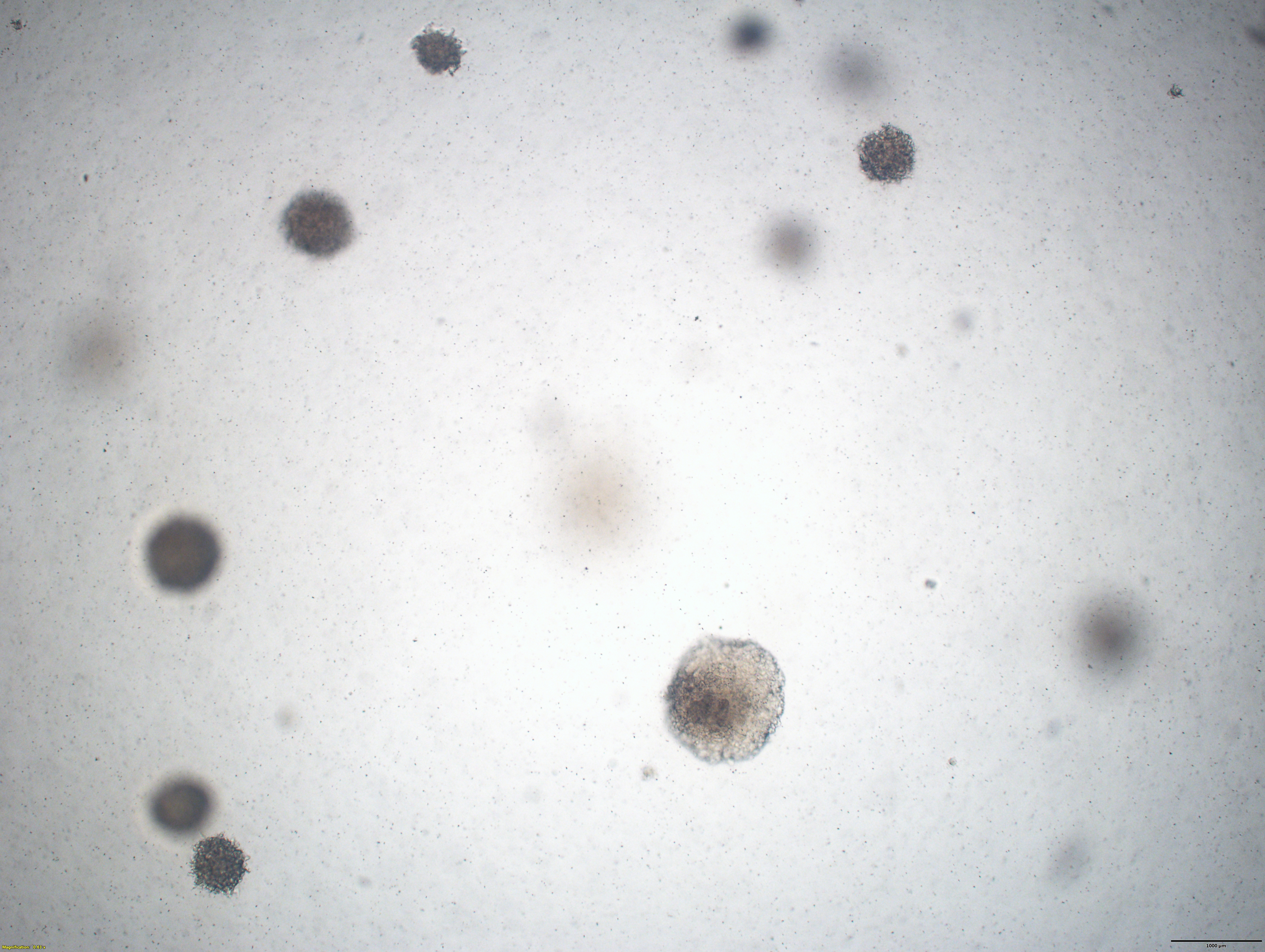

Supplement: Supplementary file 7 — Source data Fig. 2 [file 44321_2025_333_MOESM7_ESM.zip › Figure 2/2C/SNU-C1/Rep 2/1_EV_no drug.jpg]

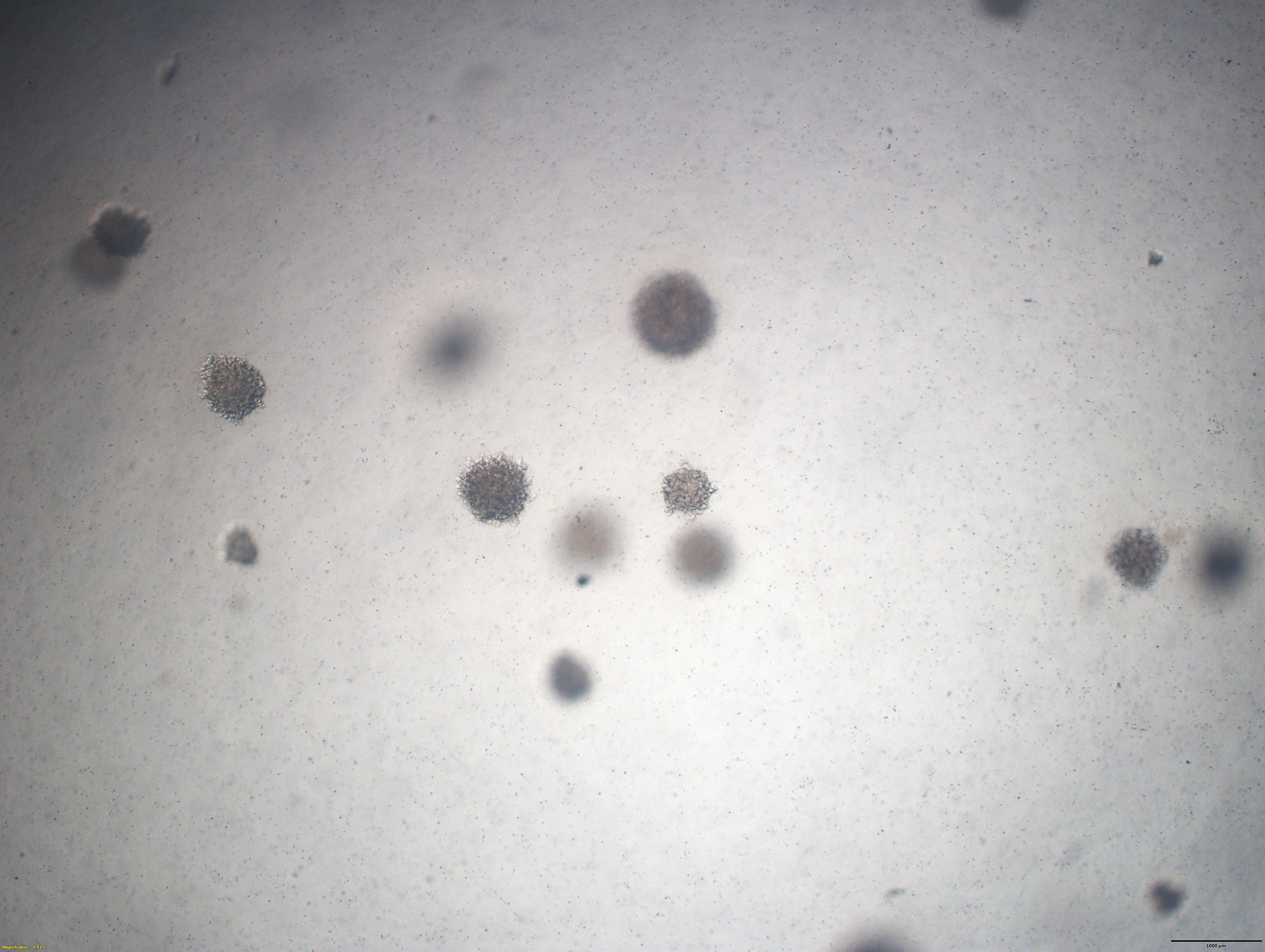

Supplement: Supplementary file 7 — Source data Fig. 2 [file 44321_2025_333_MOESM7_ESM.zip › Figure 2/2C/SNU-C1/Rep 2/2_OE_no drug.jpg]

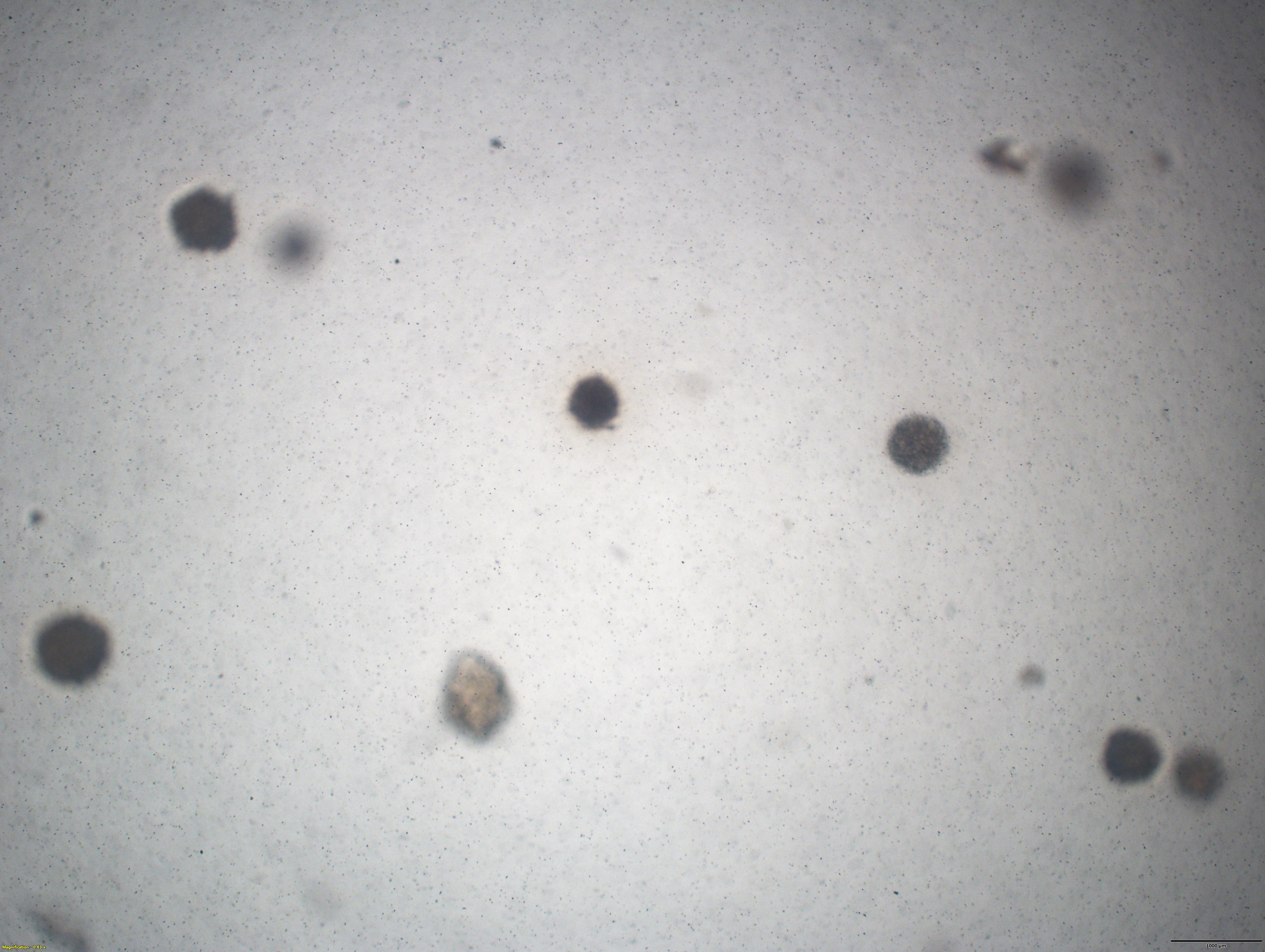

Supplement: Supplementary file 7 — Source data Fig. 2 [file 44321_2025_333_MOESM7_ESM.zip › Figure 2/2C/SNU-C1/Rep 2/3_EV_cetuximab.jpg]

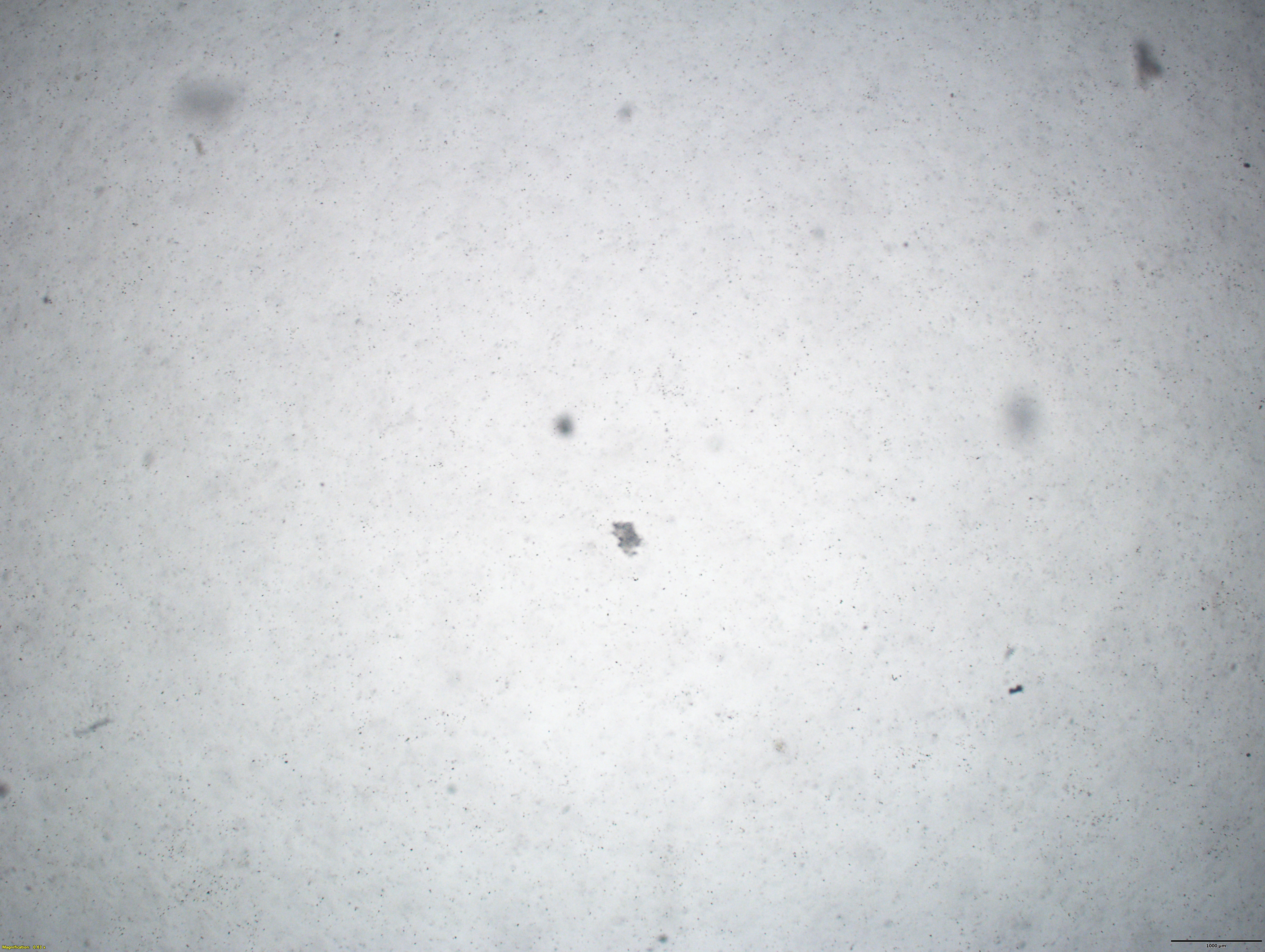

Supplement: Supplementary file 7 — Source data Fig. 2 [file 44321_2025_333_MOESM7_ESM.zip › Figure 2/2C/SNU-C1/Rep 2/4_OE_cetuximab.jpg]

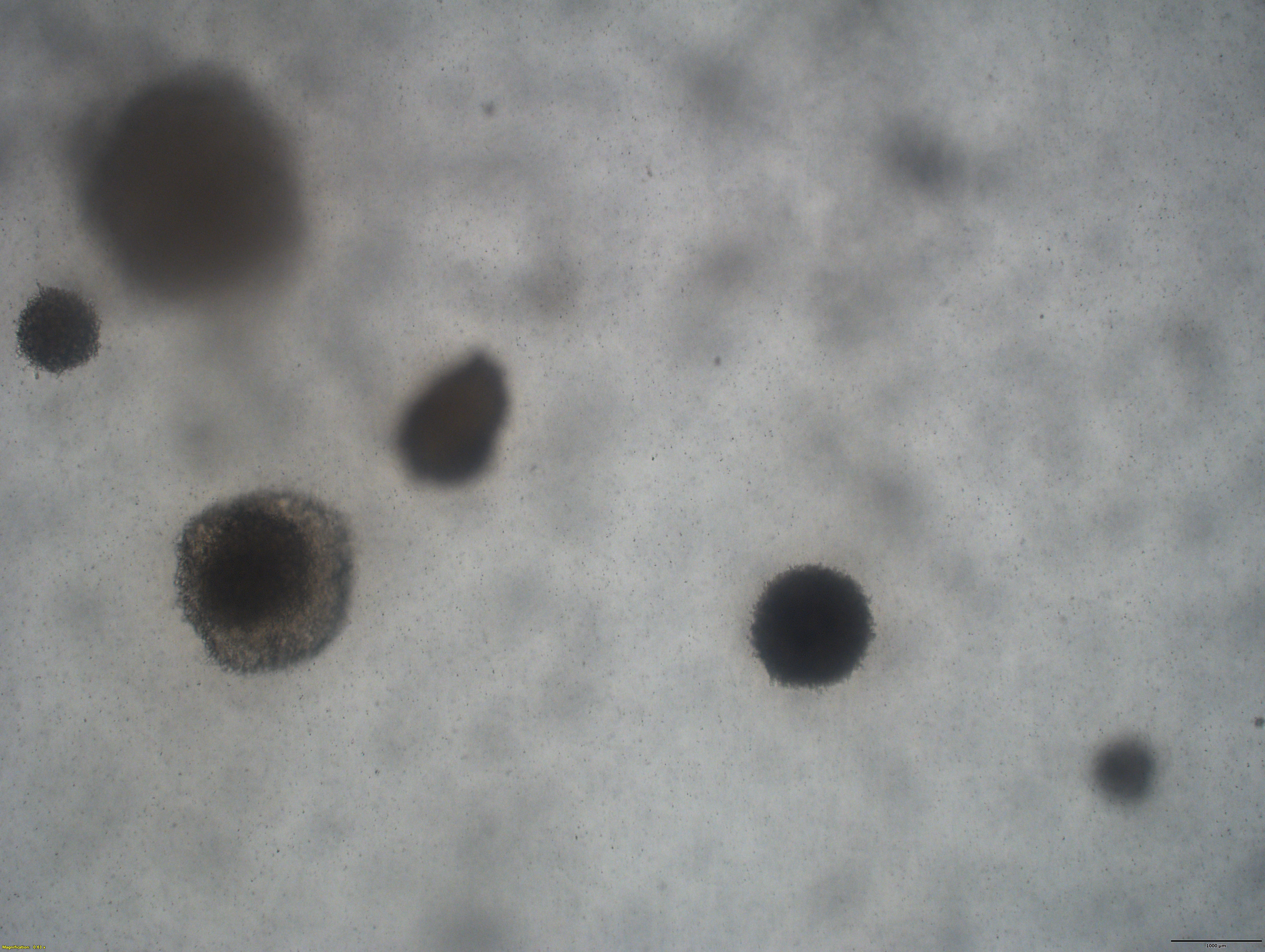

Supplement: Supplementary file 7 — Source data Fig. 2 [file 44321_2025_333_MOESM7_ESM.zip › Figure 2/2C/SNU-C1/Rep 3/1_EV_no drug.jpg]

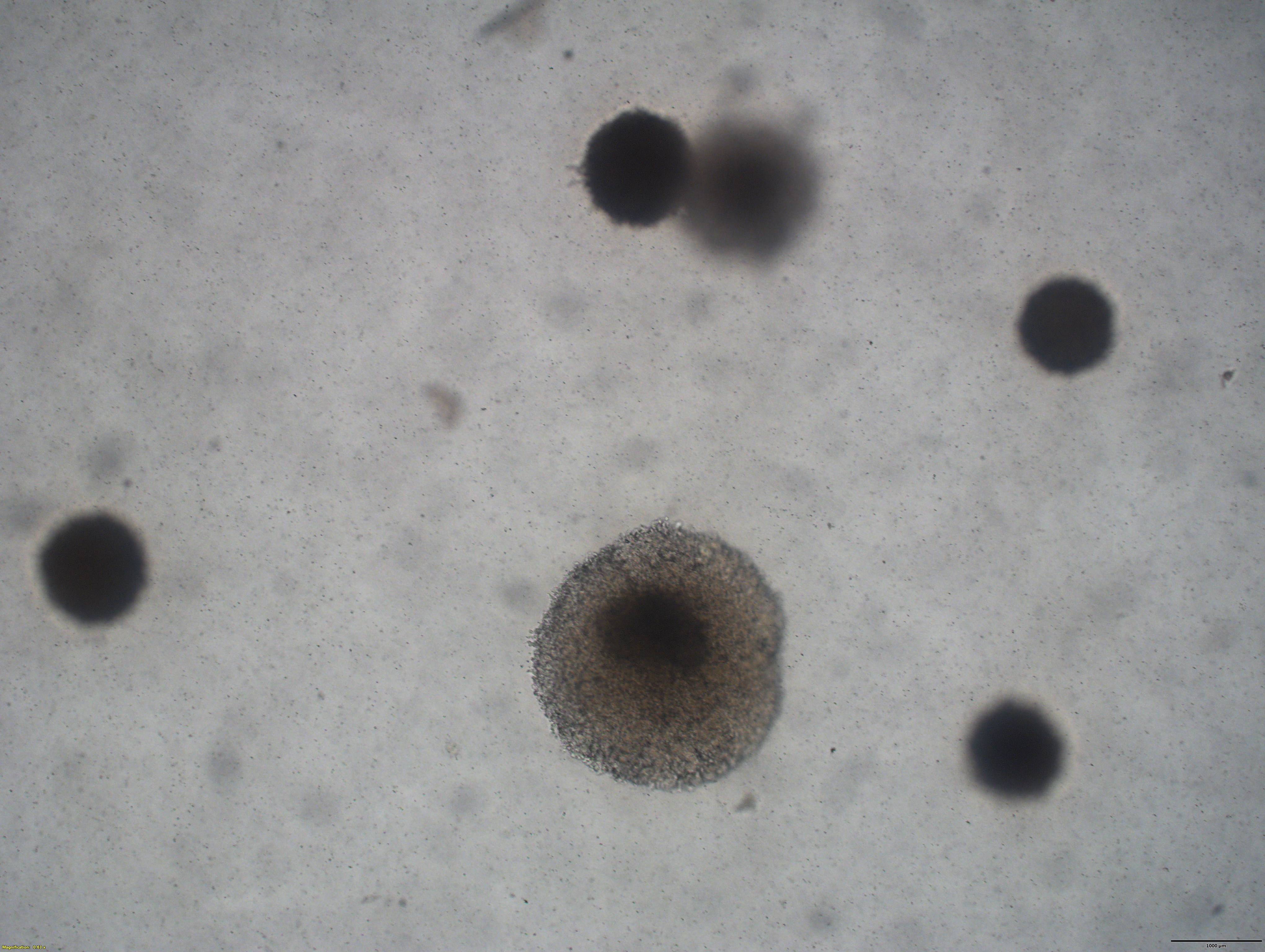

Supplement: Supplementary file 7 — Source data Fig. 2 [file 44321_2025_333_MOESM7_ESM.zip › Figure 2/2C/SNU-C1/Rep 3/2_OE_no drug.jpg]

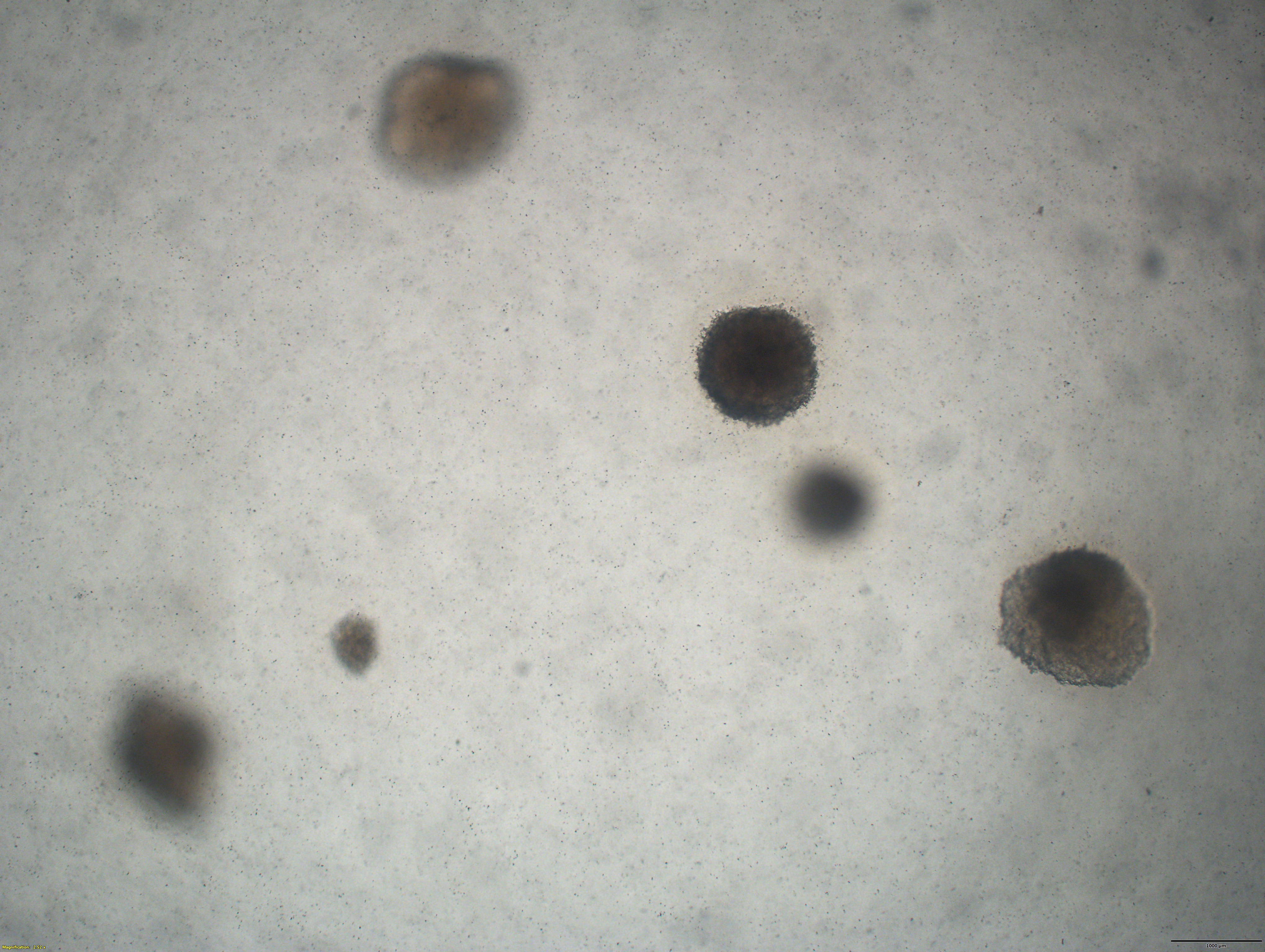

Supplement: Supplementary file 7 — Source data Fig. 2 [file 44321_2025_333_MOESM7_ESM.zip › Figure 2/2C/SNU-C1/Rep 3/3_EV_cetuximab.jpg]

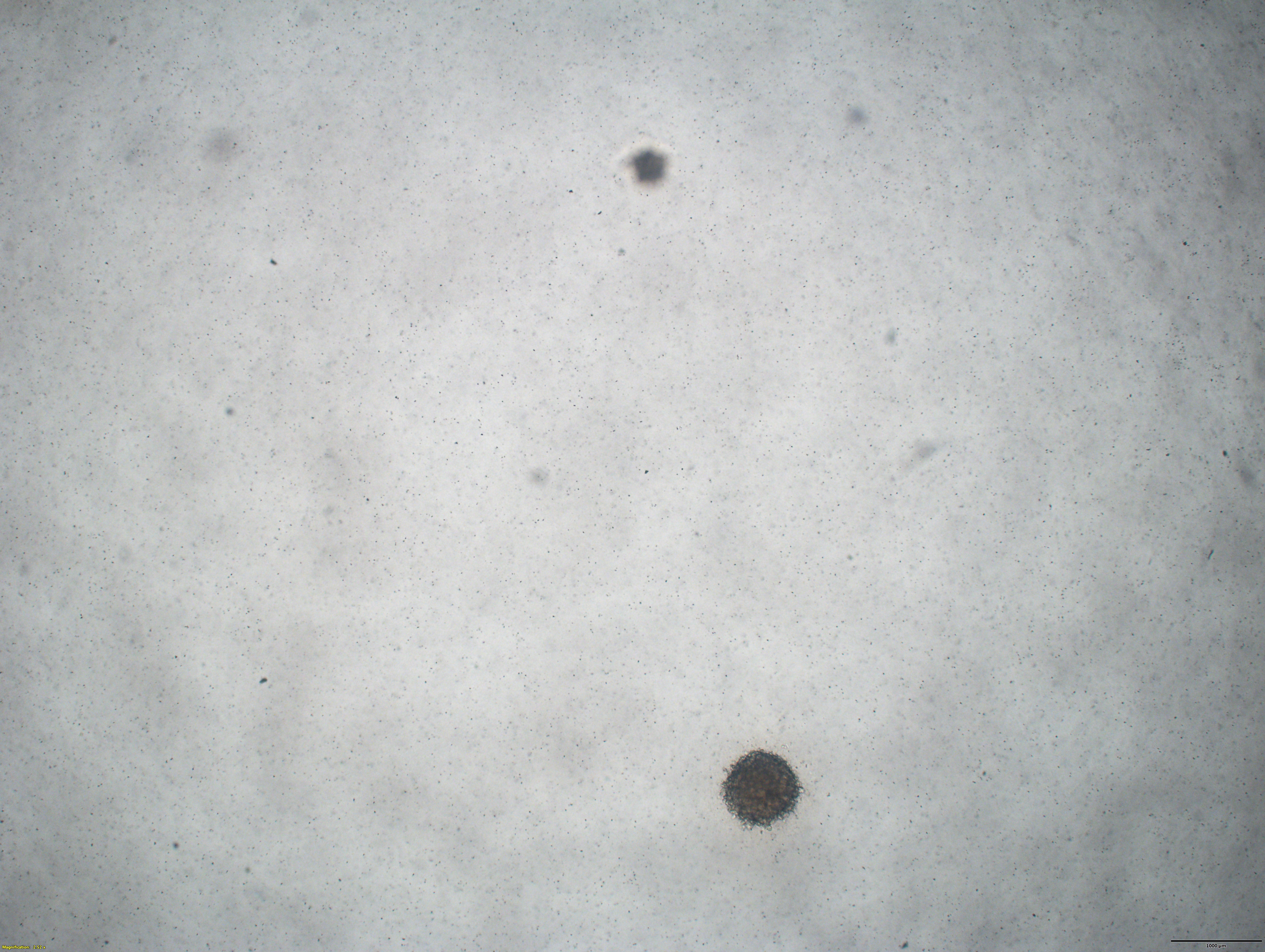

Supplement: Supplementary file 7 — Source data Fig. 2 [file 44321_2025_333_MOESM7_ESM.zip › Figure 2/2C/SNU-C1/Rep 3/4_OE_cetuximab.jpg]

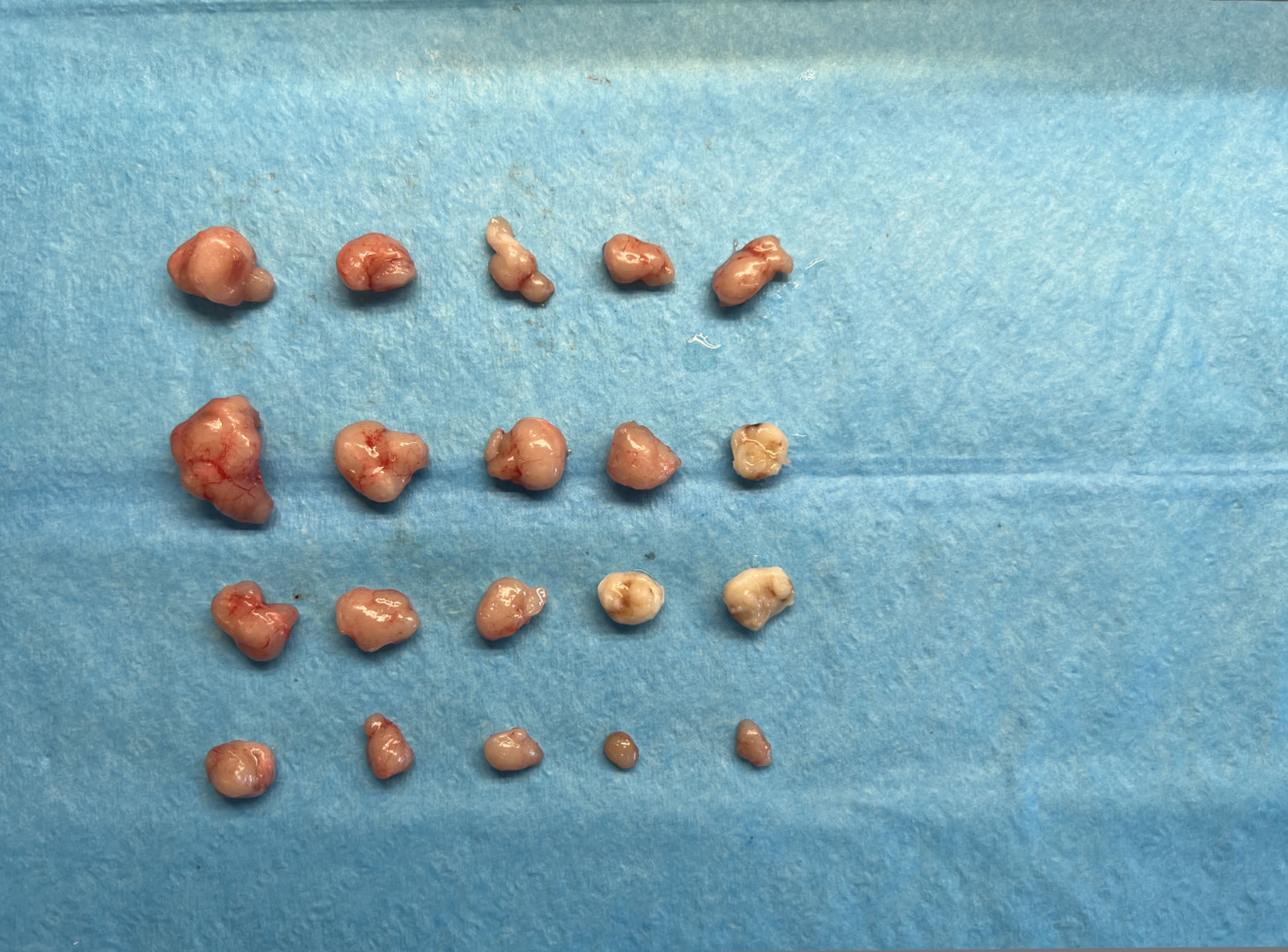

Supplement: Supplementary file 7 — Source data Fig. 2 [file 44321_2025_333_MOESM7_ESM.zip › Figure 2/2G/Figure 2G_mice tumor.tif]

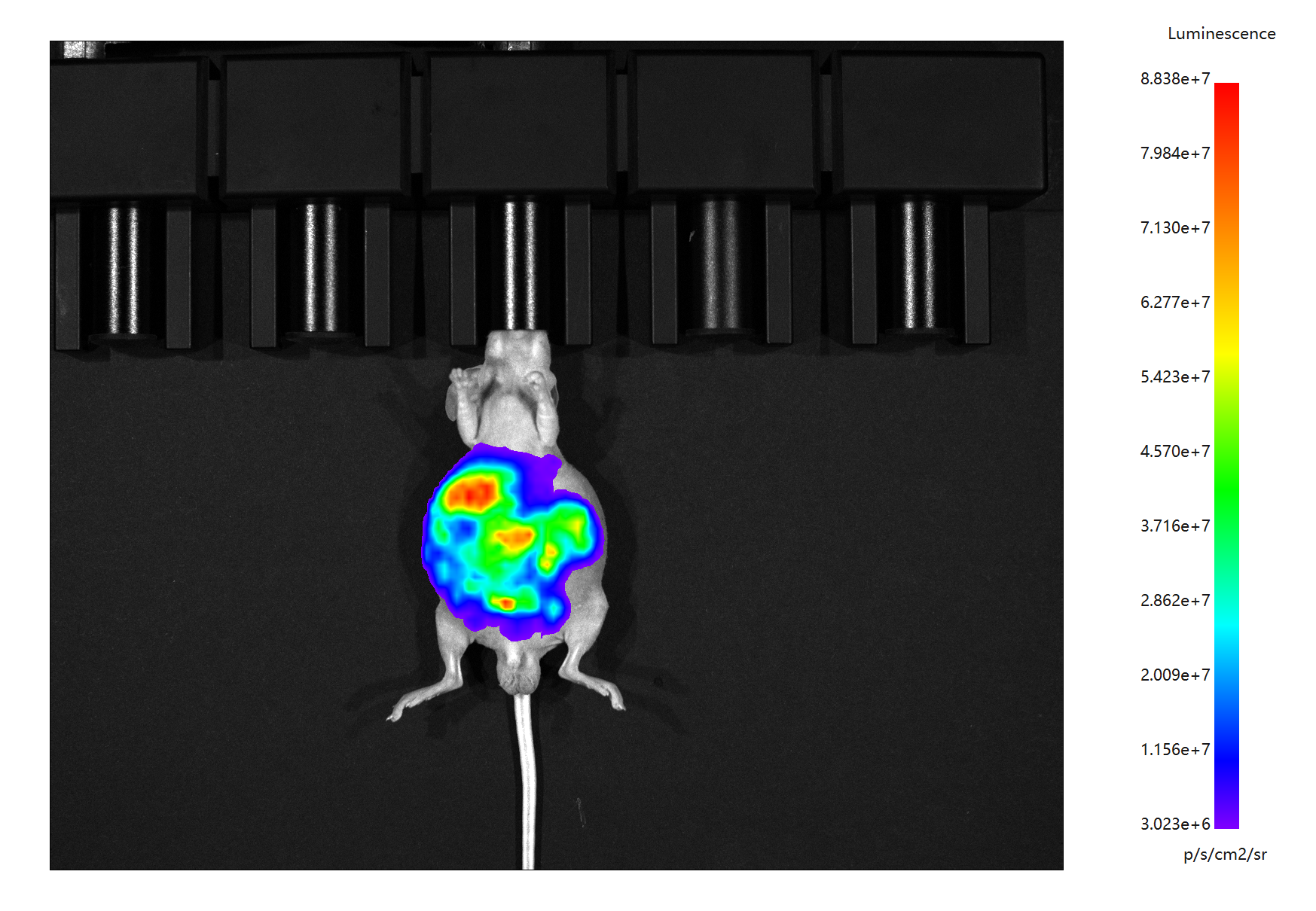

Supplement: Supplementary file 8 — Source data Fig. 3 [file 44321_2025_333_MOESM8_ESM.zip › Figure 3/3A/1. Luc/1.1_Ctrl+Saline.tif]

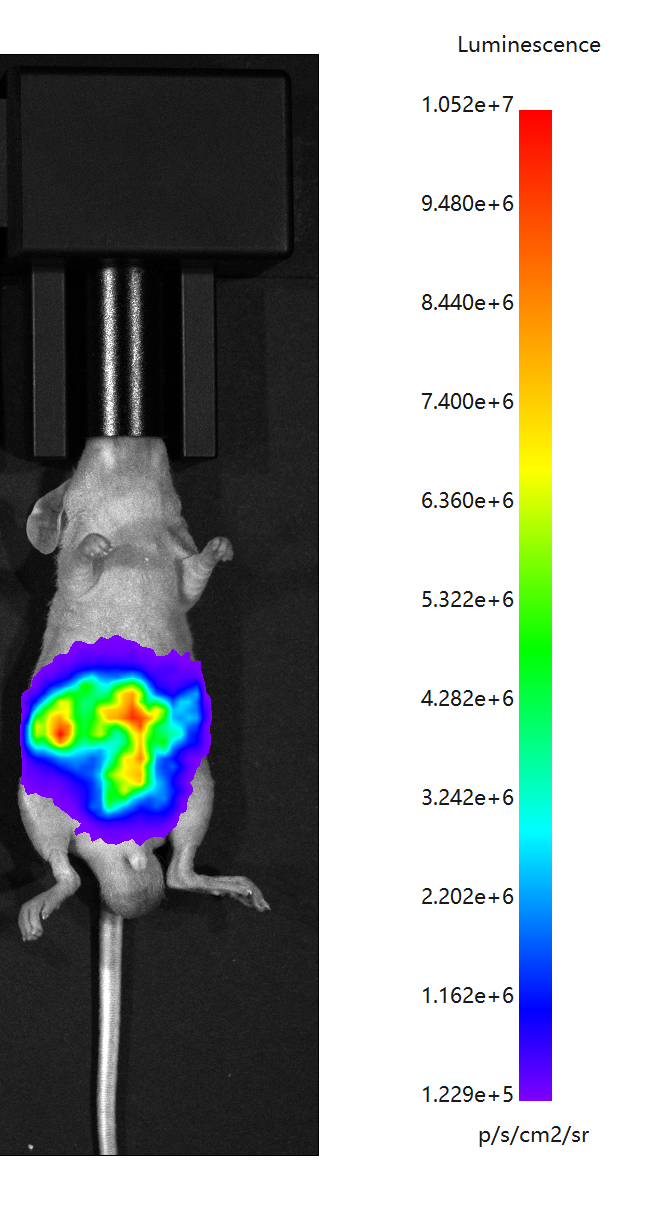

Supplement: Supplementary file 8 — Source data Fig. 3 [file 44321_2025_333_MOESM8_ESM.zip › Figure 3/3A/1. Luc/1.2_Ctrl+Saline.tif]

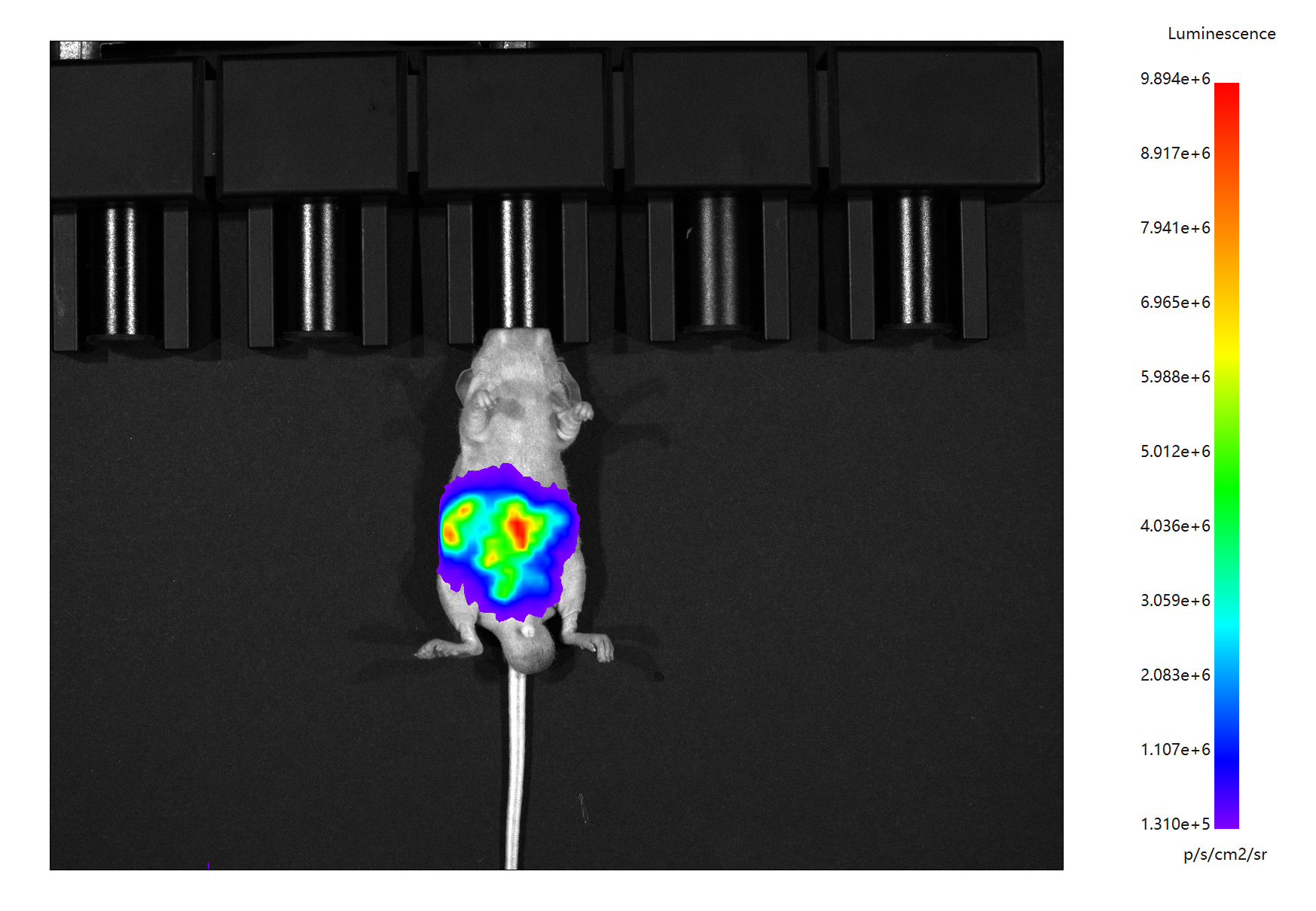

Supplement: Supplementary file 8 — Source data Fig. 3 [file 44321_2025_333_MOESM8_ESM.zip › Figure 3/3A/1. Luc/2.1_OE+Saline.tif]

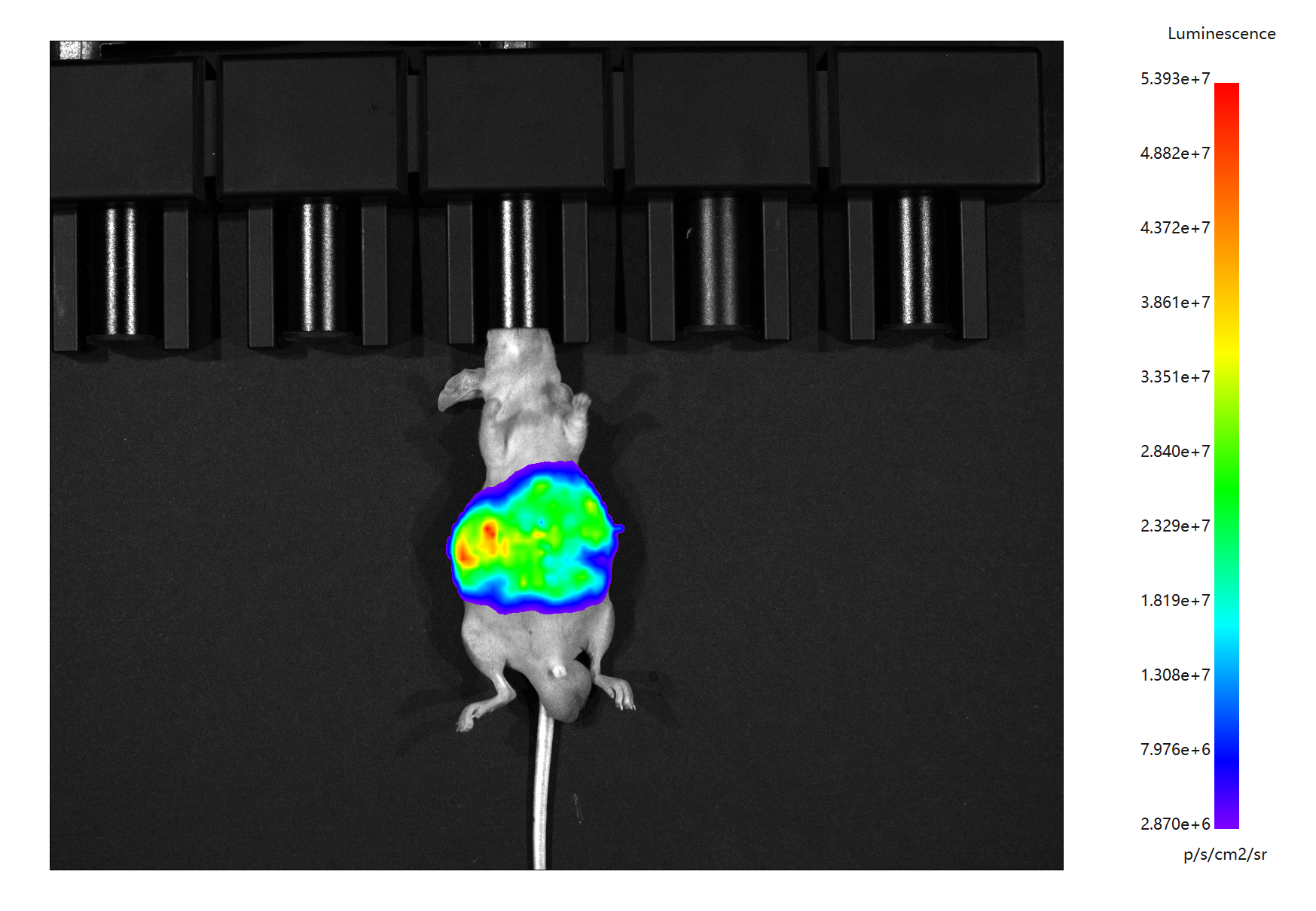

Supplement: Supplementary file 8 — Source data Fig. 3 [file 44321_2025_333_MOESM8_ESM.zip › Figure 3/3A/1. Luc/2.2_OE+Saline.tif]

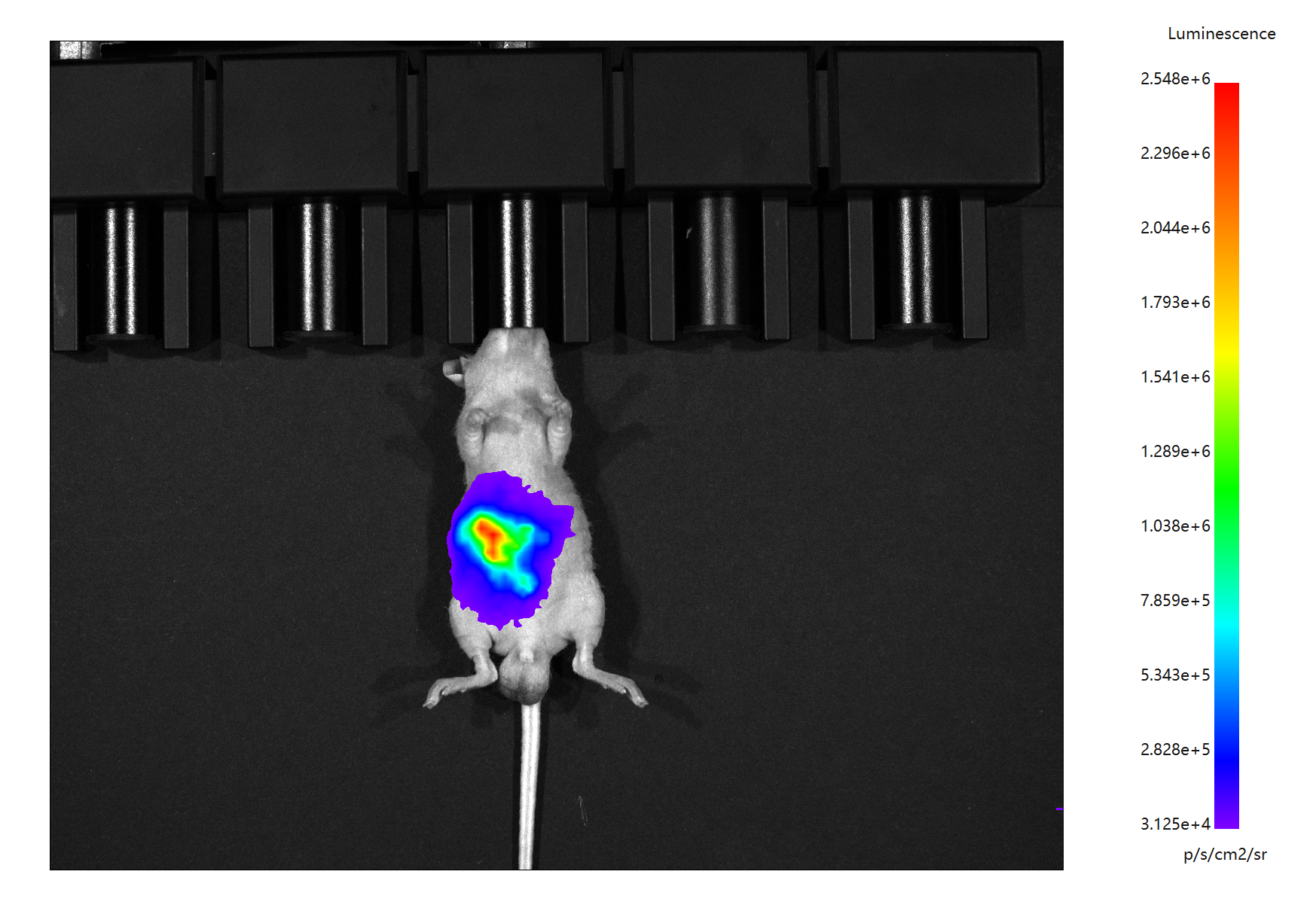

Supplement: Supplementary file 8 — Source data Fig. 3 [file 44321_2025_333_MOESM8_ESM.zip › Figure 3/3A/1. Luc/3.1_Ctrl+Cet.tif]

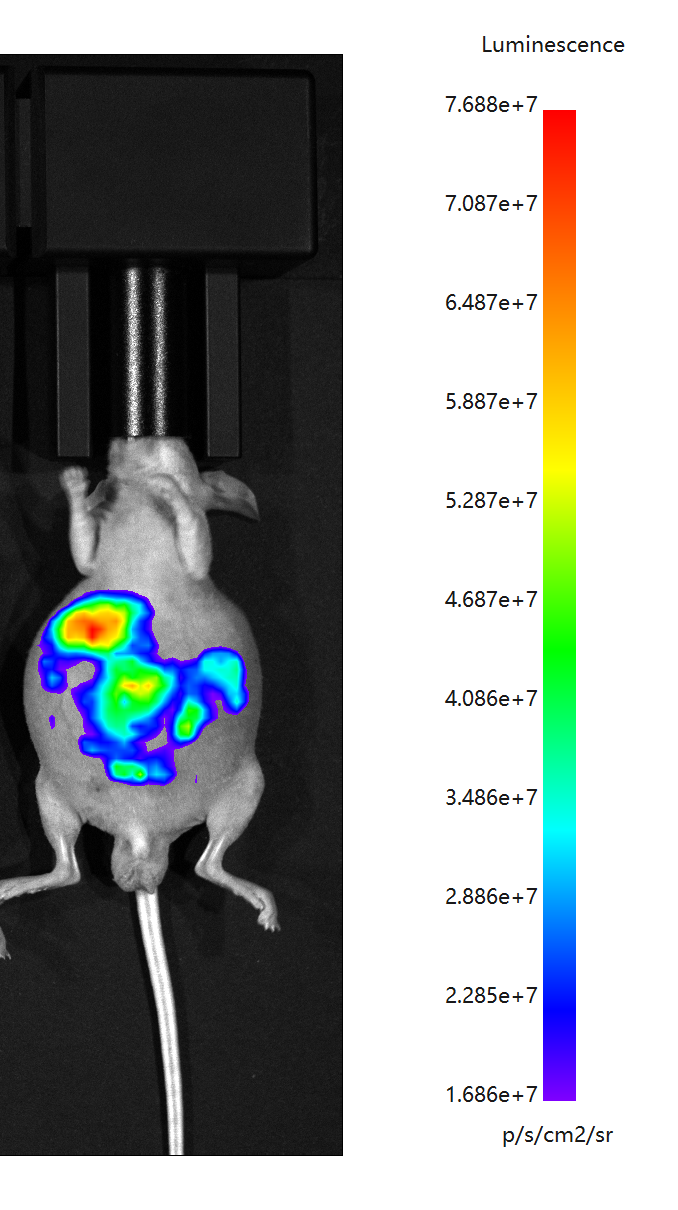

Supplement: Supplementary file 8 — Source data Fig. 3 [file 44321_2025_333_MOESM8_ESM.zip › Figure 3/3A/1. Luc/3.2_Ctrl+Cet.tif]

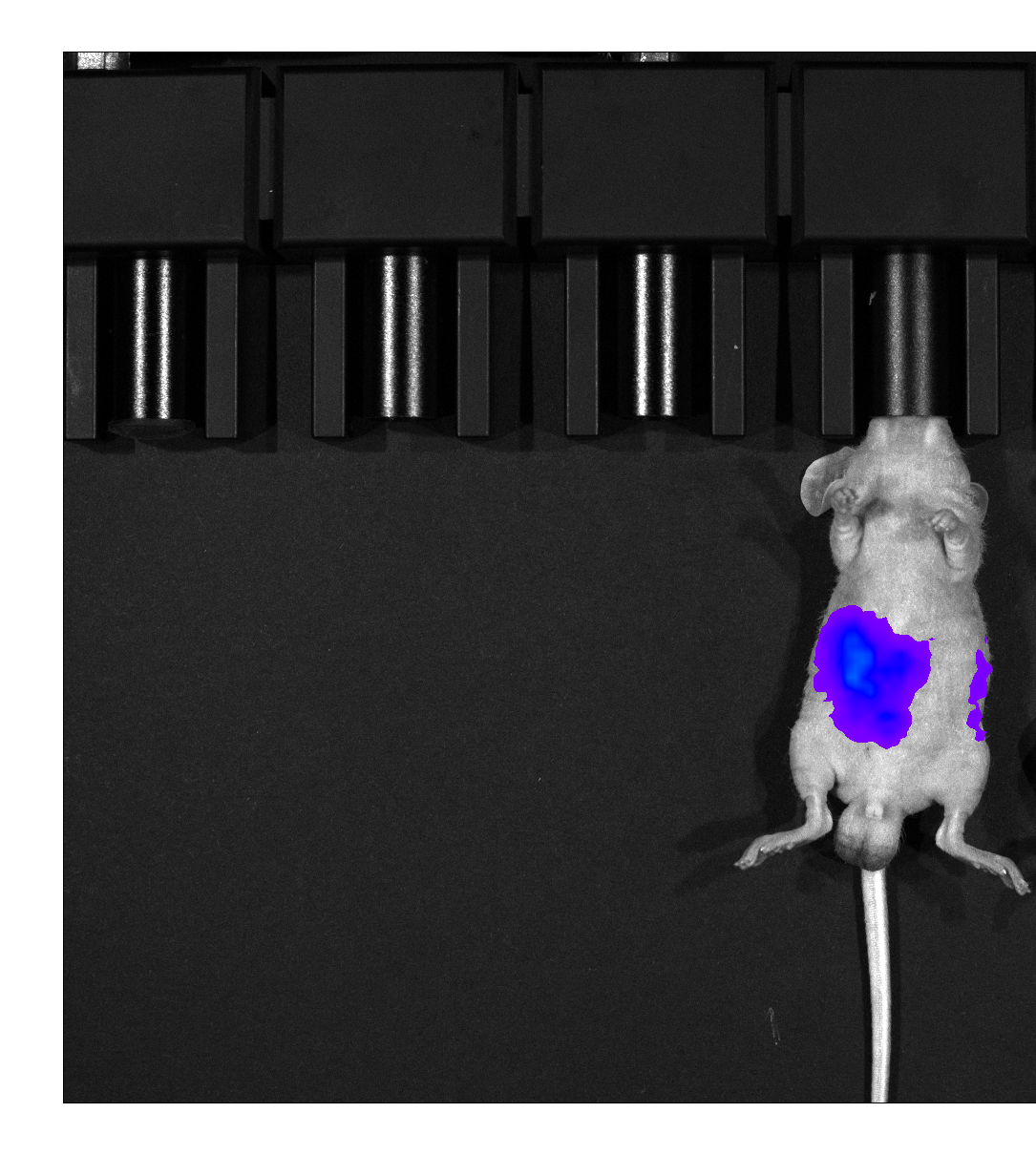

Supplement: Supplementary file 8 — Source data Fig. 3 [file 44321_2025_333_MOESM8_ESM.zip › Figure 3/3A/1. Luc/4.1_OE+Cet.tif]

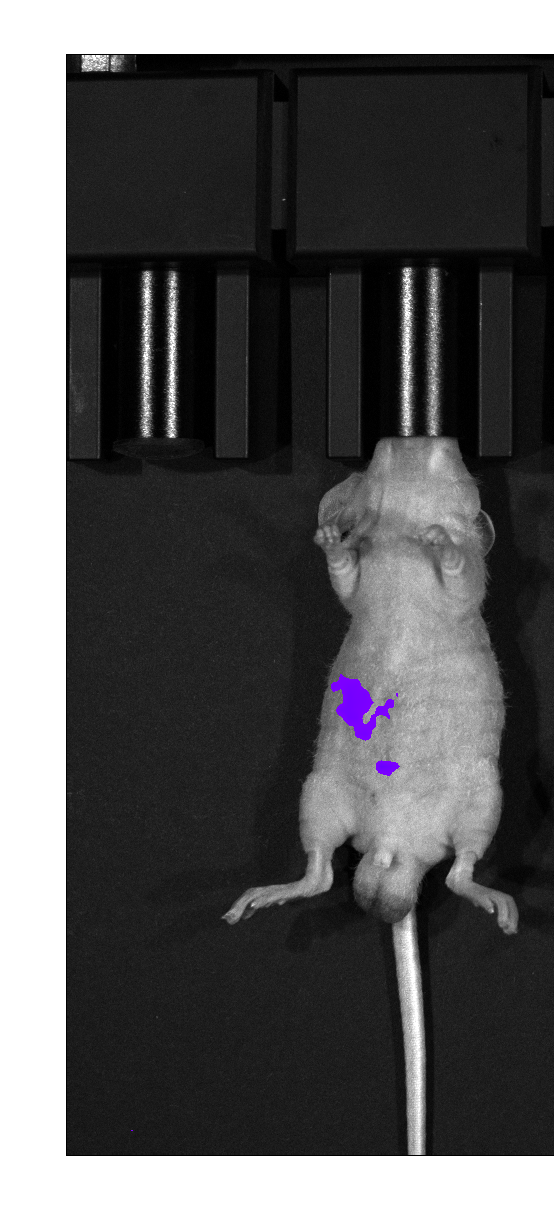

Supplement: Supplementary file 8 — Source data Fig. 3 [file 44321_2025_333_MOESM8_ESM.zip › Figure 3/3A/1. Luc/4.2_OE+Cet.tif]

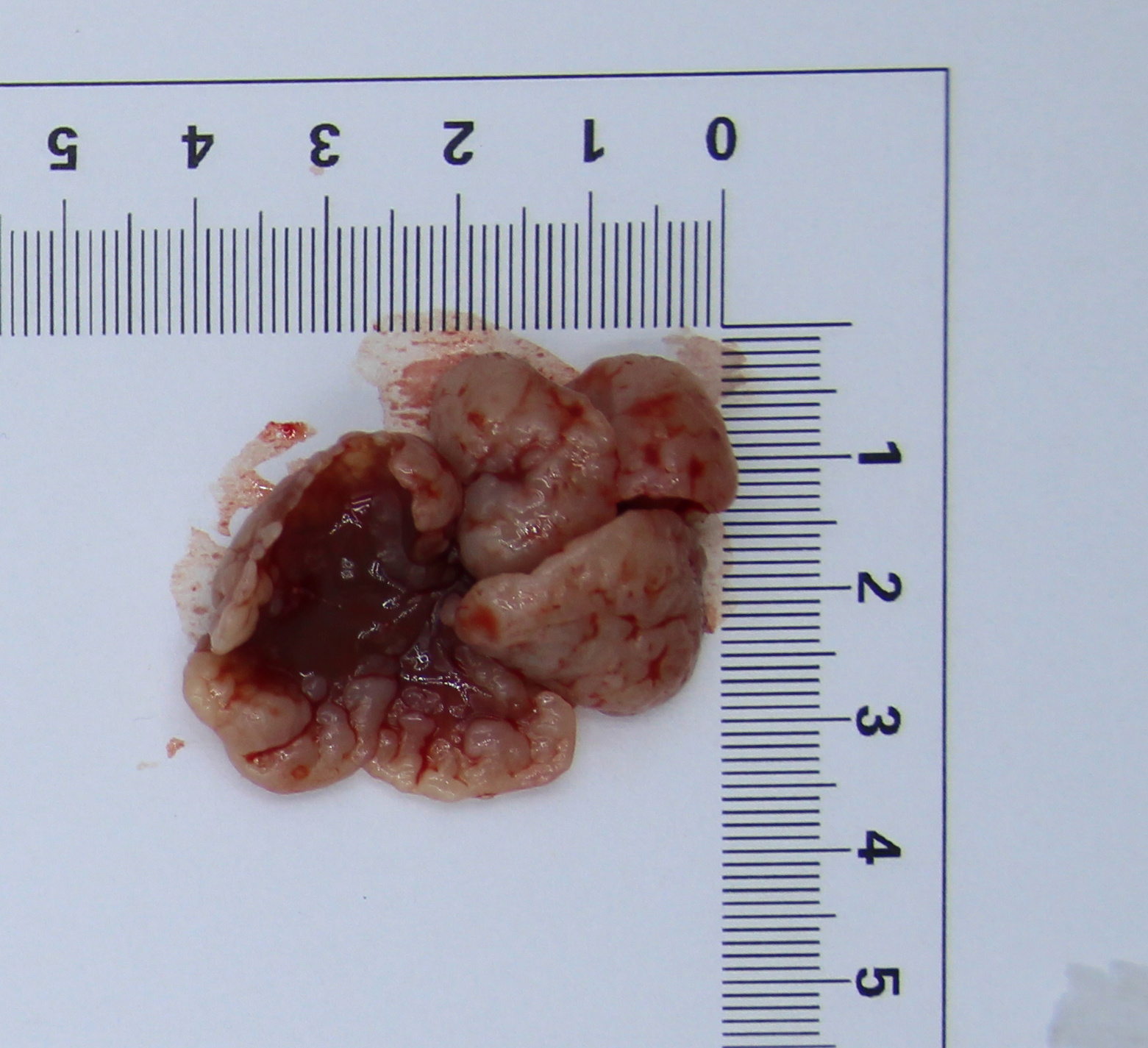

Supplement: Supplementary file 8 — Source data Fig. 3 [file 44321_2025_333_MOESM8_ESM.zip › Figure 3/3A/2. Tissue/1.1_Ctrl+Saline.JPG]

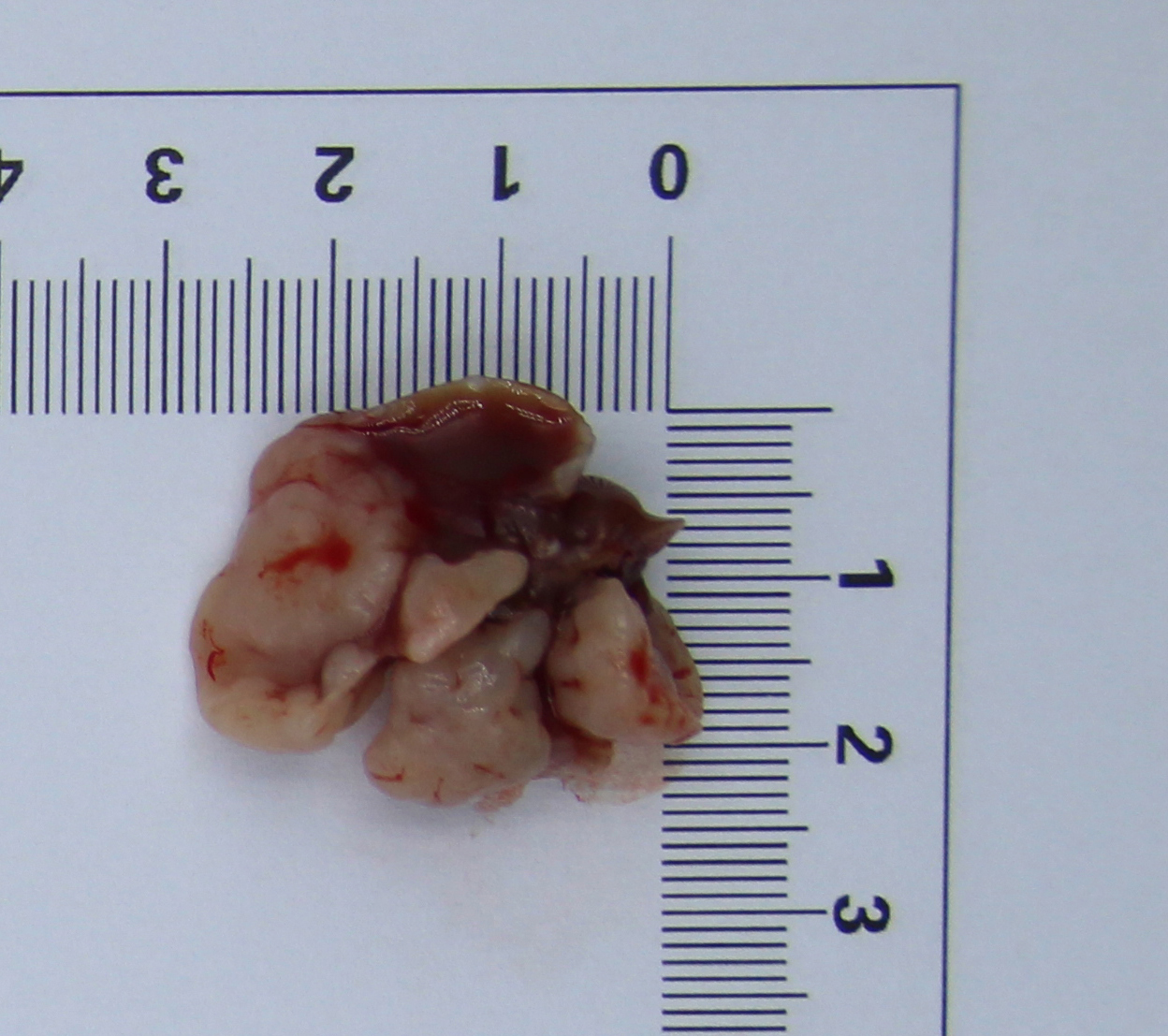

Supplement: Supplementary file 8 — Source data Fig. 3 [file 44321_2025_333_MOESM8_ESM.zip › Figure 3/3A/2. Tissue/1.2_Ctrl+Saline.JPG]

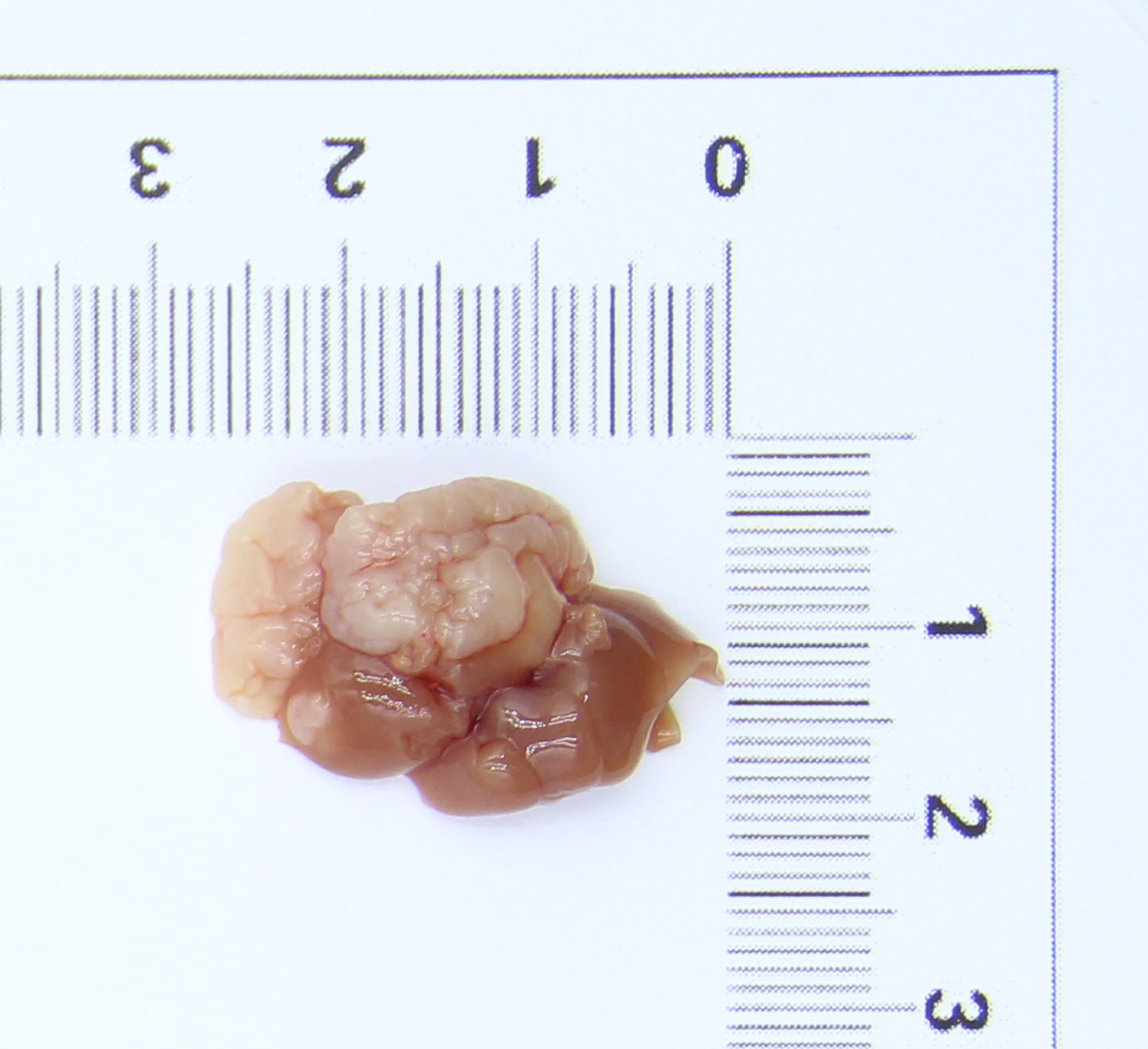

Supplement: Supplementary file 8 — Source data Fig. 3 [file 44321_2025_333_MOESM8_ESM.zip › Figure 3/3A/2. Tissue/2.1_OE+Saline.JPG]

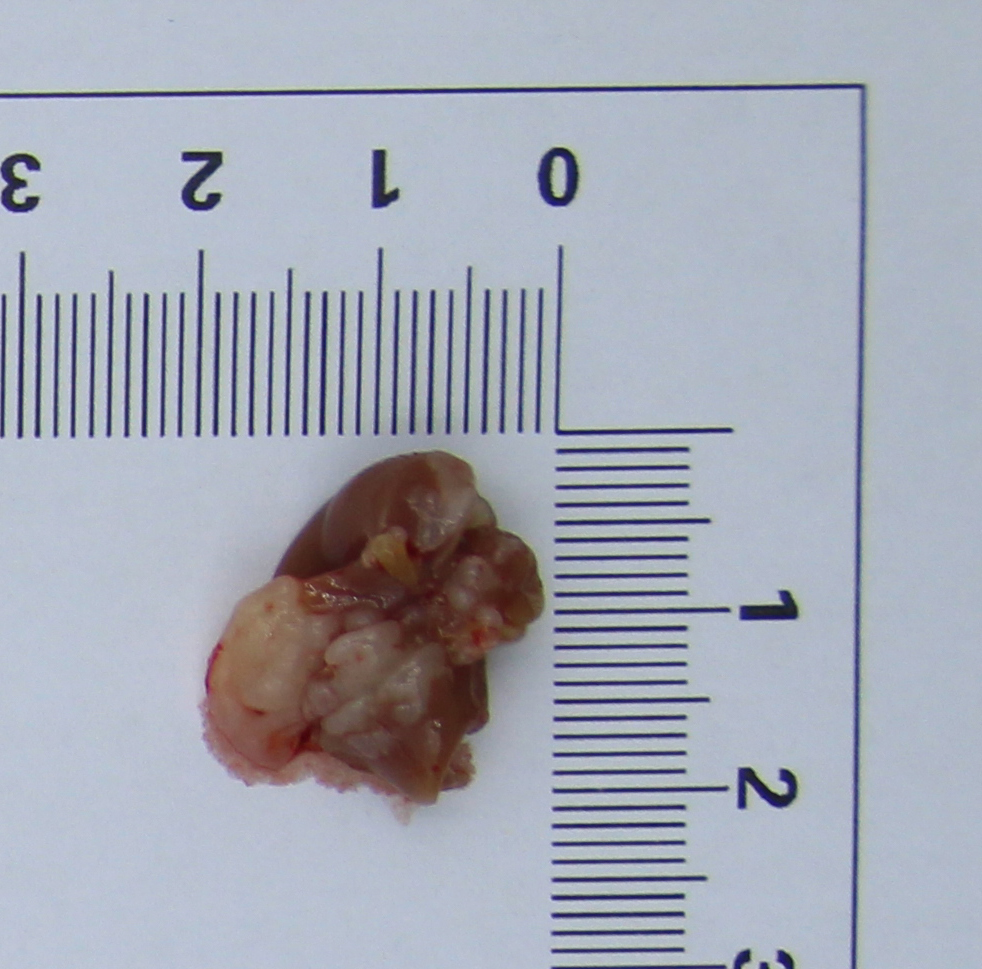

Supplement: Supplementary file 8 — Source data Fig. 3 [file 44321_2025_333_MOESM8_ESM.zip › Figure 3/3A/2. Tissue/2.2_OE+Saline.JPG]

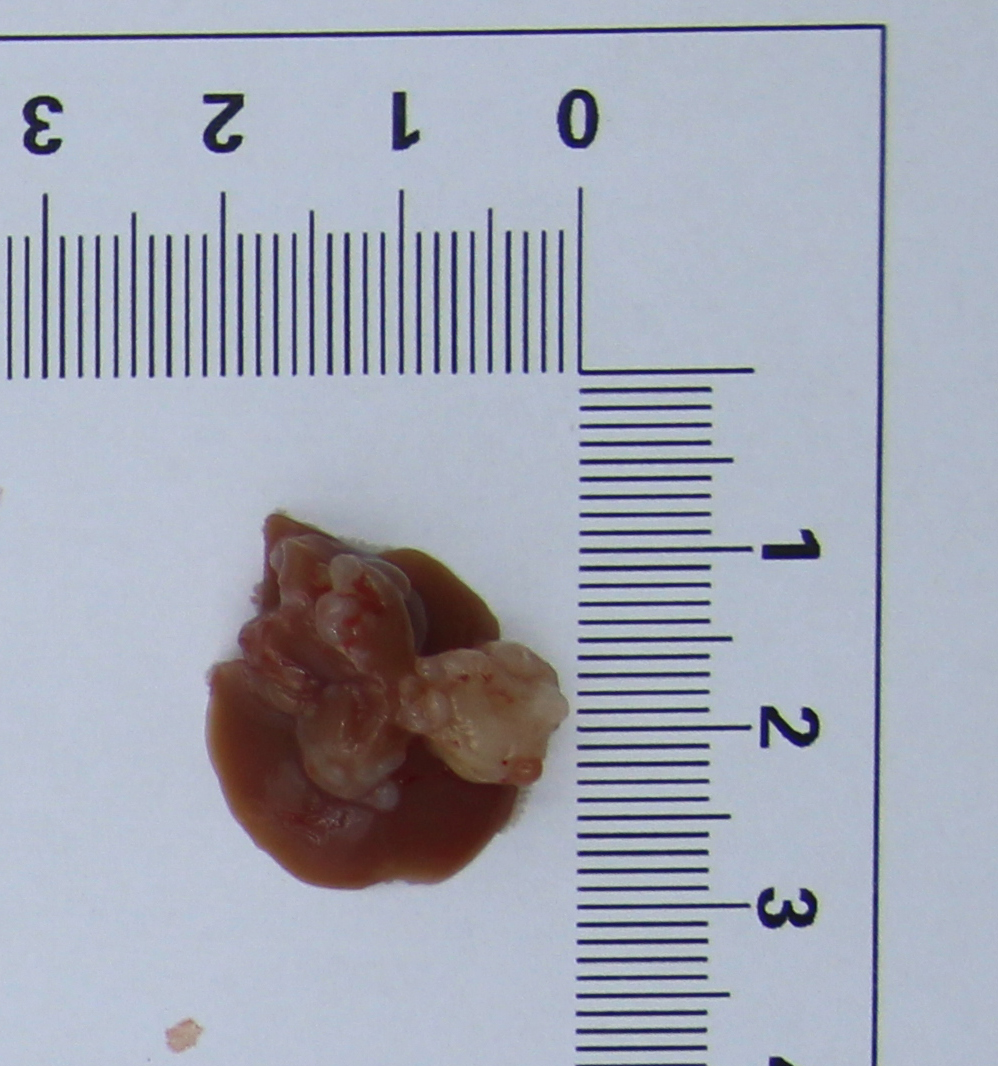

Supplement: Supplementary file 8 — Source data Fig. 3 [file 44321_2025_333_MOESM8_ESM.zip › Figure 3/3A/2. Tissue/3.1_Ctrl+Cet.JPG]

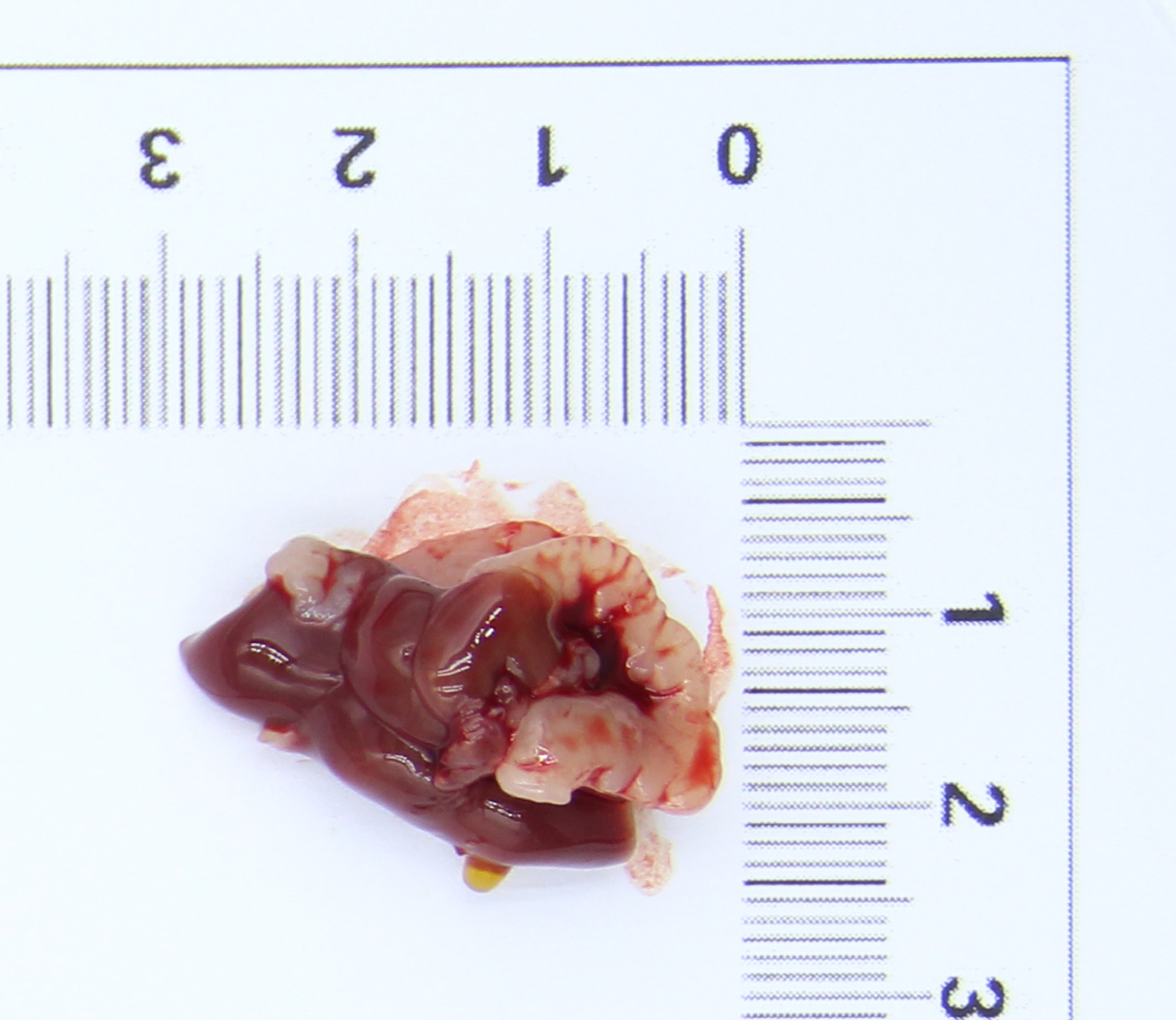

Supplement: Supplementary file 8 — Source data Fig. 3 [file 44321_2025_333_MOESM8_ESM.zip › Figure 3/3A/2. Tissue/3.2_Ctrl+Cet.JPG]

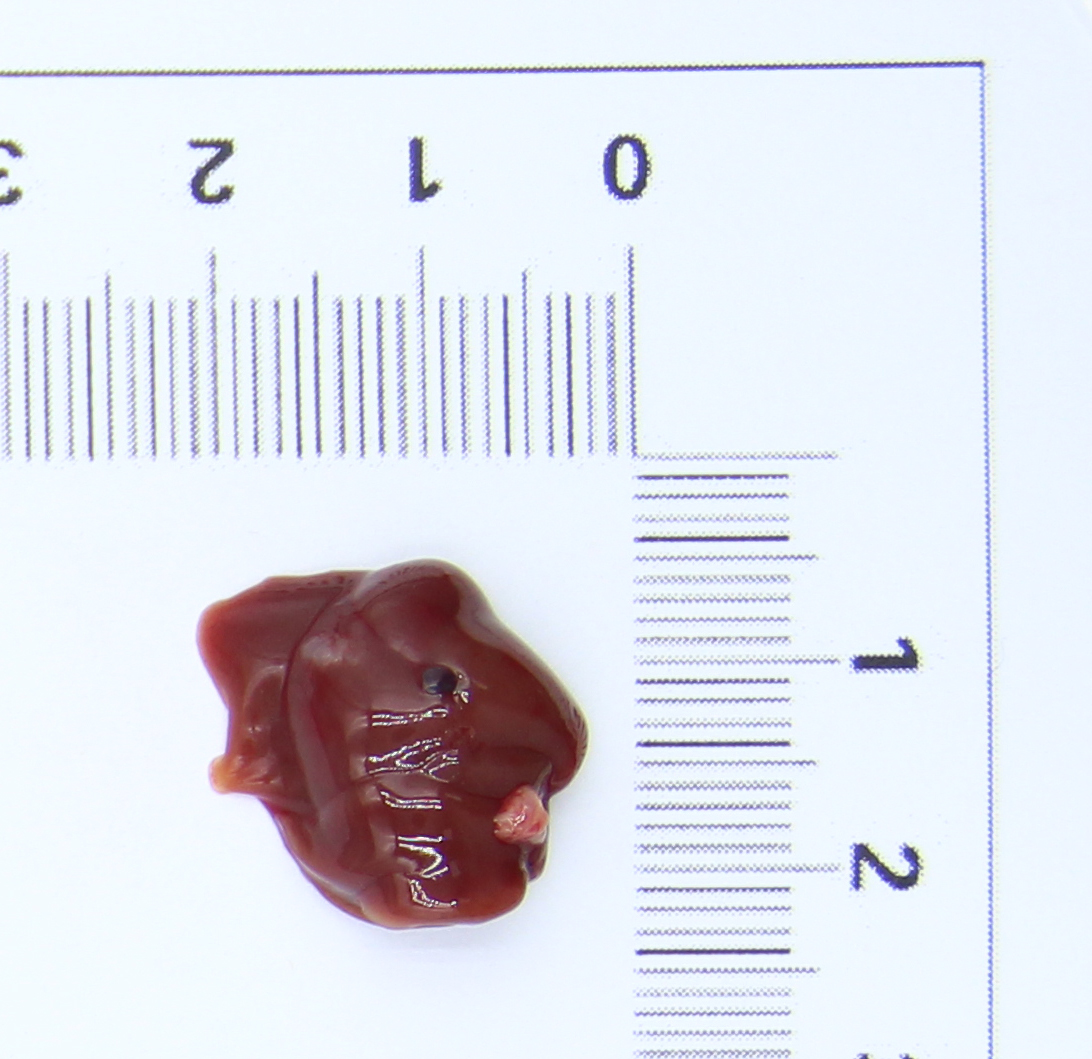

Supplement: Supplementary file 8 — Source data Fig. 3 [file 44321_2025_333_MOESM8_ESM.zip › Figure 3/3A/2. Tissue/4.1_OE+Cet.JPG]

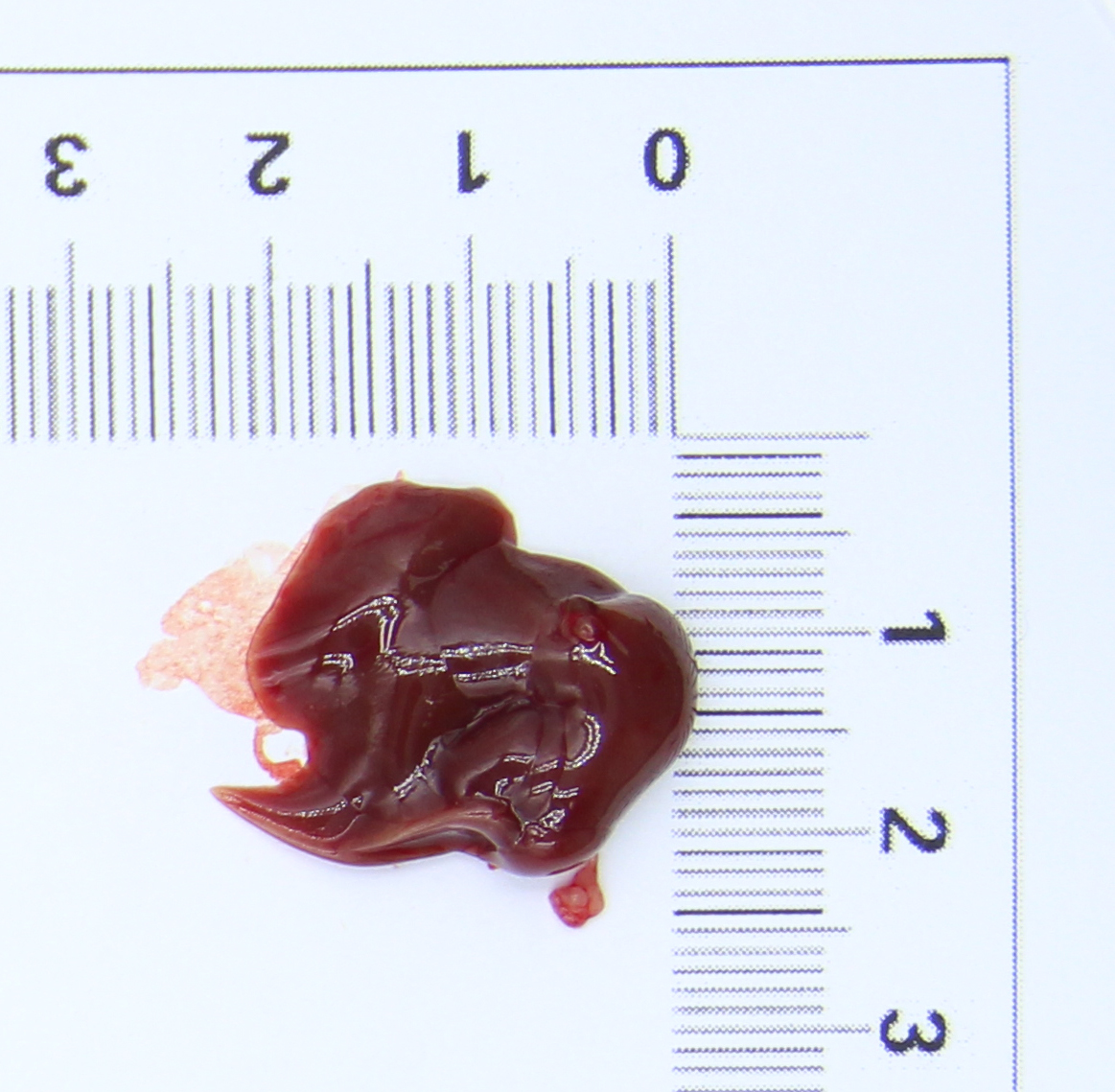

Supplement: Supplementary file 8 — Source data Fig. 3 [file 44321_2025_333_MOESM8_ESM.zip › Figure 3/3A/2. Tissue/4.2_OE+Cet.JPG]

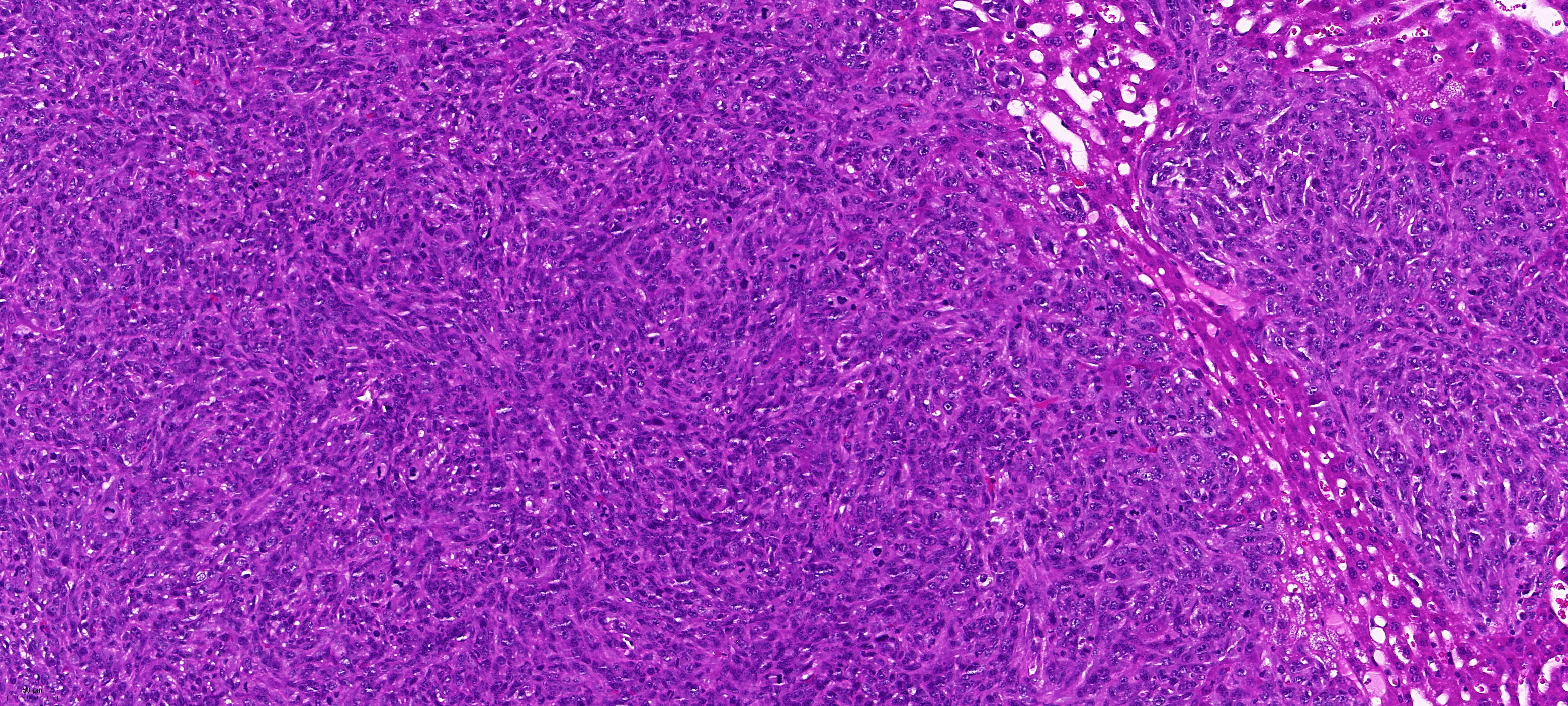

Supplement: Supplementary file 8 — Source data Fig. 3 [file 44321_2025_333_MOESM8_ESM.zip › Figure 3/3B/20x/1_Ctrl+Saline.jpg]

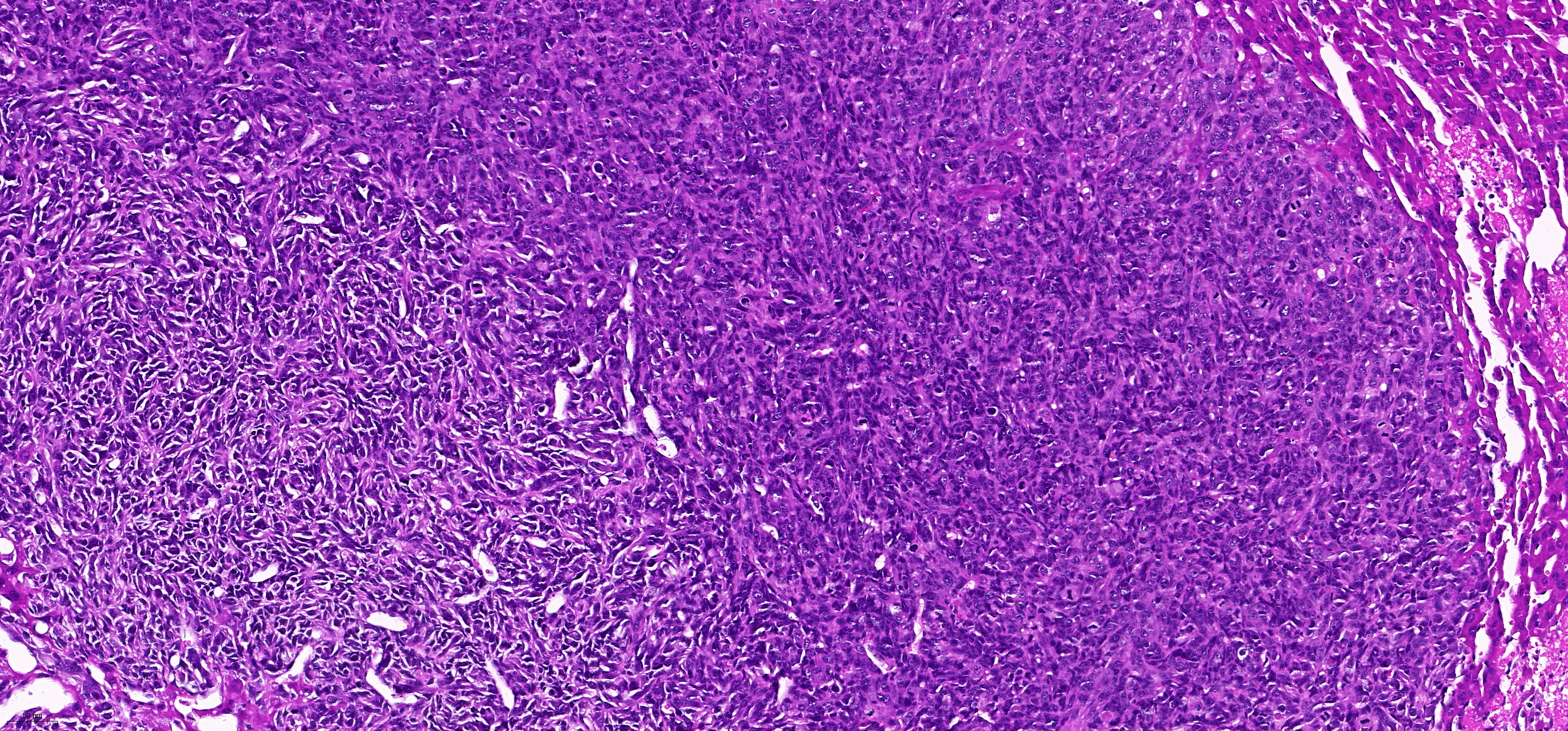

Supplement: Supplementary file 8 — Source data Fig. 3 [file 44321_2025_333_MOESM8_ESM.zip › Figure 3/3B/20x/2_OE+Saline.jpg]

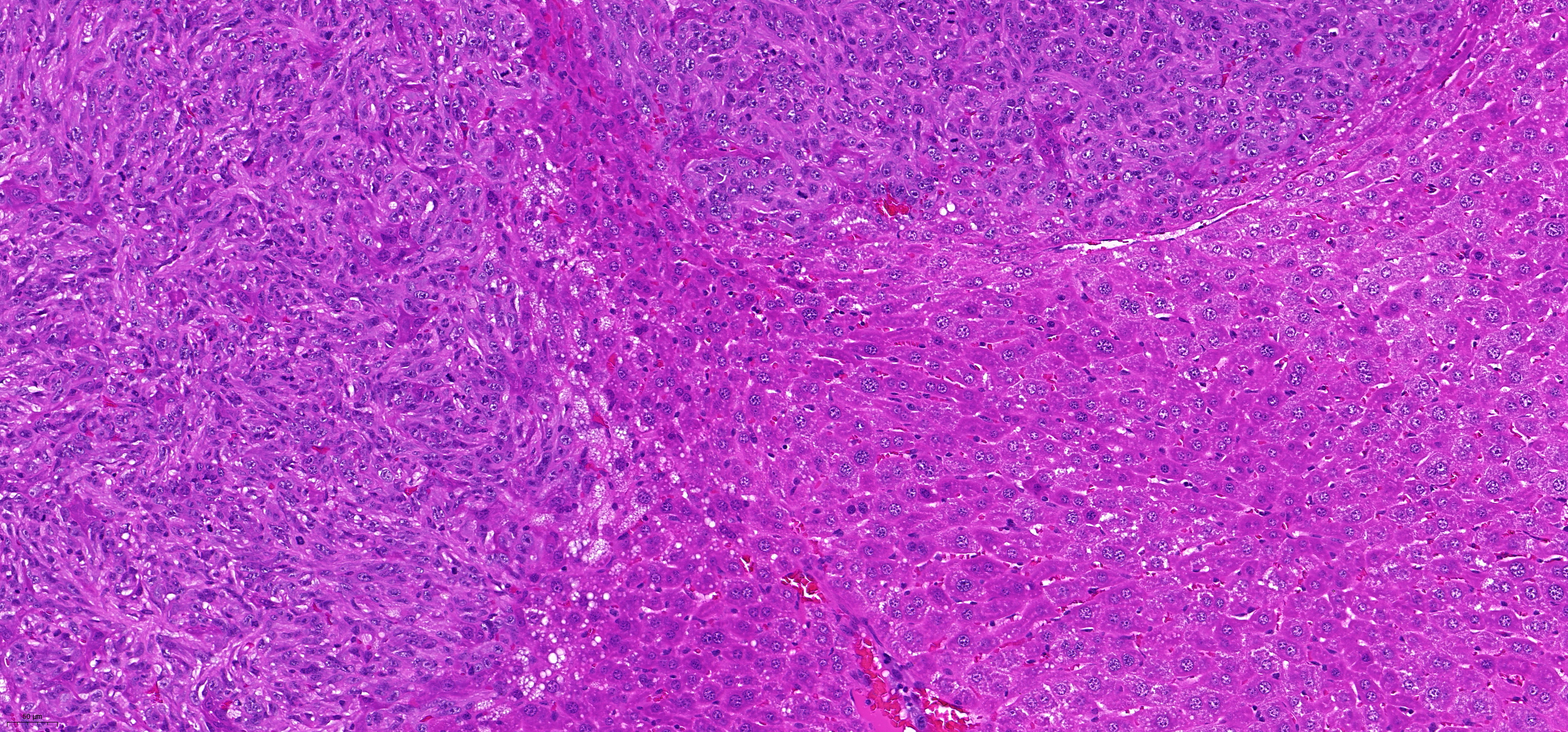

Supplement: Supplementary file 8 — Source data Fig. 3 [file 44321_2025_333_MOESM8_ESM.zip › Figure 3/3B/20x/3_Ctrl+Cet.jpg]

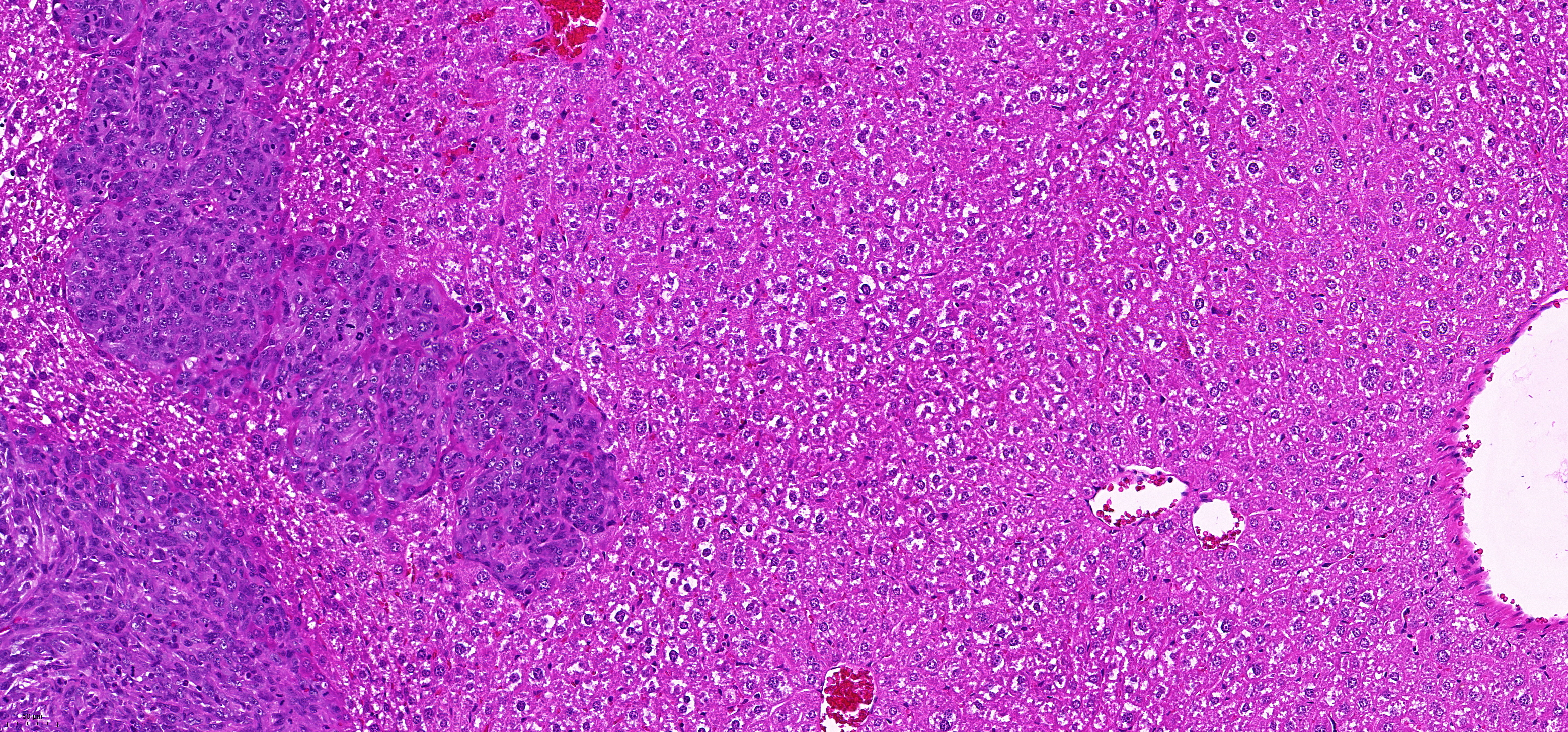

Supplement: Supplementary file 8 — Source data Fig. 3 [file 44321_2025_333_MOESM8_ESM.zip › Figure 3/3B/20x/4_OE+Cet.jpg]

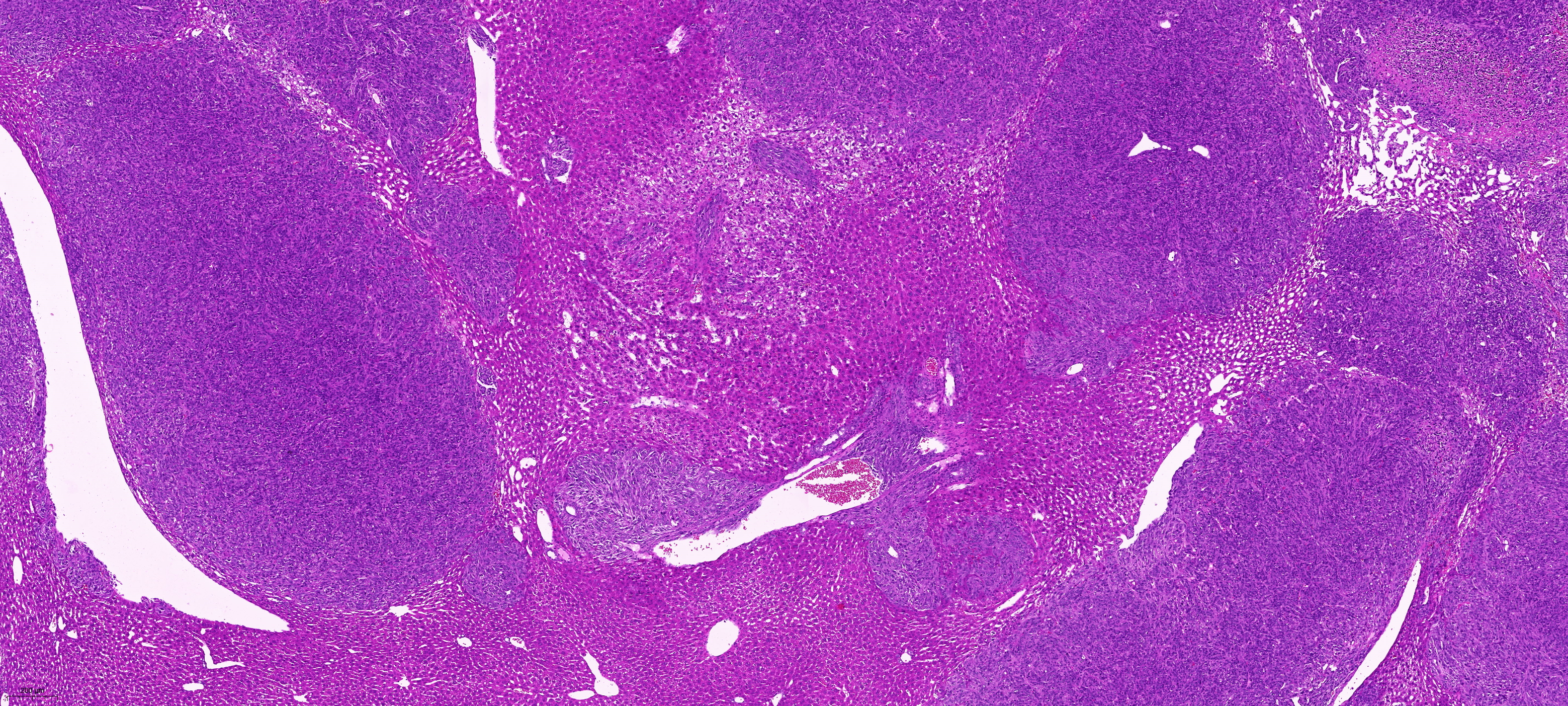

Supplement: Supplementary file 8 — Source data Fig. 3 [file 44321_2025_333_MOESM8_ESM.zip › Figure 3/3B/5x/1_Ctrl+Saline.jpg]

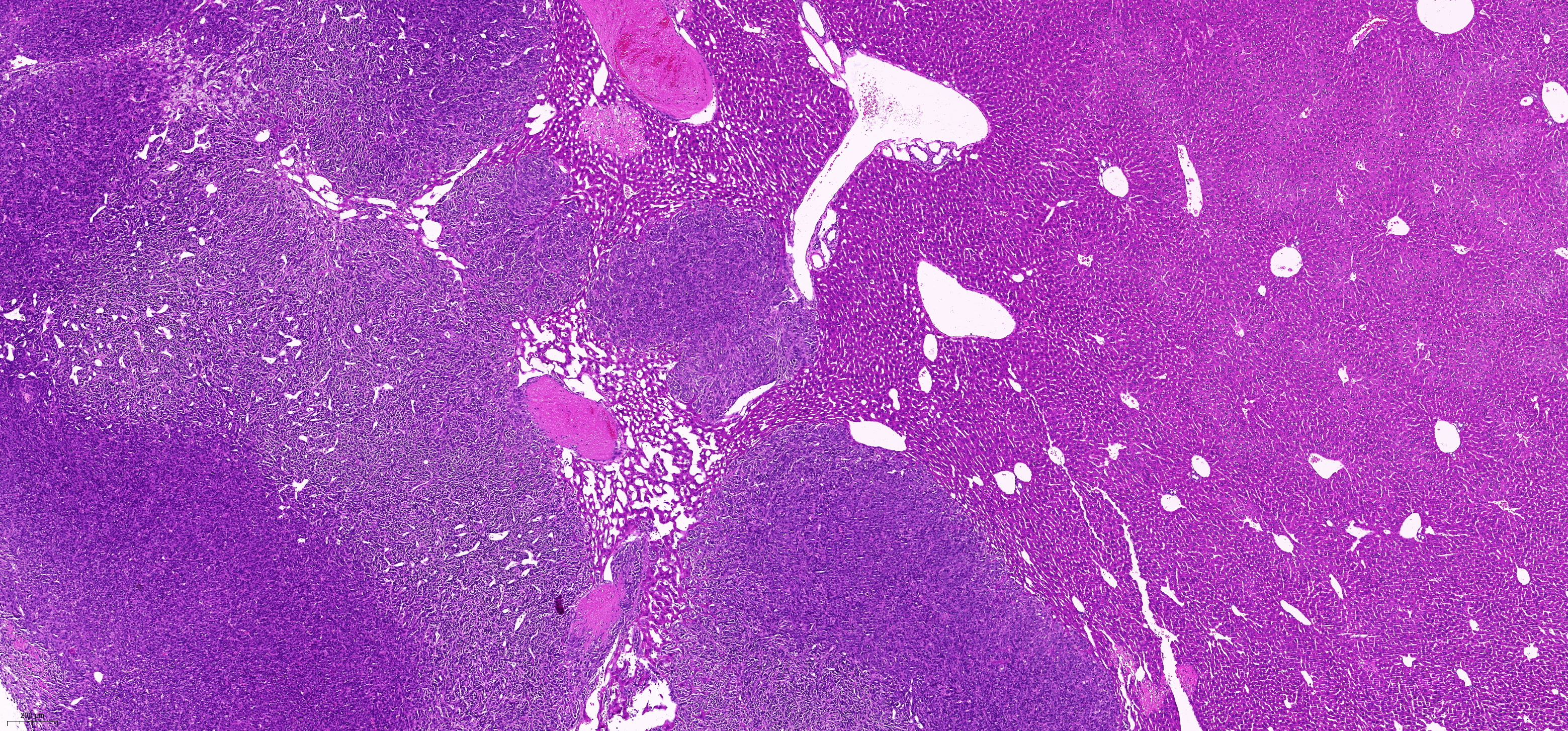

Supplement: Supplementary file 8 — Source data Fig. 3 [file 44321_2025_333_MOESM8_ESM.zip › Figure 3/3B/5x/2_OE+Saline.jpg]

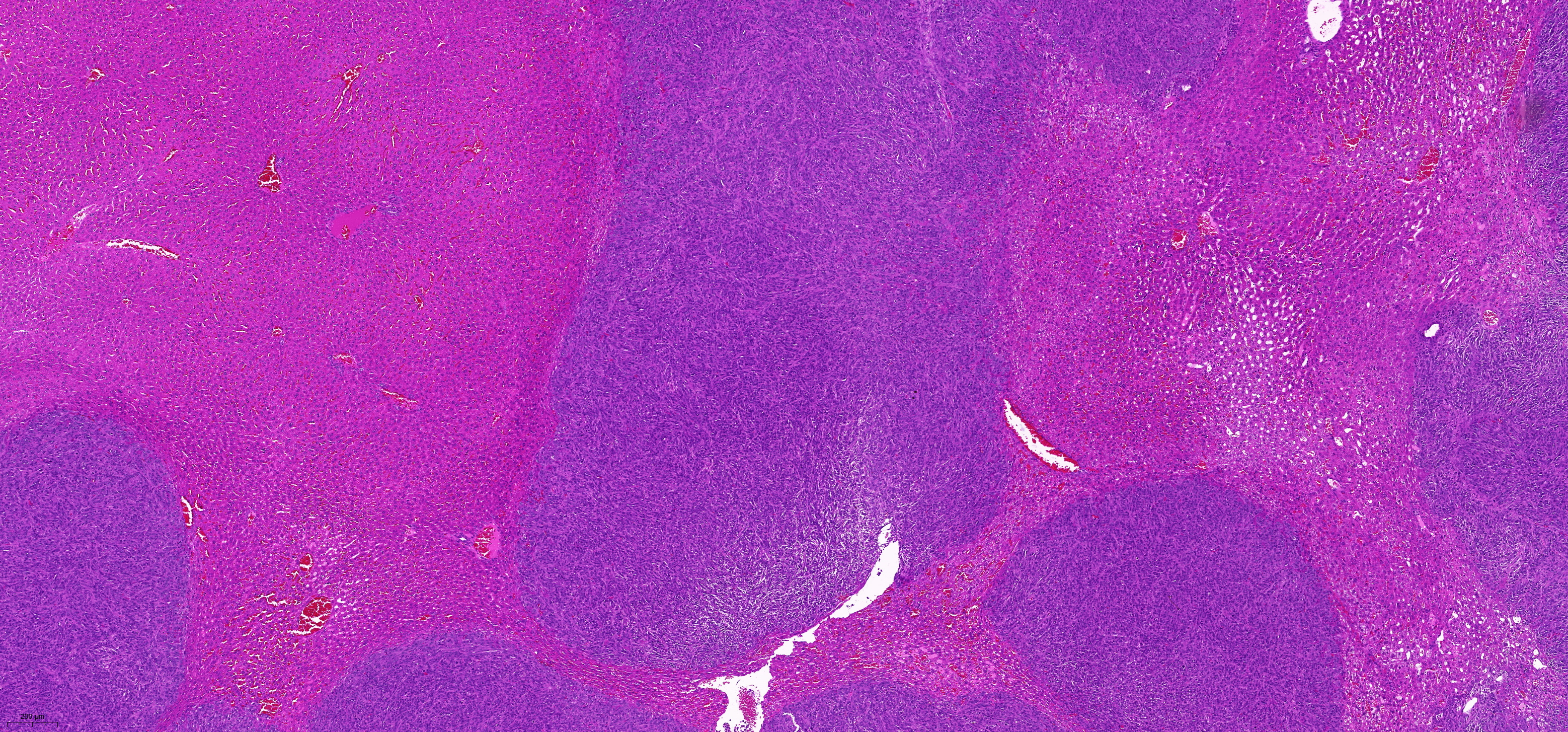

Supplement: Supplementary file 8 — Source data Fig. 3 [file 44321_2025_333_MOESM8_ESM.zip › Figure 3/3B/5x/3_Ctrl+Cet.jpg]

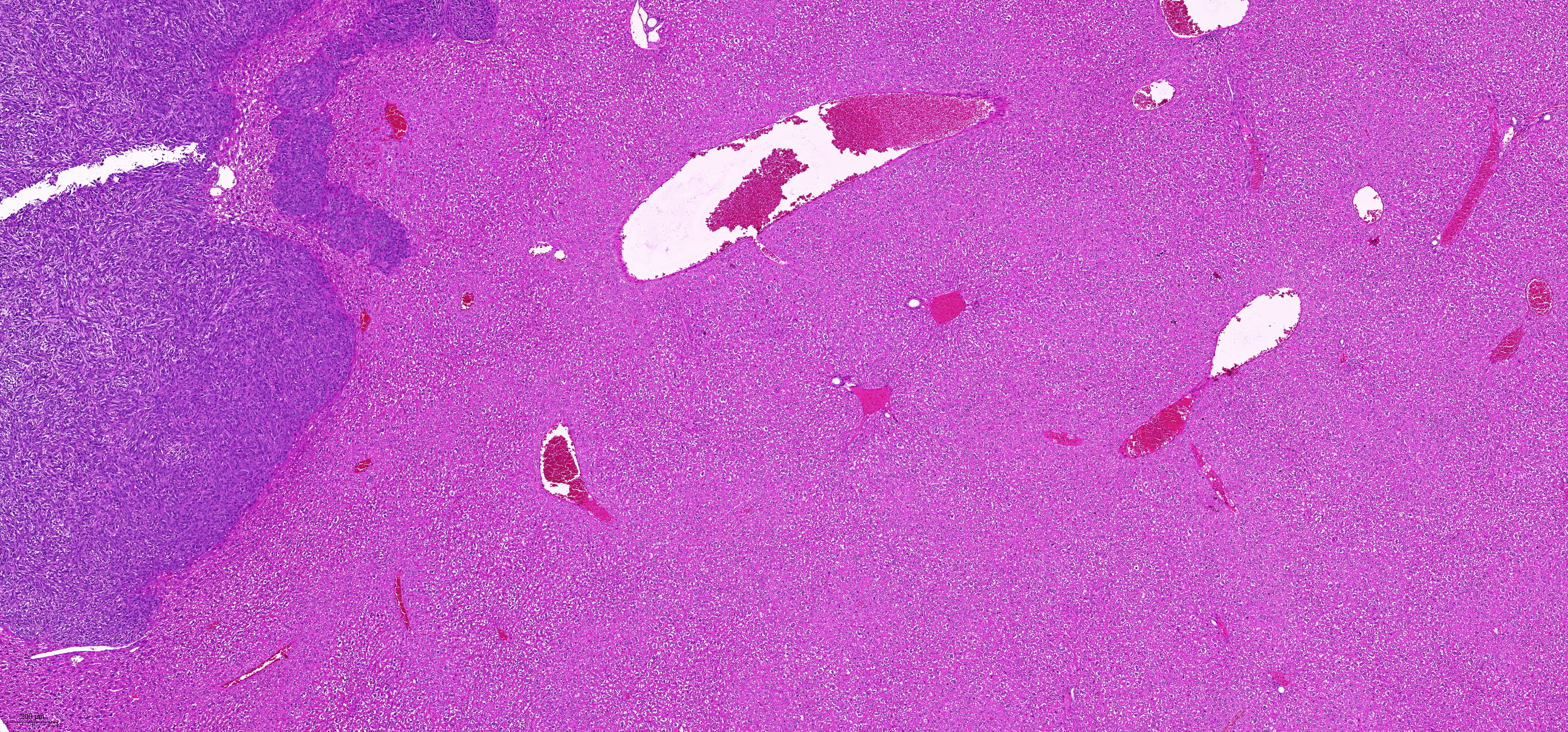

Supplement: Supplementary file 8 — Source data Fig. 3 [file 44321_2025_333_MOESM8_ESM.zip › Figure 3/3B/5x/4_OE+Cet.jpg]

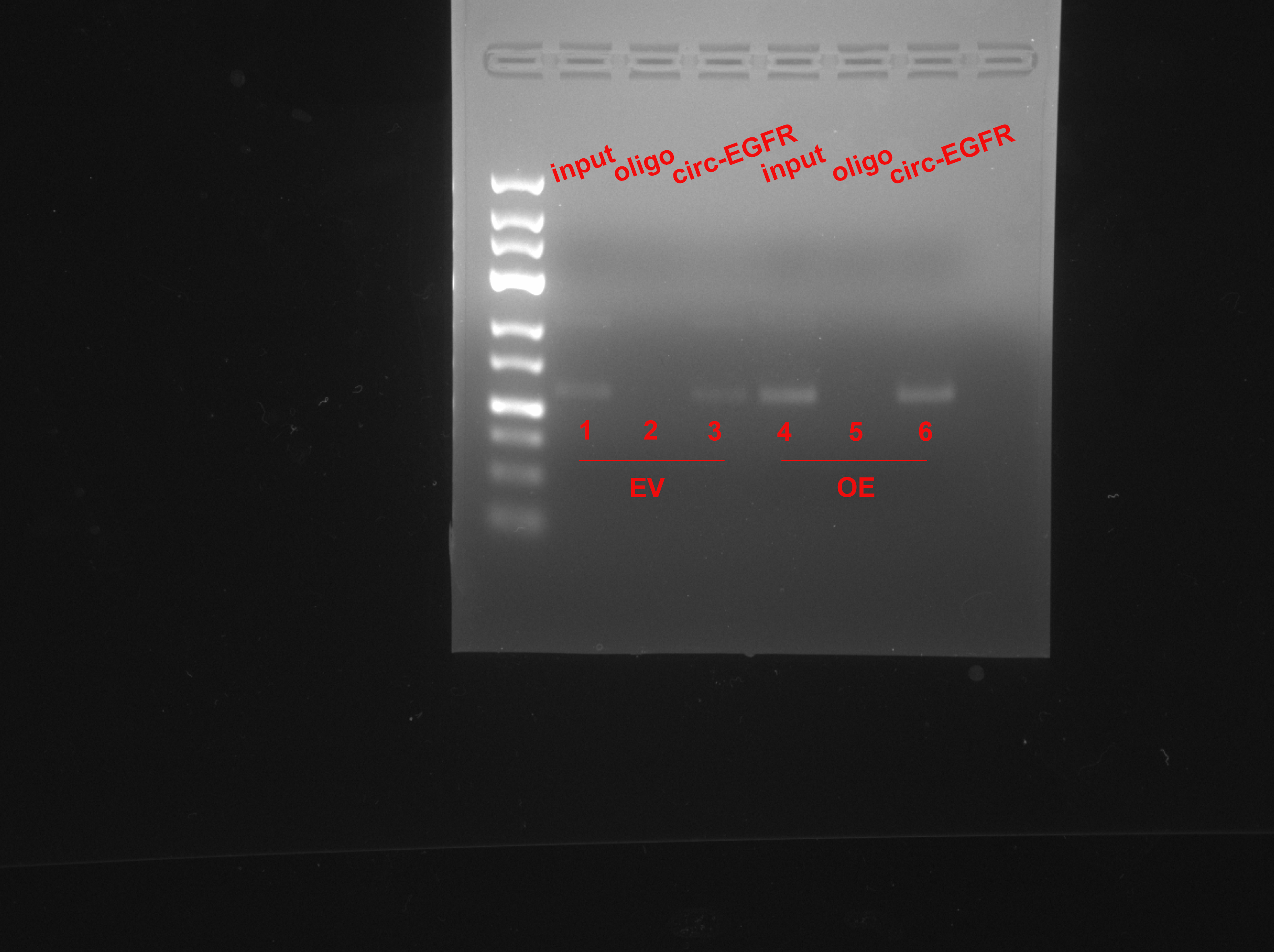

Supplement: Supplementary file 9 — Source data Fig. 4 [file 44321_2025_333_MOESM9_ESM.zip › Figure 4/4D/CaCO2/circ-EGFR/circEGFR_CaCO2.tif]

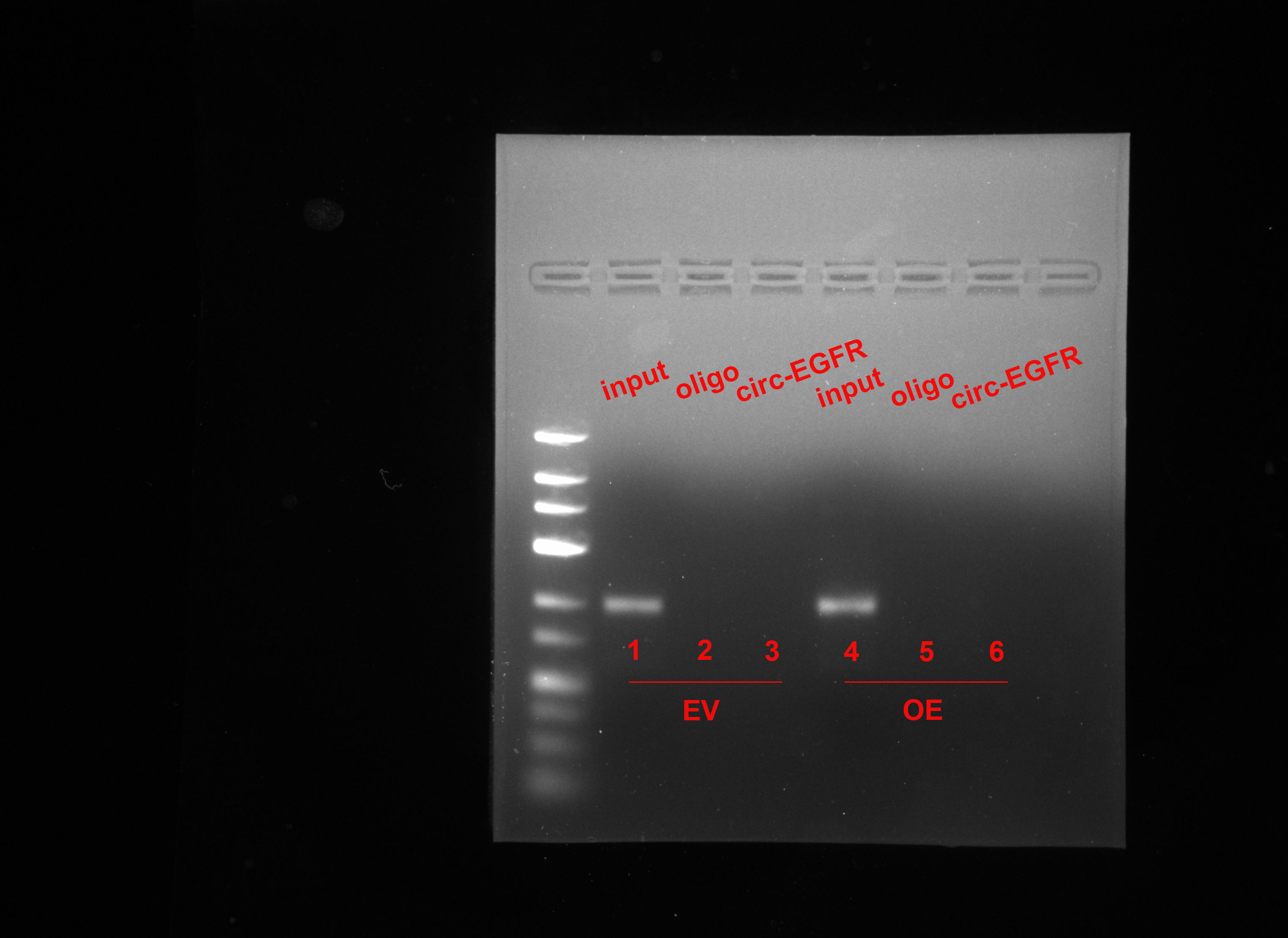

Supplement: Supplementary file 9 — Source data Fig. 4 [file 44321_2025_333_MOESM9_ESM.zip › Figure 4/4D/CaCO2/GAPDH/GAPDH_CaCO2.tif]

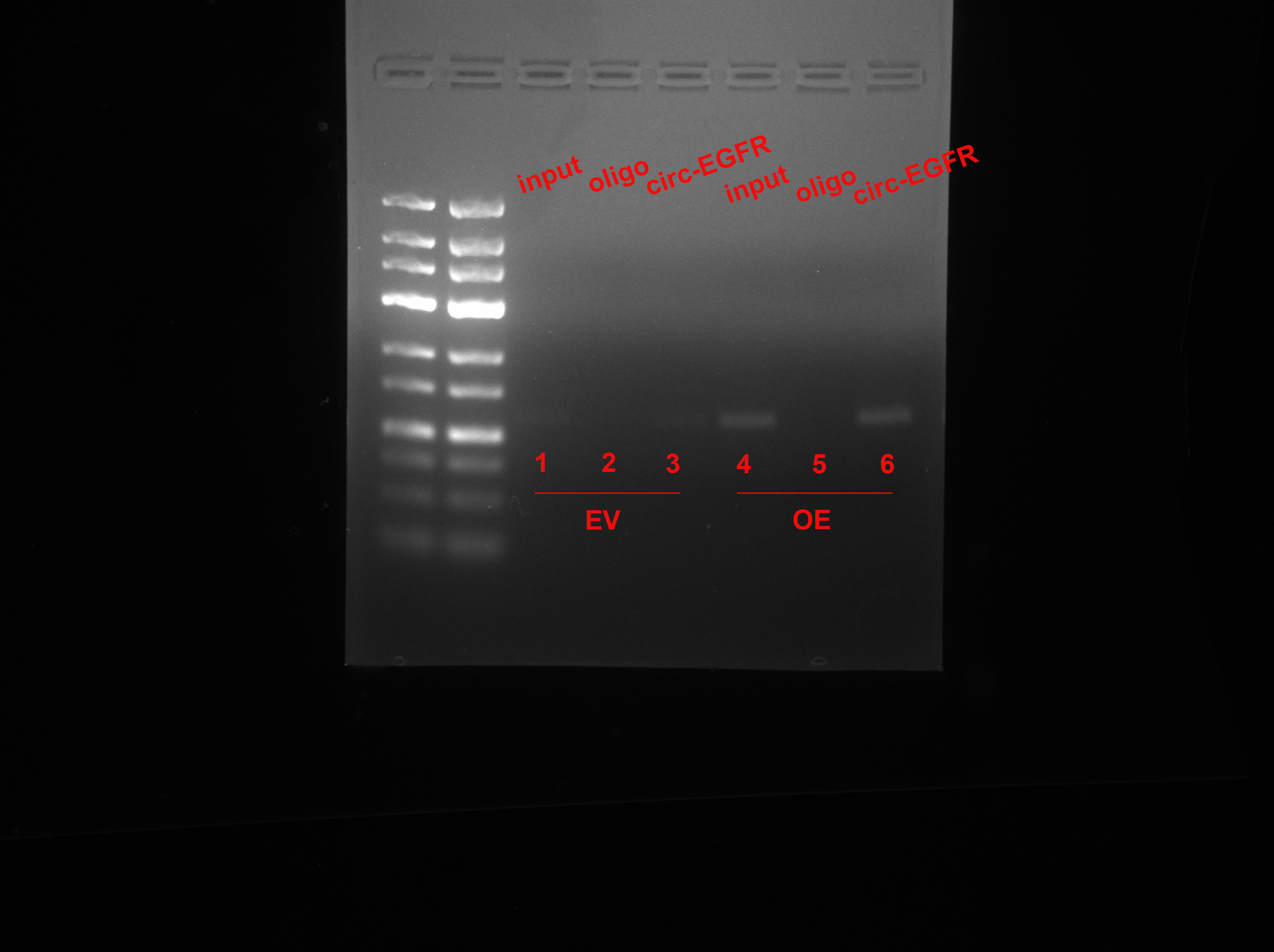

Supplement: Supplementary file 9 — Source data Fig. 4 [file 44321_2025_333_MOESM9_ESM.zip › Figure 4/4D/SNU-C1/circ-EGFR/circEGFR_SNU-C1.tif]

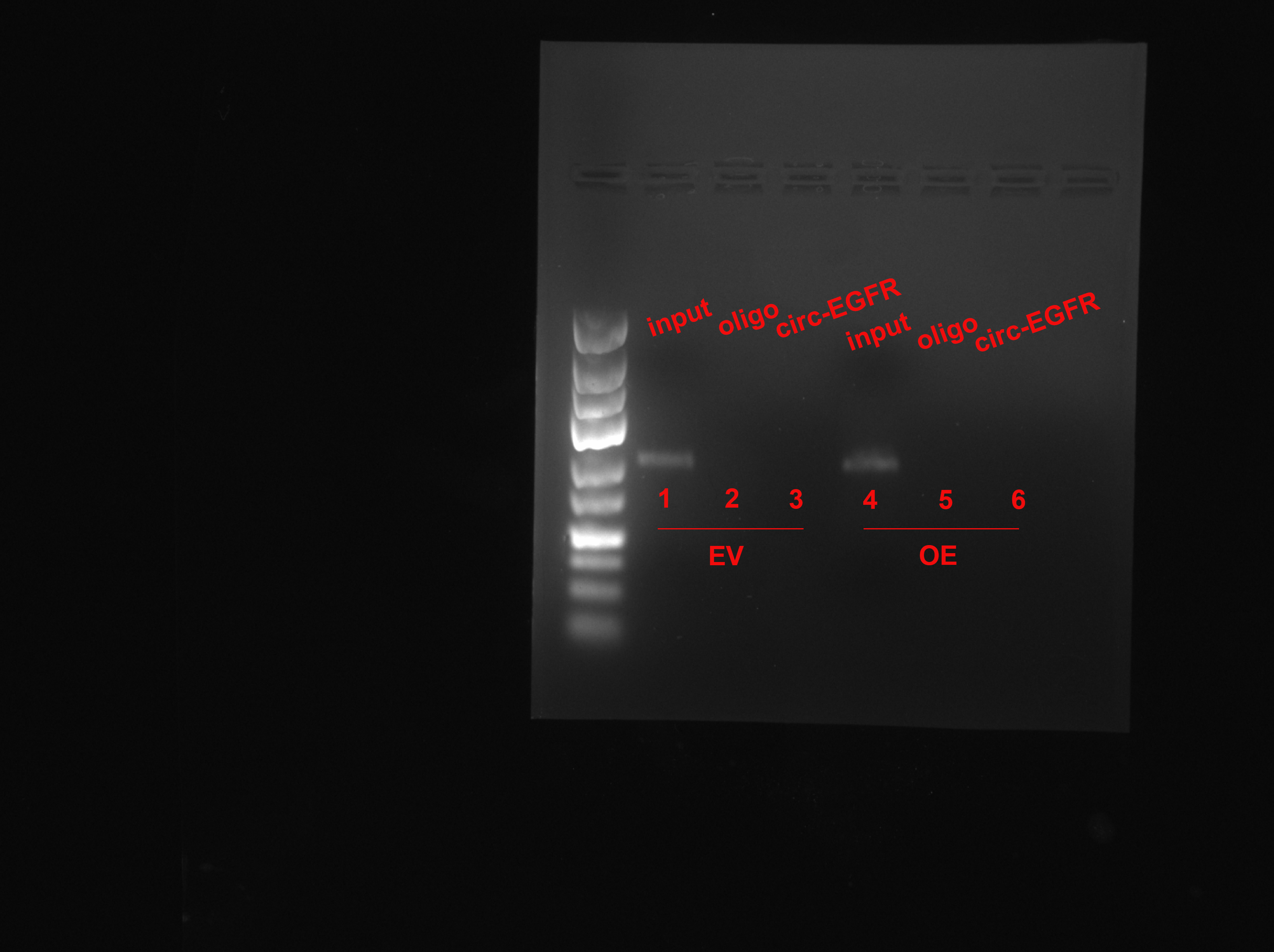

Supplement: Supplementary file 9 — Source data Fig. 4 [file 44321_2025_333_MOESM9_ESM.zip › Figure 4/4D/SNU-C1/GAPDH/GAPDH_SNU-C1.tif]

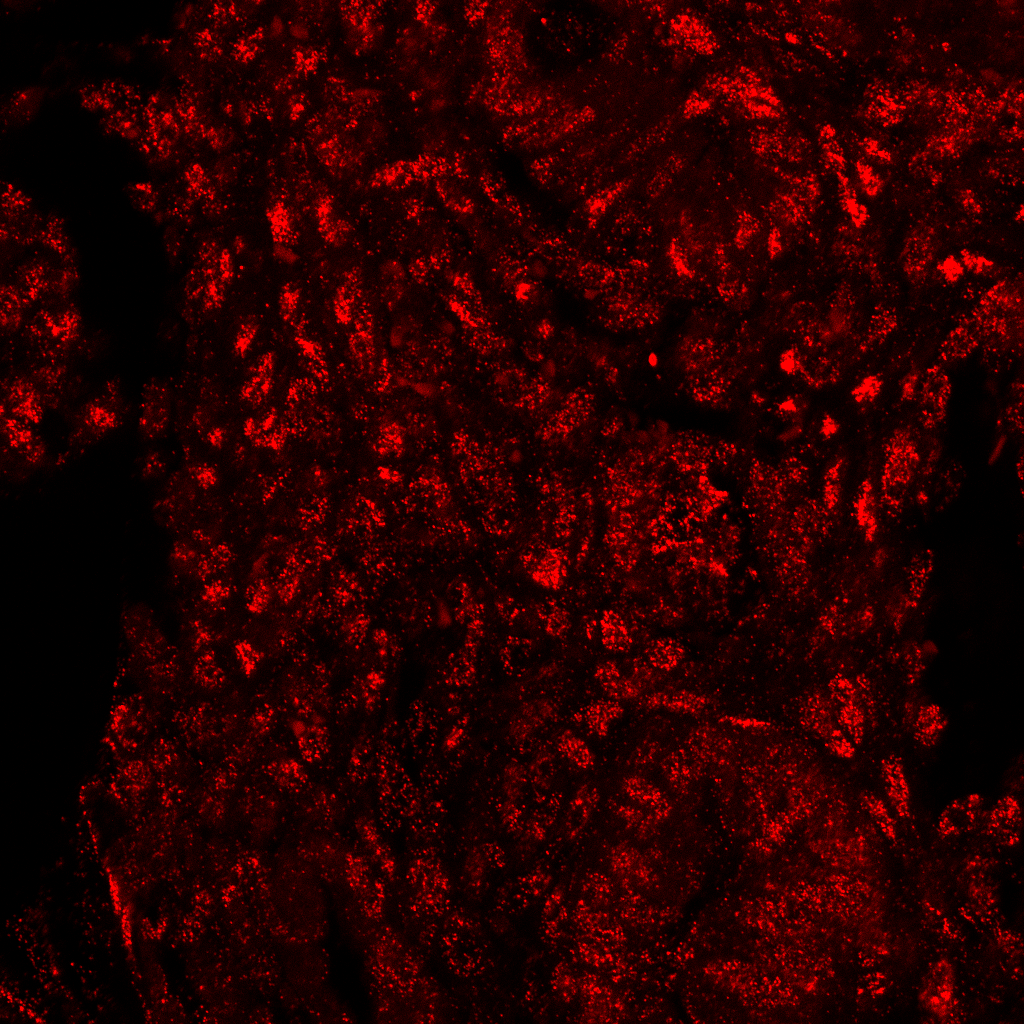

Supplement: Supplementary file 9 — Source data Fig. 4 [file 44321_2025_333_MOESM9_ESM.zip › Figure 4/4I/P01/circEGFR.tiff]

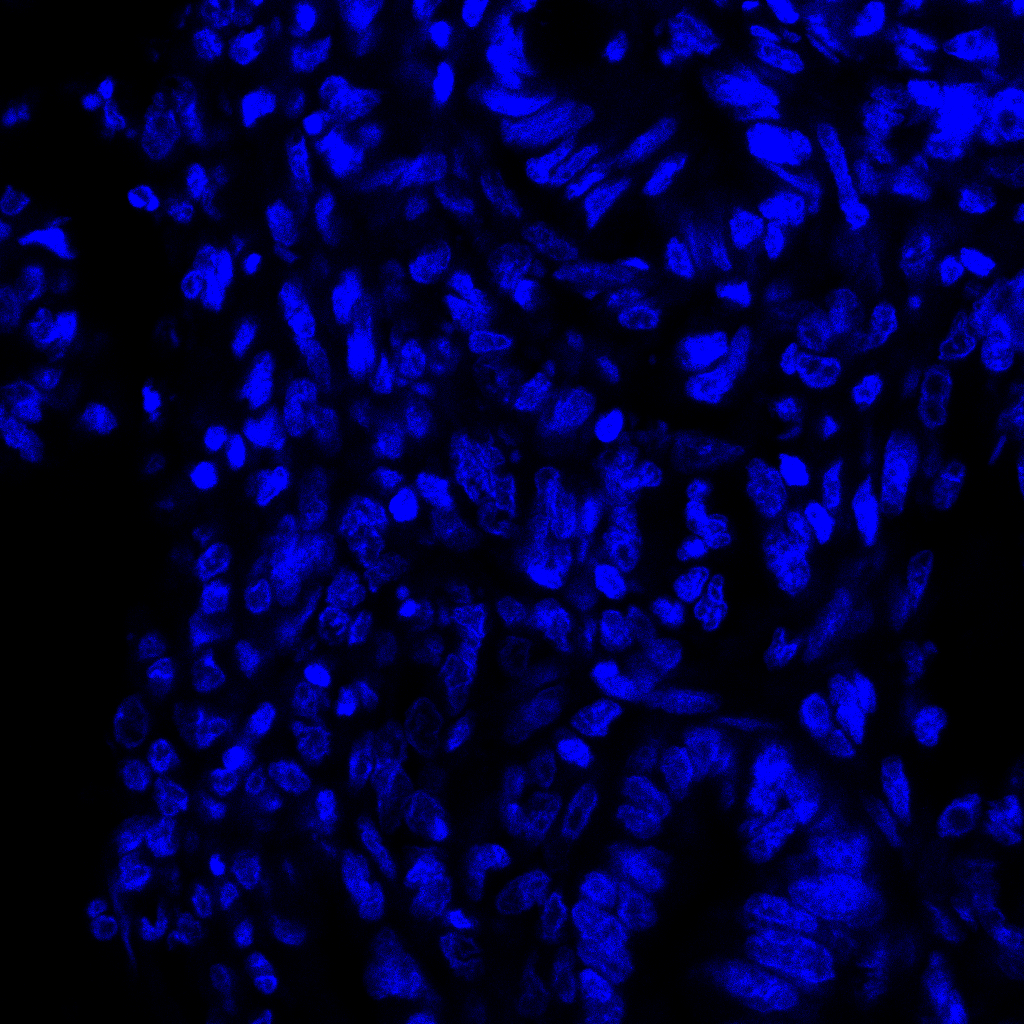

Supplement: Supplementary file 9 — Source data Fig. 4 [file 44321_2025_333_MOESM9_ESM.zip › Figure 4/4I/P01/DAPI.tiff]

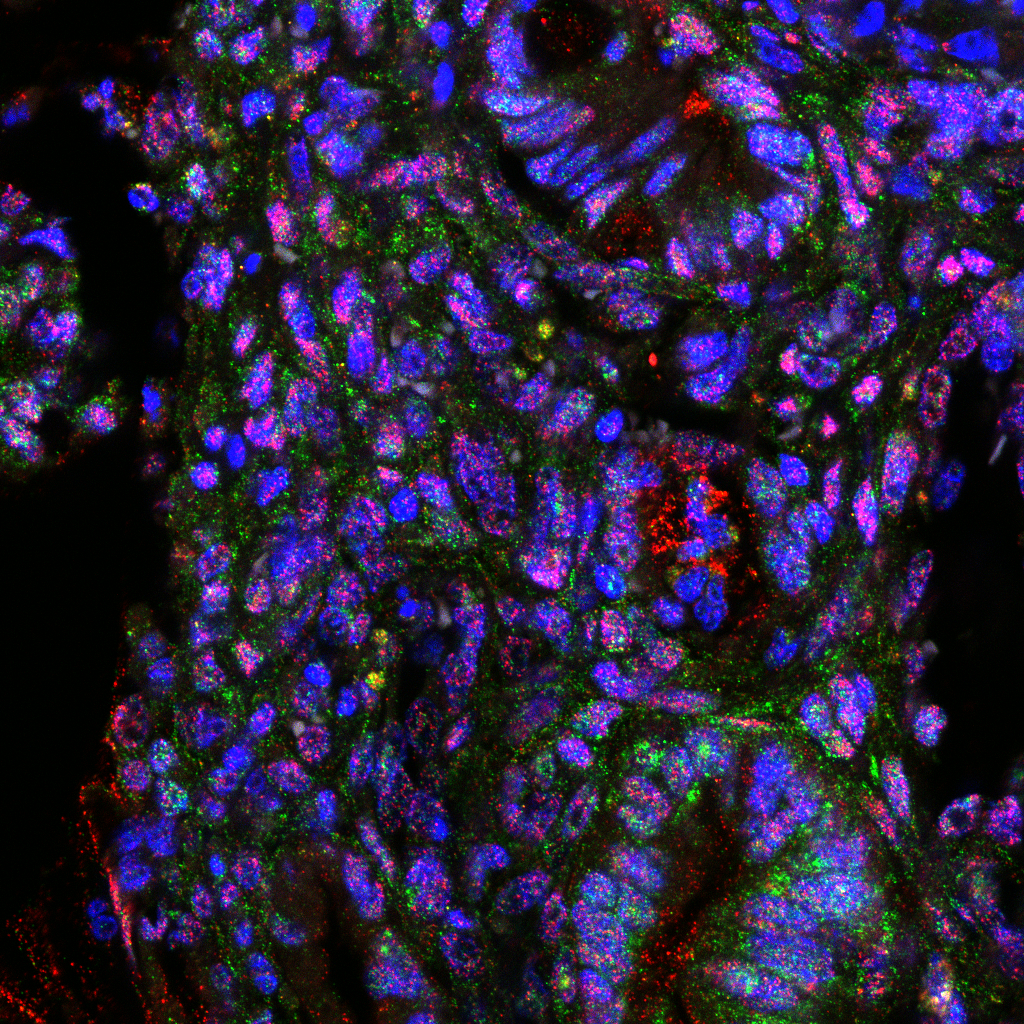

Supplement: Supplementary file 9 — Source data Fig. 4 [file 44321_2025_333_MOESM9_ESM.zip › Figure 4/4I/P01/Merge.tiff]

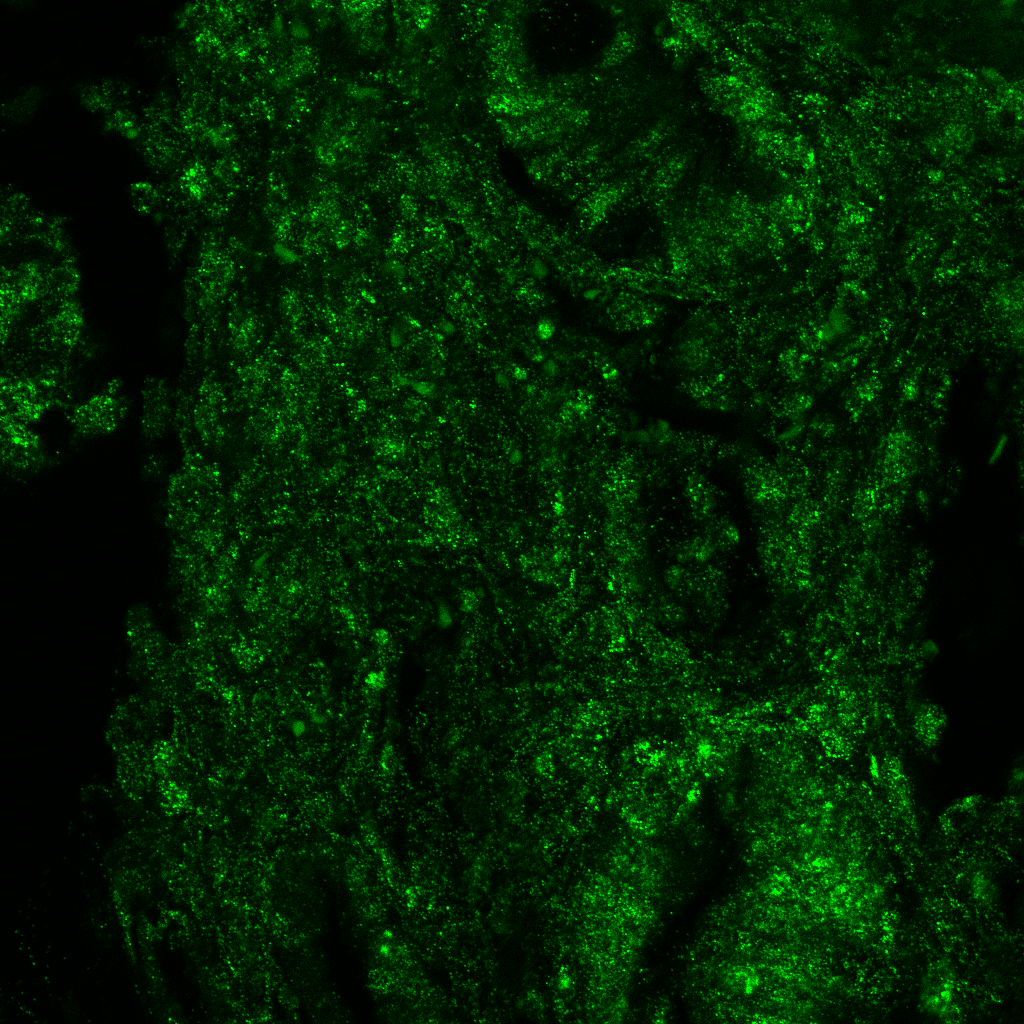

Supplement: Supplementary file 9 — Source data Fig. 4 [file 44321_2025_333_MOESM9_ESM.zip › Figure 4/4I/P01/miR-942-3p.tiff]

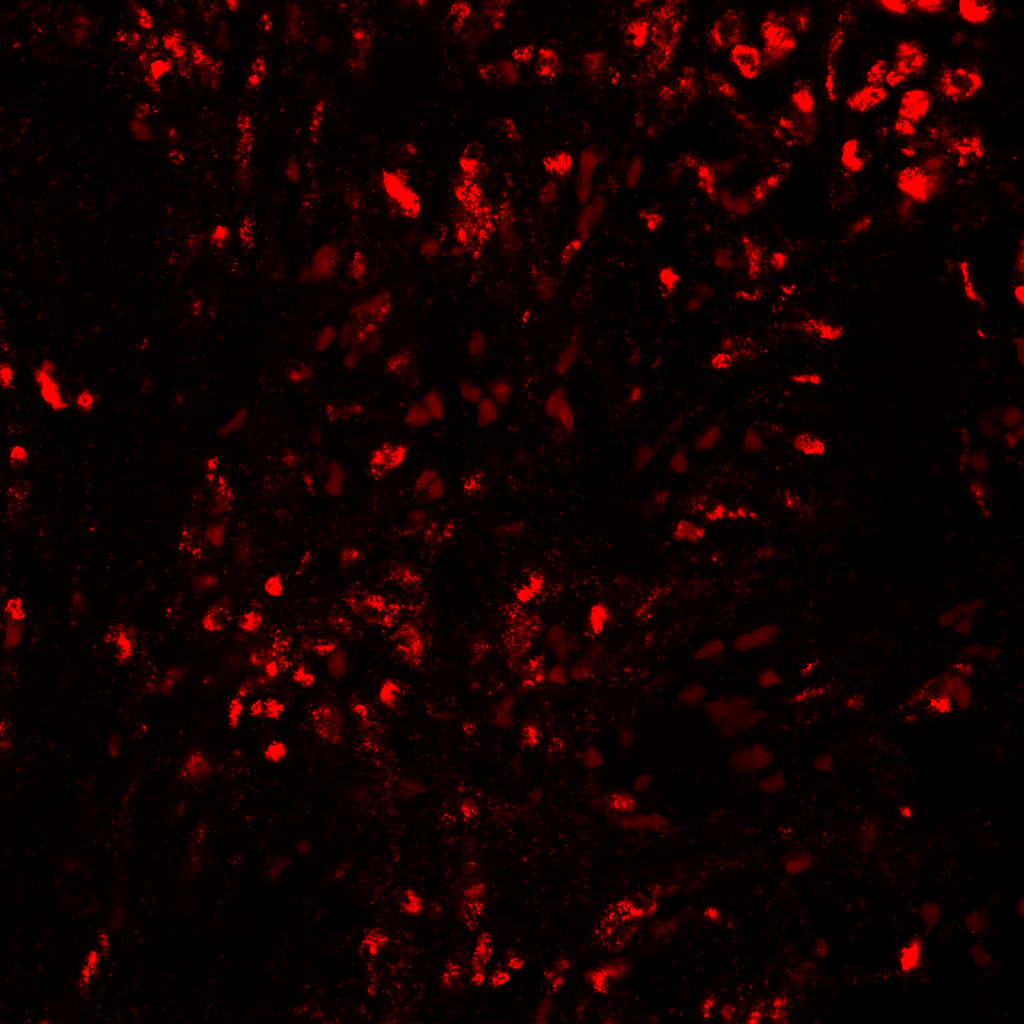

Supplement: Supplementary file 9 — Source data Fig. 4 [file 44321_2025_333_MOESM9_ESM.zip › Figure 4/4I/P02/circEGFR.tiff]

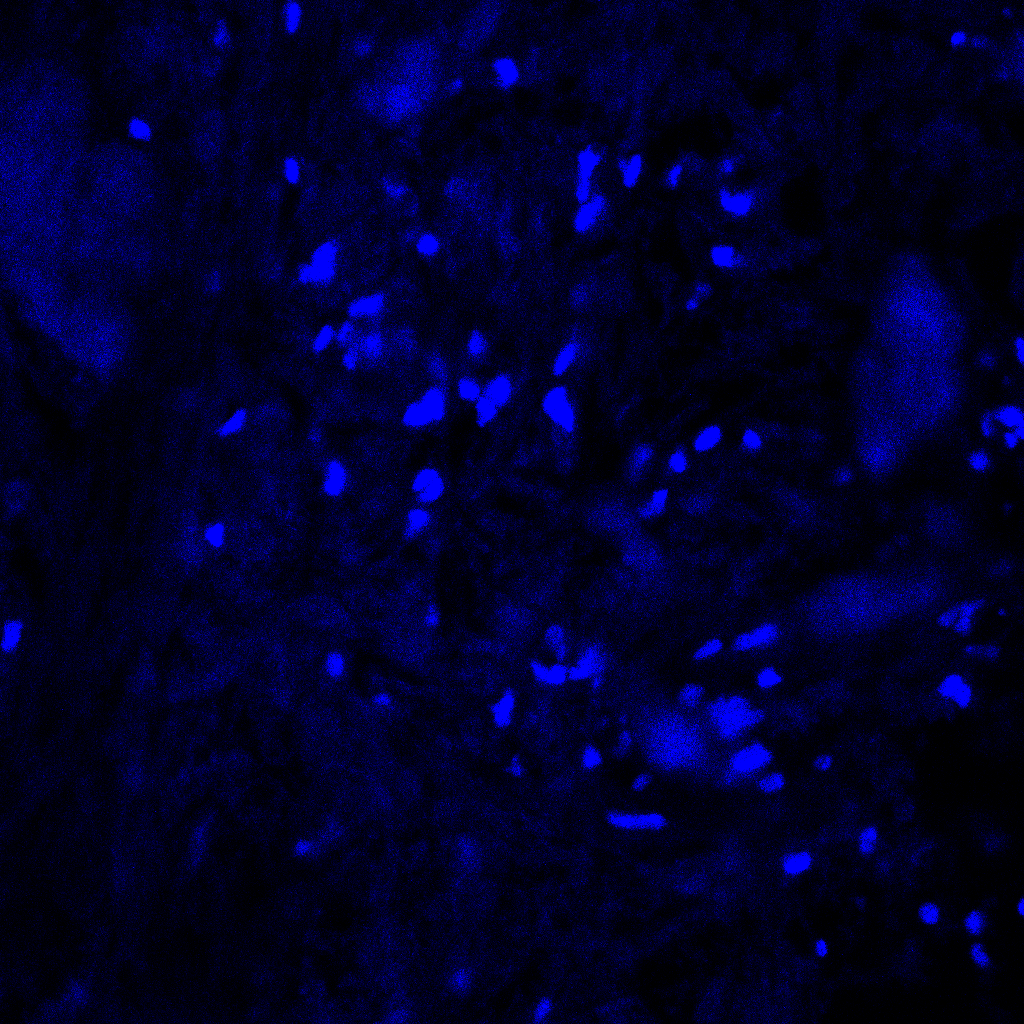

Supplement: Supplementary file 9 — Source data Fig. 4 [file 44321_2025_333_MOESM9_ESM.zip › Figure 4/4I/P02/DAPI.tiff]

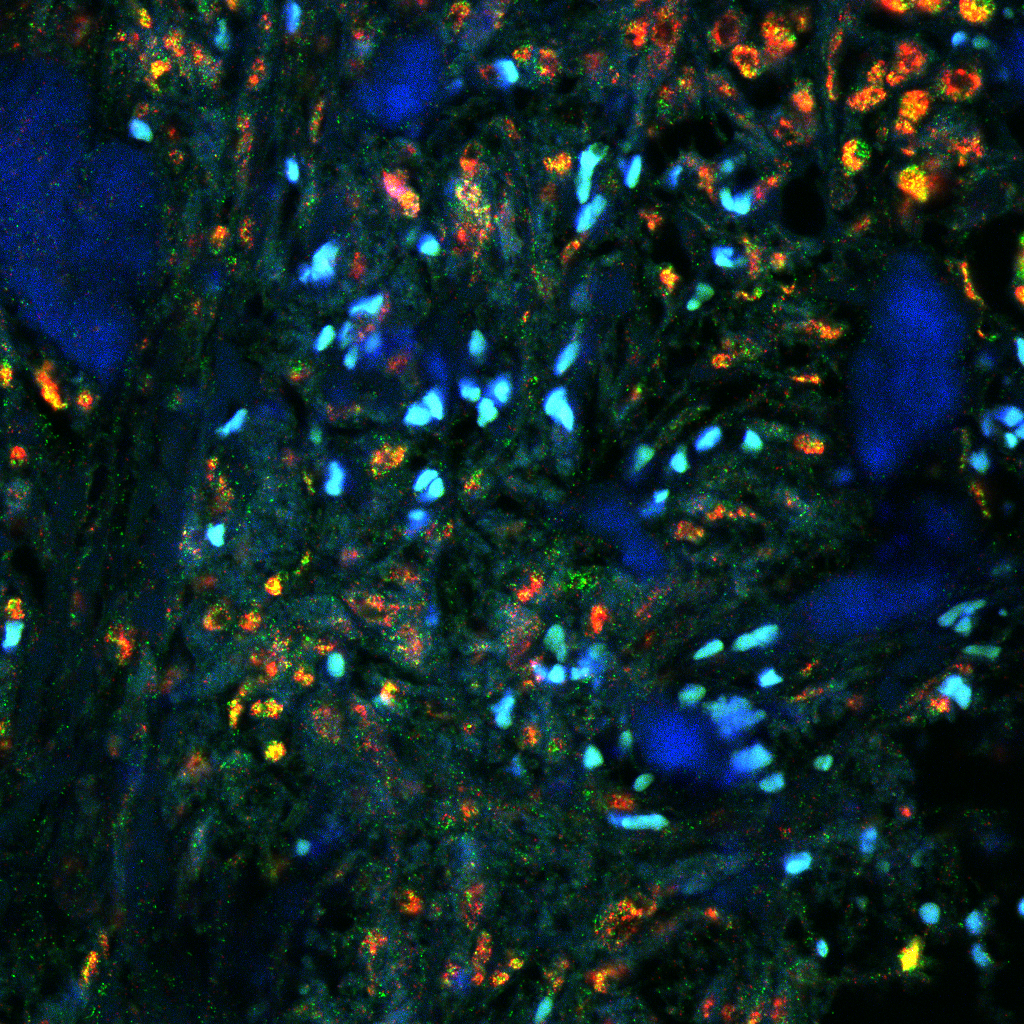

Supplement: Supplementary file 9 — Source data Fig. 4 [file 44321_2025_333_MOESM9_ESM.zip › Figure 4/4I/P02/Merge.tiff]

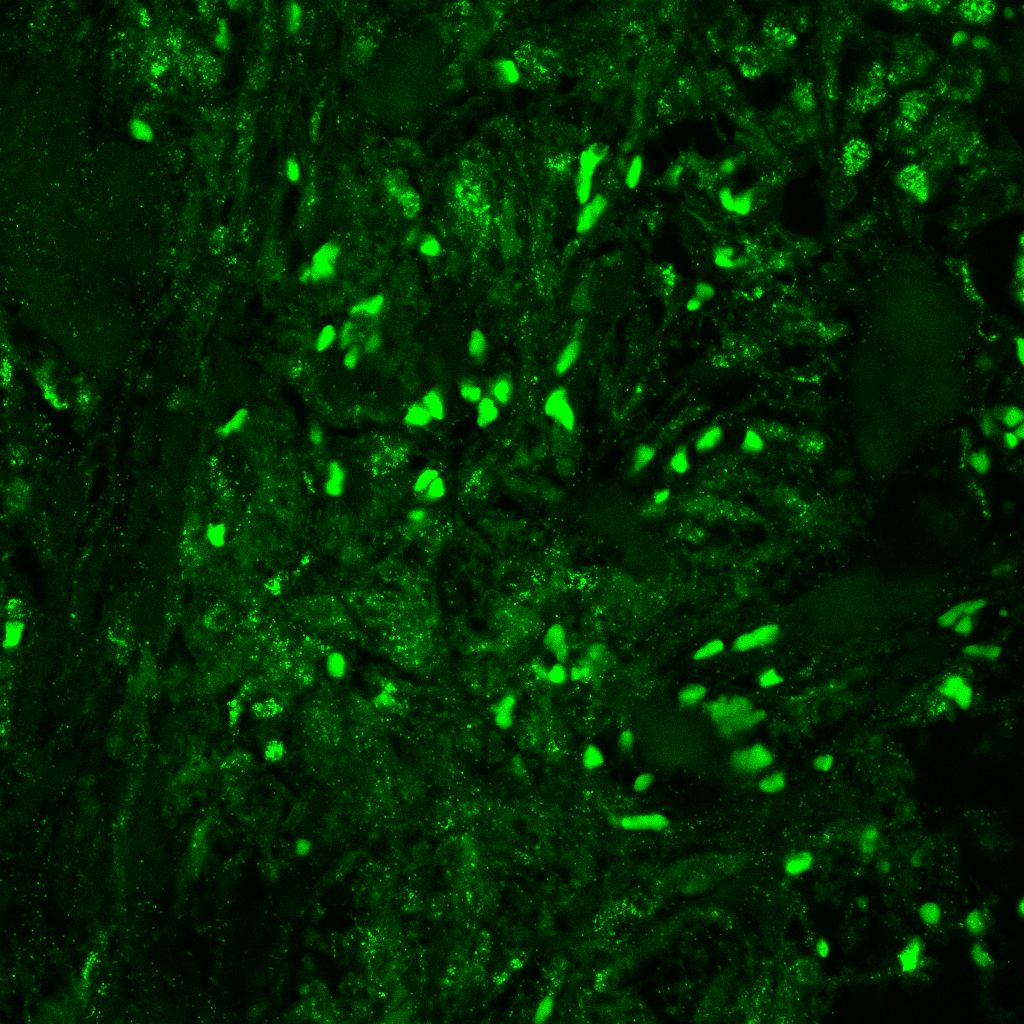

Supplement: Supplementary file 9 — Source data Fig. 4 [file 44321_2025_333_MOESM9_ESM.zip › Figure 4/4I/P02/miR-942-3p.tiff]

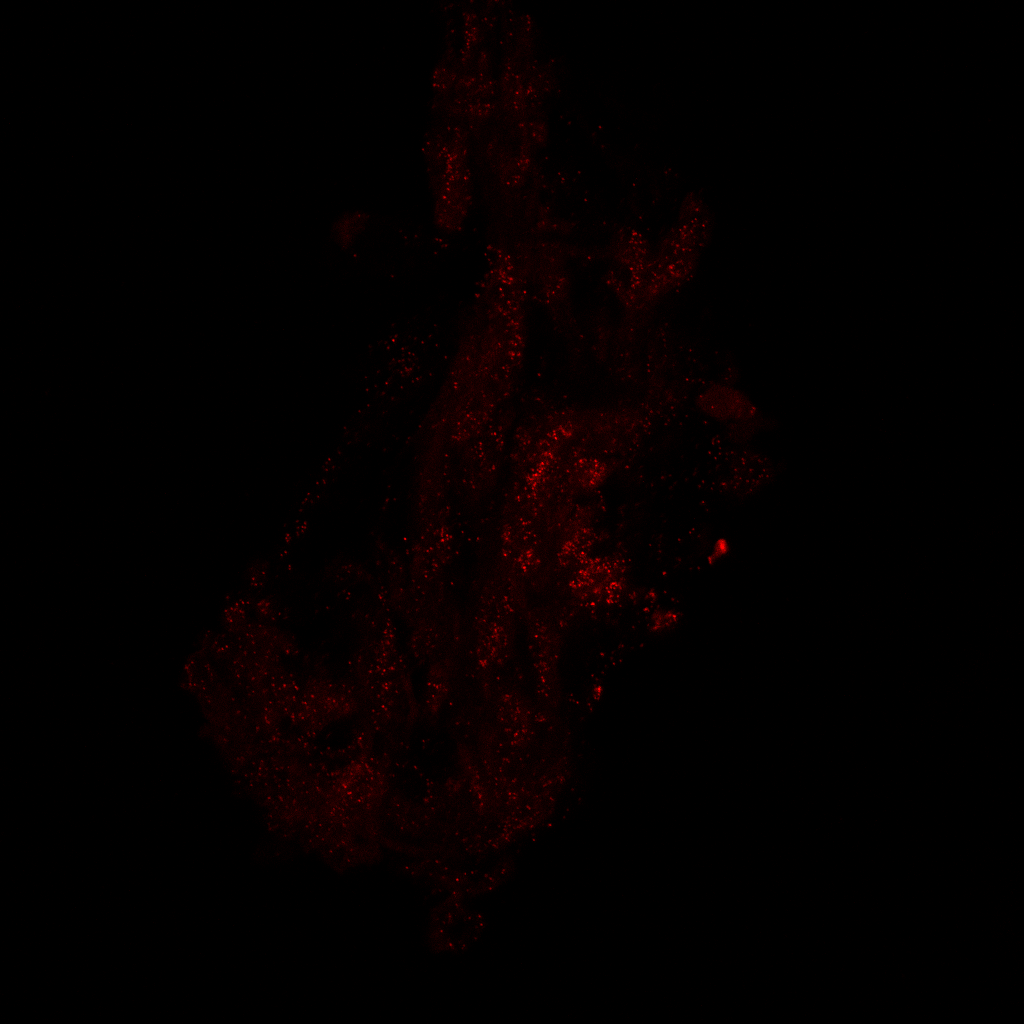

Supplement: Supplementary file 9 — Source data Fig. 4 [file 44321_2025_333_MOESM9_ESM.zip › Figure 4/4I/P03/circEGFR.tiff]

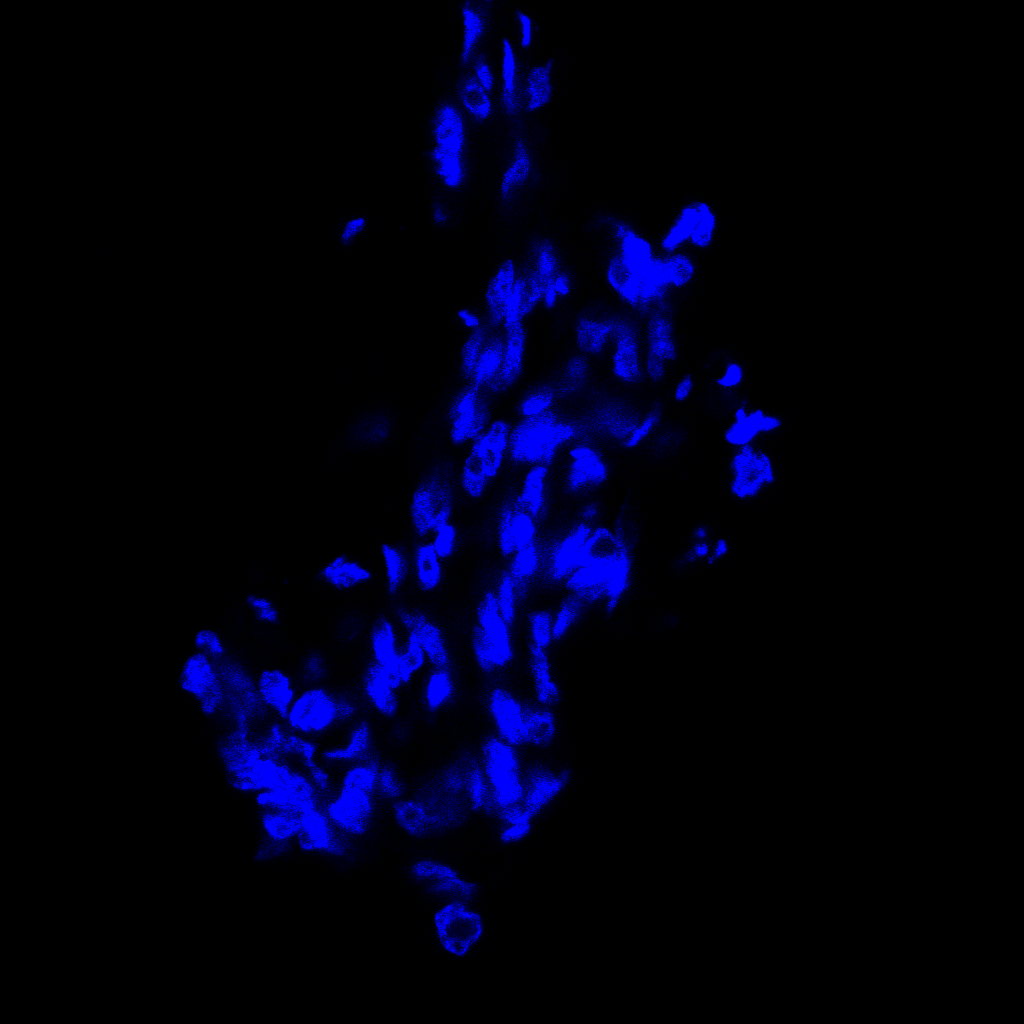

Supplement: Supplementary file 9 — Source data Fig. 4 [file 44321_2025_333_MOESM9_ESM.zip › Figure 4/4I/P03/DAPI.tiff]

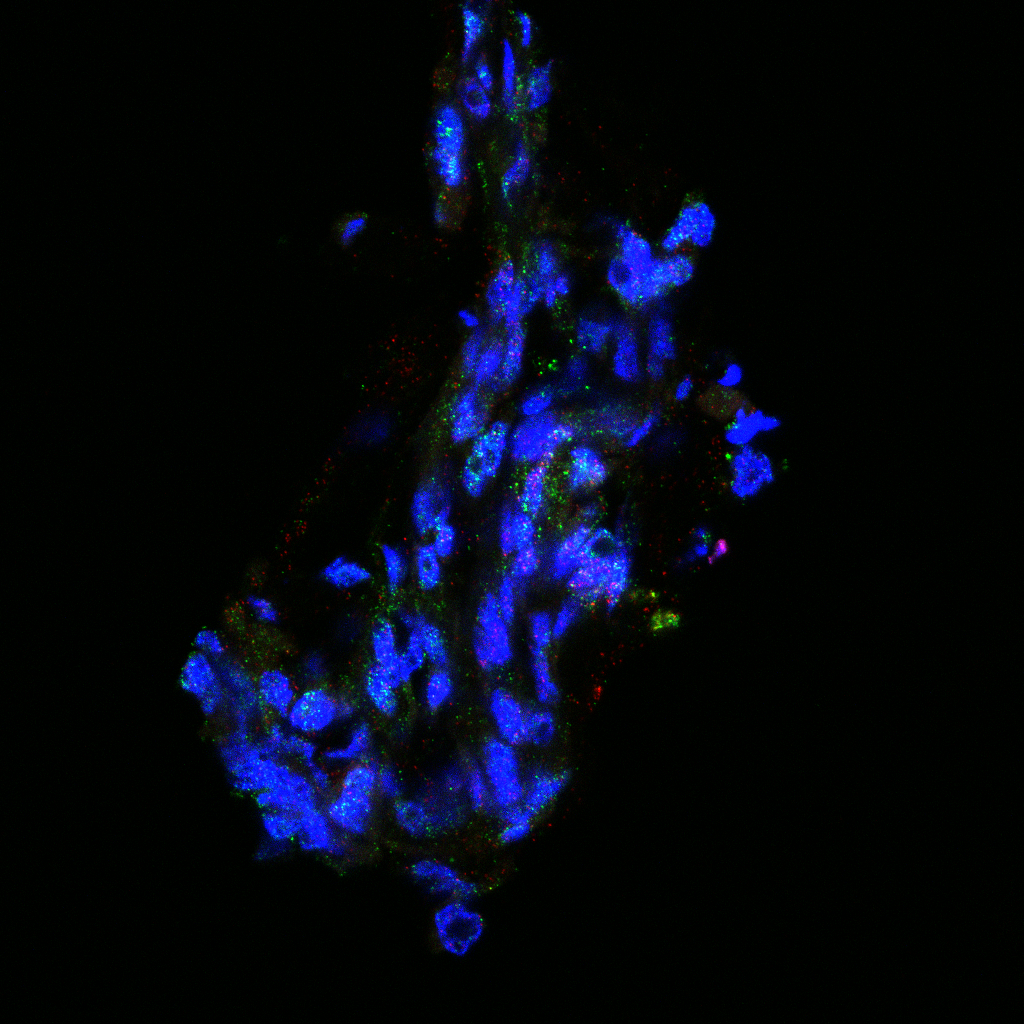

Supplement: Supplementary file 9 — Source data Fig. 4 [file 44321_2025_333_MOESM9_ESM.zip › Figure 4/4I/P03/Merge.tiff]

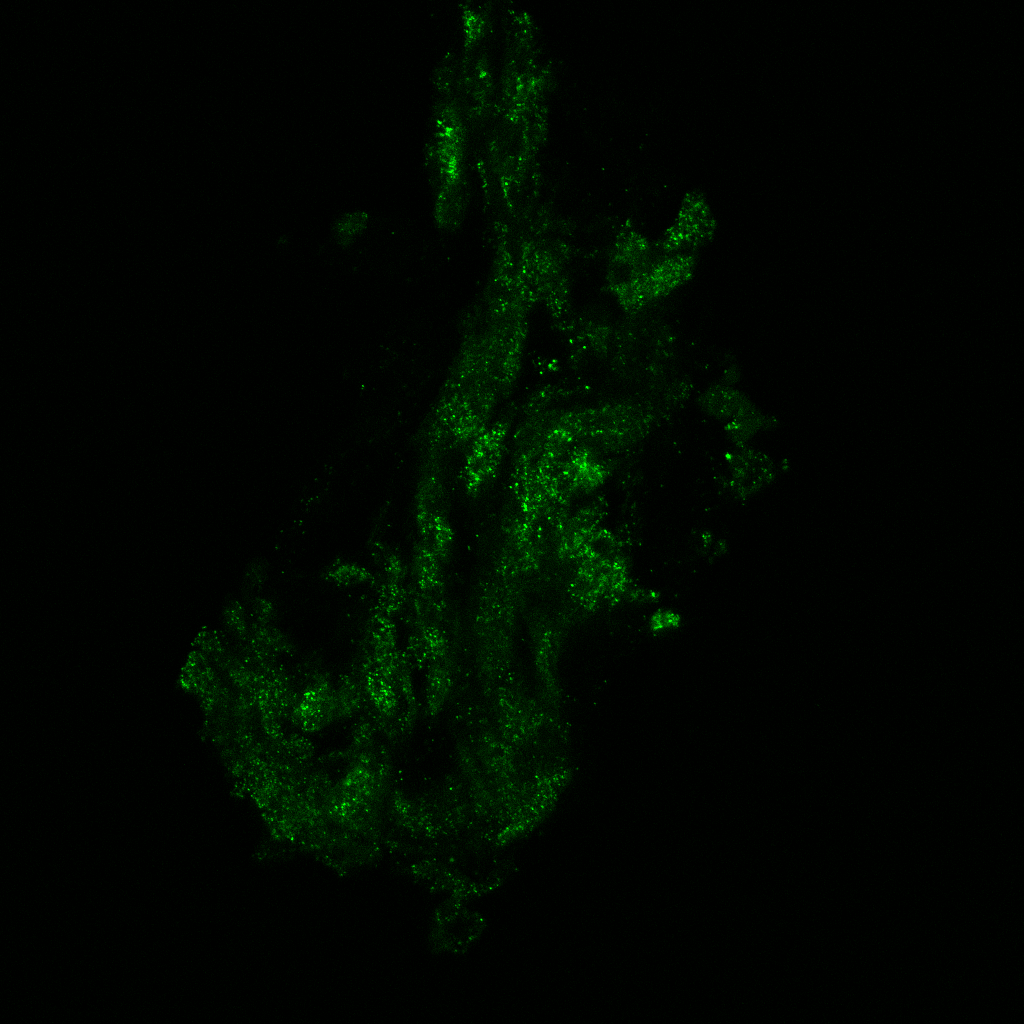

Supplement: Supplementary file 9 — Source data Fig. 4 [file 44321_2025_333_MOESM9_ESM.zip › Figure 4/4I/P03/miR-942-3p.tiff]

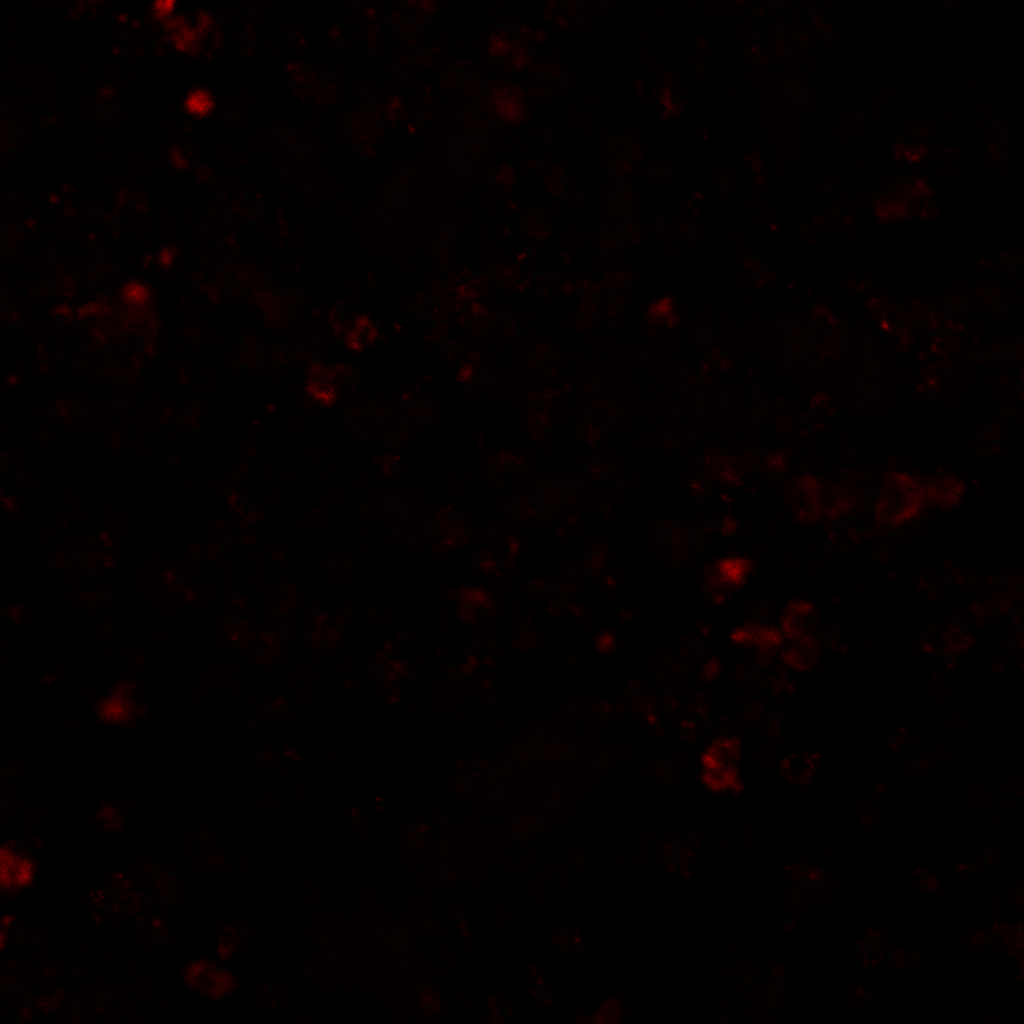

Supplement: Supplementary file 9 — Source data Fig. 4 [file 44321_2025_333_MOESM9_ESM.zip › Figure 4/4I/P04/circEGFR.tiff]

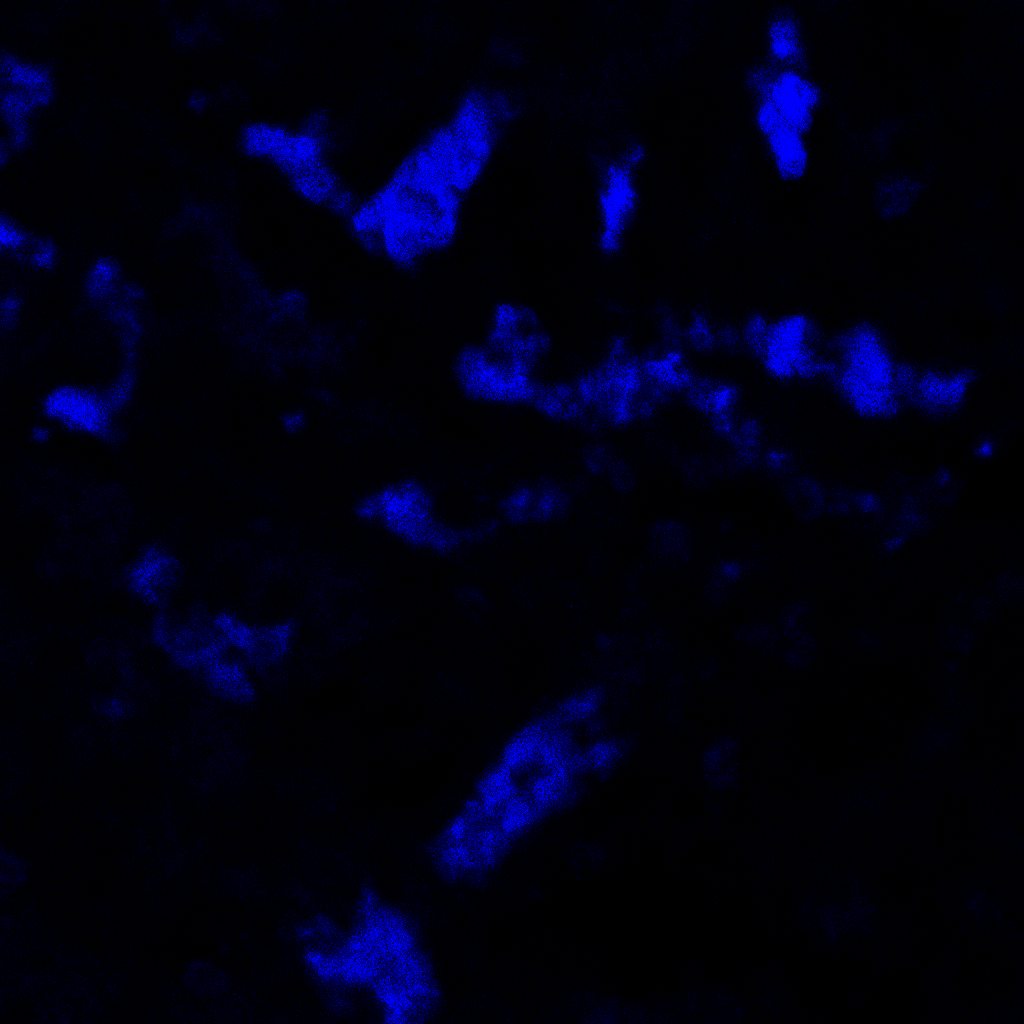

Supplement: Supplementary file 9 — Source data Fig. 4 [file 44321_2025_333_MOESM9_ESM.zip › Figure 4/4I/P04/DAPI.tiff]

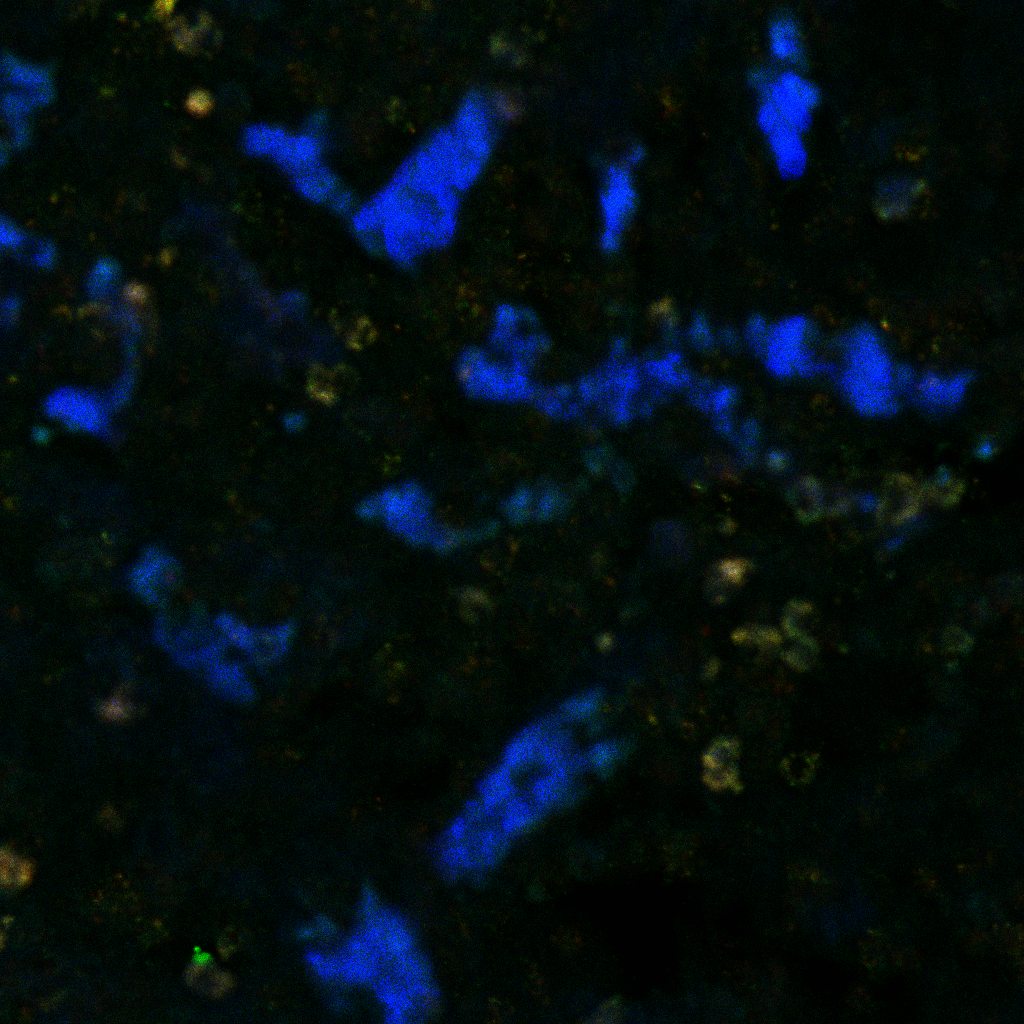

Supplement: Supplementary file 9 — Source data Fig. 4 [file 44321_2025_333_MOESM9_ESM.zip › Figure 4/4I/P04/Merge.tiff]

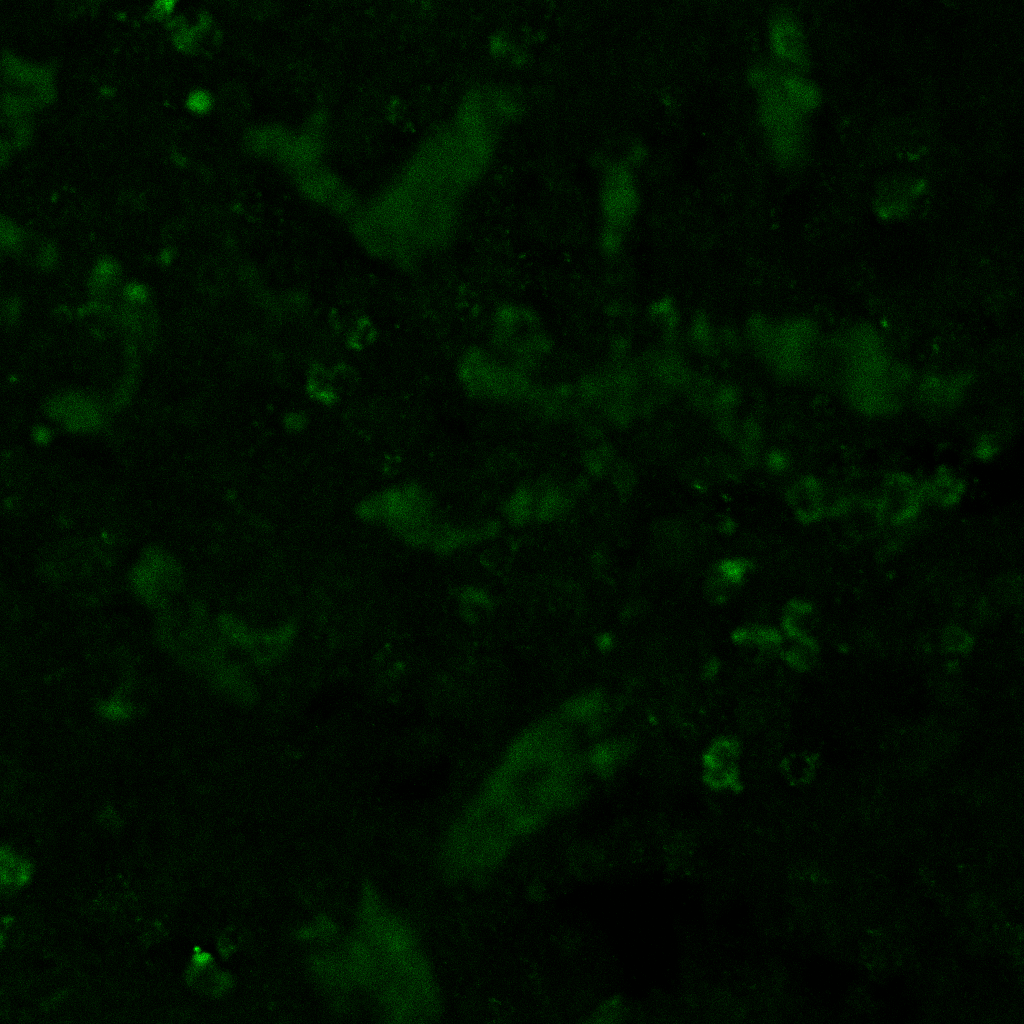

Supplement: Supplementary file 9 — Source data Fig. 4 [file 44321_2025_333_MOESM9_ESM.zip › Figure 4/4I/P04/miR-942-3p.tiff]

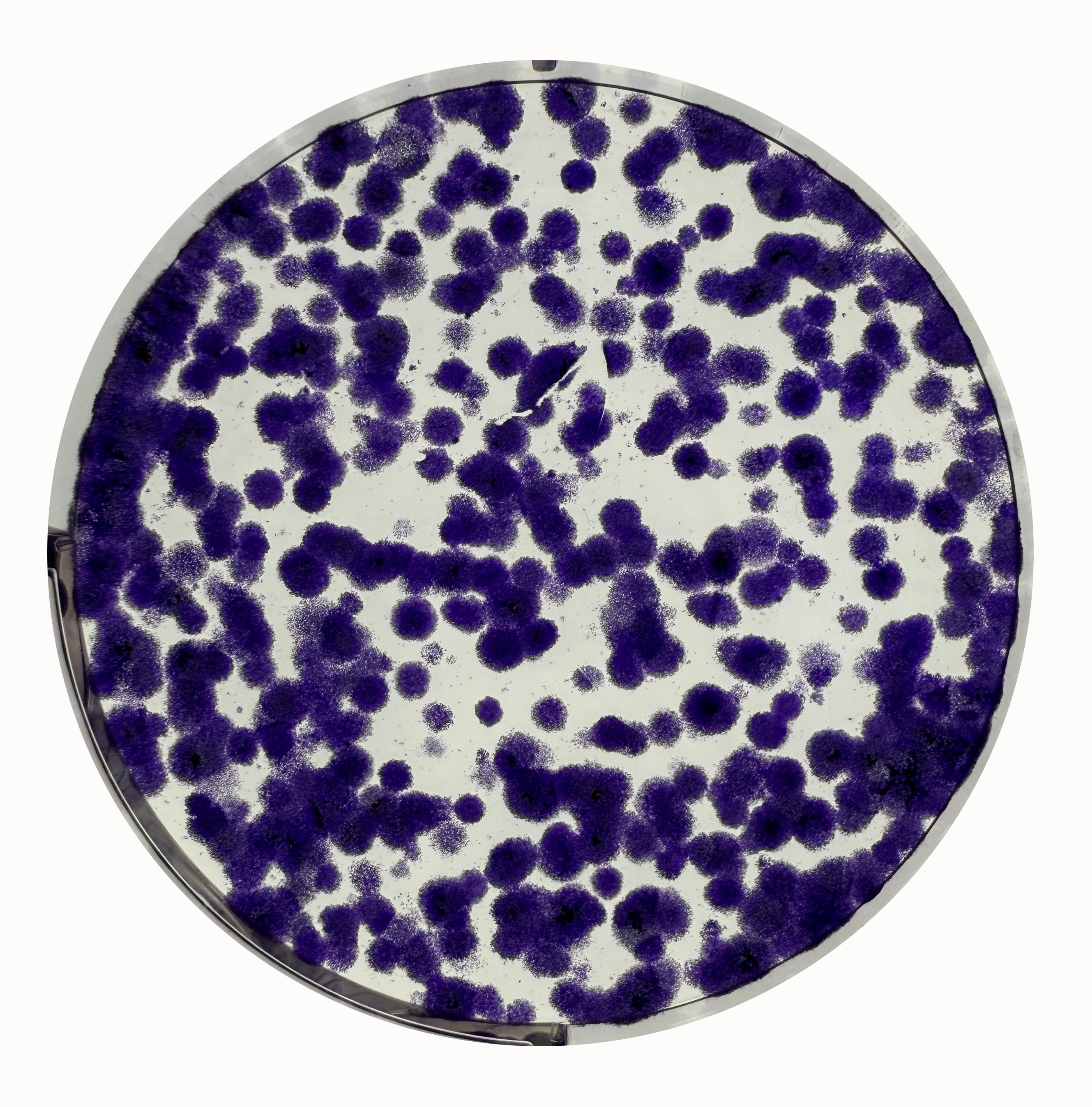

Supplement: Supplementary file 10 — Source data Fig. 5 [file 44321_2025_333_MOESM10_ESM.zip › Figure 5/5C/CaCO2/Rep 1/1_NC.tif]

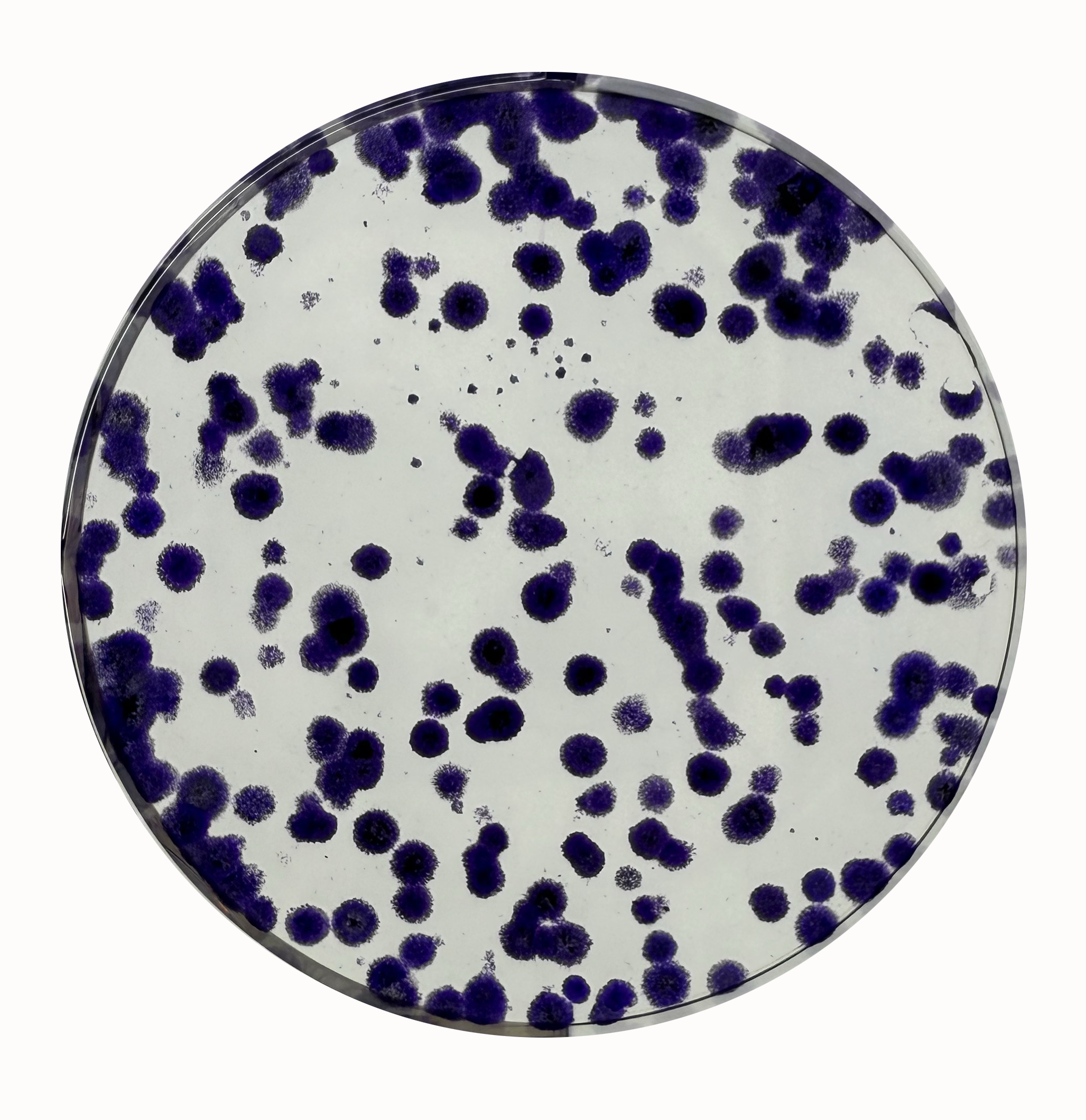

Supplement: Supplementary file 10 — Source data Fig. 5 [file 44321_2025_333_MOESM10_ESM.zip › Figure 5/5C/CaCO2/Rep 1/2_OE circ.tif]

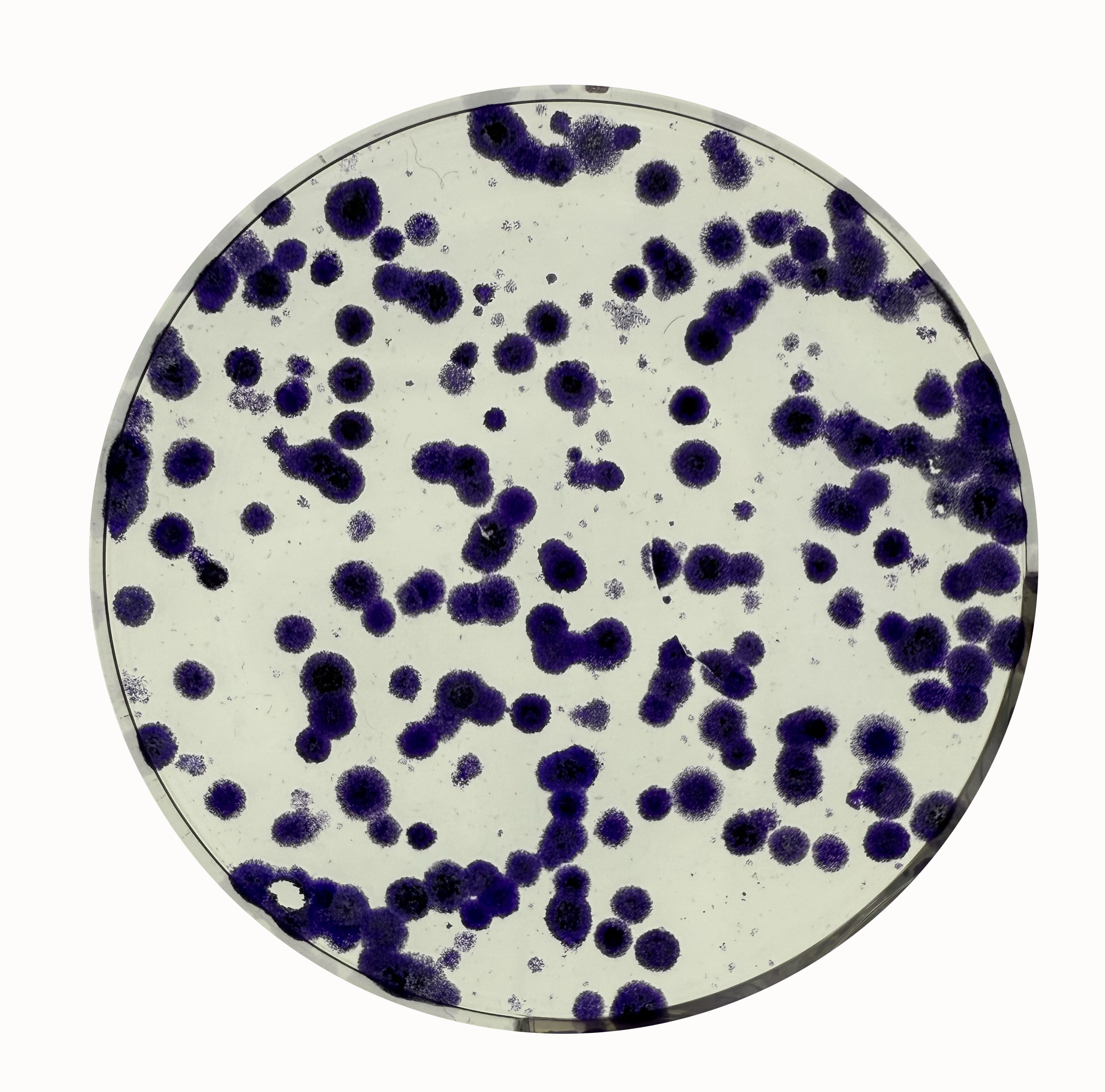

Supplement: Supplementary file 10 — Source data Fig. 5 [file 44321_2025_333_MOESM10_ESM.zip › Figure 5/5C/CaCO2/Rep 1/3_OE+NC.tif]

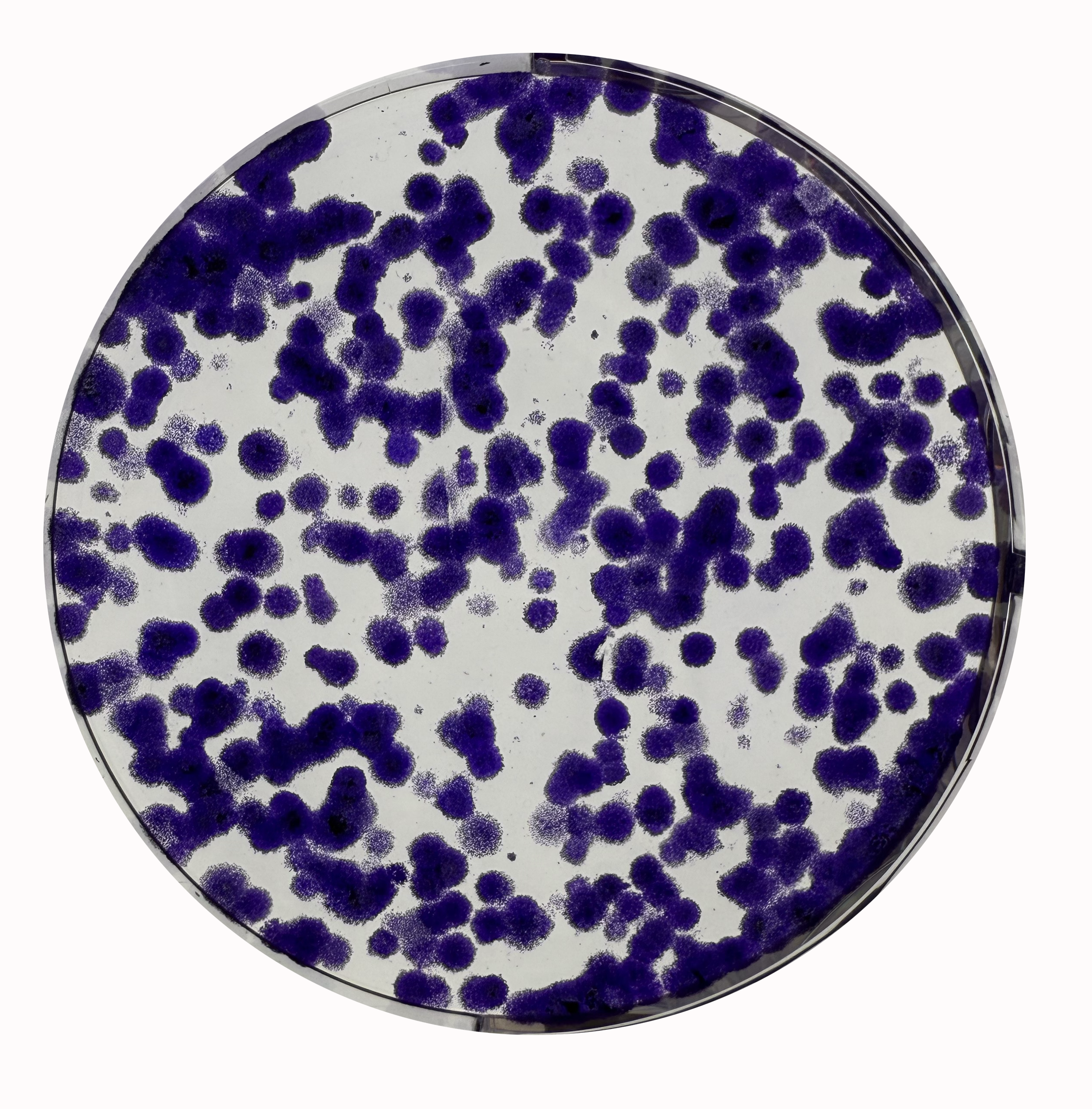

Supplement: Supplementary file 10 — Source data Fig. 5 [file 44321_2025_333_MOESM10_ESM.zip › Figure 5/5C/CaCO2/Rep 1/4_OE+OE.tif]

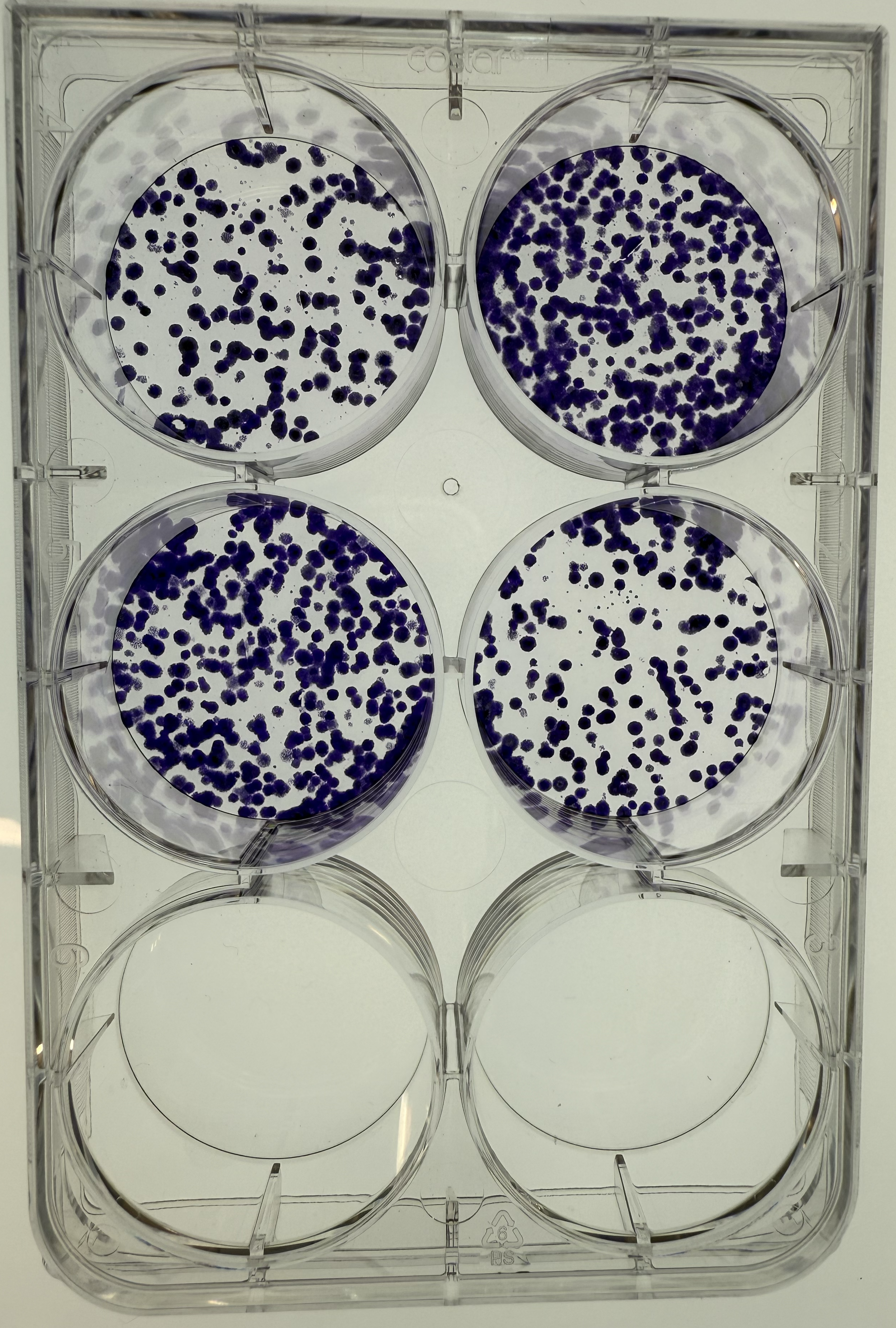

Supplement: Supplementary file 10 — Source data Fig. 5 [file 44321_2025_333_MOESM10_ESM.zip › Figure 5/5C/CaCO2/Rep 1/Overall.tif]

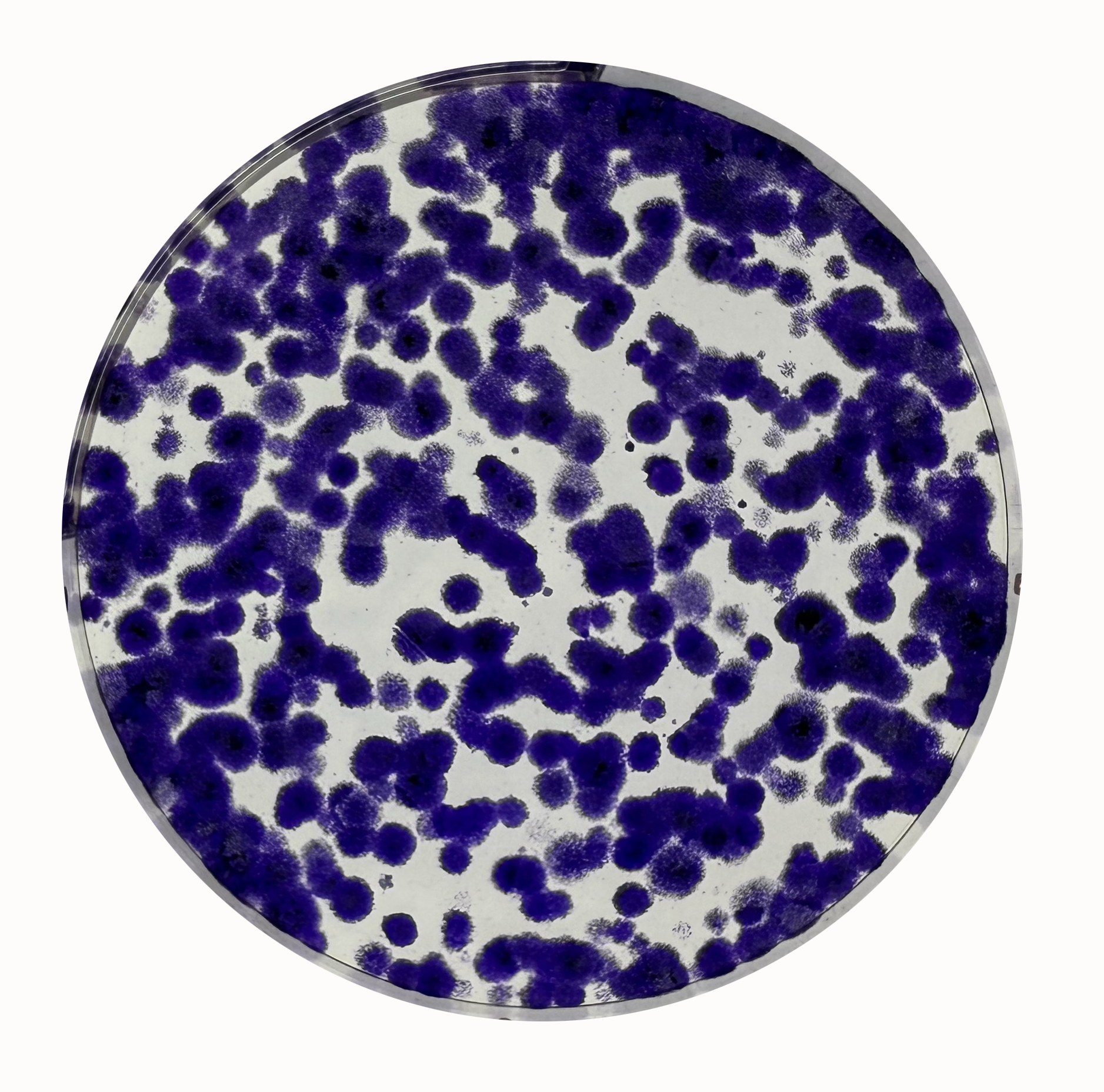

Supplement: Supplementary file 10 — Source data Fig. 5 [file 44321_2025_333_MOESM10_ESM.zip › Figure 5/5C/CaCO2/Rep 2/1_NC.tif]

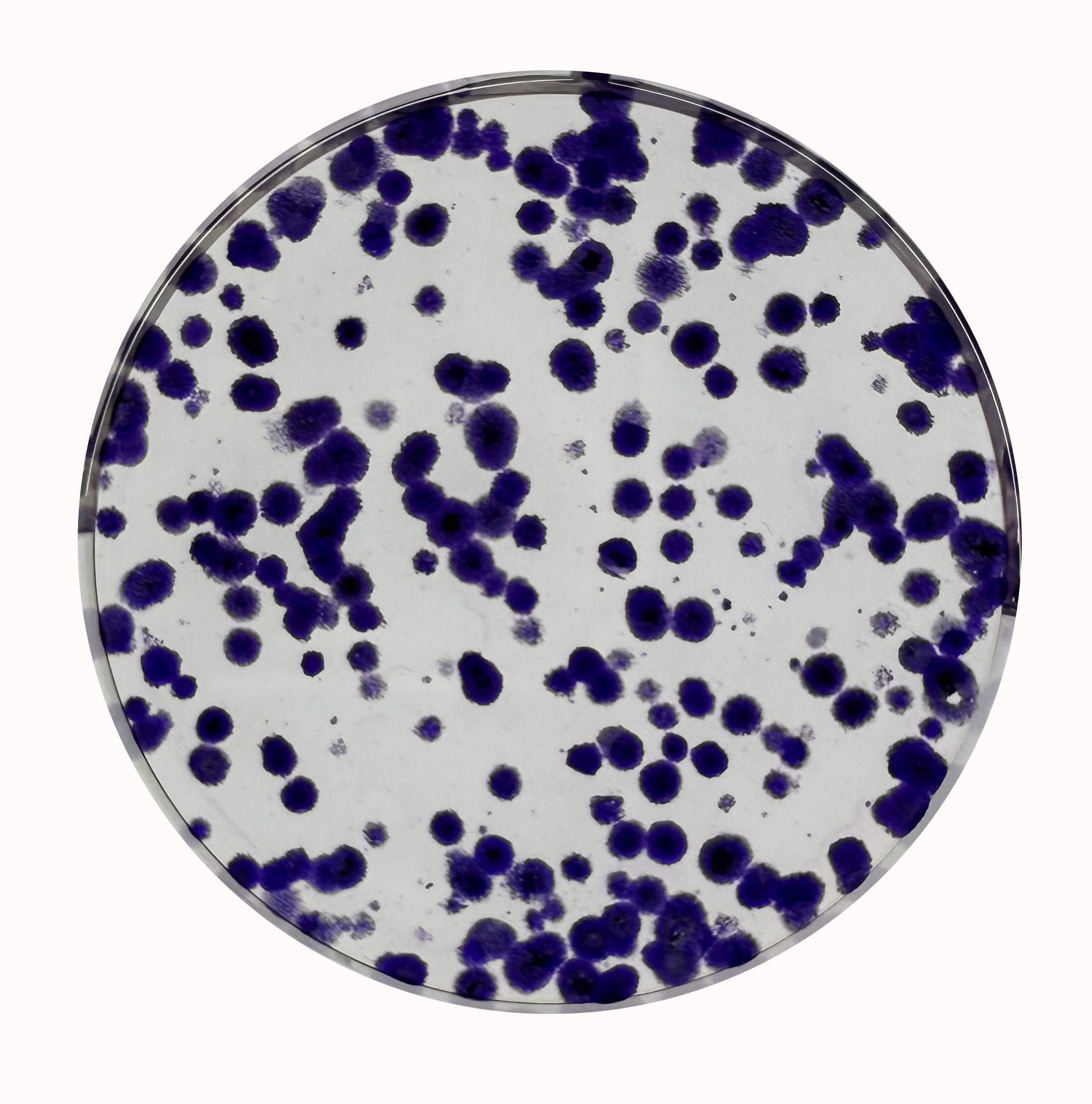

Supplement: Supplementary file 10 — Source data Fig. 5 [file 44321_2025_333_MOESM10_ESM.zip › Figure 5/5C/CaCO2/Rep 2/2_OE.tif]

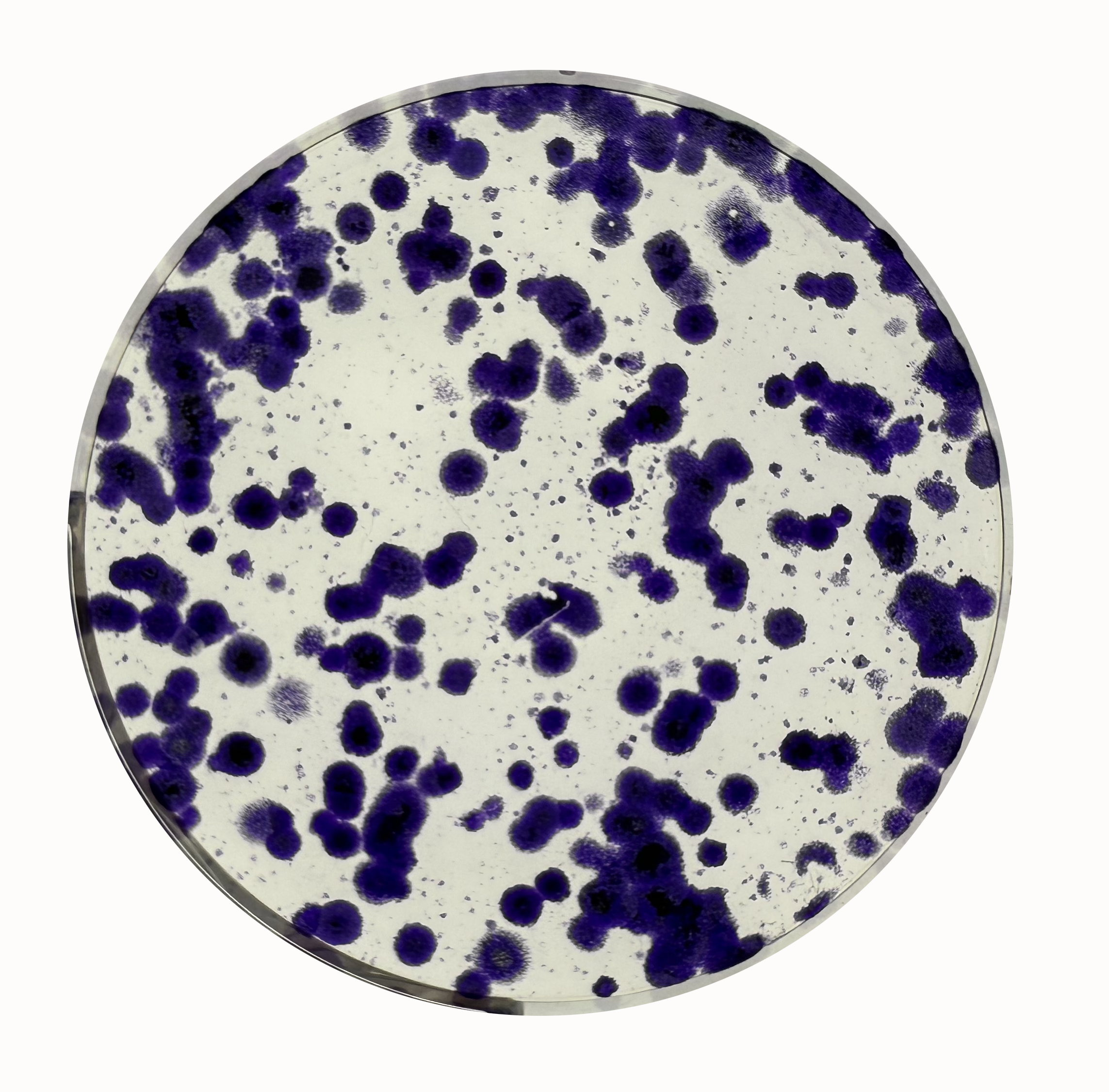

Supplement: Supplementary file 10 — Source data Fig. 5 [file 44321_2025_333_MOESM10_ESM.zip › Figure 5/5C/CaCO2/Rep 2/3_OE+NC.tif]

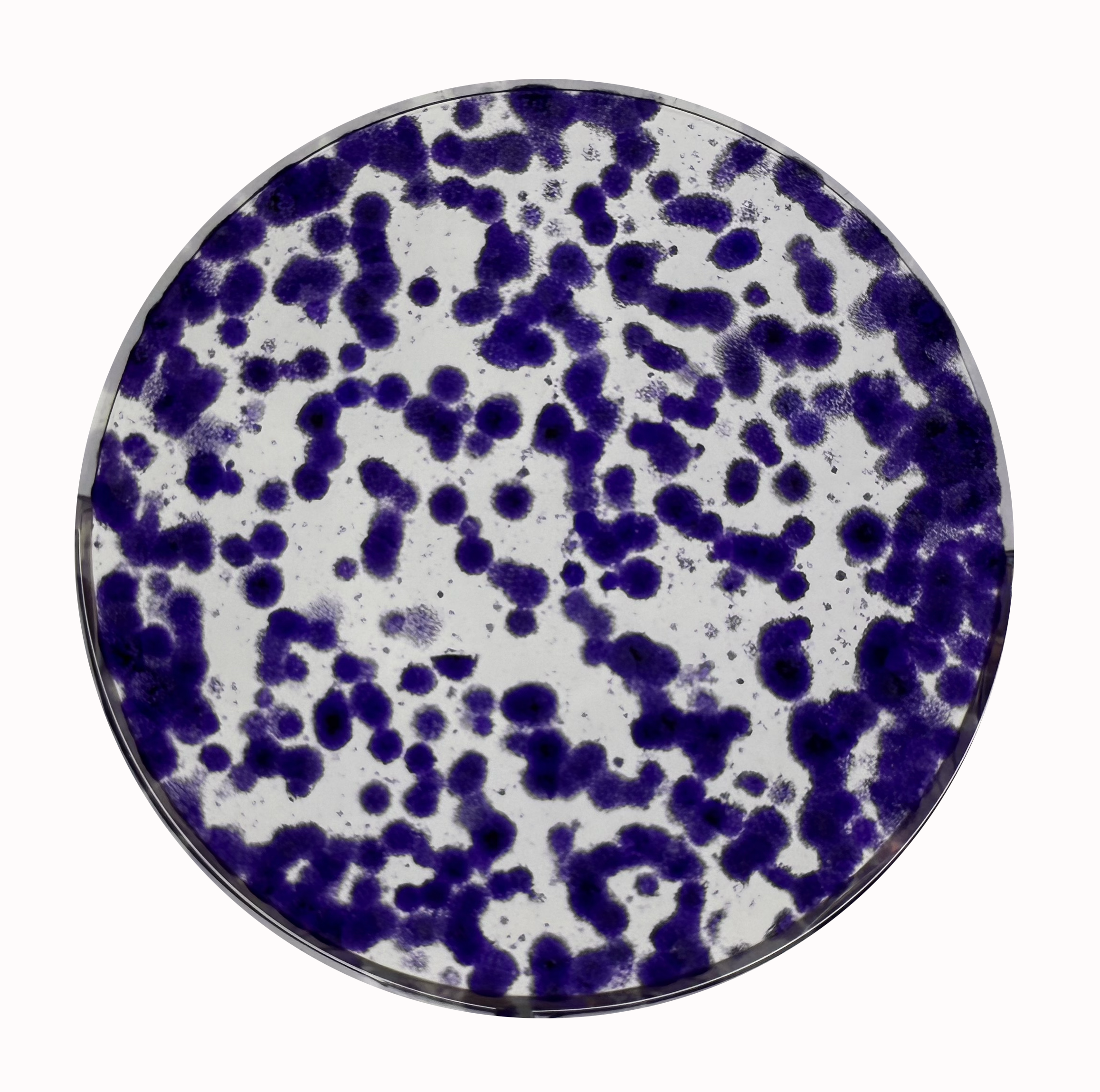

Supplement: Supplementary file 10 — Source data Fig. 5 [file 44321_2025_333_MOESM10_ESM.zip › Figure 5/5C/CaCO2/Rep 2/4_OE.tif]

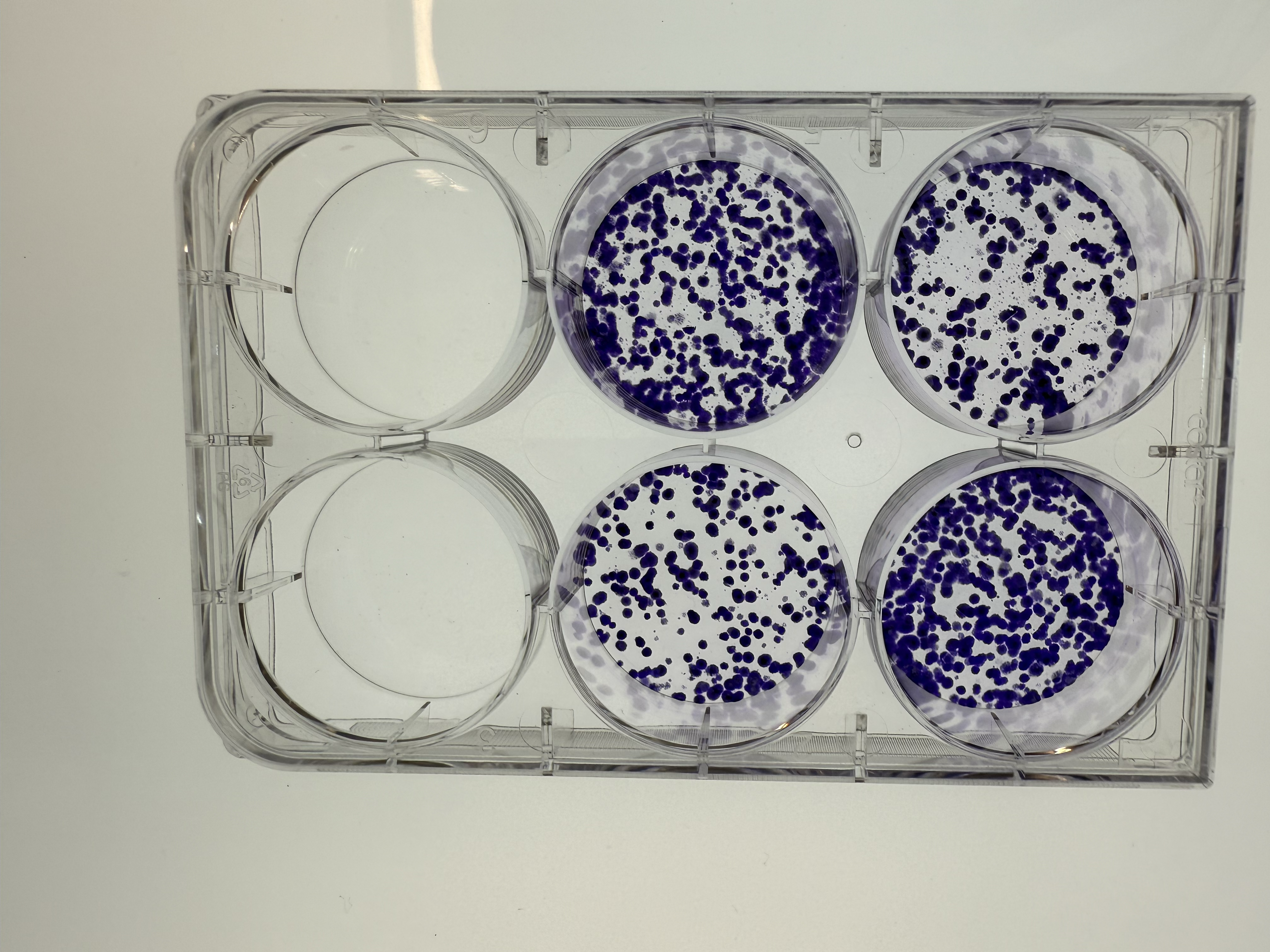

Supplement: Supplementary file 10 — Source data Fig. 5 [file 44321_2025_333_MOESM10_ESM.zip › Figure 5/5C/CaCO2/Rep 2/Overall.tif]

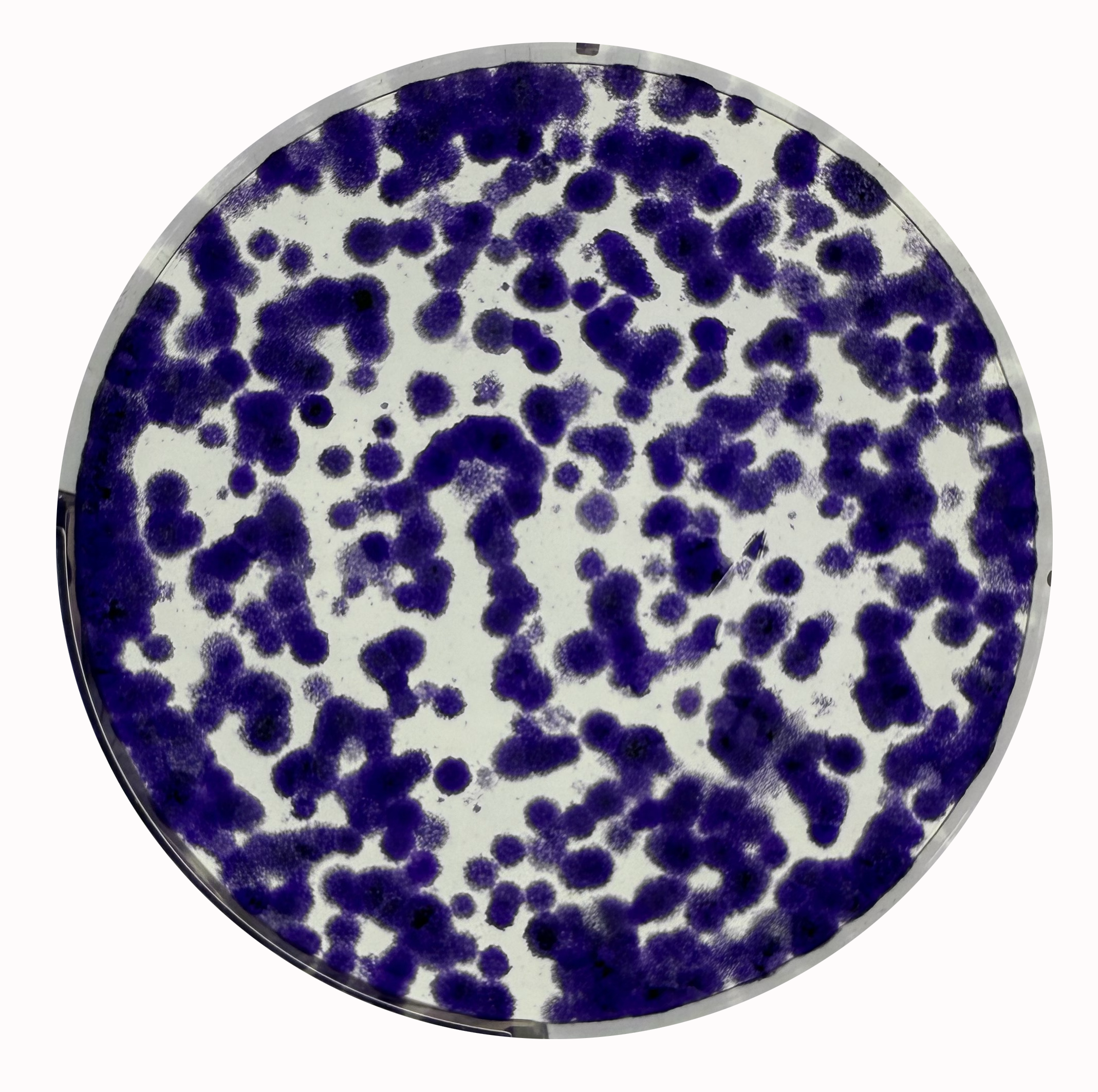

Supplement: Supplementary file 10 — Source data Fig. 5 [file 44321_2025_333_MOESM10_ESM.zip › Figure 5/5C/CaCO2/Rep 3/1_NC.tif]

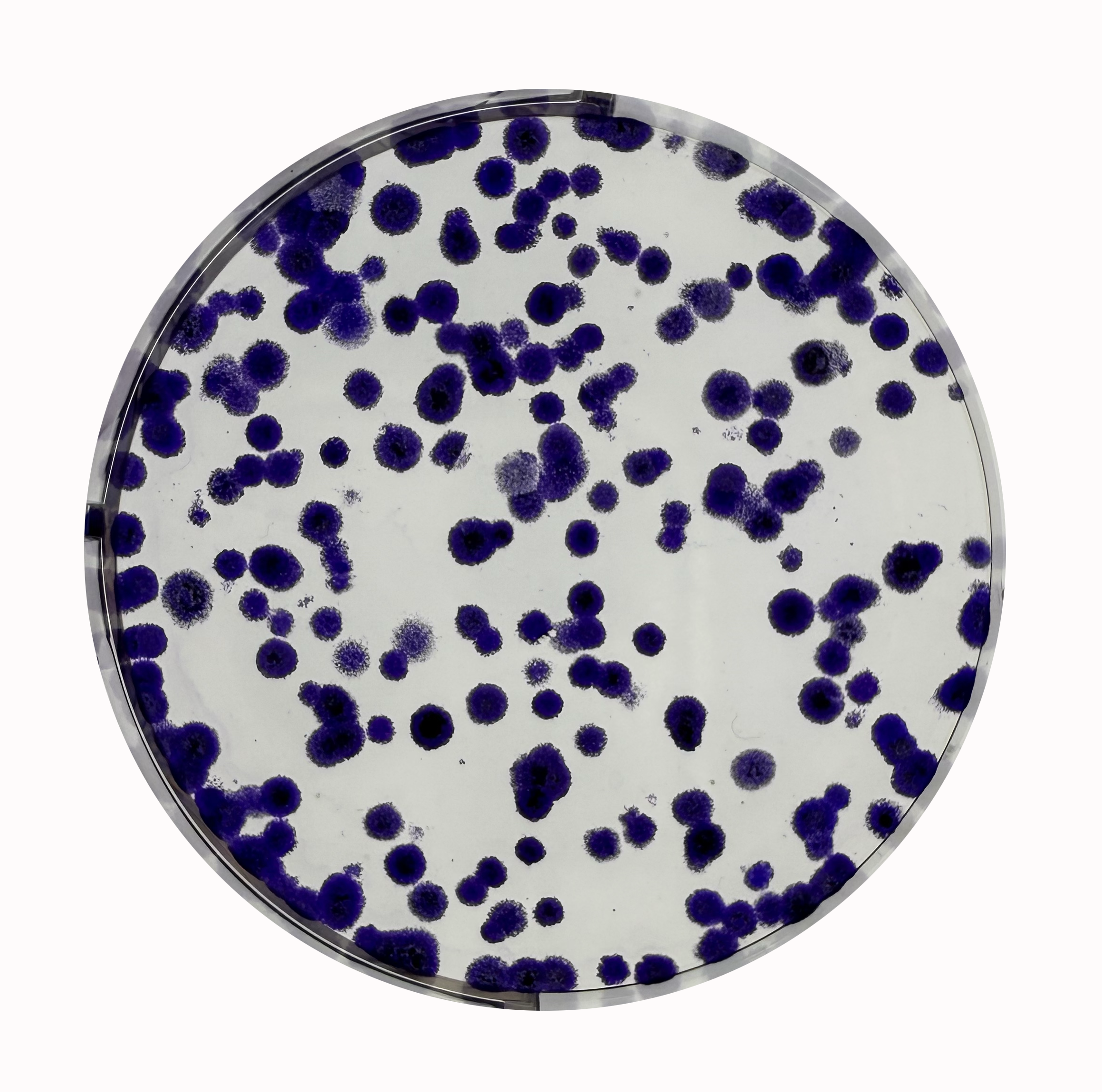

Supplement: Supplementary file 10 — Source data Fig. 5 [file 44321_2025_333_MOESM10_ESM.zip › Figure 5/5C/CaCO2/Rep 3/2_OE circ.tif]

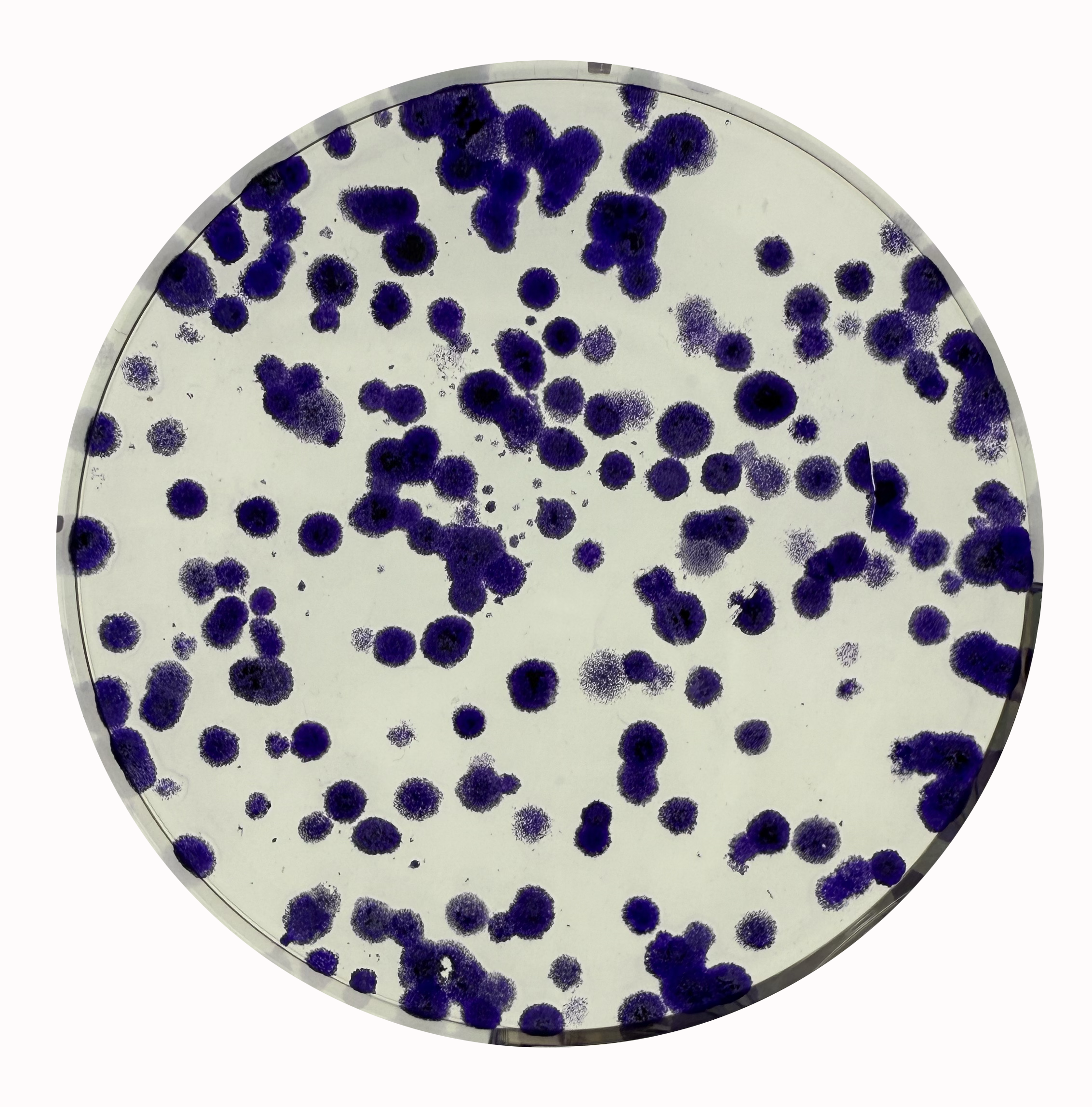

Supplement: Supplementary file 10 — Source data Fig. 5 [file 44321_2025_333_MOESM10_ESM.zip › Figure 5/5C/CaCO2/Rep 3/3_OE+NC.tif]

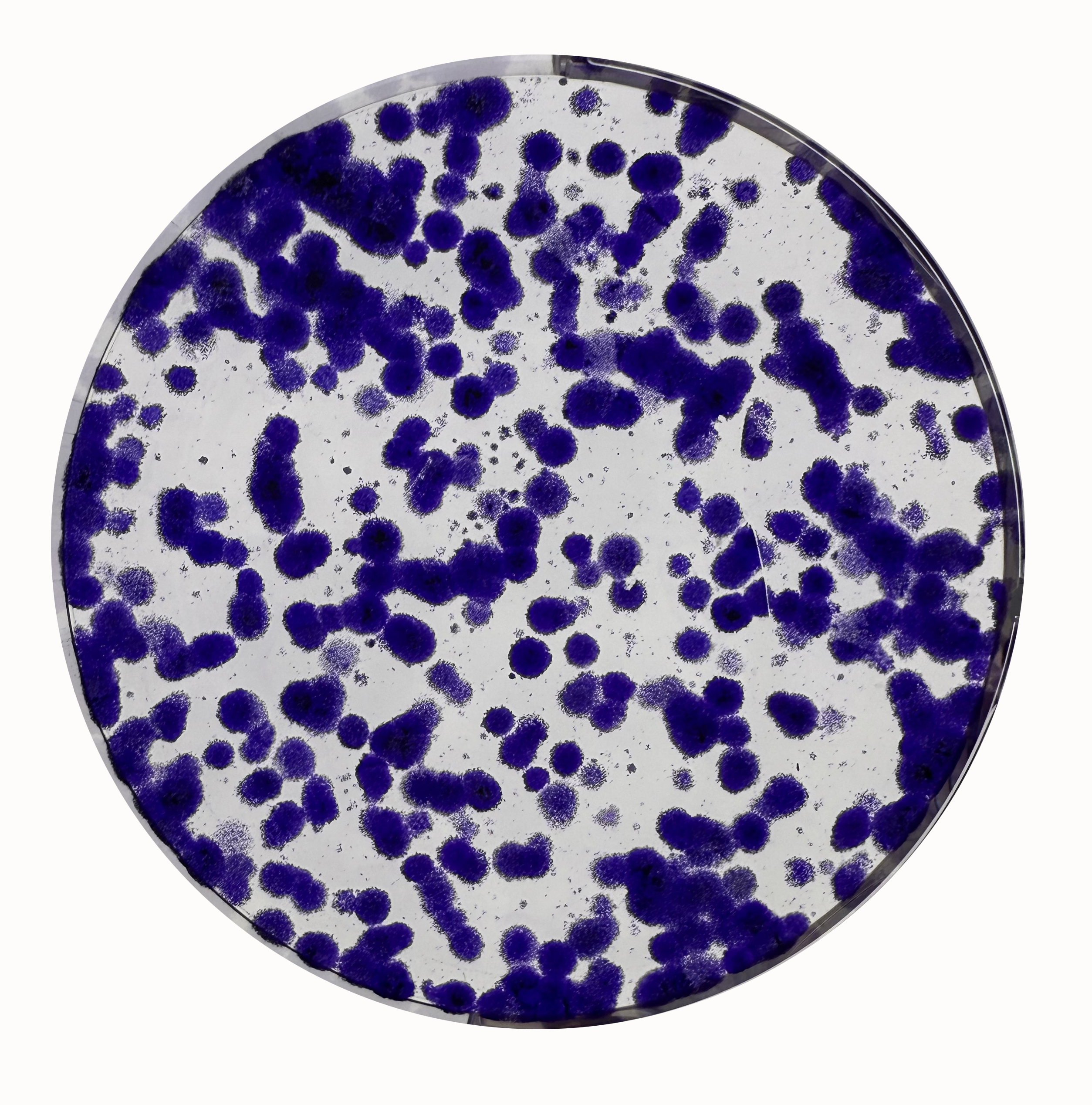

Supplement: Supplementary file 10 — Source data Fig. 5 [file 44321_2025_333_MOESM10_ESM.zip › Figure 5/5C/CaCO2/Rep 3/4_OE+OE.tif]

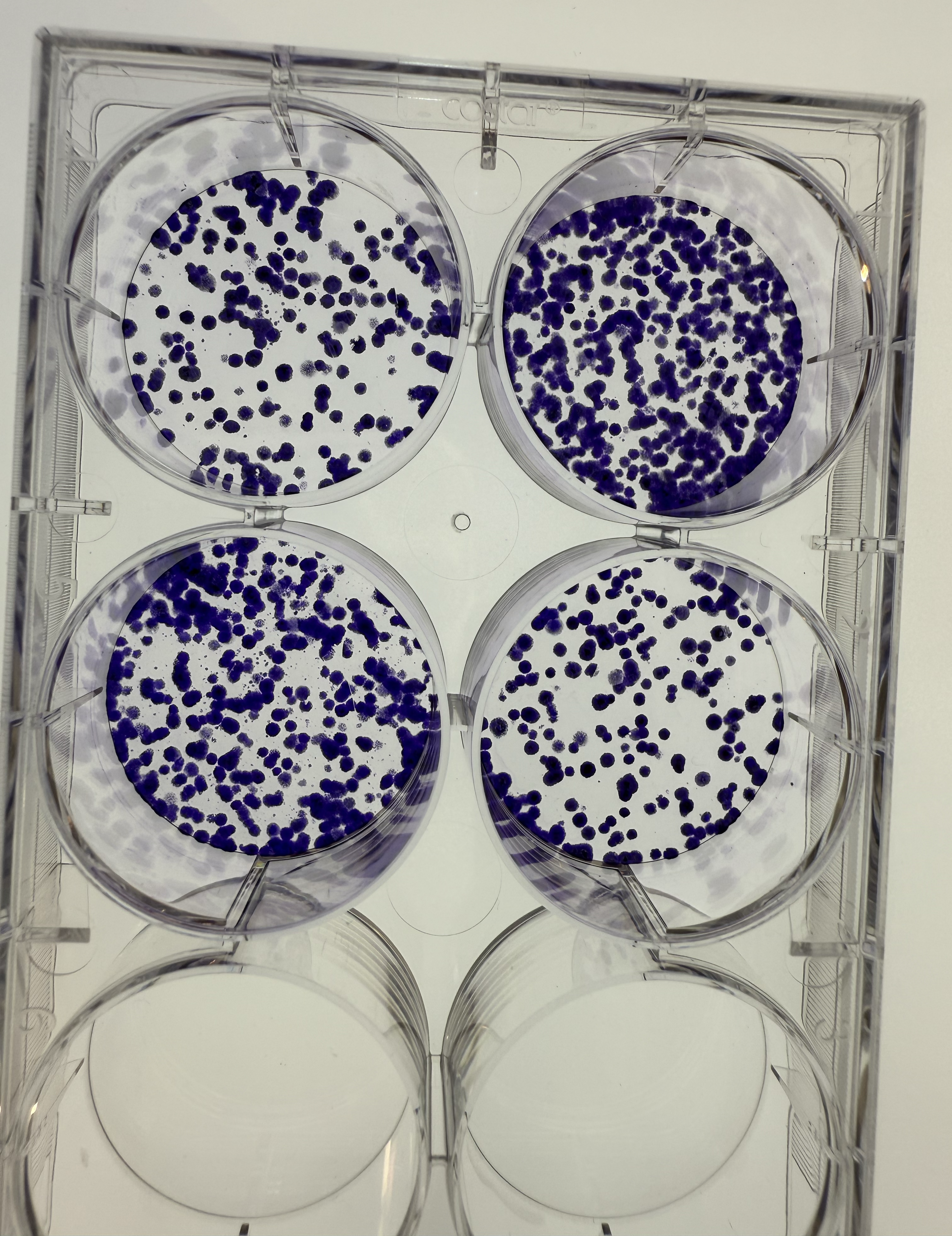

Supplement: Supplementary file 10 — Source data Fig. 5 [file 44321_2025_333_MOESM10_ESM.zip › Figure 5/5C/CaCO2/Rep 3/Overall.tif]

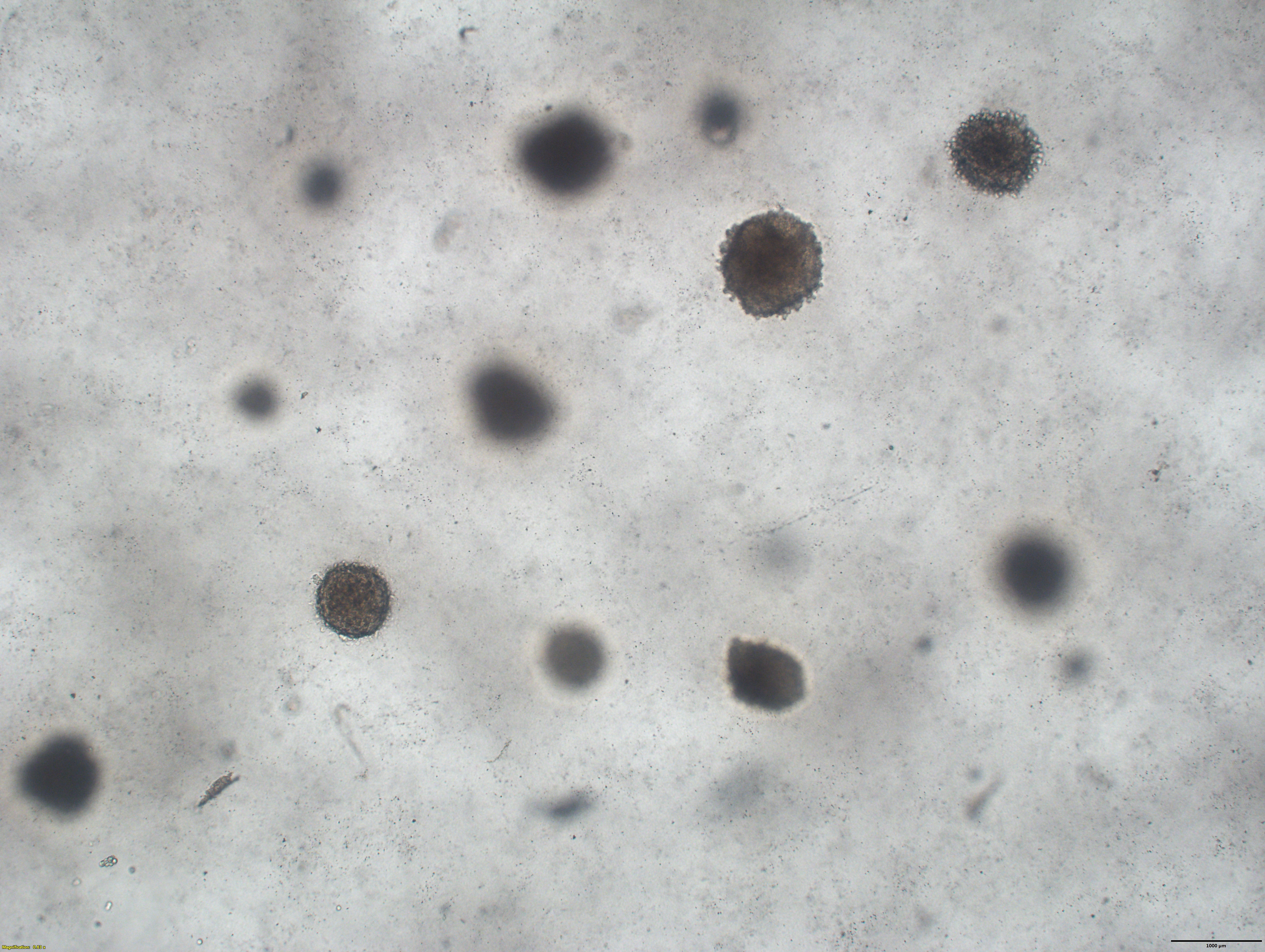

Supplement: Supplementary file 10 — Source data Fig. 5 [file 44321_2025_333_MOESM10_ESM.zip › Figure 5/5C/SNU-C1/Rep 1/1_NC.jpg]

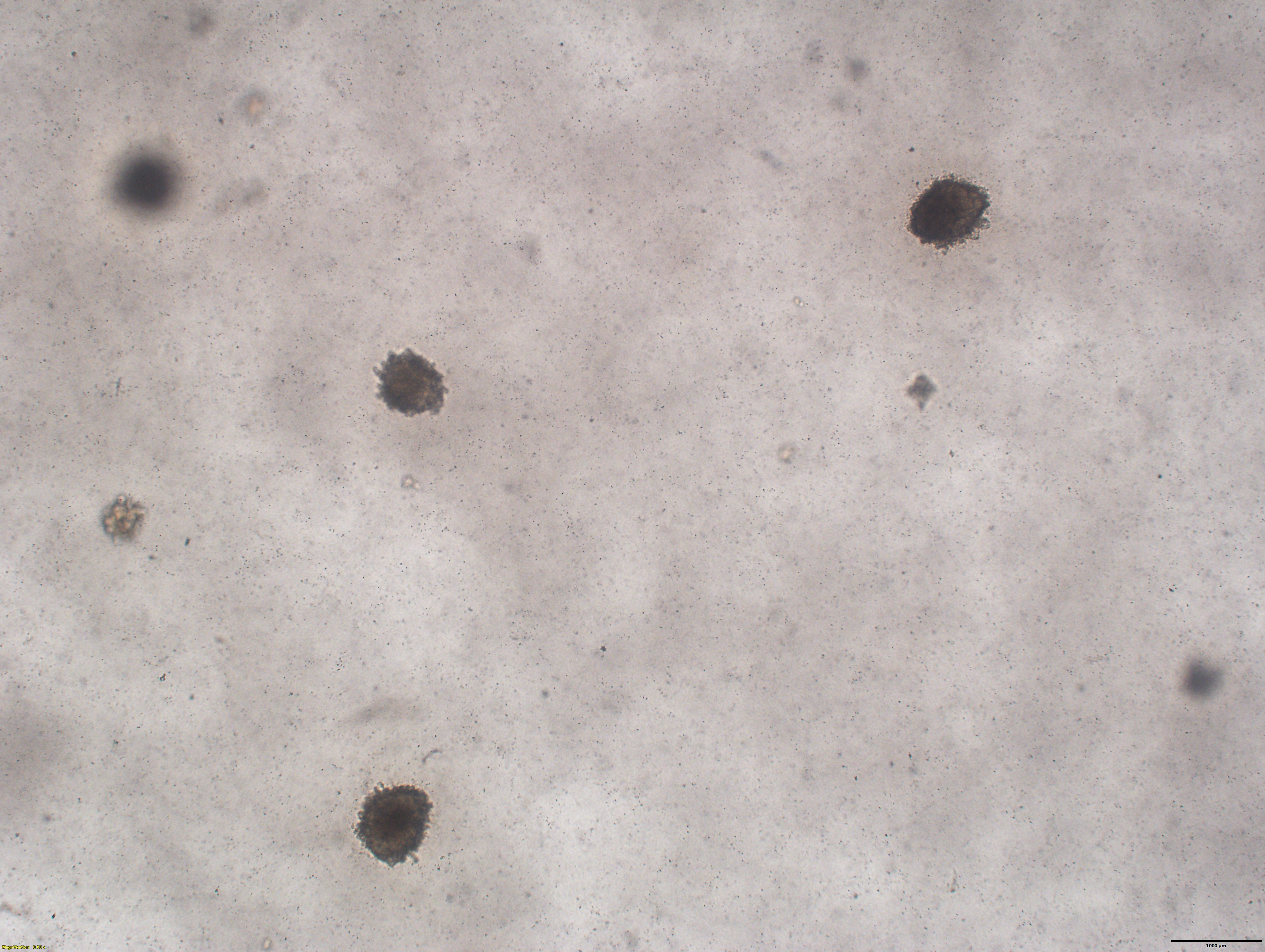

Supplement: Supplementary file 10 — Source data Fig. 5 [file 44321_2025_333_MOESM10_ESM.zip › Figure 5/5C/SNU-C1/Rep 1/2_OE.jpg]

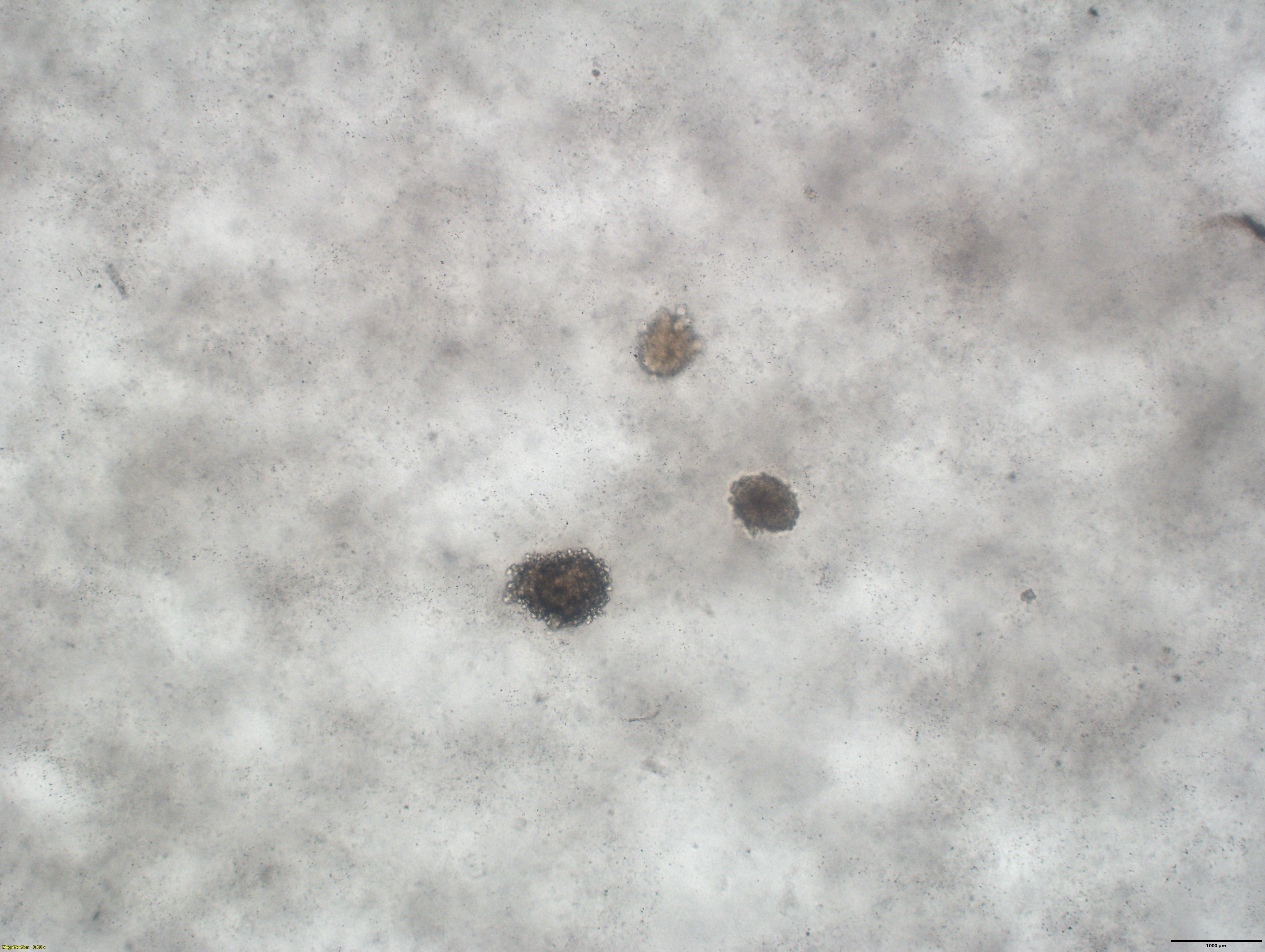

Supplement: Supplementary file 10 — Source data Fig. 5 [file 44321_2025_333_MOESM10_ESM.zip › Figure 5/5C/SNU-C1/Rep 1/3_OE+NC.jpg]
