# Supplementary material for: circ-EGFR is a predictor of response to Cetuximab and a potential target in colorectal cancer
Source: EMBO Mol Med. 2025 Nov 10;17(12):3525–54. doi: 10.1038/s44321-025-00333-0 (PMC12686431; doi:10.1038/s44321-025-00333-0)

CaCO2 apoptosis assay

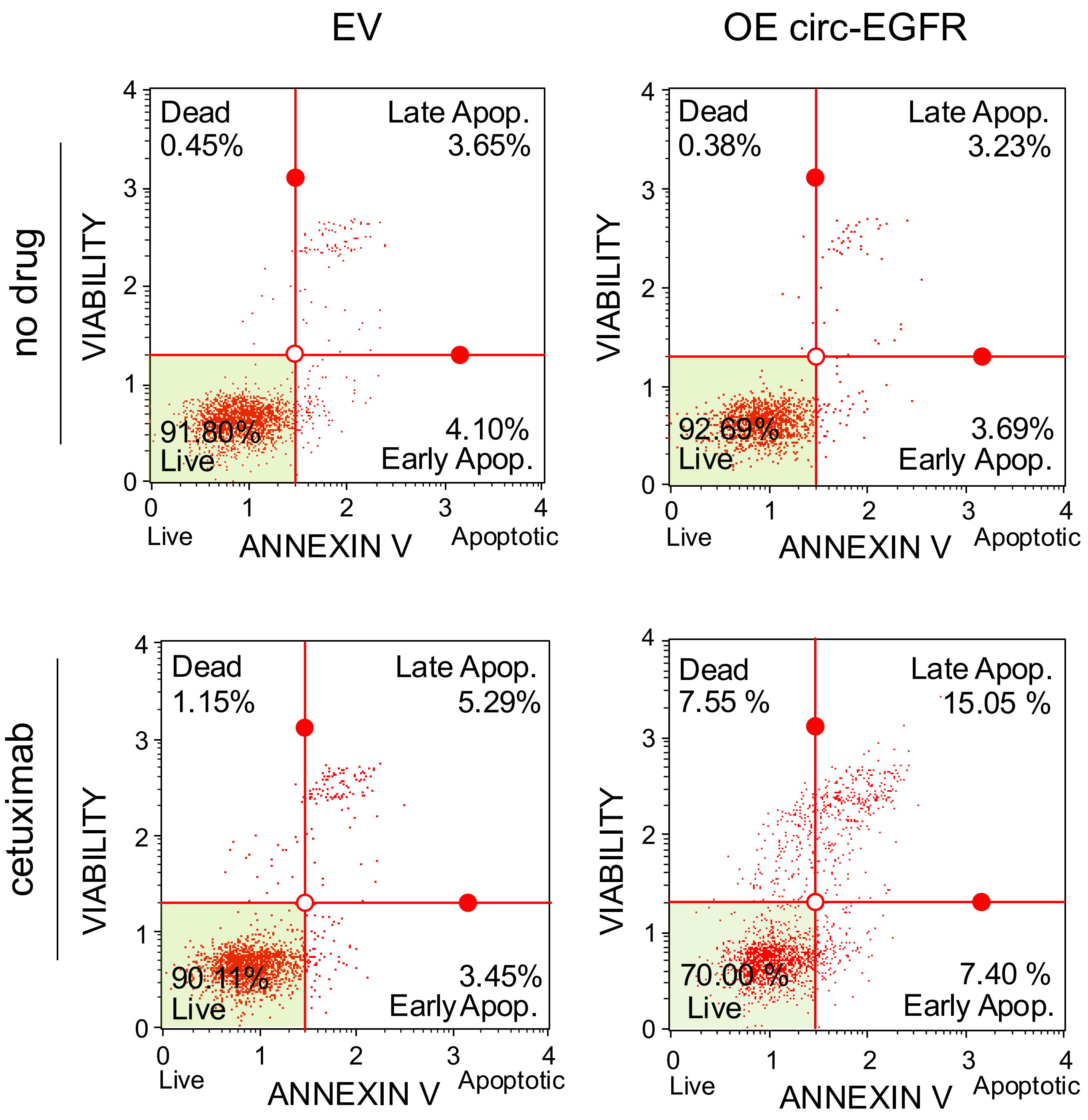

CaCO2 apoptosis assay (Duplicate Experiment)

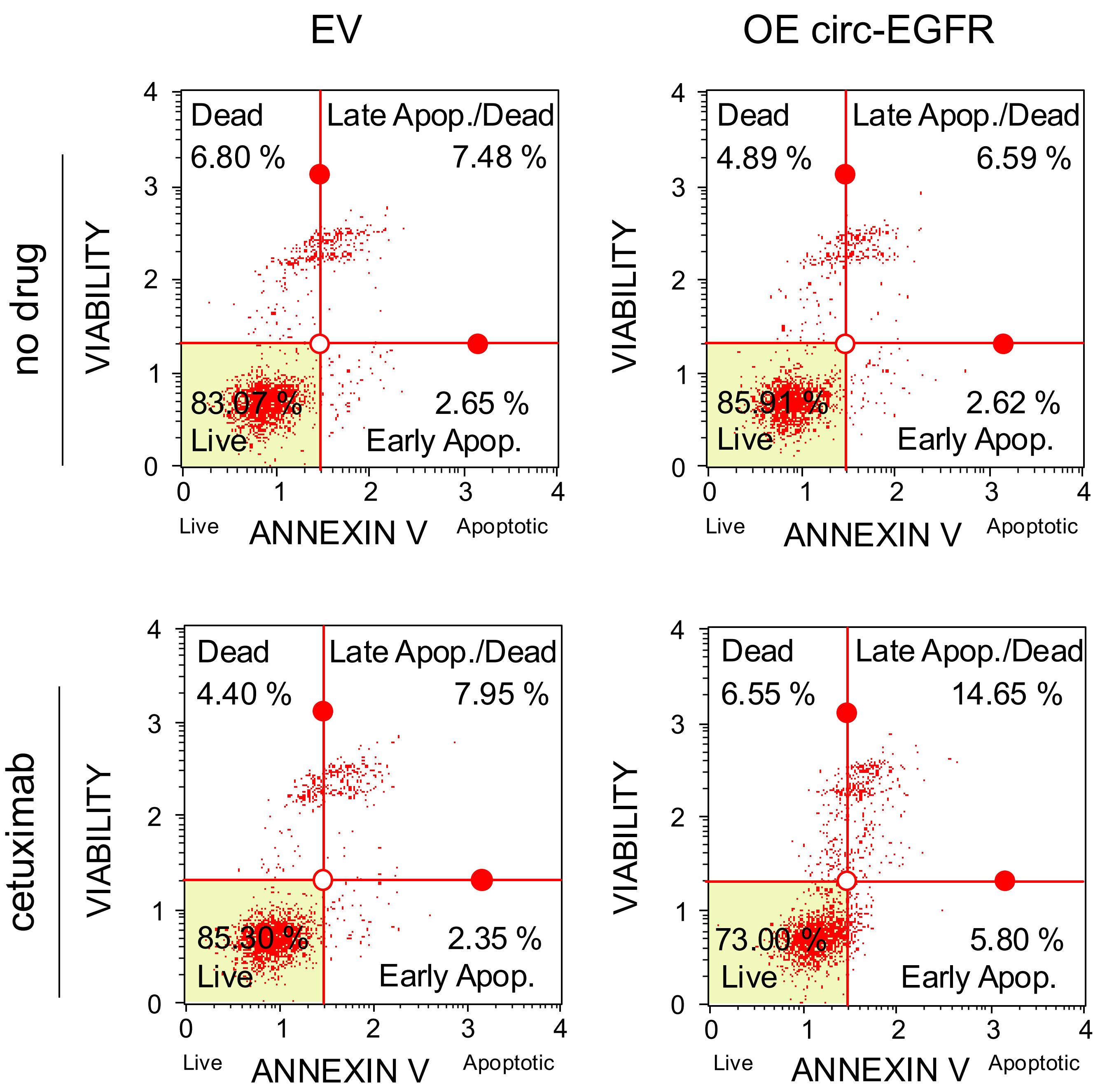

SNU-C1 apoptosis assay

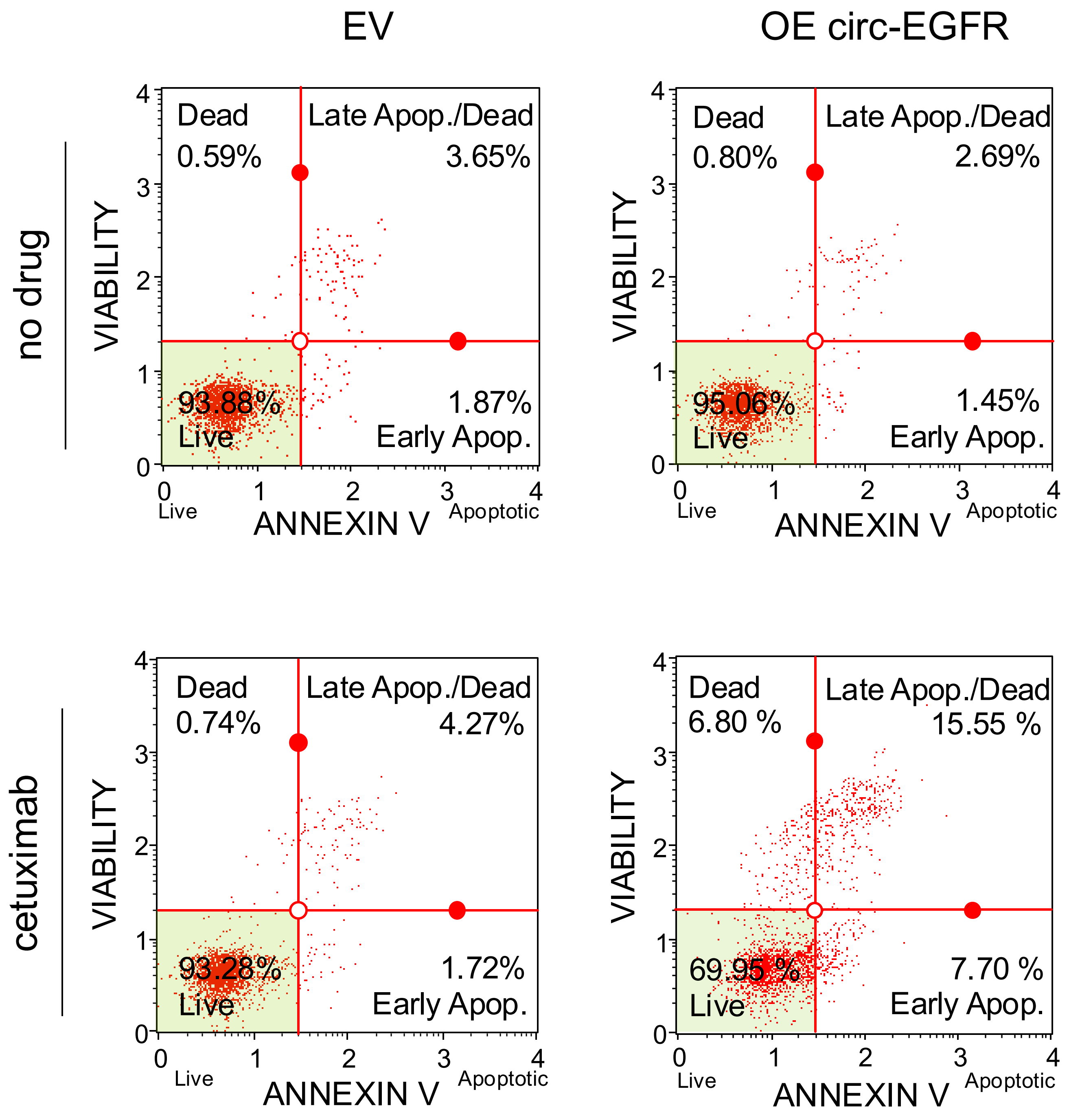

SNU-C1 apoptosis assay (Duplicate Experiment)

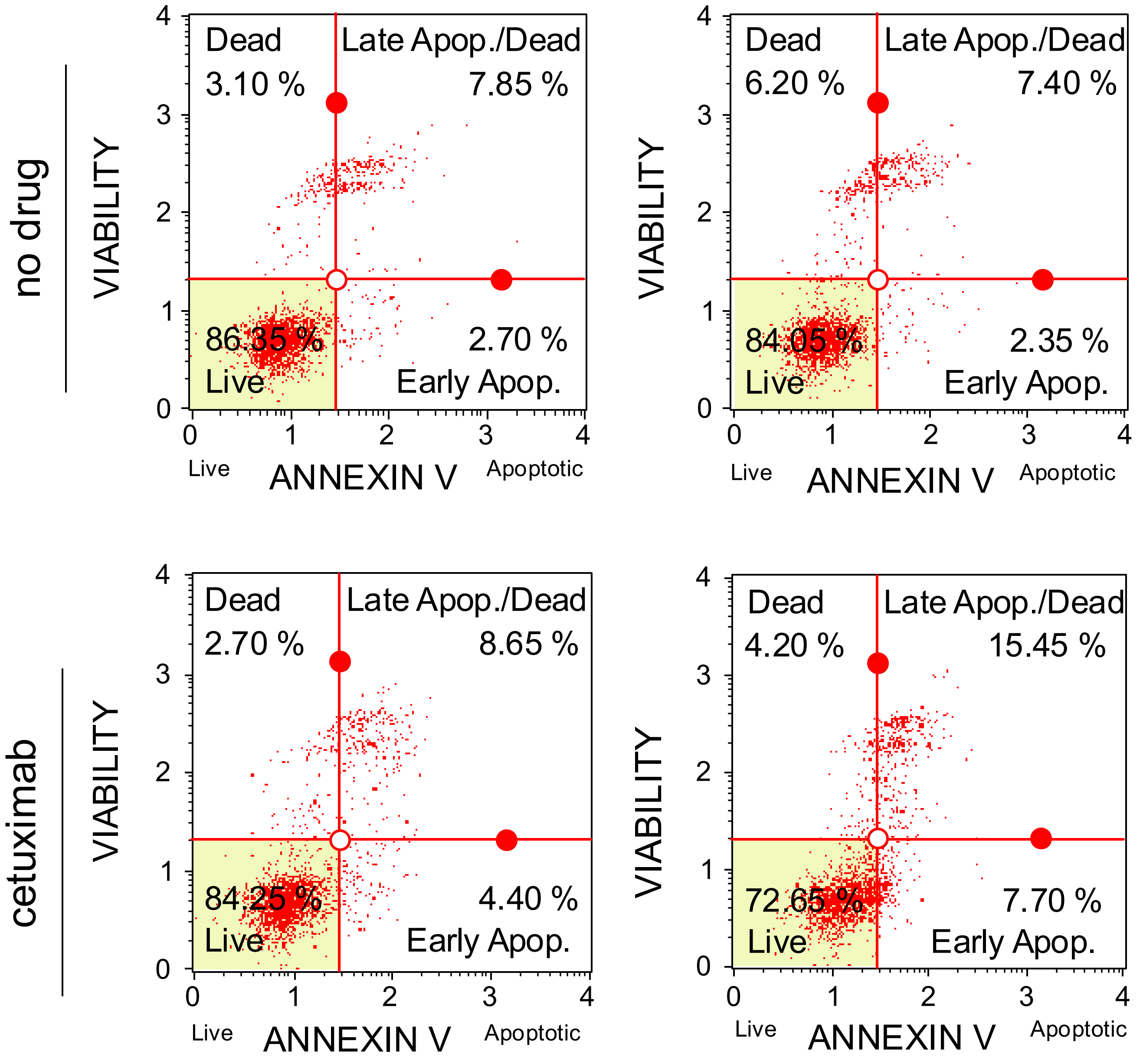

Supplement: Supplementary file 7 — Source data Fig. 2 [file 44321_2025_333_MOESM7_ESM.zip › Figure 2/2D/Figure 2D_Apoptosis.pdf]
