# Supplementary material for: circ-EGFR is a predictor of response to Cetuximab and a potential target in colorectal cancer
Source: EMBO Mol Med. 2025 Nov 10;17(12):3525–54. doi: 10.1038/s44321-025-00333-0 (PMC12686431; doi:10.1038/s44321-025-00333-0)
Supplement: Supplementary file 7 — Source data Fig. 2 [file 44321_2025_333_MOESM7_ESM.zip › Figure 2/2D/Figure 2D_Apoptosis.pptx]

## Slide 1
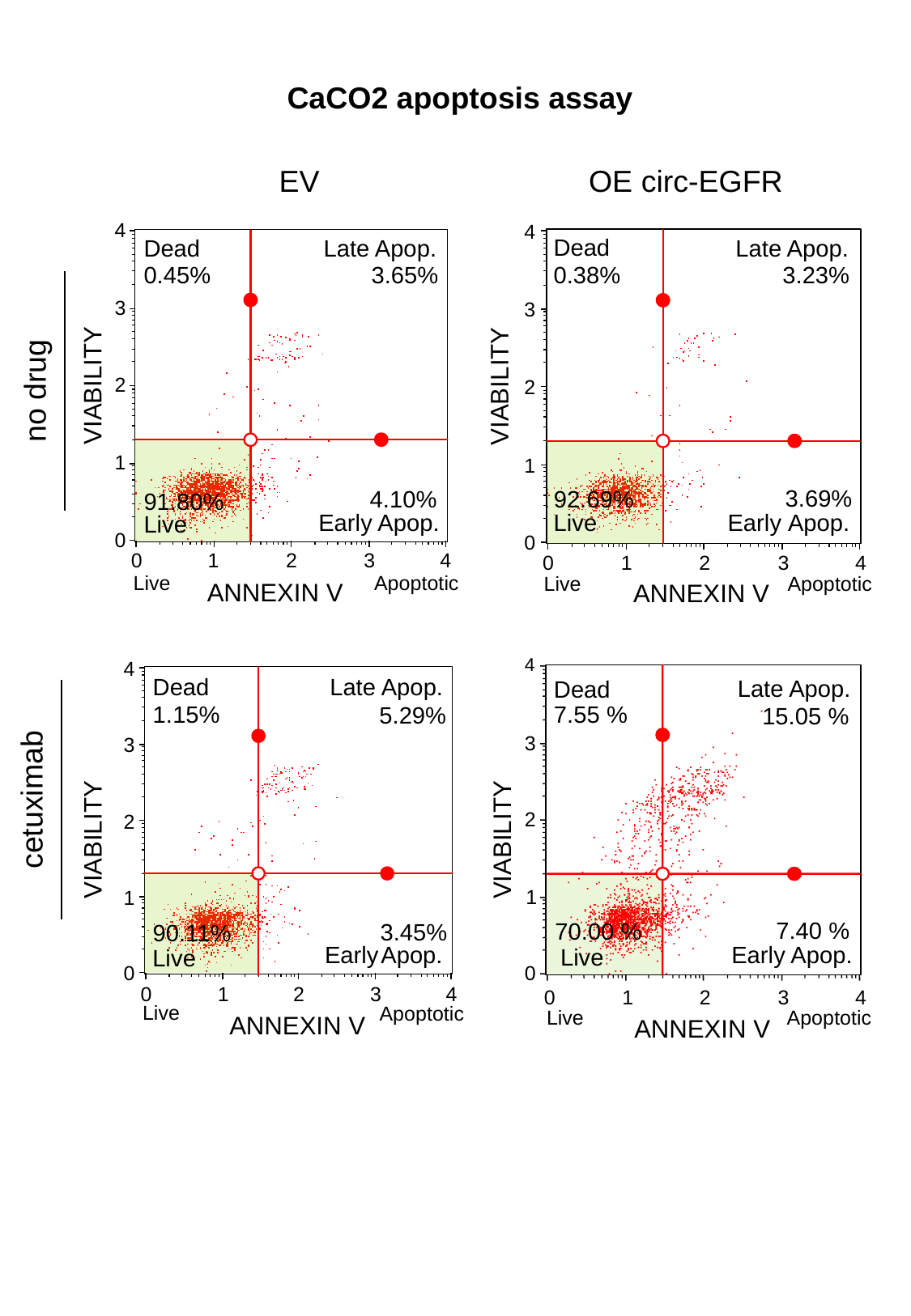

CaCO2 apoptosis assay
EV
OE circ-EGFR
Dead
0.38%
Late Apop.
3.23%
0
1
2
3
4
Live
Apoptotic
Late Apop.
3.65%
4
3
VIABILITY
2
1
0
Dead
0.45%
4.10%
Early Apop.
91.80%
Live
0
1
2
3
4
ANNEXIN V
Live
Apoptotic
4
3
VIABILITY
2
1
0
no drug
3.69%
Early
Apop.
92.69%
Live
ANNEXIN V
Dead
1.15%
0
1
2
3
4
Live
4
3
2
VIABILITY
1
0
ANNEXIN V
4
3
2
1
0
0
1
2
3
4
Apoptotic
Live
ANNEXIN V
Late Apop.
Dead
7.55 %
15.05 %
7.40 %
70.00 %
Early Apop.
Live
Late Apop.
5.29%
cetuximab
VIABILITY
3.45%
Early
Apop.
90.11%
Live
Apoptotic

## Slide 2
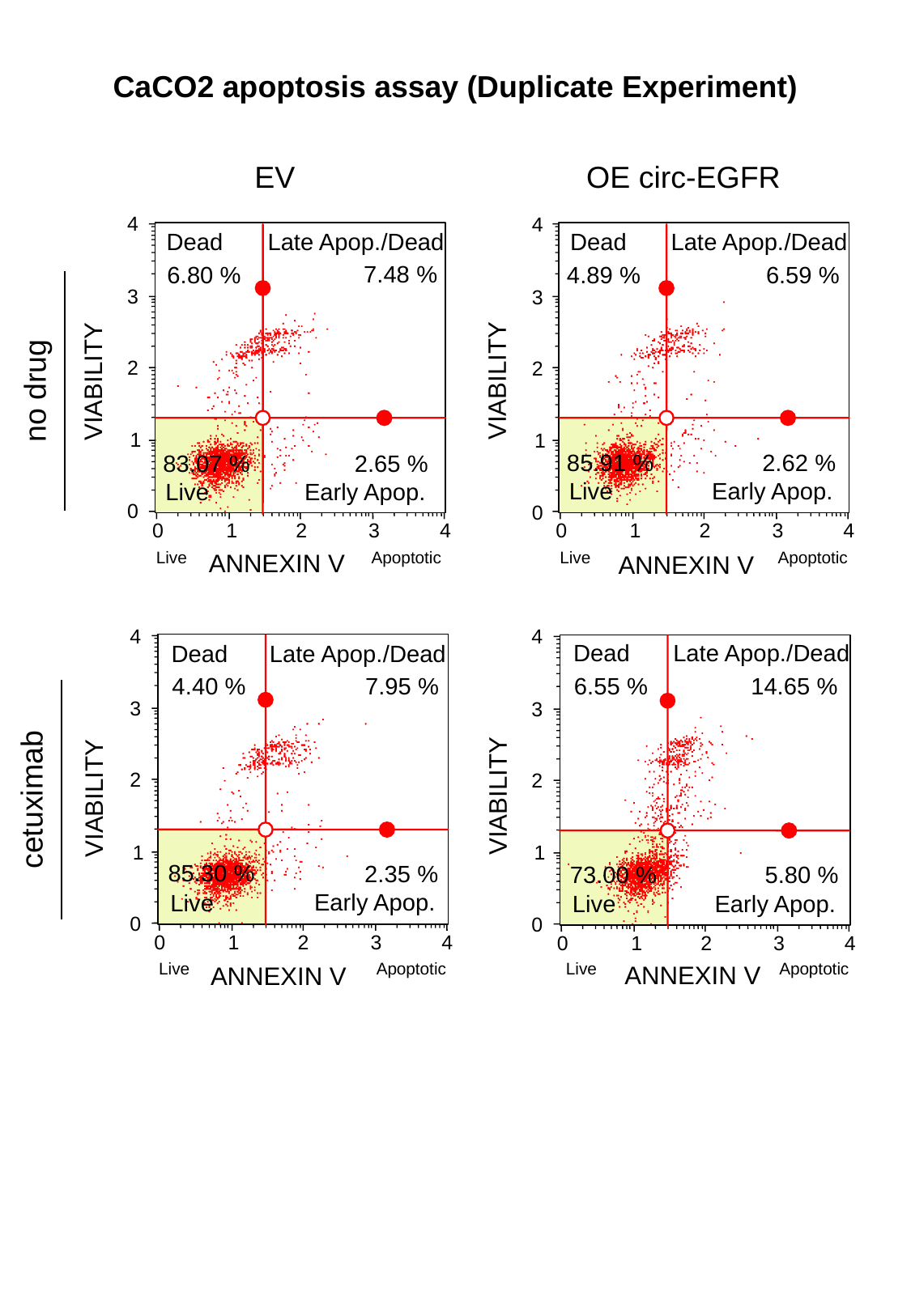

CaCO2 apoptosis assay (Duplicate Experiment)
EV
OE circ-EGFR
4
3
2
VIABILITY
1
0
0
1
2
3
4
Live
Apoptotic
ANNEXIN V
Dead
Late Apop./Dead
7.48 %
6.80 %
83.07 %
2.65 %
Live
Early Apop.
4
3
2
VIABILITY
1
0
0
1
2
3
4
Live
Apoptotic
ANNEXIN V
Dead
Late Apop./Dead
4.89 %
6.59 %
85.91 %
2.62 %
Live
Early Apop.
no drug
4
3
2
VIABILITY
1
0
0
1
2
3
4
Live
Apoptotic
ANNEXIN V
Dead
Late Apop./Dead
4.40 %
7.95 %
85.30 %
2.35 %
Early Apop.
Live
4
3
2
VIABILITY
1
0
0
1
2
3
4
Live
Apoptotic
ANNEXIN V
Dead
Late Apop./Dead
6.55 %
14.65 %
73.00 %
5.80 %
Live
Early Apop.
cetuximab

## Slide 3
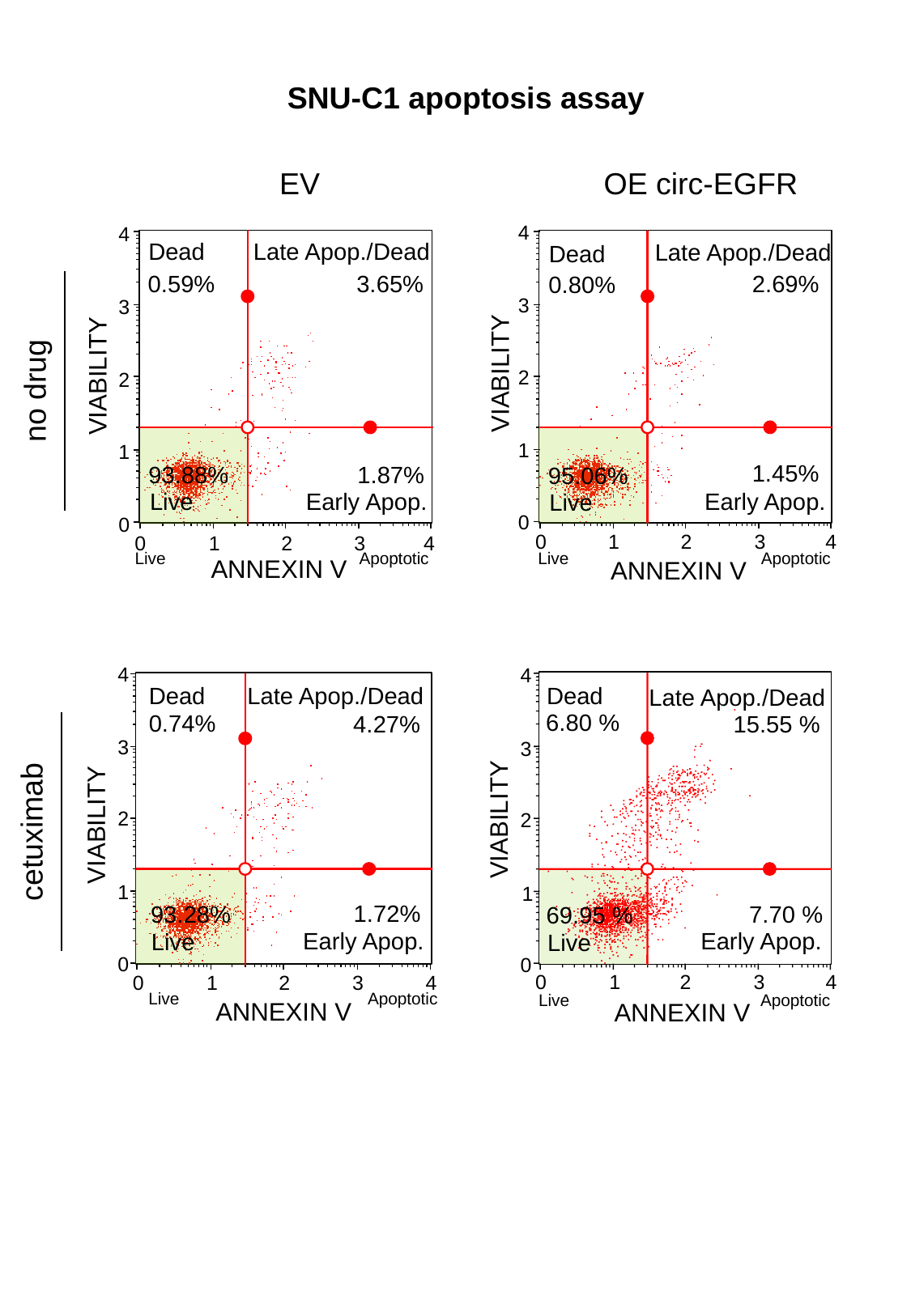

SNU-C1 apoptosis assay
EV
OE circ-EGFR
4
3
VIABILITY
2
1
0
Dead
0.59%
Late Apop./Dead
3.65%
1.87%
Early Apop.
93.88%
Live
0
1
2
3
4
Live
Apoptotic
ANNEXIN V
4
3
VIABILITY
2
1
0
Late Apop./Dead
2.69%
Dead
0.80%
1.45%
Early Apop.
95.06%
Live
0
1
2
3
4
Live
Apoptotic
ANNEXIN V
no drug
0
1
2
3
4
4
3
2
VIABILITY
1
0
Dead
0.74%
Late Apop./Dead
4.27%
1.72%
Early Apop.
93.28%
Live
Live
Apoptotic
ANNEXIN V
4
3
VIABILITY
2
1
0
0
1
2
3
4
Live
Apoptotic
ANNEXIN V
Dead
Late Apop./Dead
6.80 %
15.55 %
7.70 %
69.95 %
Early Apop.
Live
cetuximab

## Slide 4
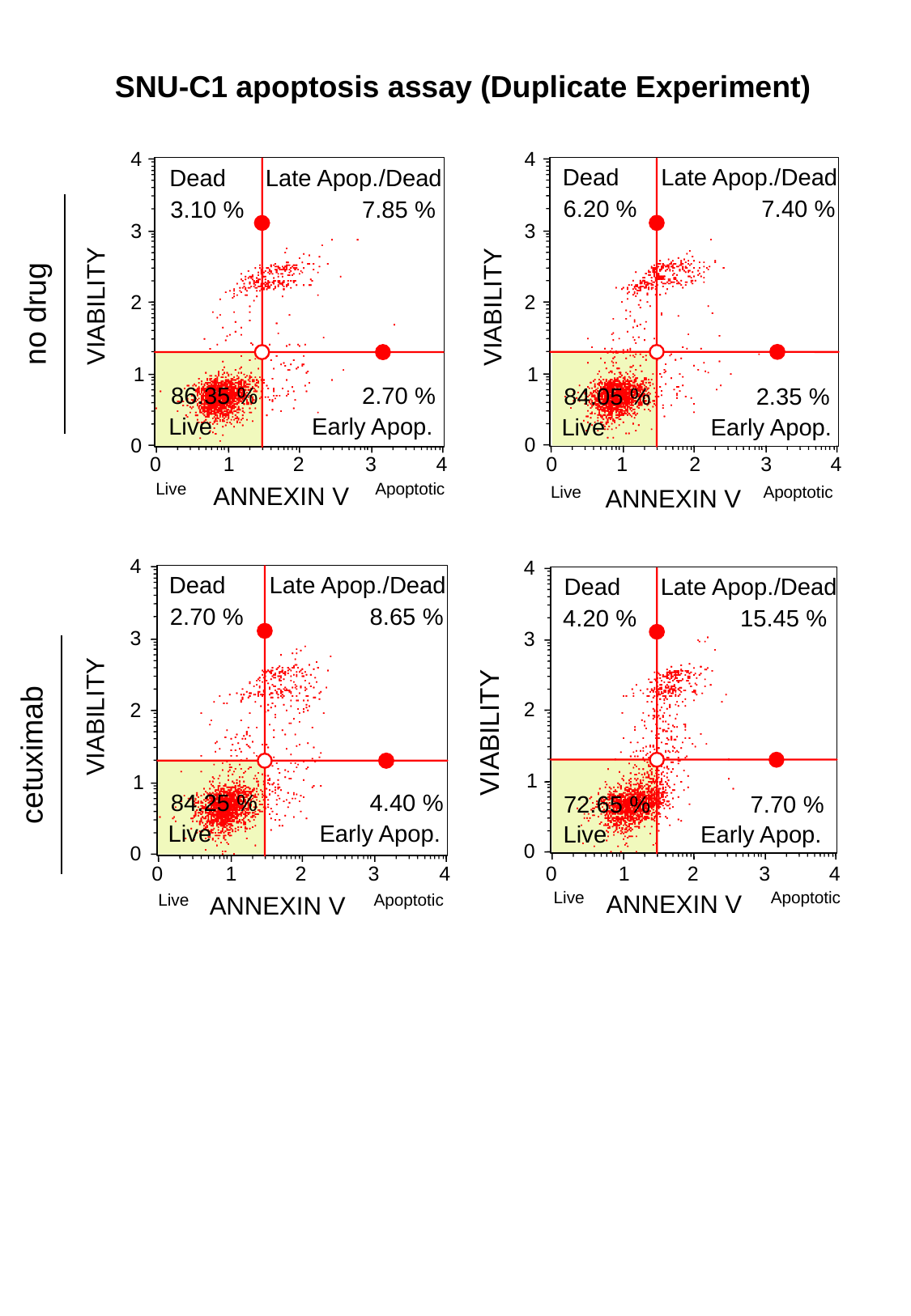

SNU-C1 apoptosis assay (Duplicate Experiment)
4
3
2
VIABILITY
1
0
0
1
2
3
4
Live
Apoptotic
ANNEXIN V
Dead
Late Apop./Dead
3.10 %
7.85 %
86.35 %
2.70 %
Live
Early Apop.
4
3
2
VIABILITY
1
0
0
1
2
3
4
Live
Apoptotic
ANNEXIN V
Dead
Late Apop./Dead
6.20 %
7.40 %
84.05 %
2.35 %
Live
Early Apop.
no drug
4
3
2
VIABILITY
1
0
0
1
2
3
4
Live
Apoptotic
ANNEXIN V
Dead
Late Apop./Dead
2.70 %
8.65 %
84.25 %
4.40 %
Live
Early Apop.
4
3
2
VIABILITY
1
0
0
1
2
3
4
Live
Apoptotic
ANNEXIN V
Dead
Late Apop./Dead
4.20 %
15.45 %
72.65 %
7.70 %
Live
Early Apop.
cetuximab
