# Supplementary material for: circ-EGFR is a predictor of response to Cetuximab and a potential target in colorectal cancer
Source: EMBO Mol Med. 2025 Nov 10;17(12):3525–54. doi: 10.1038/s44321-025-00333-0 (PMC12686431; doi:10.1038/s44321-025-00333-0)
Supplement: Supplementary file 10 — Source data Fig. 5 [file 44321_2025_333_MOESM10_ESM.zip › Figure 5/5D/Figure 5D_WB.pdf]

CaCO2

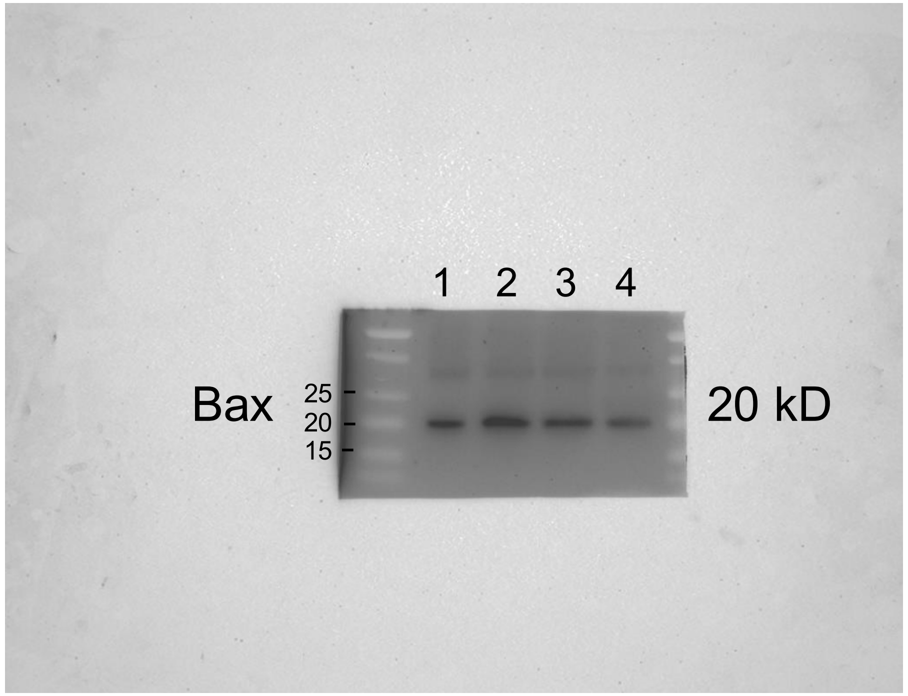

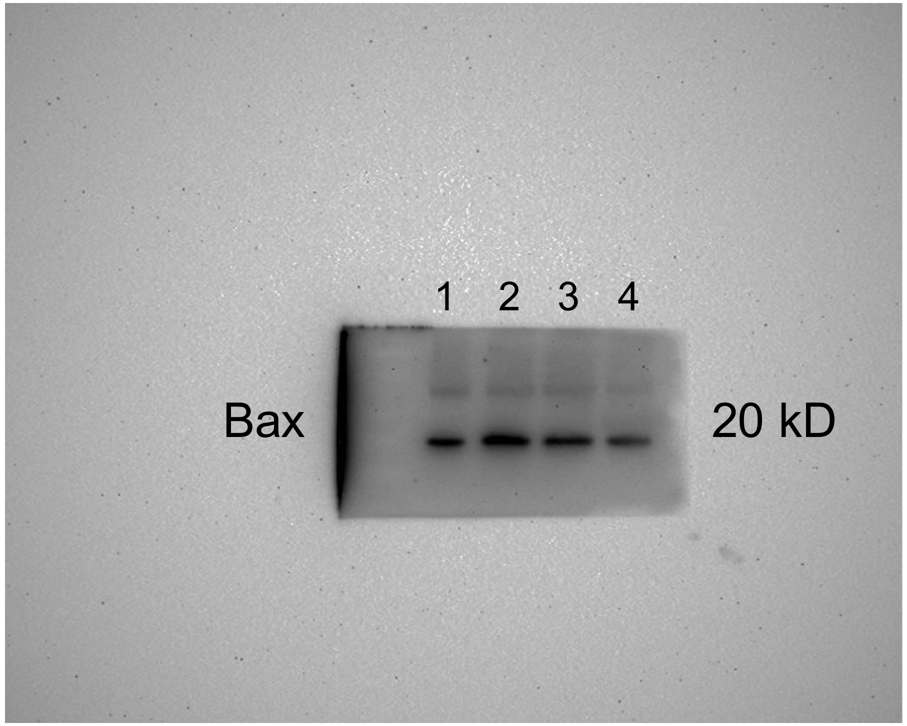

1. circc-EV
2. circ-OE
3. circ-OE + miR-NC
4. circ-OE + miR-OE

SNU-C1

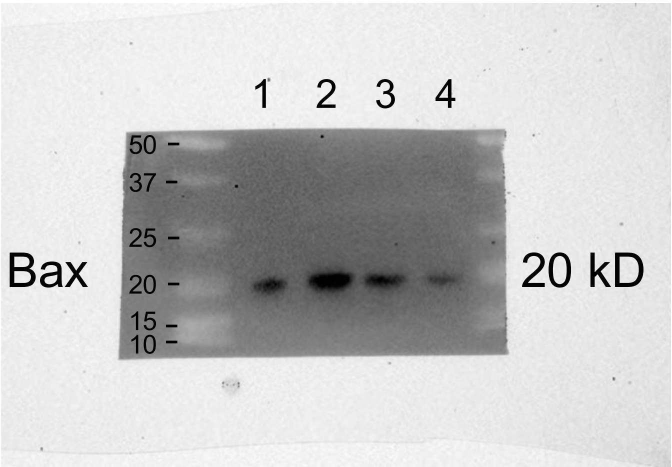

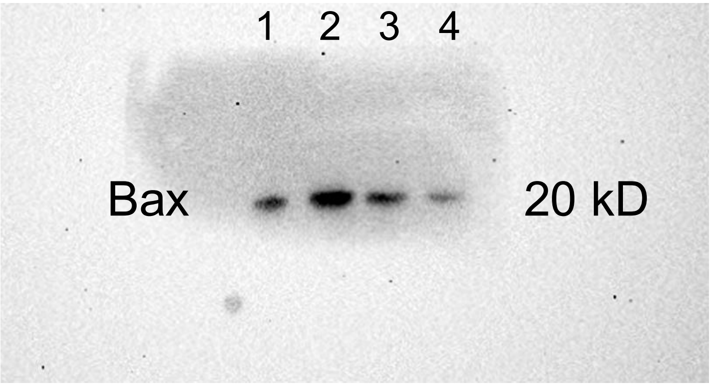

1. circc-EV
2. circ-OE
3. circ-OE + miR-NC
4. circ-OE + miR-OE

CaCO2

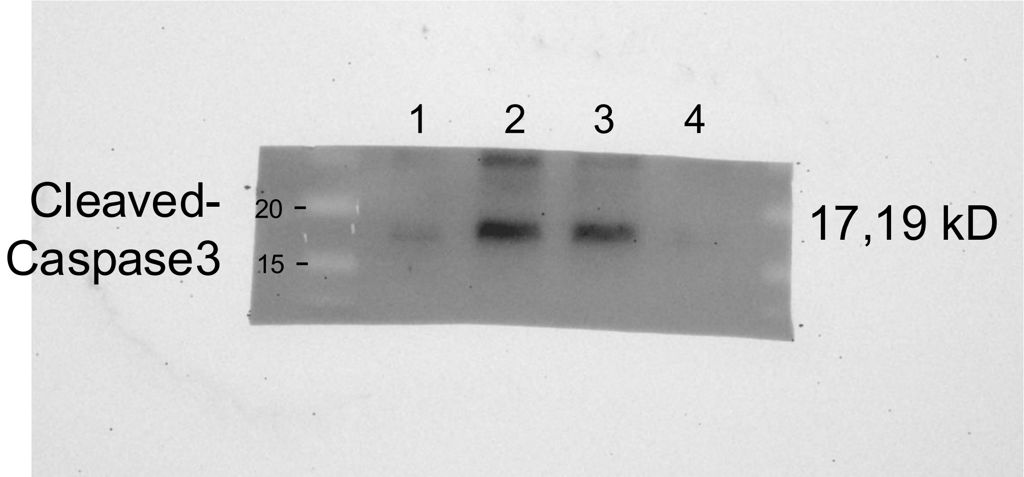

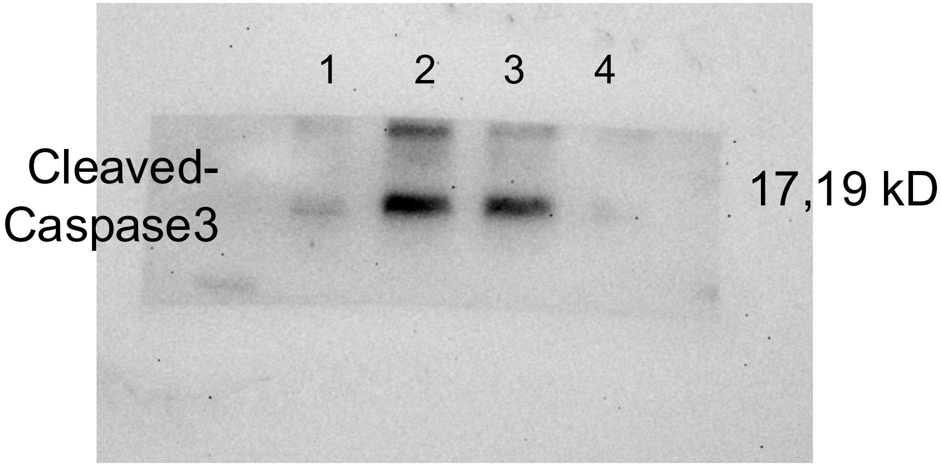

1. circc-EV
2. circ-OE
3. circ-OE + miR-NC
4. circ-OE + miR-OE

SNU-C1

1 2 3 4

Cleaved-
Caspase3

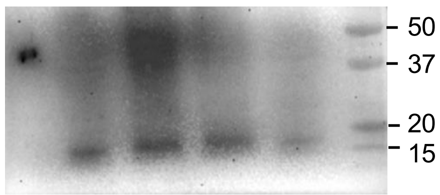

17,19 kD

1 2 3 4

Cleaved-
Caspase3

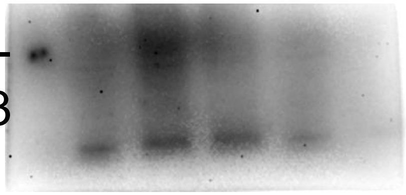

17,19 kD

1. circc-EV
2. circ-OE
3. circ-OE + miR-NC
4. circ-OE + miR-OE

CaCO2

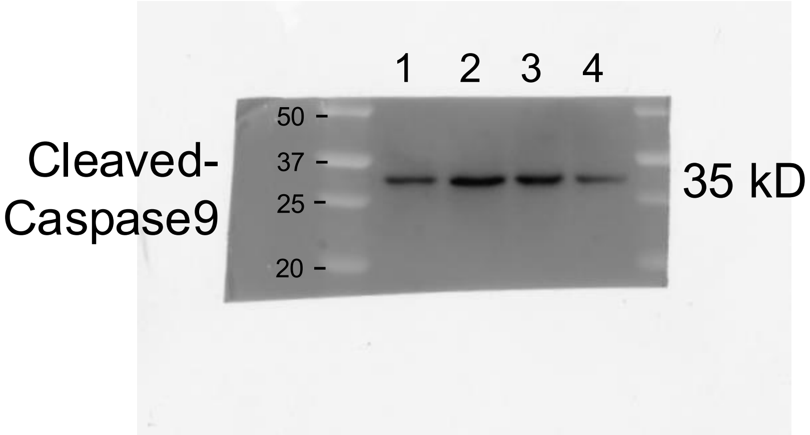

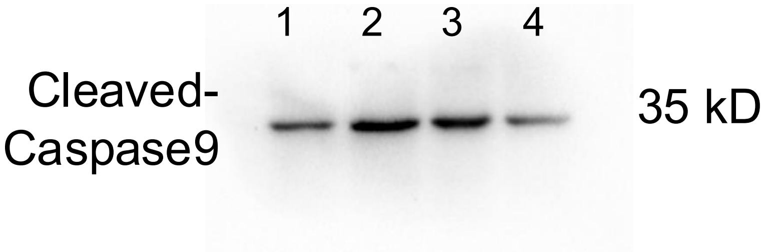

1. circc-EV
2. circ-OE
3. circ-OE + miR-NC
4. circ-OE + miR-OE

SNU-C1

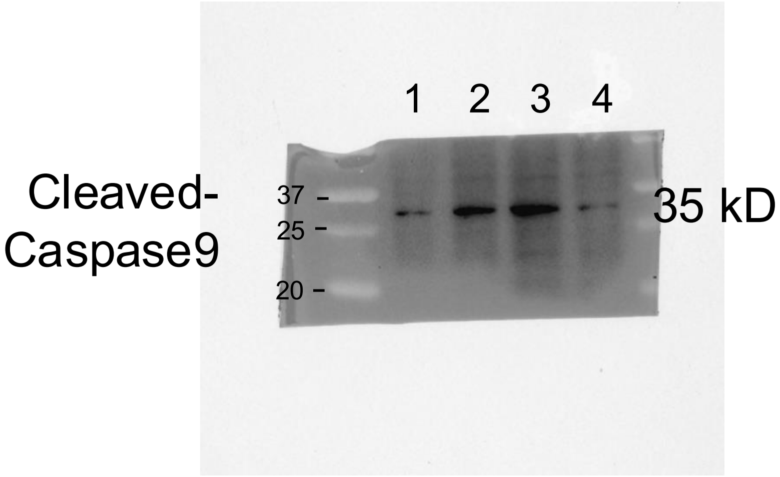

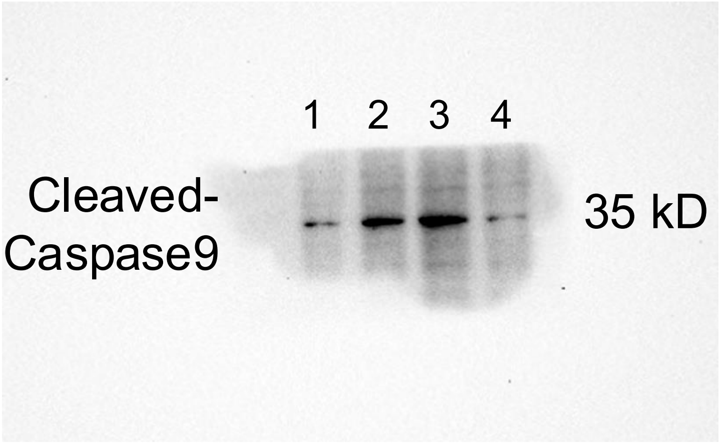

1. circc-EV
2. circ-OE
3. circ-OE + miR-NC
4. circ-OE + miR-OE

CaCO2

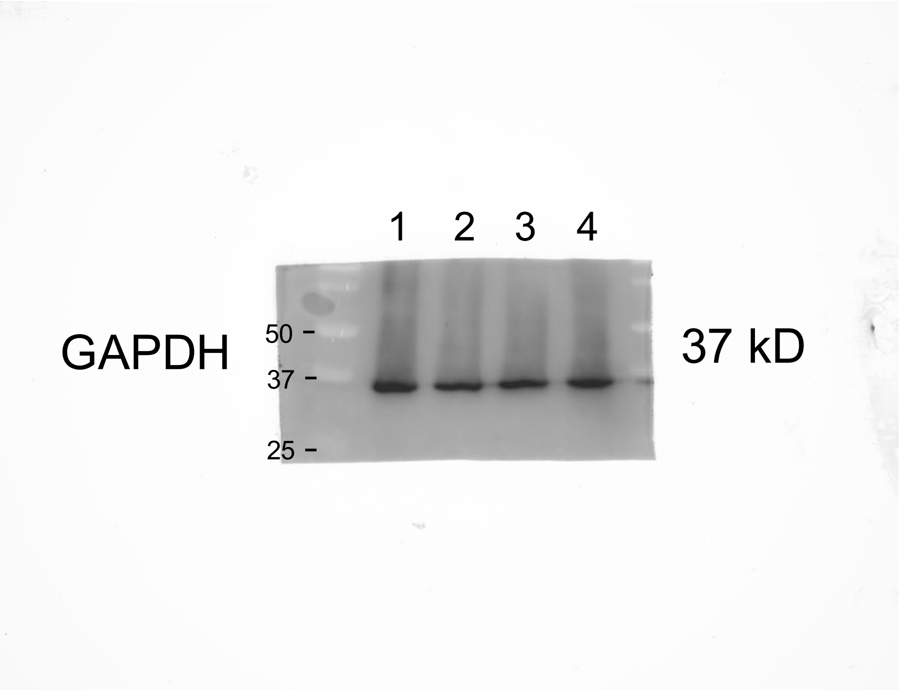

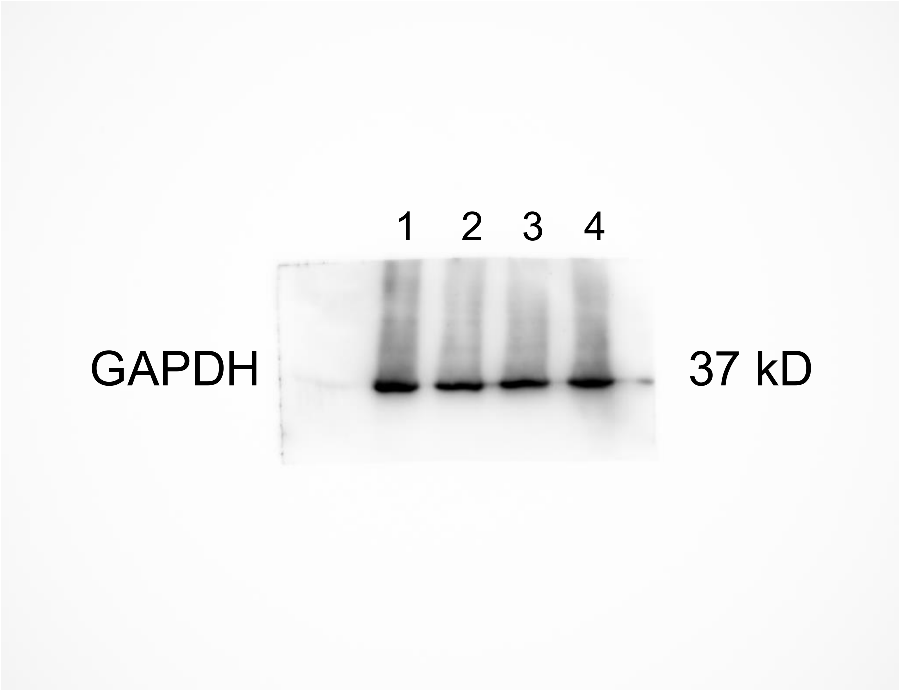

1. circc-EV
2. circ-OE
3. circ-OE + miR-NC
4. circ-OE + miR-OE

SNU-C1

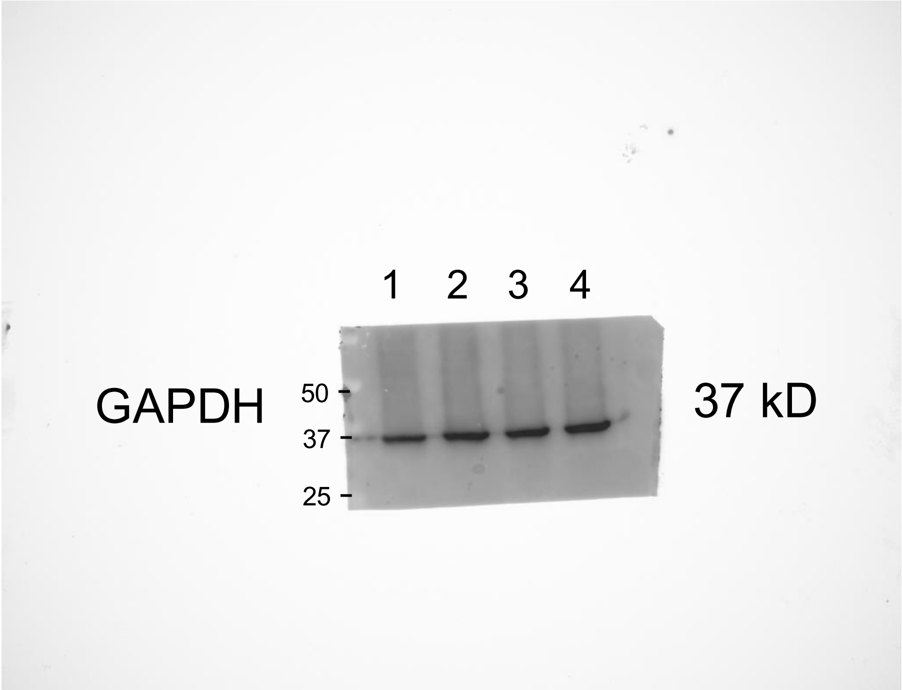

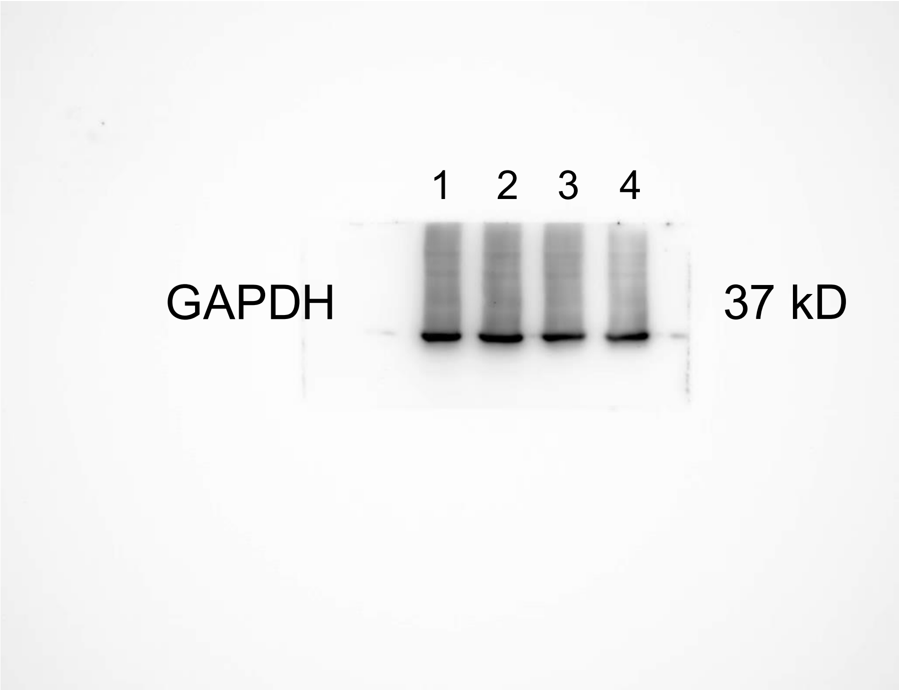

1. circc-EV
2. circ-OE
3. circ-OE + miR-NC
4. circ-OE + miR-OE
