# Supplementary material for: circ-EGFR is a predictor of response to Cetuximab and a potential target in colorectal cancer
Source: EMBO Mol Med. 2025 Nov 10;17(12):3525–54. doi: 10.1038/s44321-025-00333-0 (PMC12686431; doi:10.1038/s44321-025-00333-0)
Supplement: Supplementary file 10 — Source data Fig. 5 [file 44321_2025_333_MOESM10_ESM.zip › Figure 5/5D/Figure 5D_WB.pptx]

## Slide 1
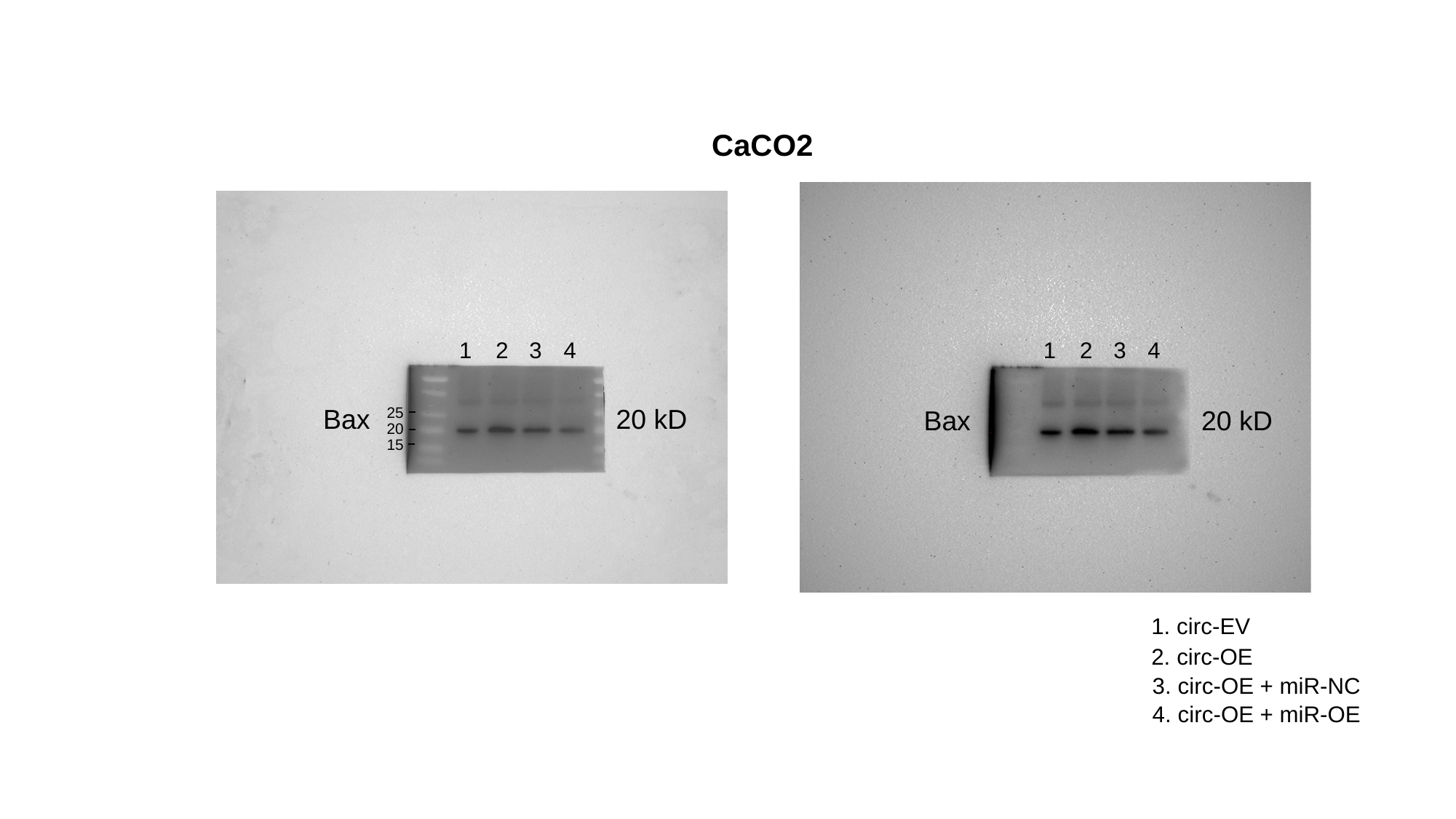

CaCO2
1
2
3
4
1
2
3
4
Bax
20 kD
25
Bax
20 kD
20
15
1. circ-EV
2. circ-OE
3. circ-OE + miR-NC
4. circ-OE + miR-OE

## Slide 2
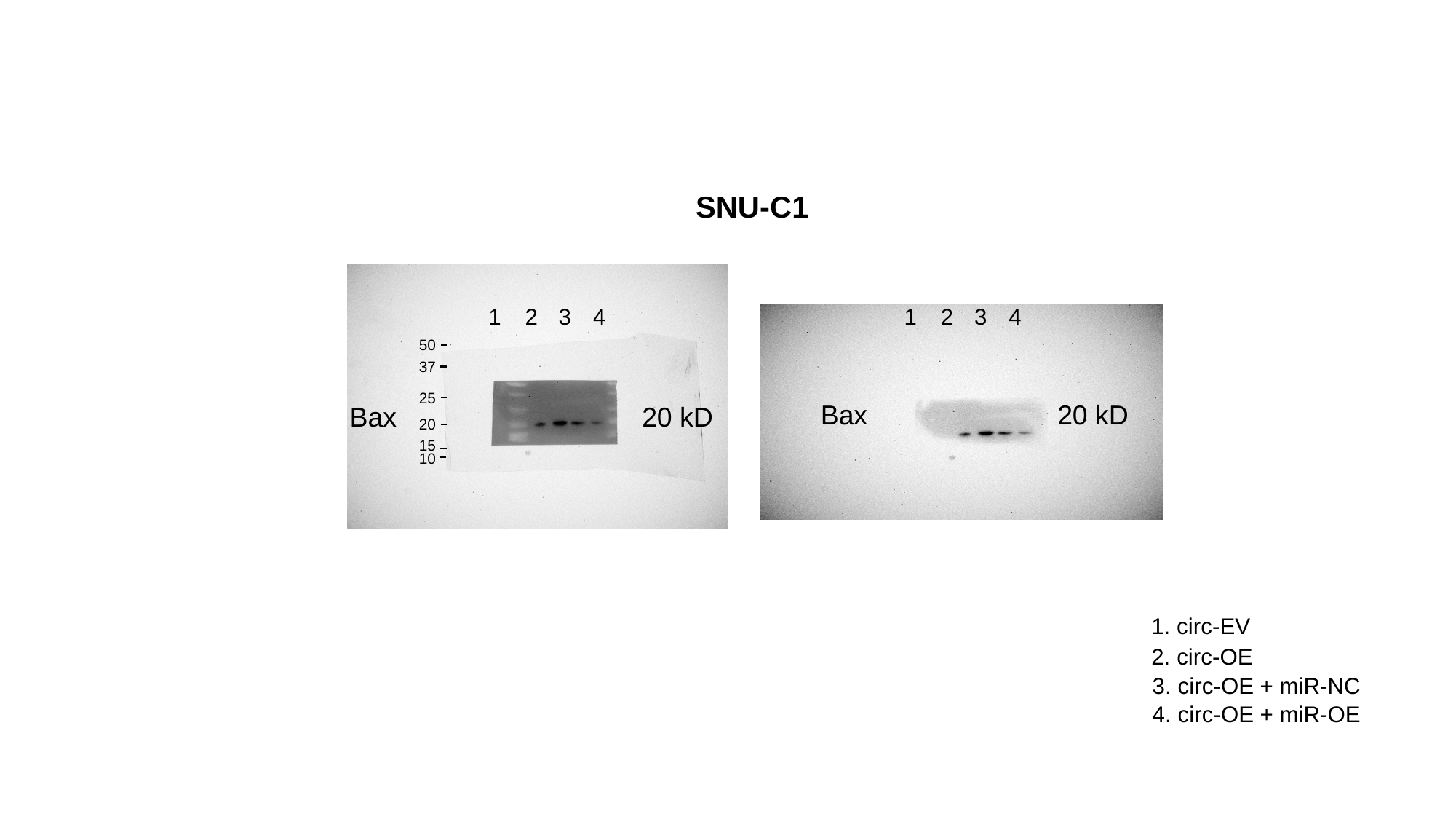

SNU-C1
1
2
3
4
1
2
3
4
50
37
25
Bax
20 kD
Bax
20 kD
20
15
10
1. circ-EV
2. circ-OE
3. circ-OE + miR-NC
4. circ-OE + miR-OE

## Slide 3
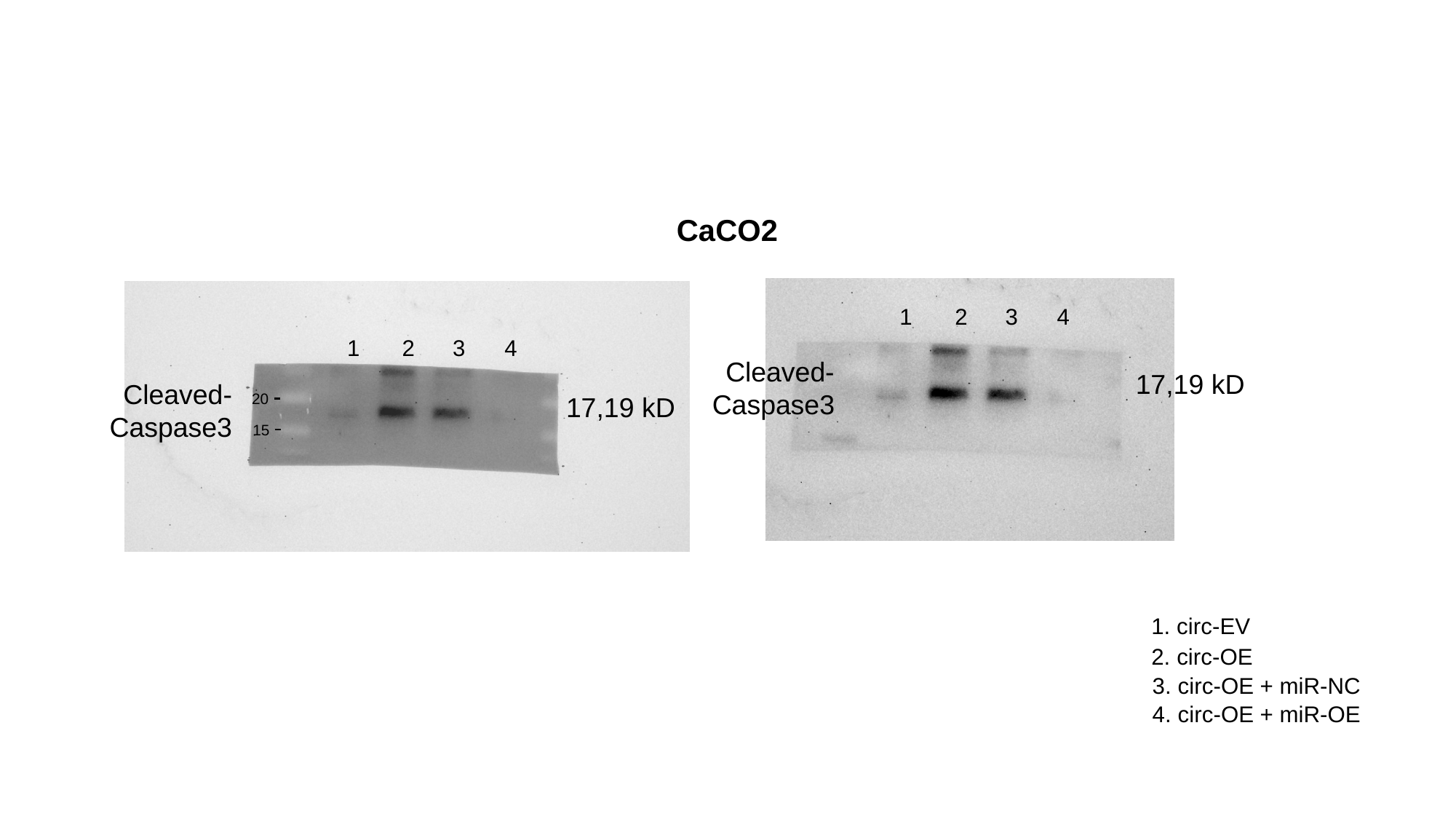

CaCO2
1
2
3
4
1
2
3
4
Cleaved-
Caspase3
17,19 kD
Cleaved-
Caspase3
20
17,19 kD
15
1. circ-EV
2. circ-OE
3. circ-OE + miR-NC
4. circ-OE + miR-OE

## Slide 4
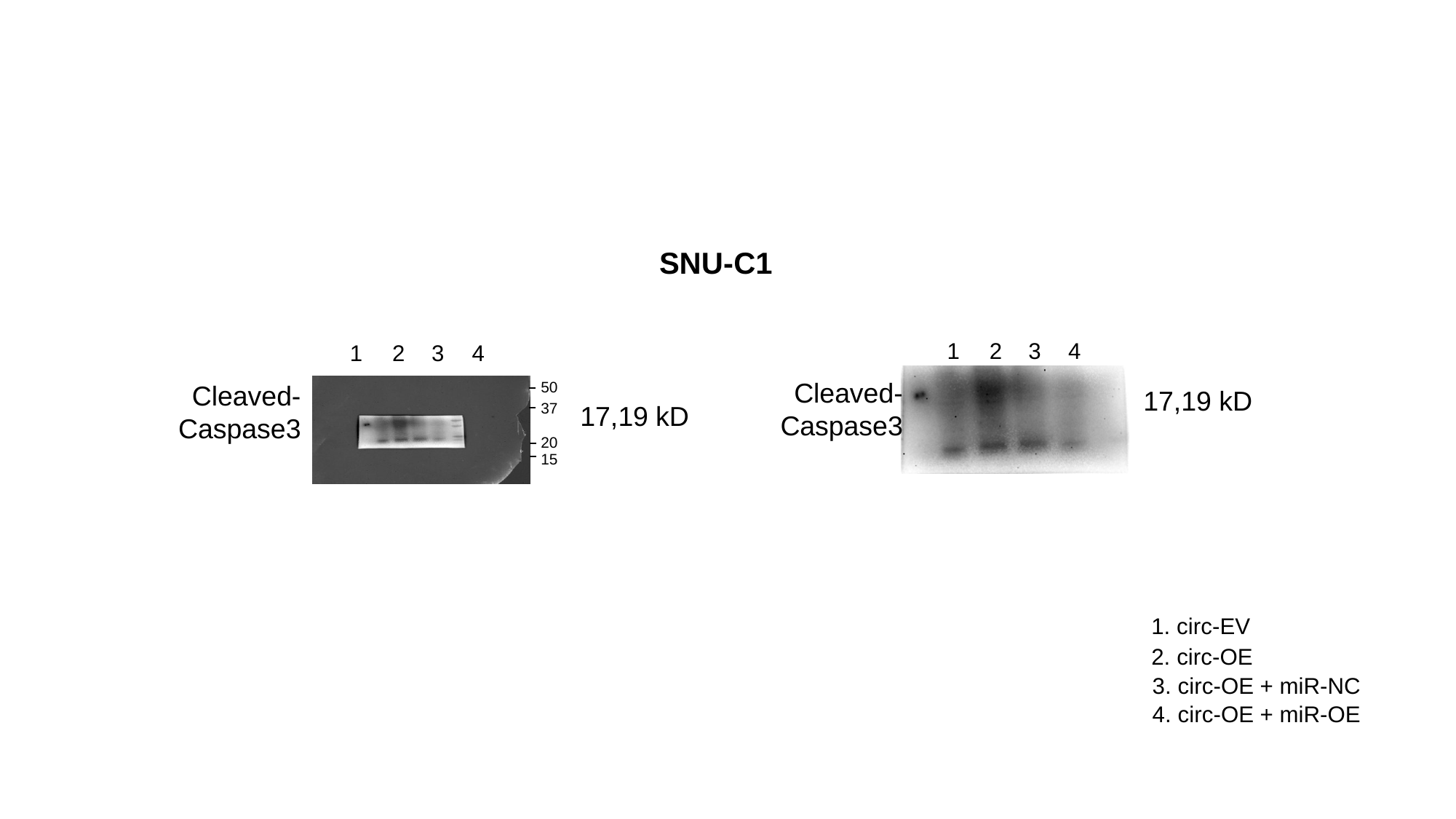

SNU-C1
1
2
3
4
1
2
3
4
Cleaved-
Caspase3
50
Cleaved-
Caspase3
17,19 kD
37
17,19 kD
20
15
1. circ-EV
2. circ-OE
3. circ-OE + miR-NC
4. circ-OE + miR-OE

## Slide 5
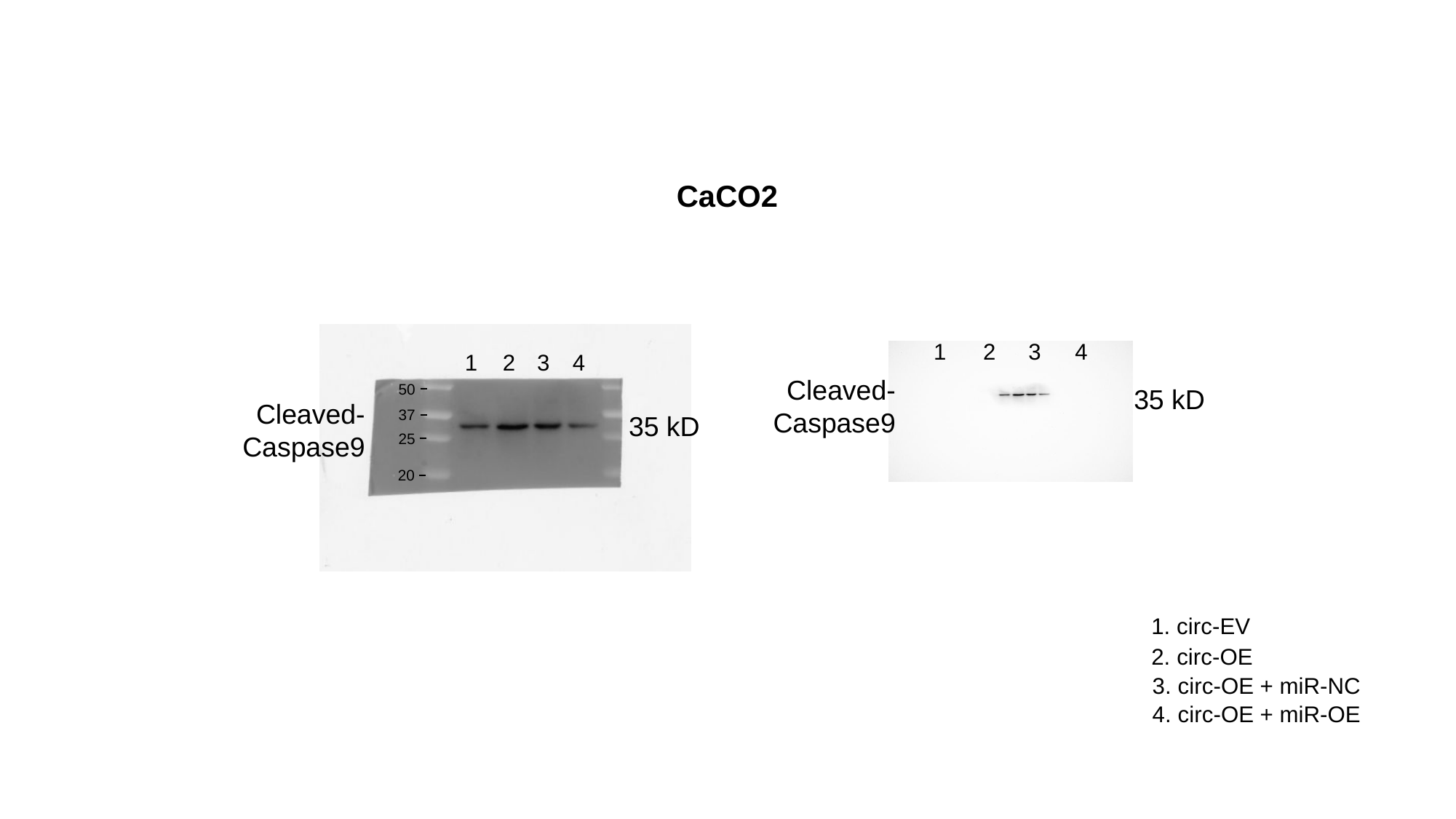

CaCO2
1
2
3
4
1
2
3
4
Cleaved-
Caspase9
50
35 kD
Cleaved-
Caspase9
37
35 kD
25
20
1. circ-EV
2. circ-OE
3. circ-OE + miR-NC
4. circ-OE + miR-OE

## Slide 6
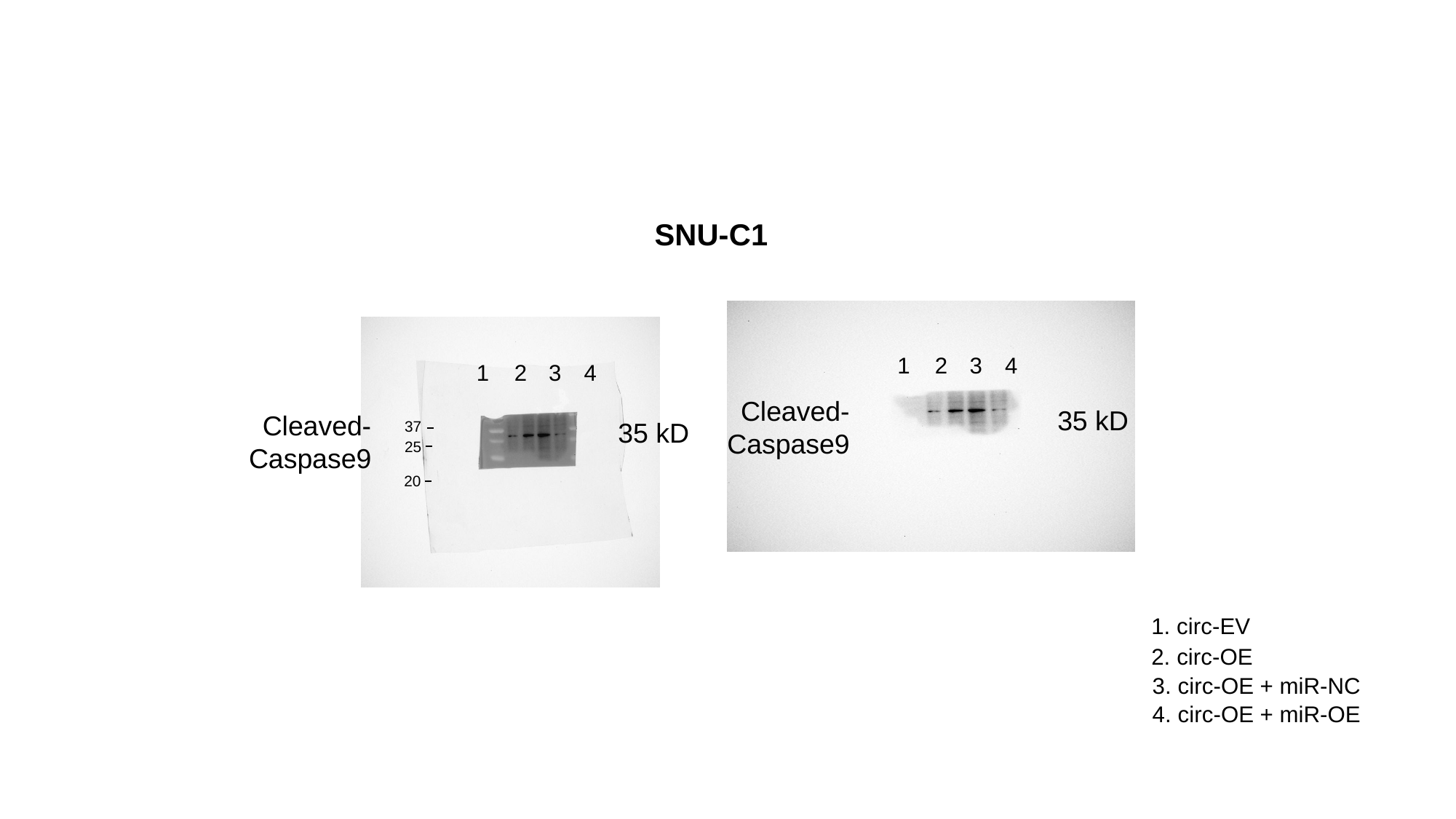

SNU-C1
1
2
3
4
1
2
3
4
Cleaved-
Caspase9
35 kD
Cleaved-
Caspase9
35 kD
37
25
20
1. circ-EV
2. circ-OE
3. circ-OE + miR-NC
4. circ-OE + miR-OE

## Slide 7
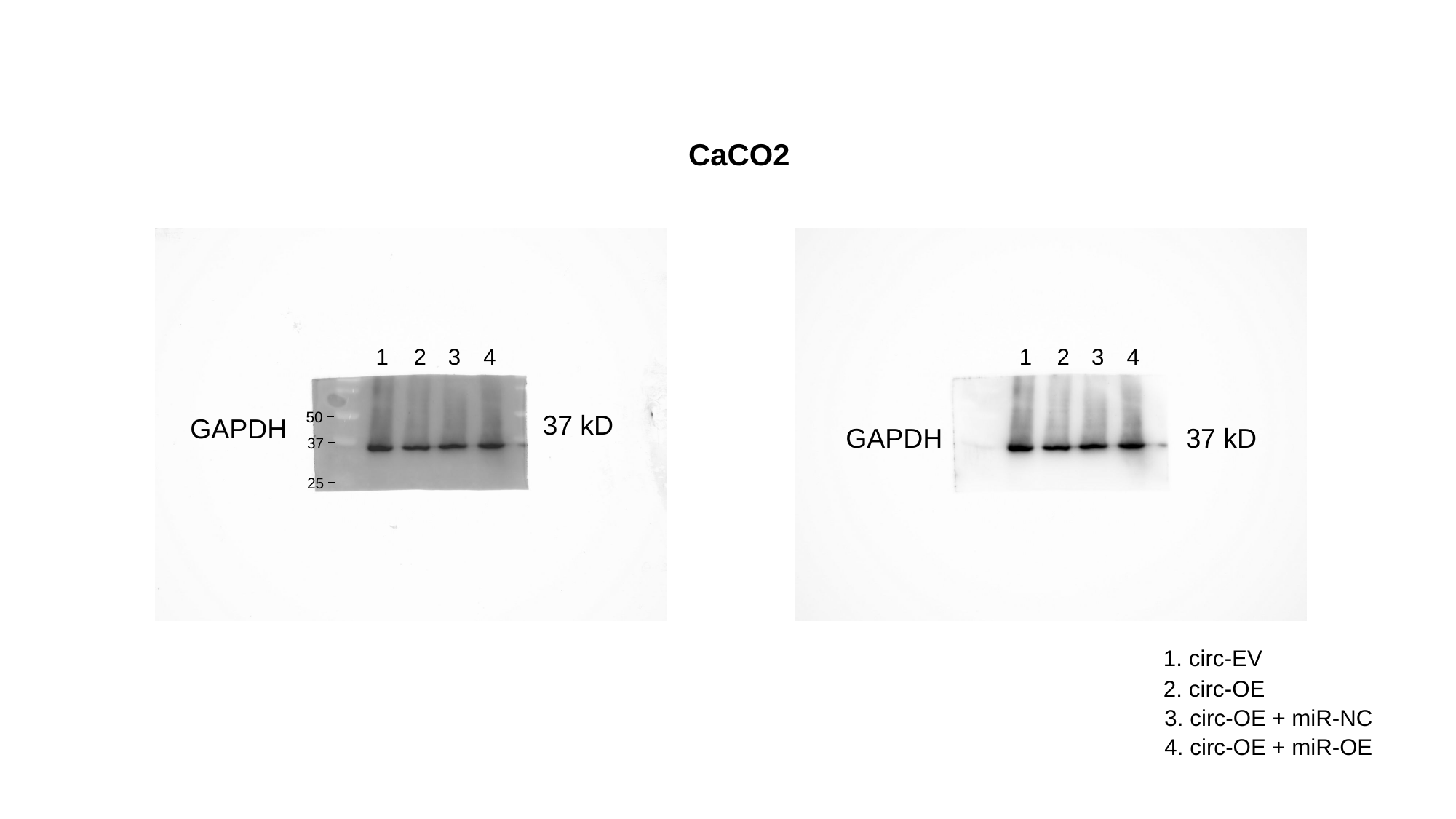

CaCO2
1
2
3
4
1
2
3
4
50
37 kD
GAPDH
GAPDH
37 kD
37
25
1. circ-EV
2. circ-OE
3. circ-OE + miR-NC
4. circ-OE + miR-OE

## Slide 8
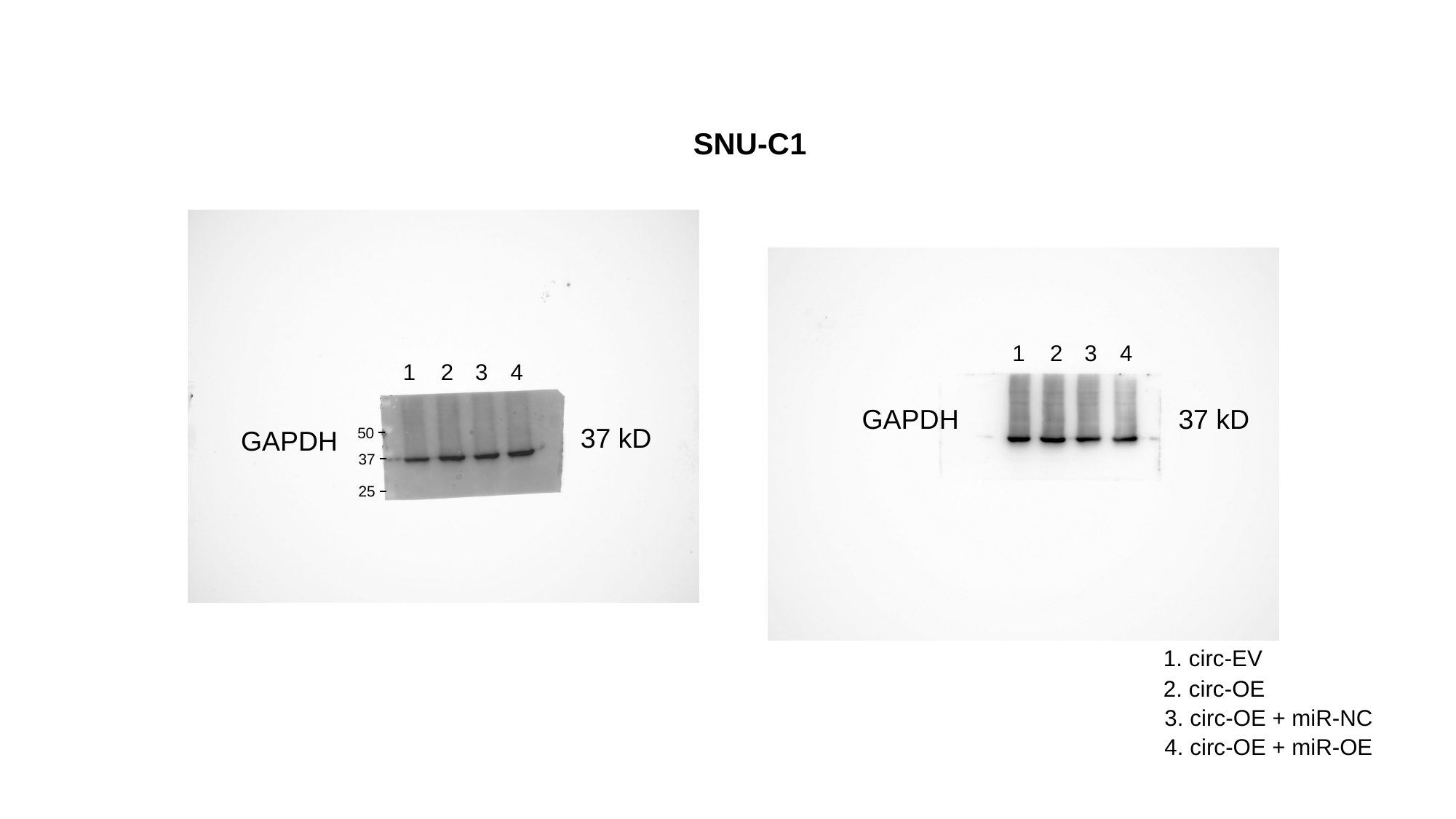

SNU-C1
1
2
3
4
1
2
3
4
37 kD
GAPDH
37 kD
50
GAPDH
37
25
1. circ-EV
2. circ-OE
3. circ-OE + miR-NC
4. circ-OE + miR-OE
