# Supplementary figures and images for: circ-EGFR is a predictor of response to Cetuximab and a potential target in colorectal cancer (part 2 of 2)
Source: EMBO Mol Med. 2025 Nov 10;17(12):3525–54. doi: 10.1038/s44321-025-00333-0 (PMC12686431; doi:10.1038/s44321-025-00333-0)

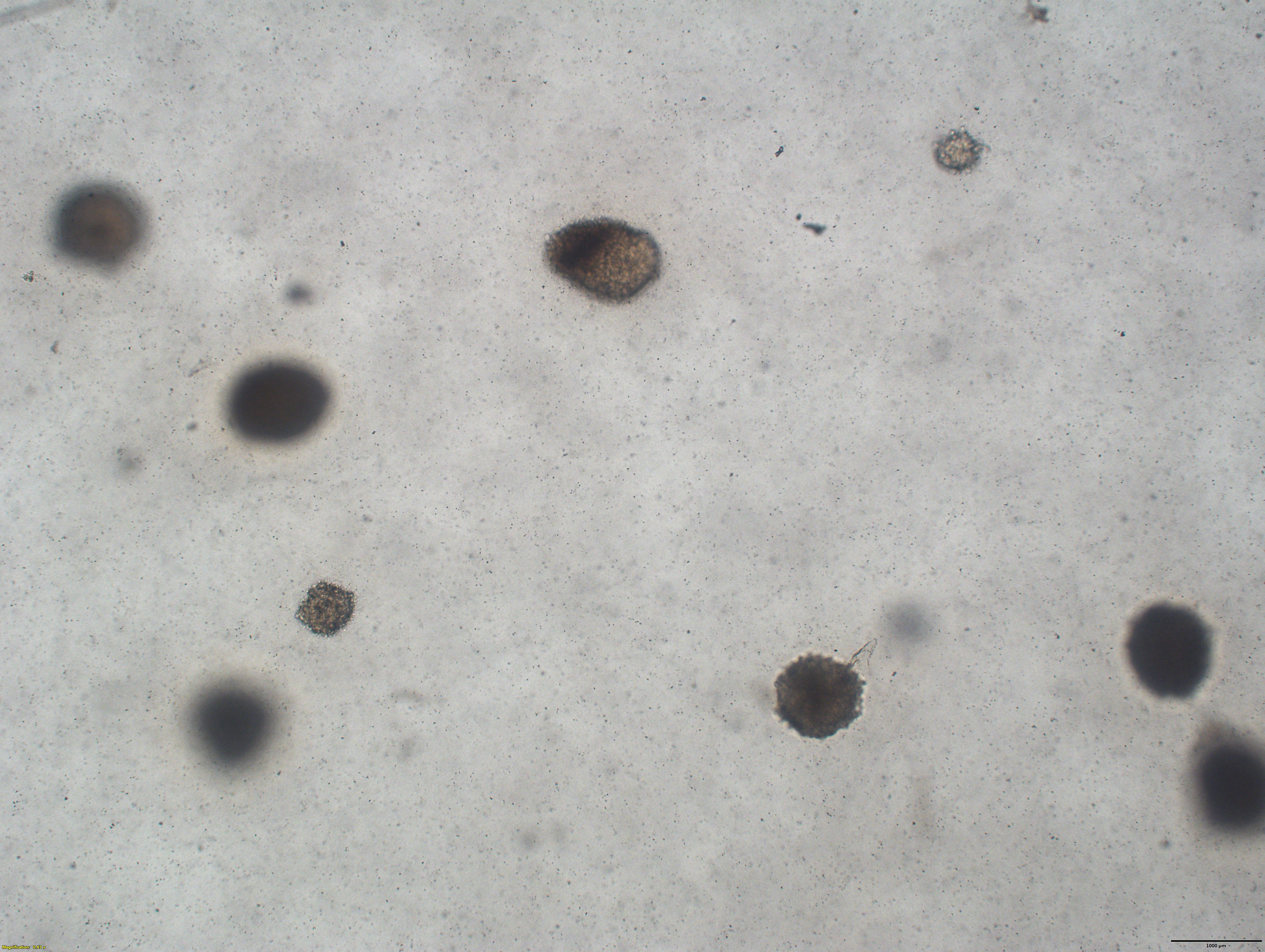

Supplement: Supplementary file 10 — Source data Fig. 5 [file 44321_2025_333_MOESM10_ESM.zip › Figure 5/5C/SNU-C1/Rep 1/4_OE+OE.jpg]

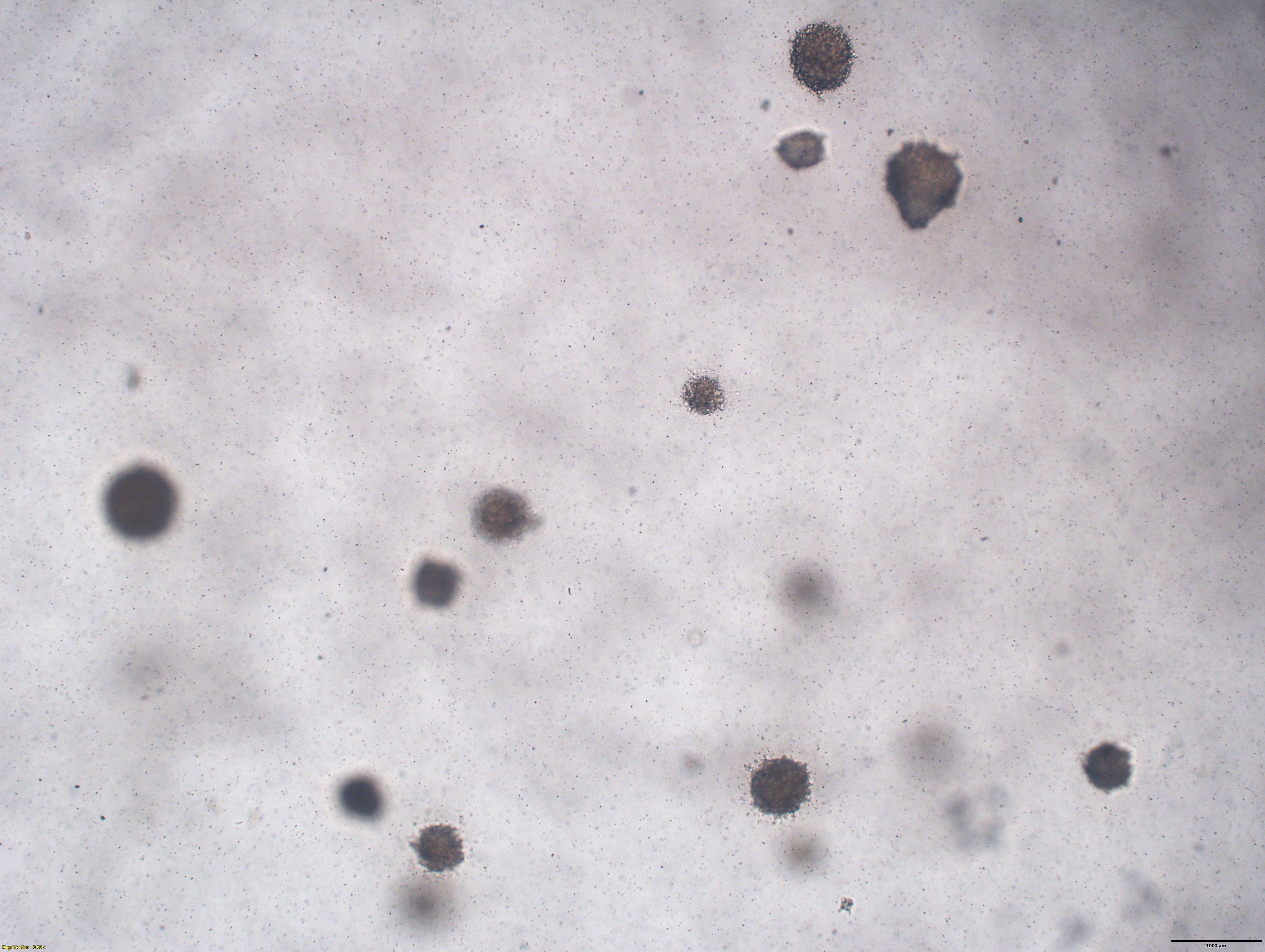

Supplement: Supplementary file 10 — Source data Fig. 5 [file 44321_2025_333_MOESM10_ESM.zip › Figure 5/5C/SNU-C1/Rep 2/1_NC.jpg]

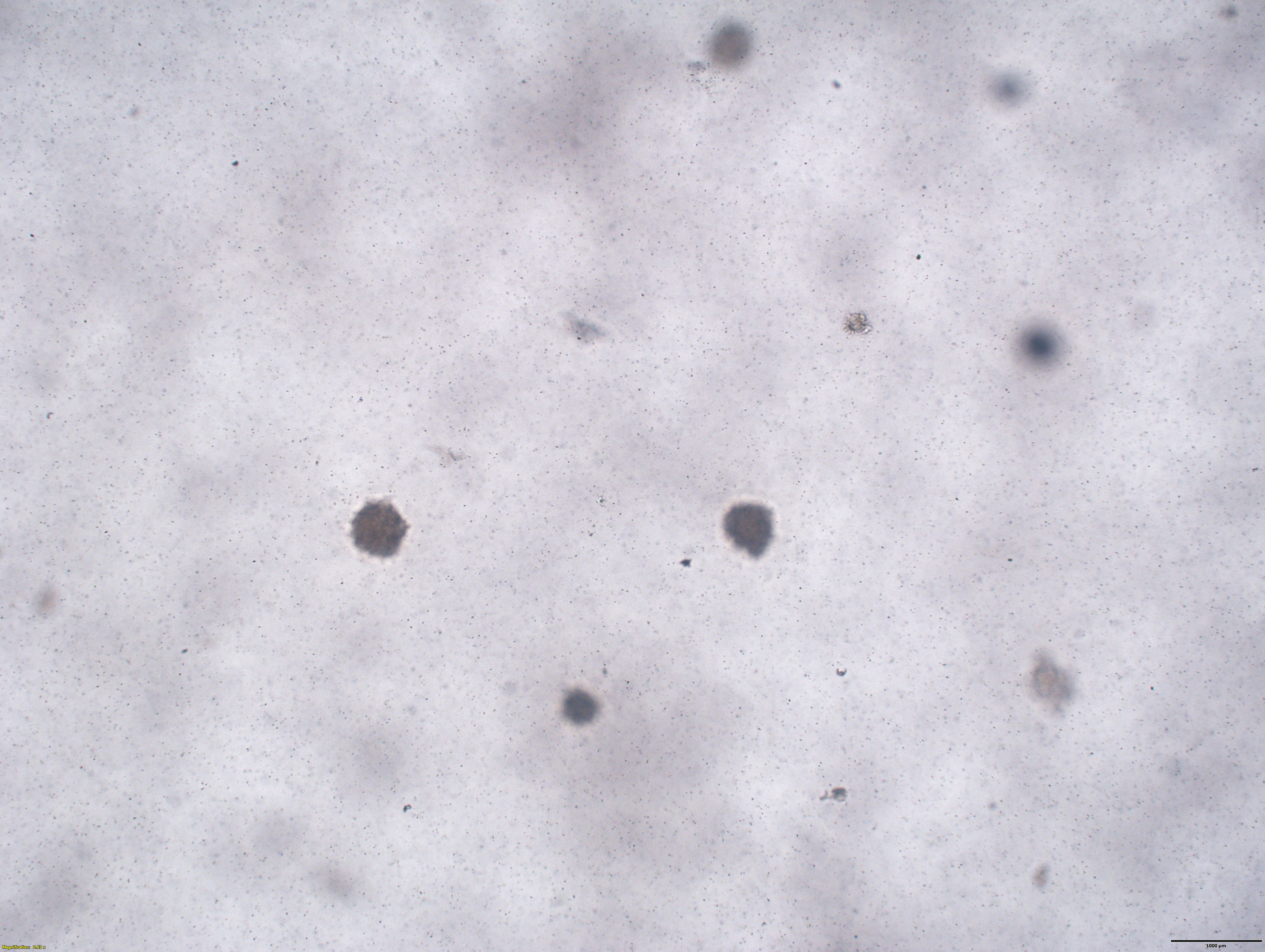

Supplement: Supplementary file 10 — Source data Fig. 5 [file 44321_2025_333_MOESM10_ESM.zip › Figure 5/5C/SNU-C1/Rep 2/2_OE.jpg]

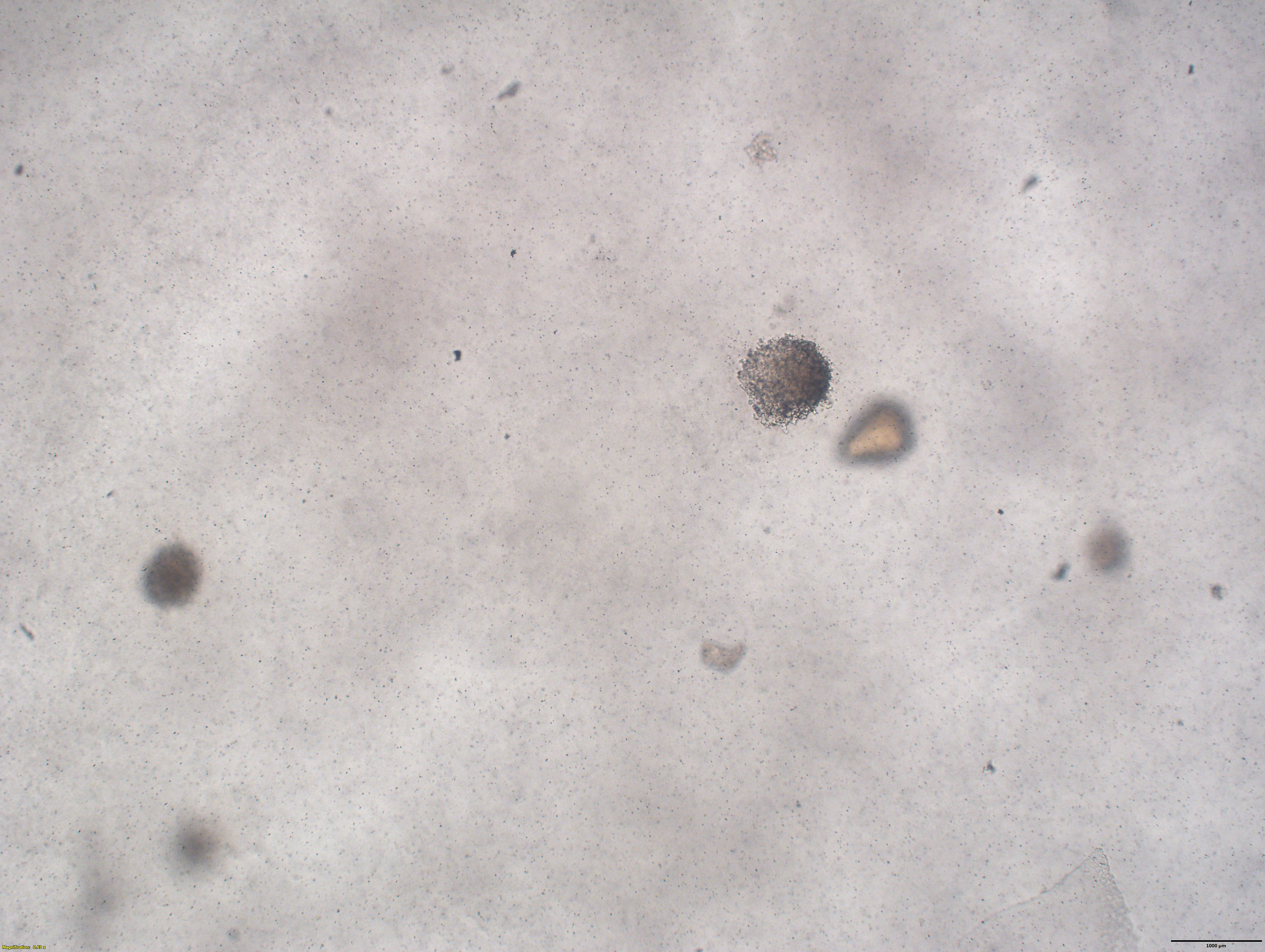

Supplement: Supplementary file 10 — Source data Fig. 5 [file 44321_2025_333_MOESM10_ESM.zip › Figure 5/5C/SNU-C1/Rep 2/3_OE+NC.jpg]

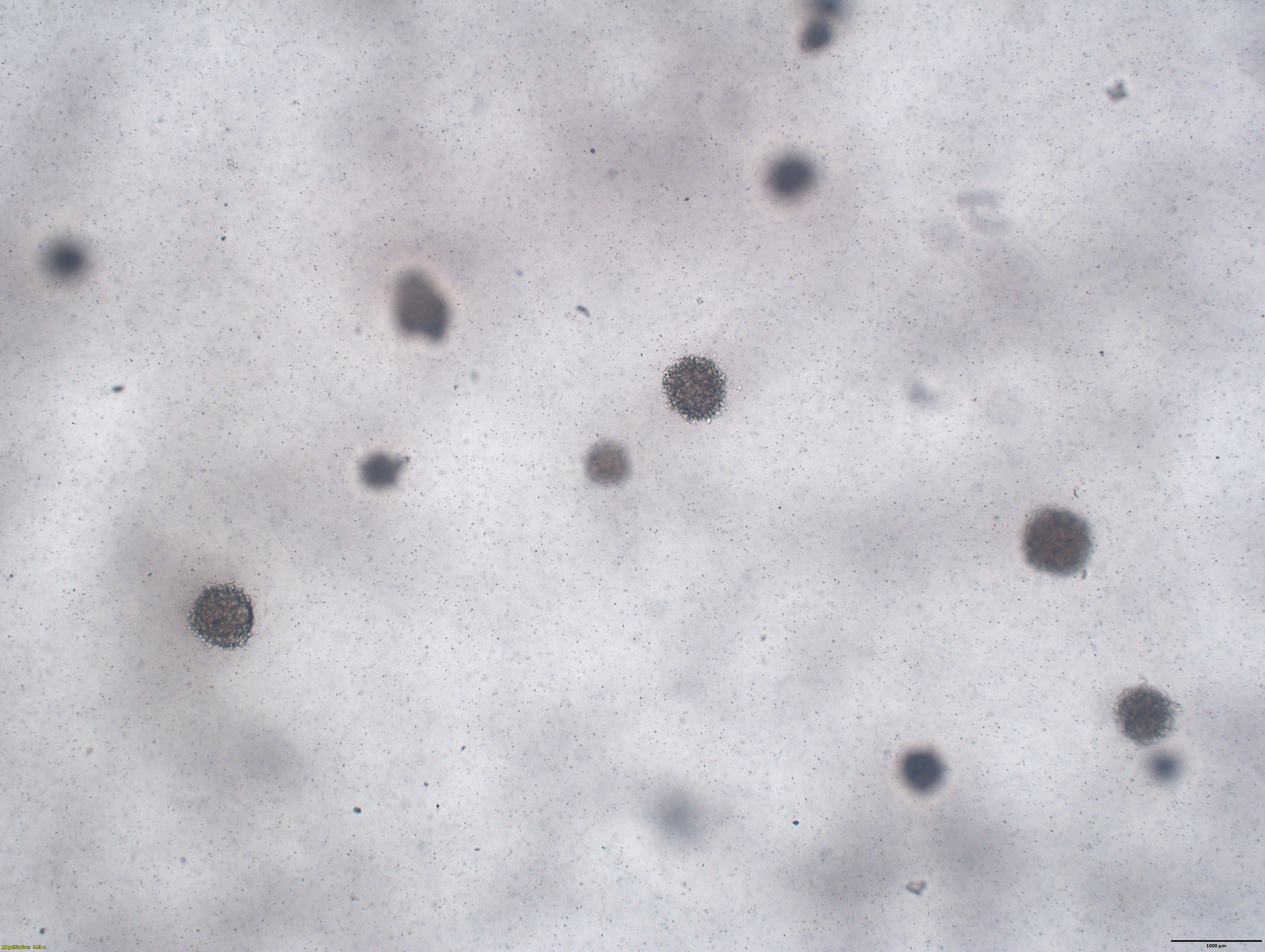

Supplement: Supplementary file 10 — Source data Fig. 5 [file 44321_2025_333_MOESM10_ESM.zip › Figure 5/5C/SNU-C1/Rep 2/4_OE+OE.jpg]

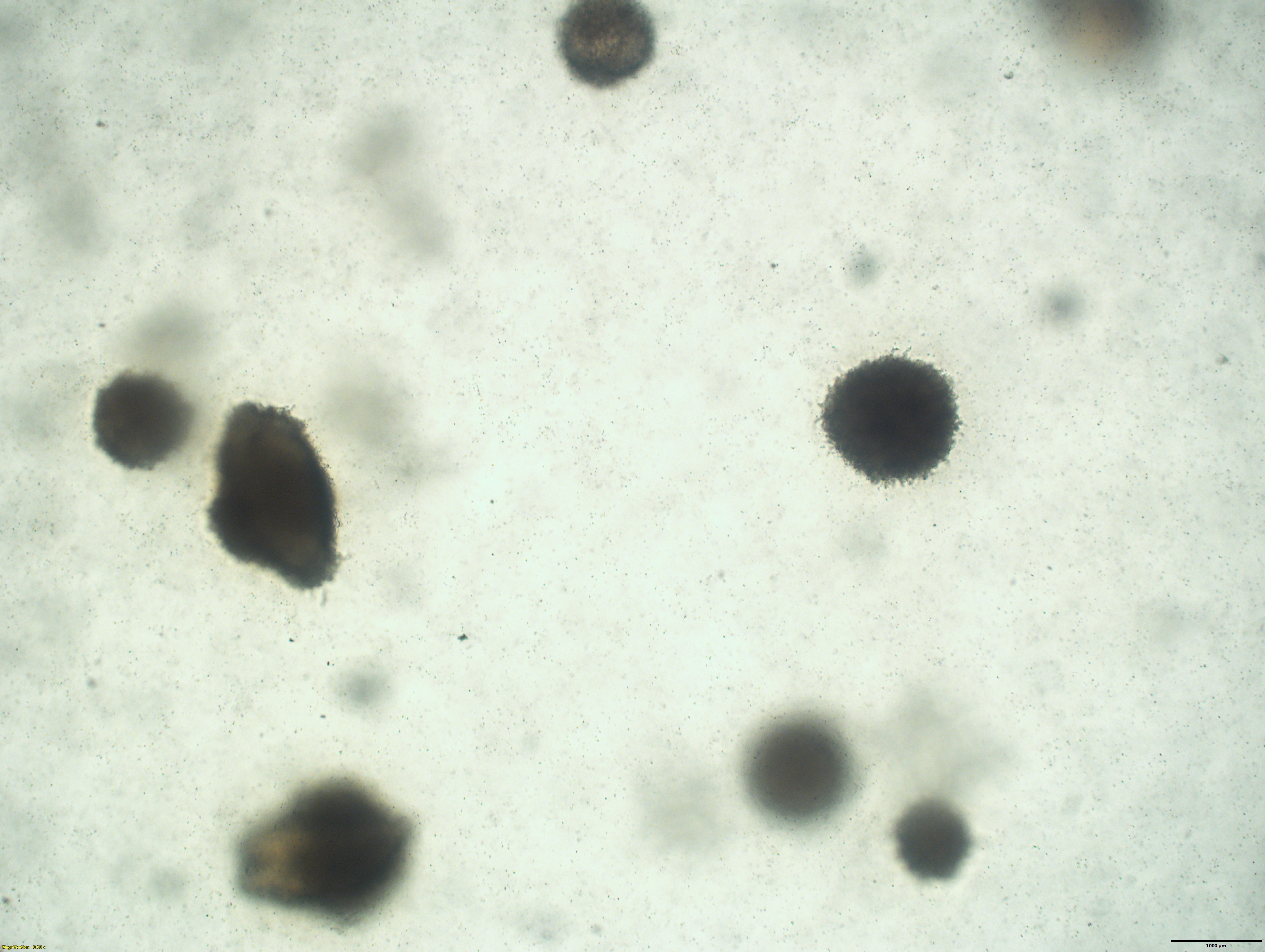

Supplement: Supplementary file 10 — Source data Fig. 5 [file 44321_2025_333_MOESM10_ESM.zip › Figure 5/5C/SNU-C1/Rep 3/1_NC.jpg]

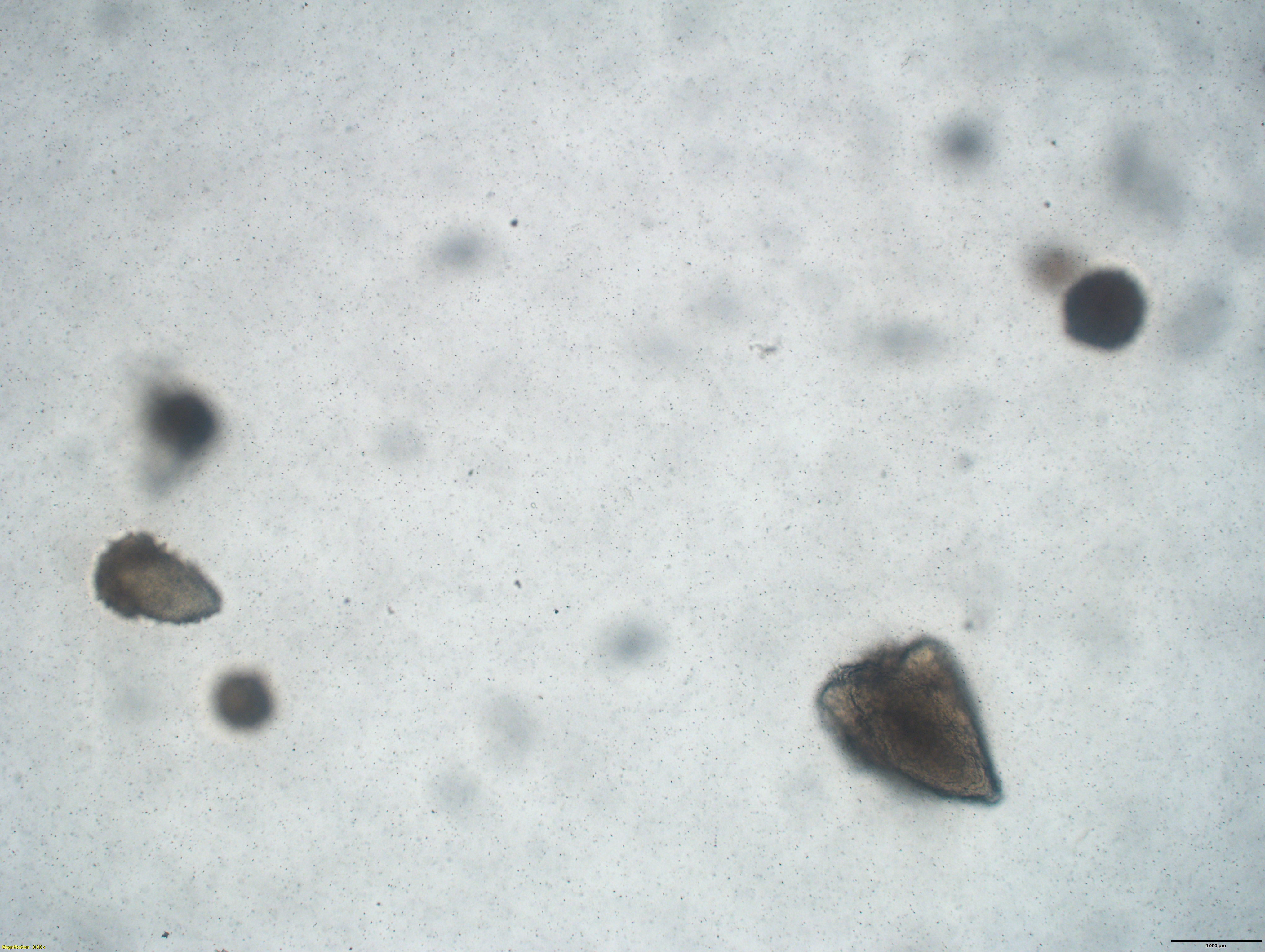

Supplement: Supplementary file 10 — Source data Fig. 5 [file 44321_2025_333_MOESM10_ESM.zip › Figure 5/5C/SNU-C1/Rep 3/2_OE.jpg]

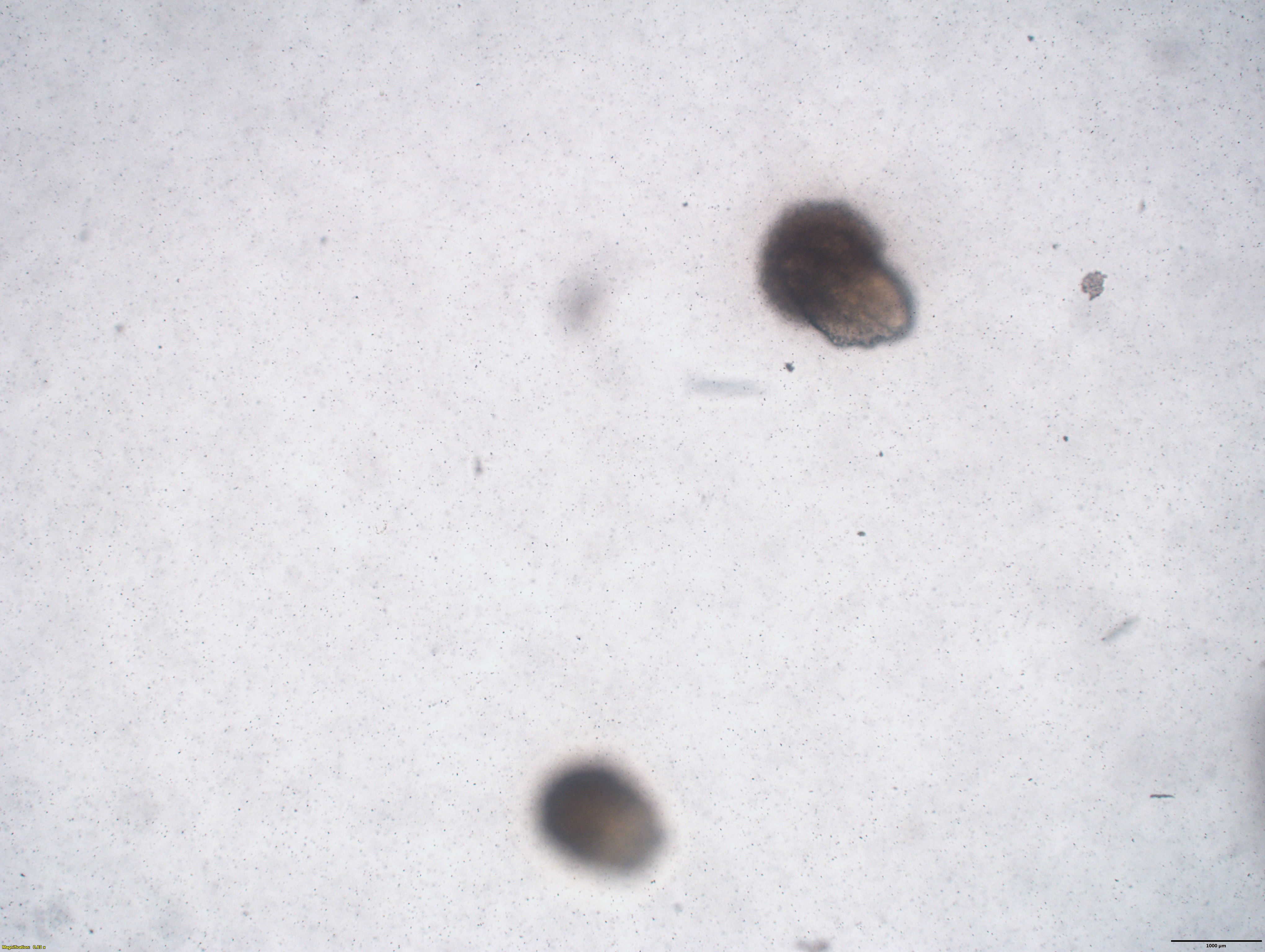

Supplement: Supplementary file 10 — Source data Fig. 5 [file 44321_2025_333_MOESM10_ESM.zip › Figure 5/5C/SNU-C1/Rep 3/3_OE+NC.jpg]

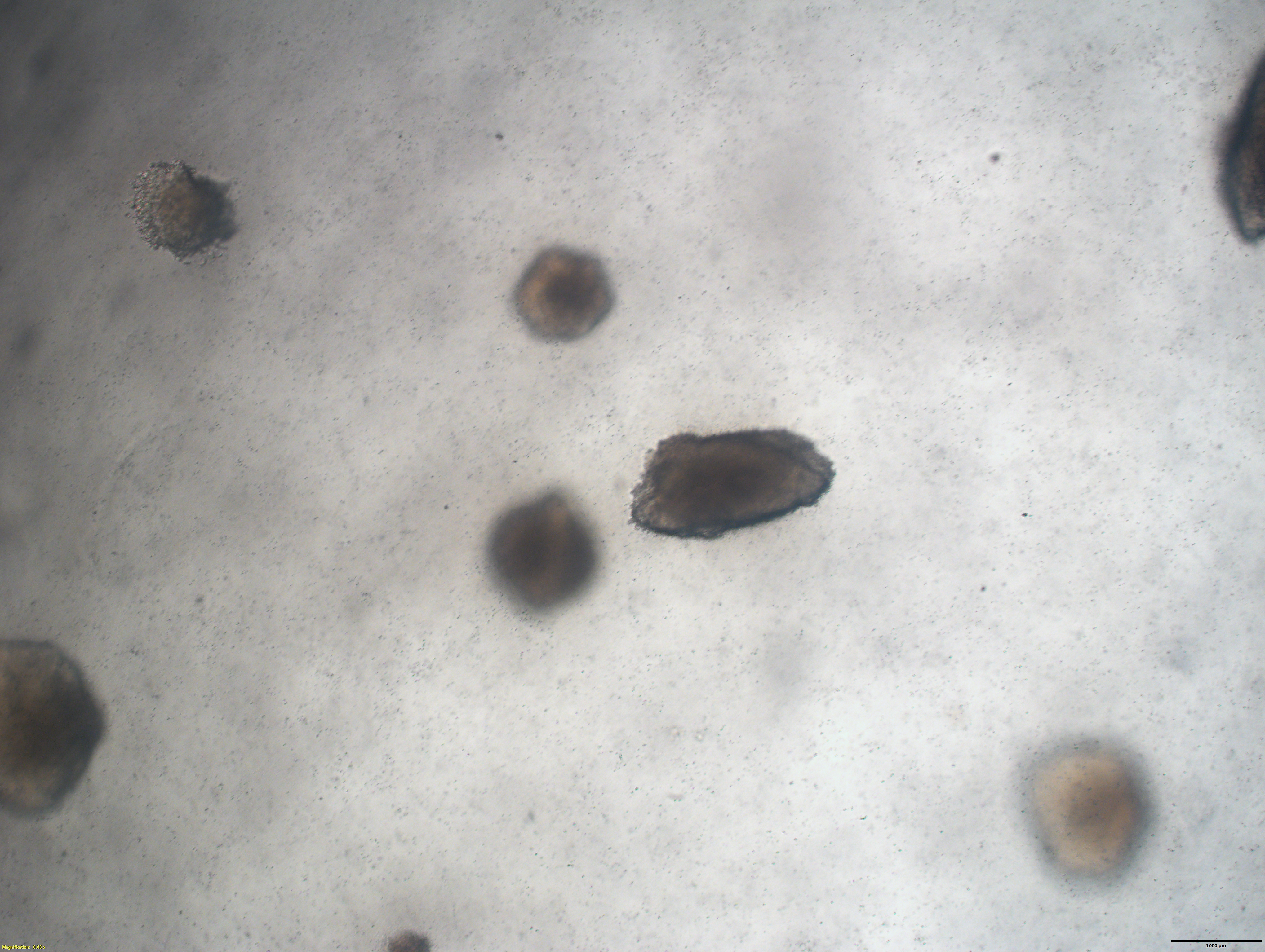

Supplement: Supplementary file 10 — Source data Fig. 5 [file 44321_2025_333_MOESM10_ESM.zip › Figure 5/5C/SNU-C1/Rep 3/4_OE+OE.jpg]

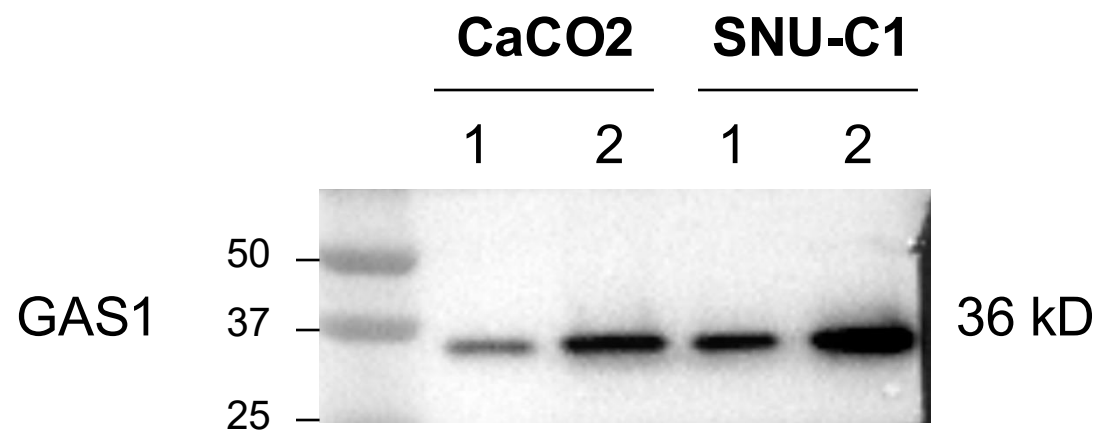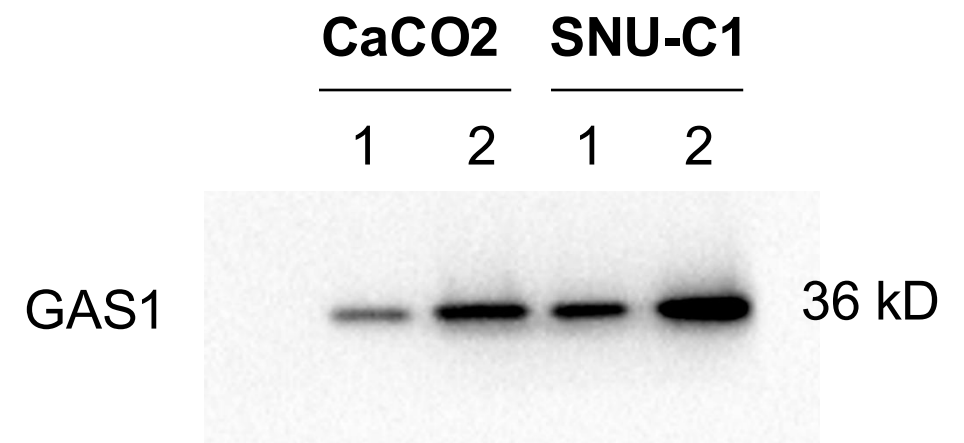

1. EV

2. OE circ-EGFR

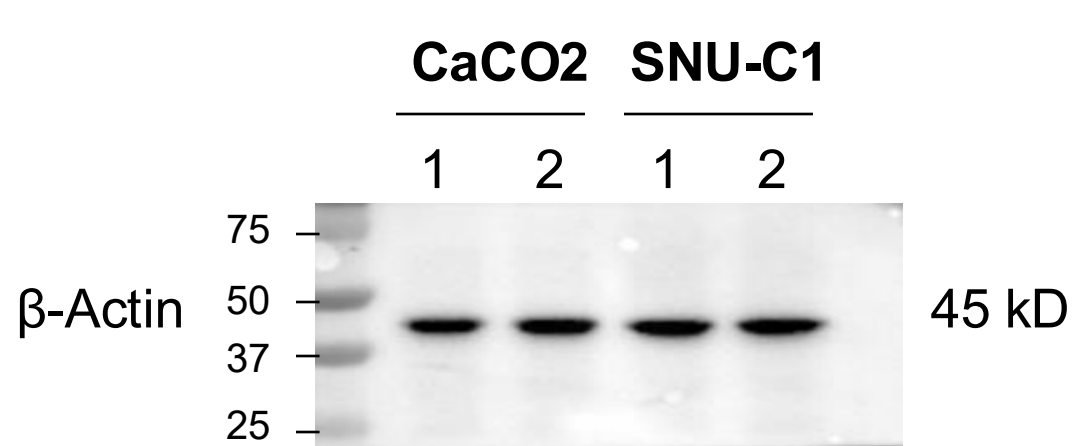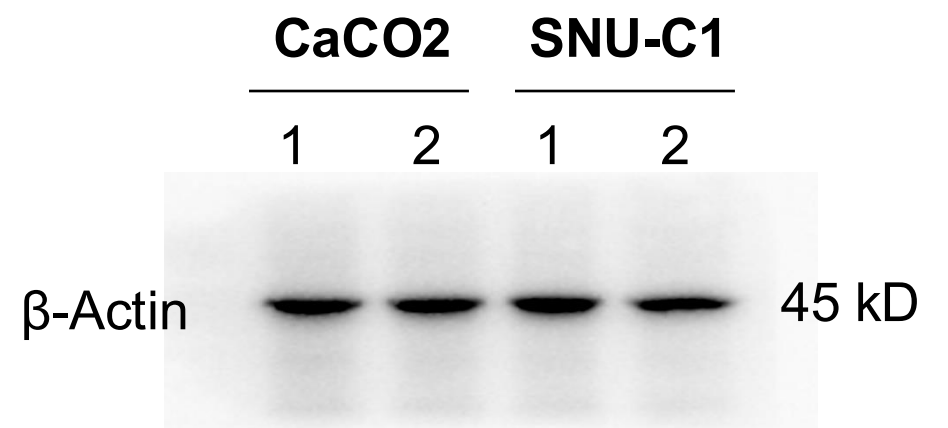

1. EV
2. OE circ-EGFR

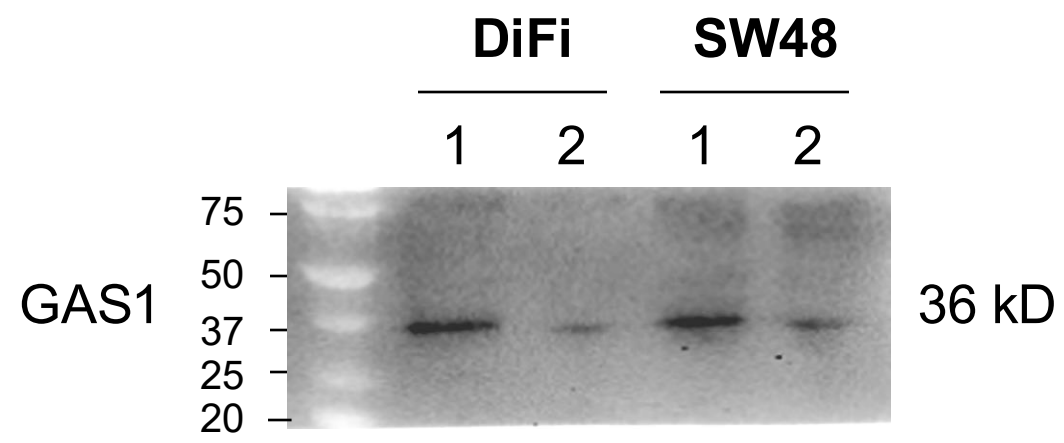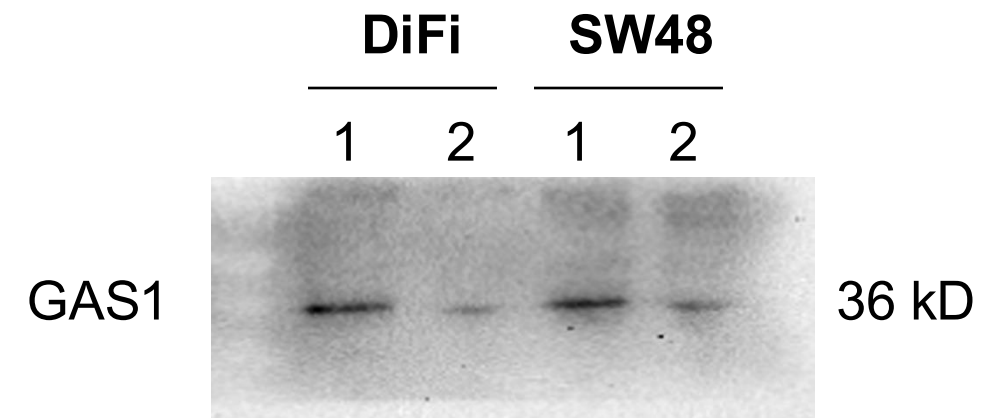

1. EV
2. OE miR-942-3p

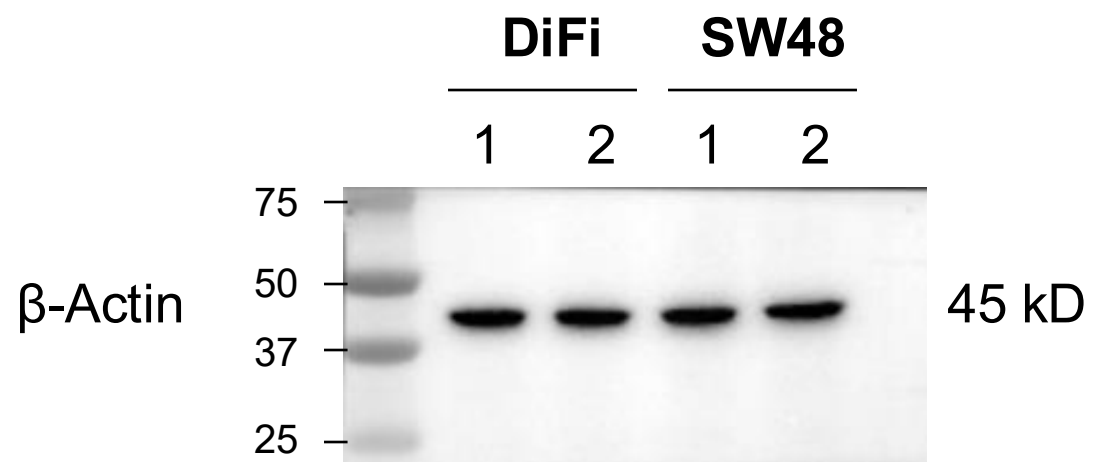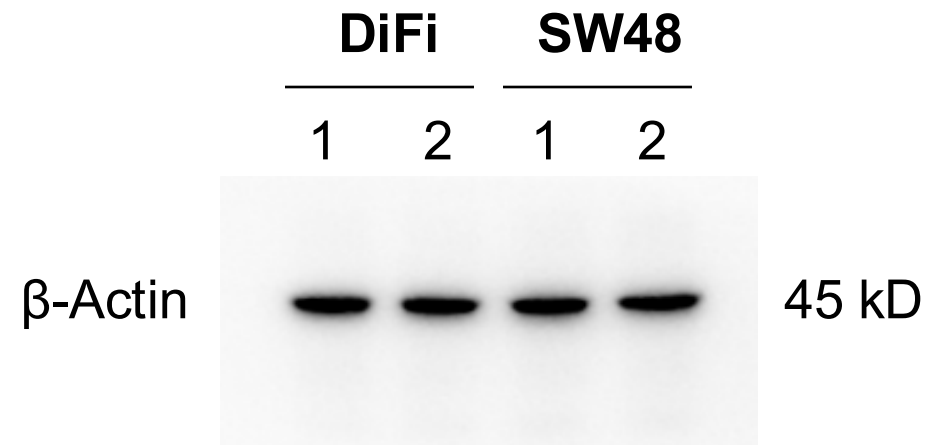

1. EV
2. OE miR-942-3p

Supplement: Supplementary file 11 — Source data Fig. 6 [file 44321_2025_333_MOESM11_ESM.zip › Figure 6/6C/Fiugre 6C_WB.pdf]

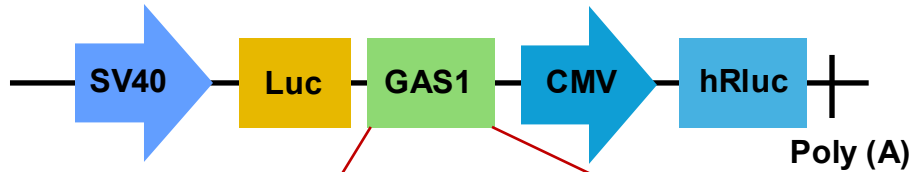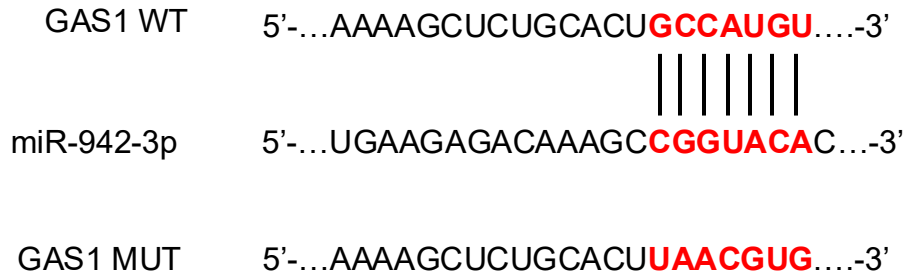

Supplement: Supplementary file 11 — Source data Fig. 6 [file 44321_2025_333_MOESM11_ESM.zip › Figure 6/6D/Figure 6D_Luciferase.pdf]
