# Supplementary material for: circ-EGFR is a predictor of response to Cetuximab and a potential target in colorectal cancer
Source: EMBO Mol Med. 2025 Nov 10;17(12):3525–54. doi: 10.1038/s44321-025-00333-0 (PMC12686431; doi:10.1038/s44321-025-00333-0)
Supplement: Supplementary file 11 — Source data Fig. 6 [file 44321_2025_333_MOESM11_ESM.zip › Figure 6/6C/Fiugre 6C_WB.pptx]

## Slide 1
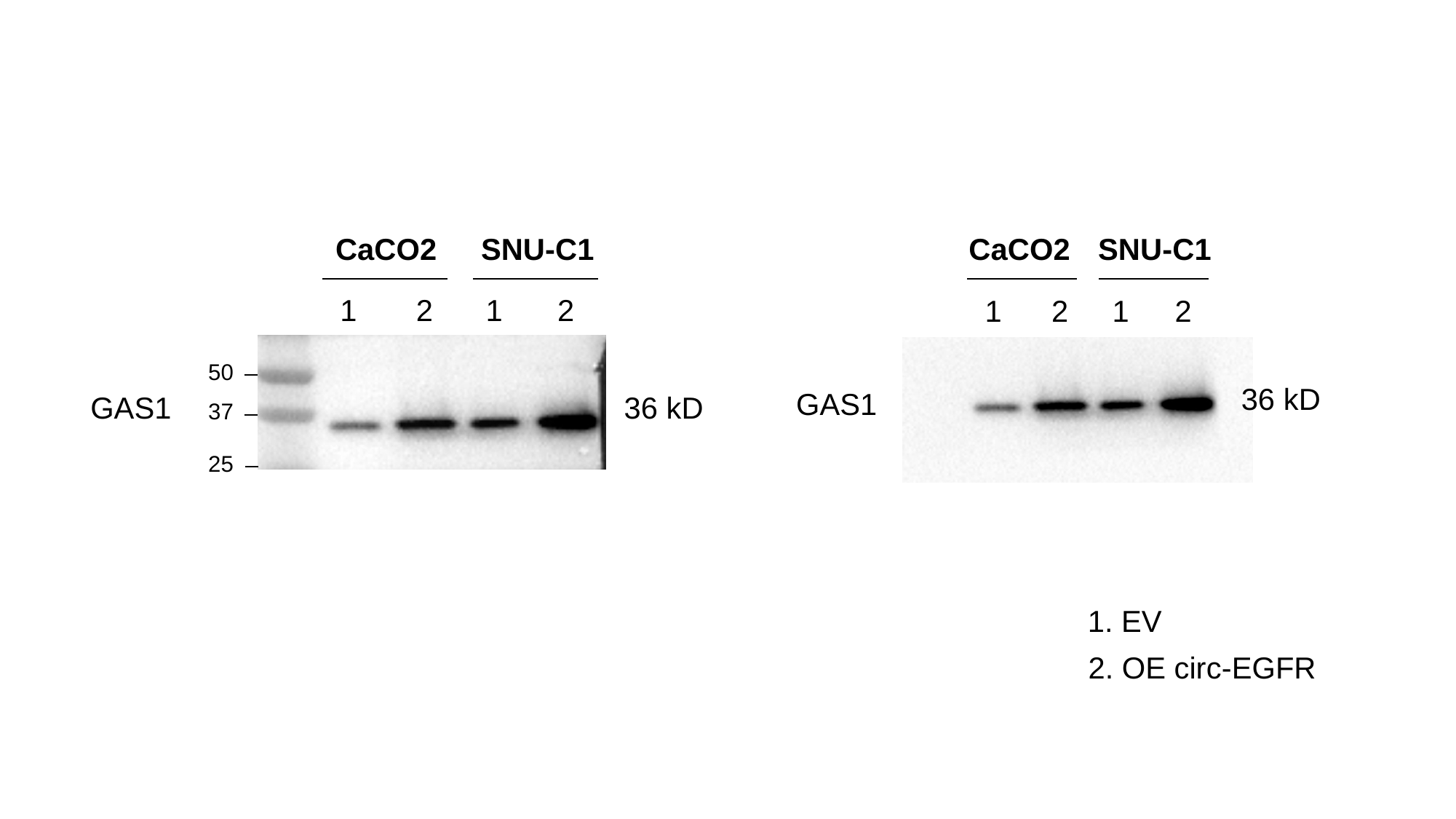

CaCO2
SNU-C1
CaCO2
SNU-C1
1
2
1
2
36 kD
GAS1
1
2
1
2
50
GAS1
36 kD
37
25
1. EV
2. OE circ-EGFR

## Slide 2
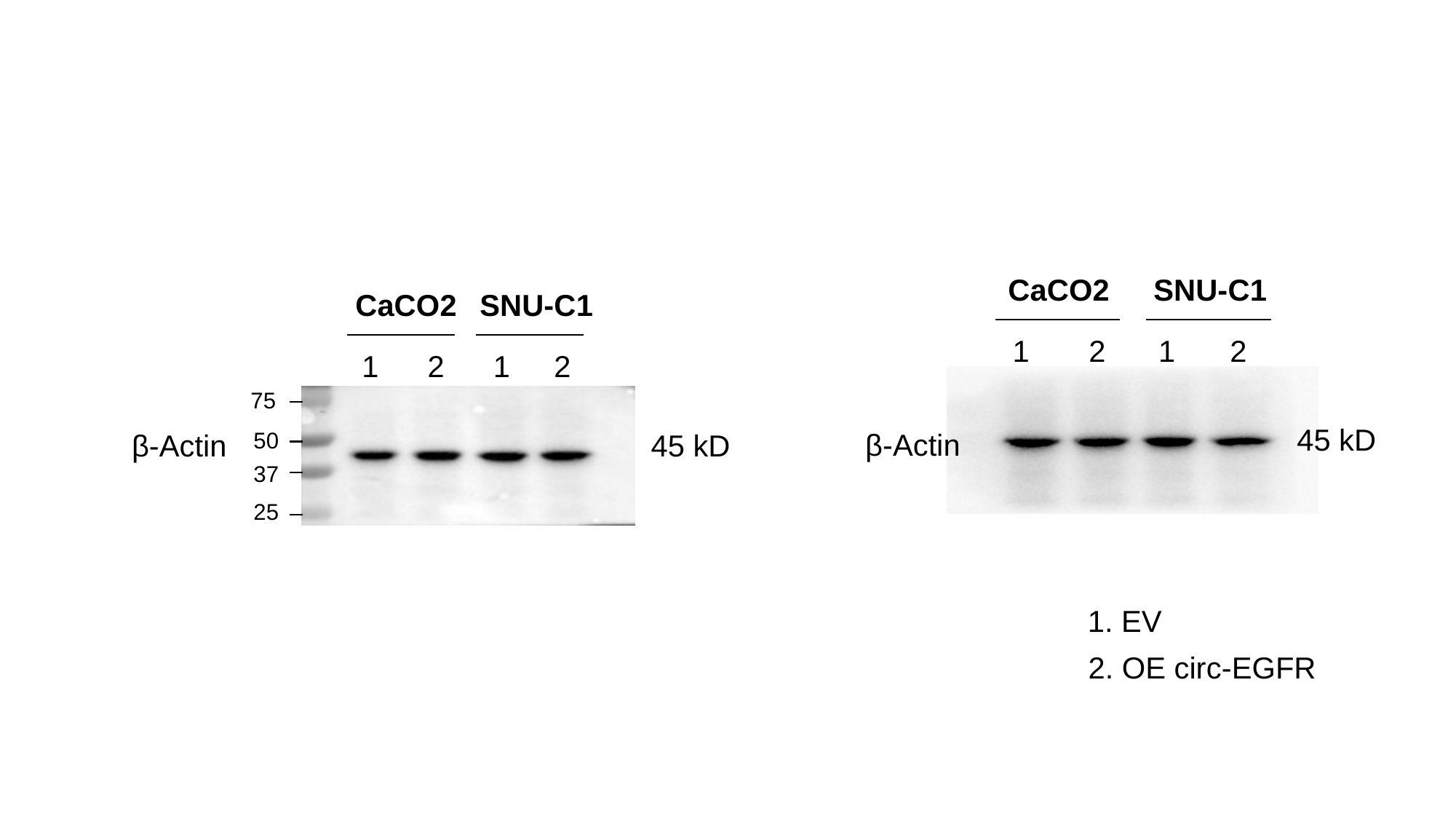

CaCO2
SNU-C1
CaCO2
SNU-C1
1
2
1
2
1
2
1
2
75
50
37
25
45 kD
β-Actin
β-Actin
45 kD
1. EV
2. OE circ-EGFR

## Slide 3
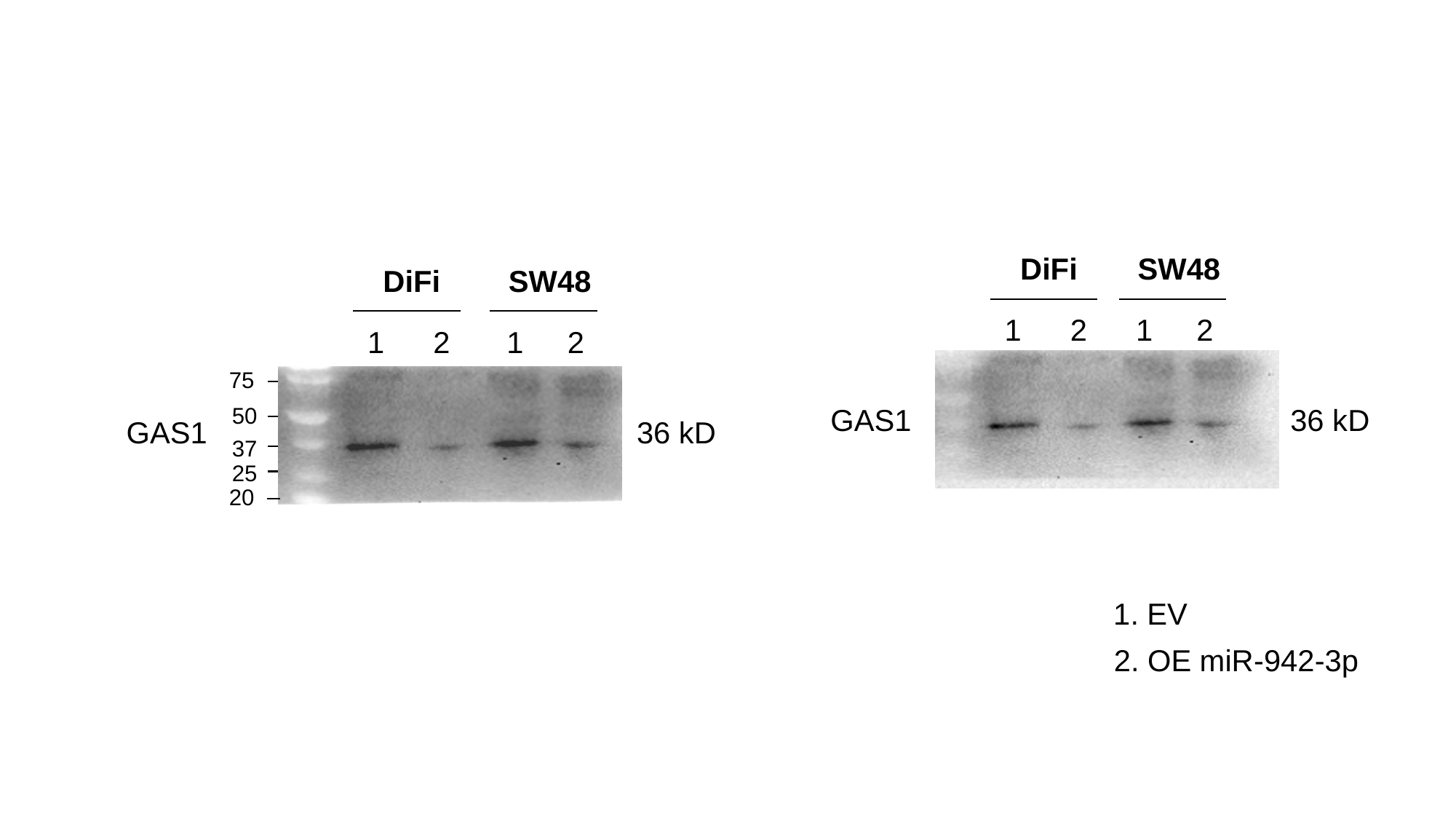

DiFi
SW48
1
2
1
2
GAS1
36 kD
DiFi
SW48
1
2
1
2
75
50
GAS1
36 kD
37
25
20
1. EV
2. OE miR-942-3p

## Slide 4
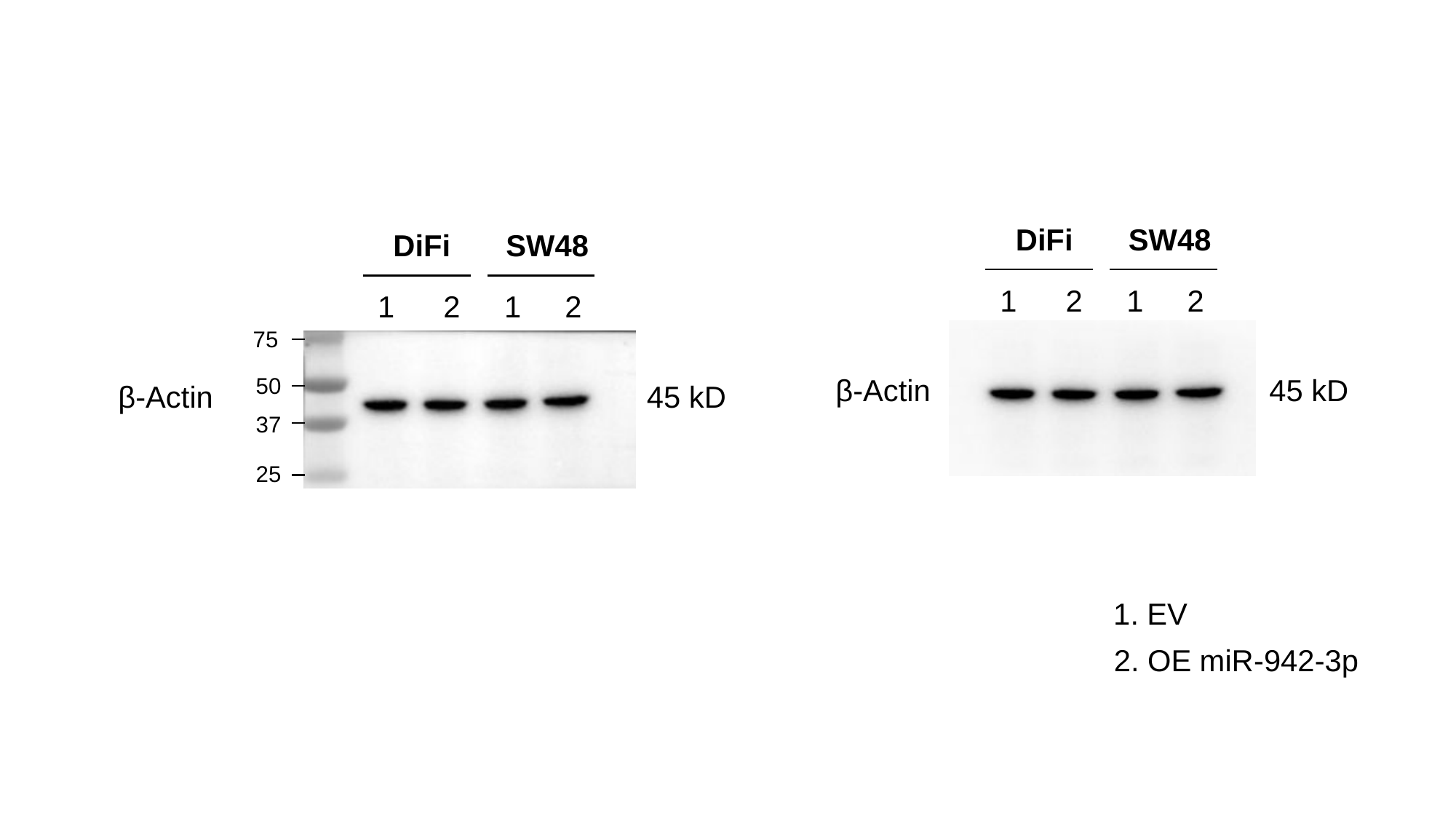

DiFi
SW48
1
2
1
2
β-Actin
45 kD
DiFi
SW48
1
2
1
2
75
50
β-Actin
45 kD
37
25
1. EV
2. OE miR-942-3p
