# Supplementary material for: circ-EGFR is a predictor of response to Cetuximab and a potential target in colorectal cancer
Source: EMBO Mol Med. 2025 Nov 10;17(12):3525–54. doi: 10.1038/s44321-025-00333-0 (PMC12686431; doi:10.1038/s44321-025-00333-0)
Supplement: Supplementary file 11 — Source data Fig. 6 [file 44321_2025_333_MOESM11_ESM.zip › Figure 6/6D/Figure 6D_Luciferase.pptx]

## Slide 1
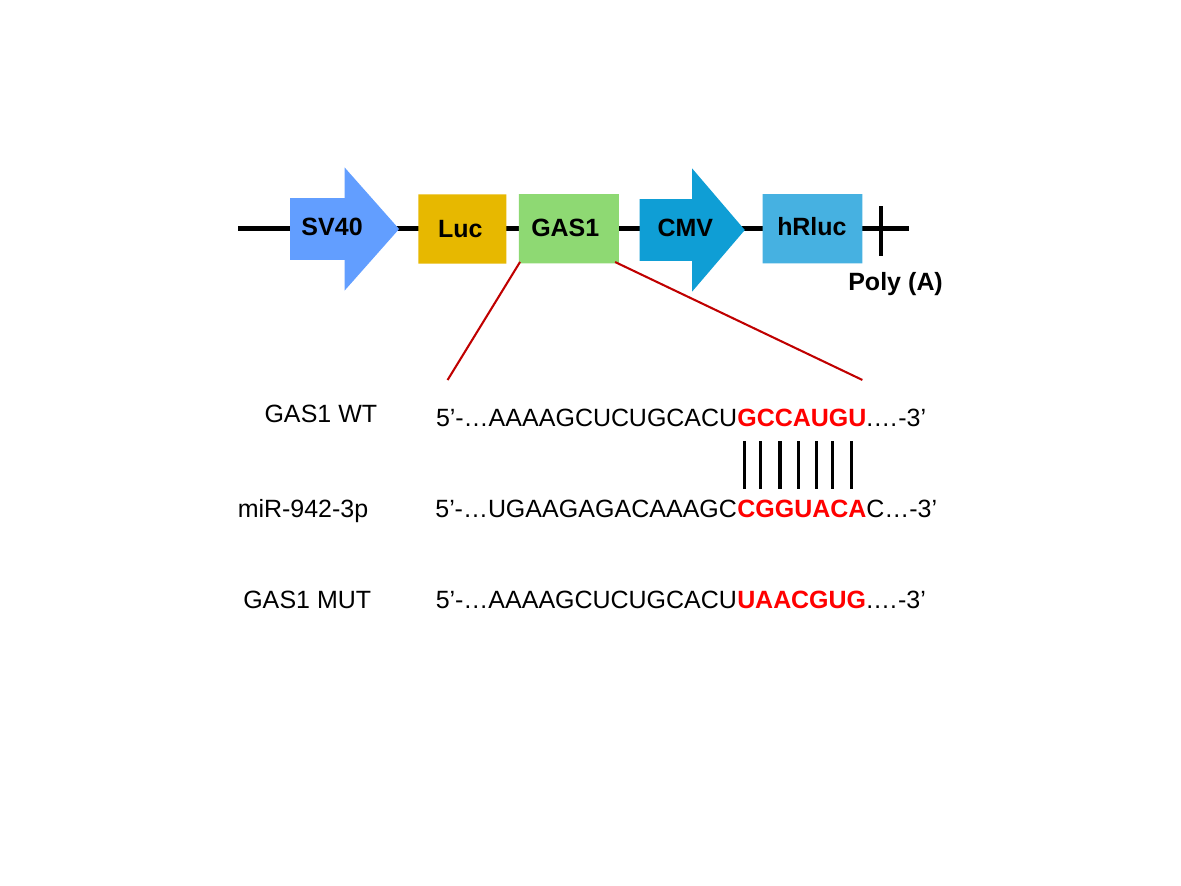

SV40
hRluc
GAS1
CMV
Luc
Poly (A)
GAS1 WT
5’-…AAAAGCUCUGCACUGCCAUGU.…-3’
miR-942-3p
5’-…UGAAGAGACAAAGCCGGUACAC…-3’
GAS1 MUT
5’-…AAAAGCUCUGCACUUAACGUG.…-3’
