# Supplementary material for: circ-EGFR is a predictor of response to Cetuximab and a potential target in colorectal cancer
Source: EMBO Mol Med. 2025 Nov 10;17(12):3525–54. doi: 10.1038/s44321-025-00333-0 (PMC12686431; doi:10.1038/s44321-025-00333-0)
Supplement: Supplementary file 11 — Source data Fig. 6 [file 44321_2025_333_MOESM11_ESM.zip › Figure 6/6F/Figure 6F_WB.pdf]

CaCO2

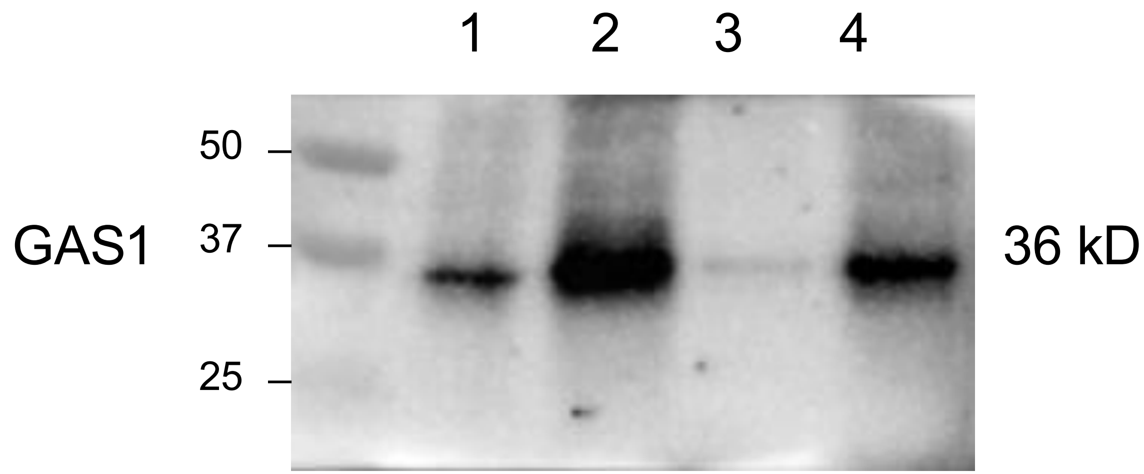

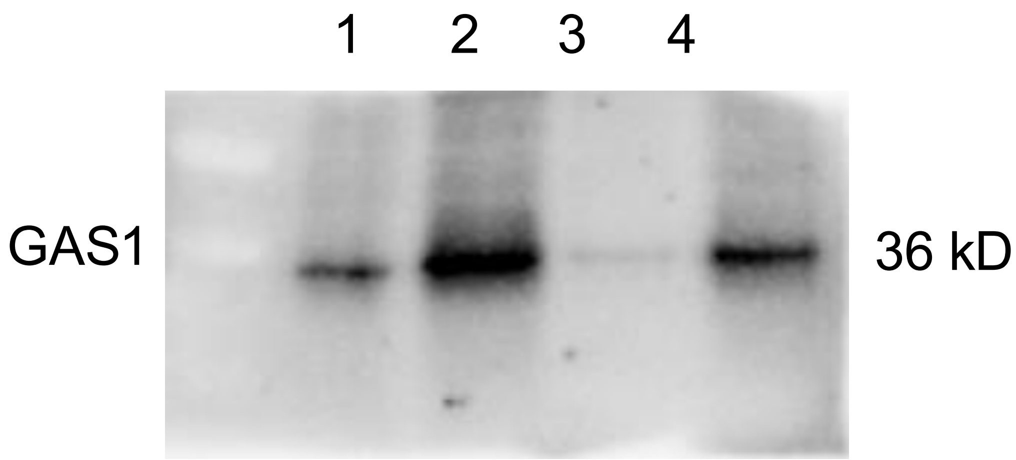

1. circc-EV + miR-NC
2. circ-OE + miR-NC
3. circ-EV + miR-OE
4. circ-OE + miR-OE

SNU-C1

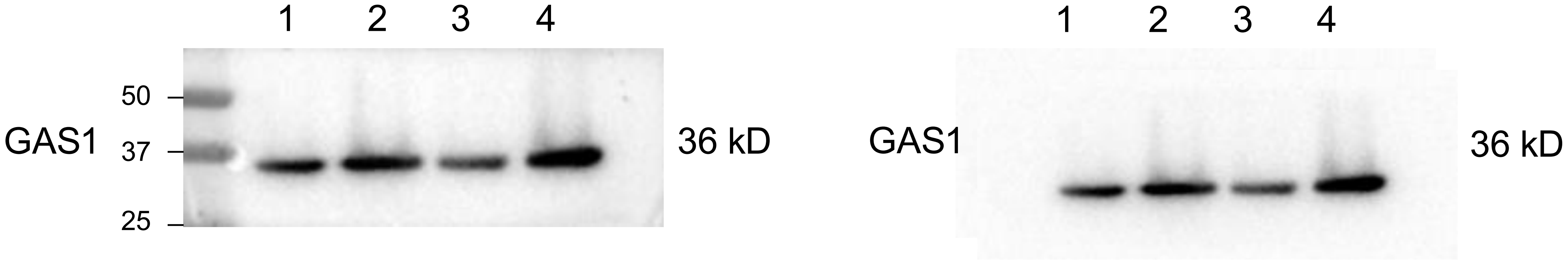

1. circ-EV + miR-NC
2. circ-OE + miR-NC
3. circ-EV + miR-OE
4. circ-OE + miR-OE

CaCO2

Gli1

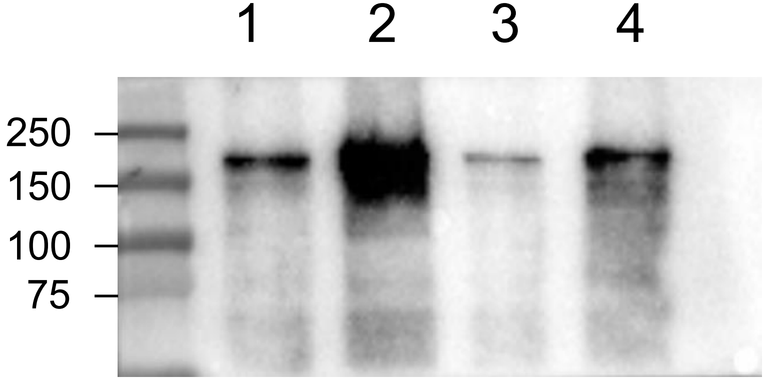

160 kD

Gli1

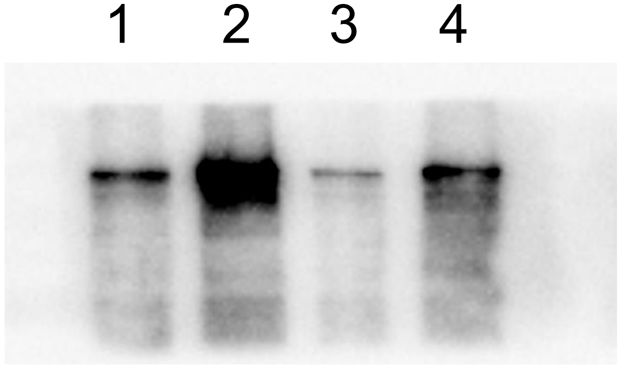

160 kD

1. circ-EV + miR-NC
2. circ-OE + miR-NC
3. circ-EV + miR-OE
4. circ-OE + miR-OE

SNU-C1

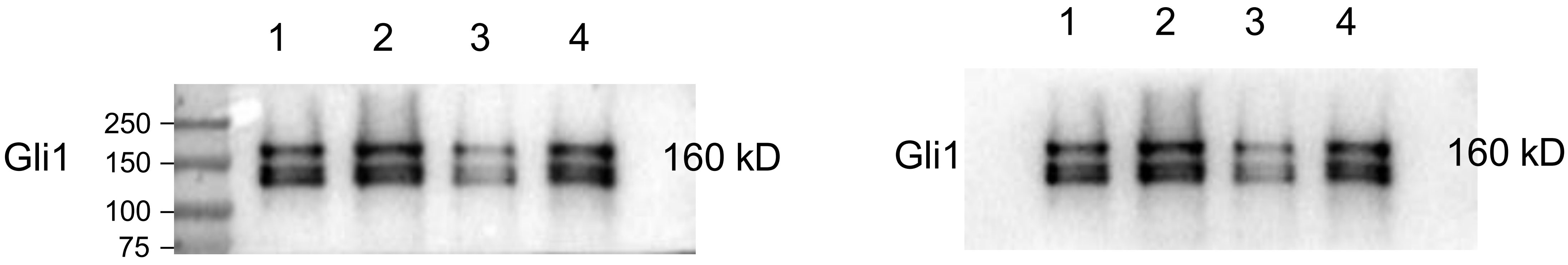

1. circ-EV + miR-NC
2. circ-OE + miR-NC
3. circ-EV + miR-OE
4. circ-OE + miR-OE

CaCO2

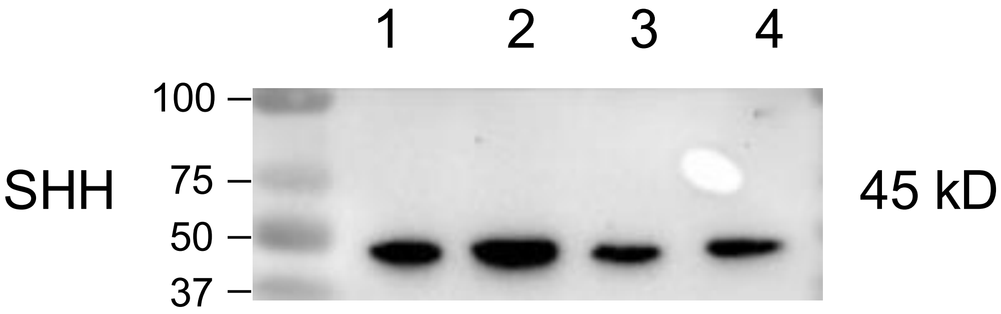

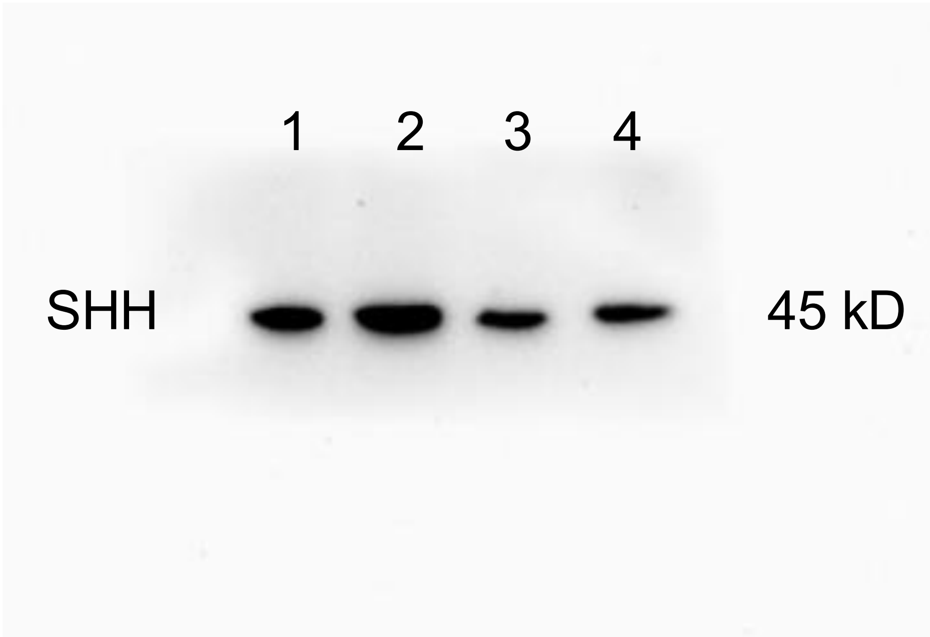

1. circ-EV + miR-NC
2. circ-OE + miR-NC
3. circ-EV + miR-OE
4. circ-OE + miR-OE

SNU-C1

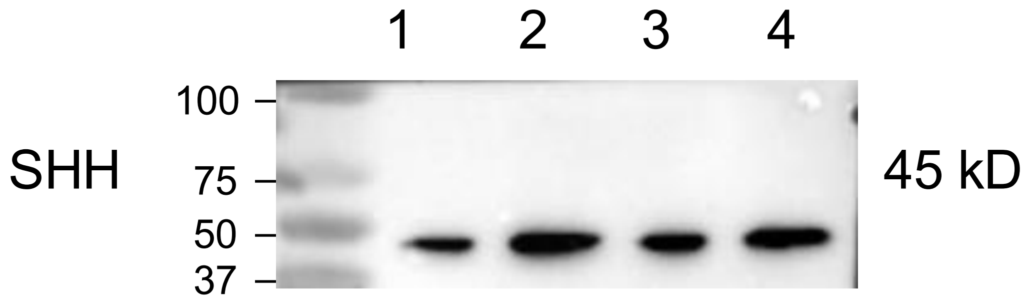

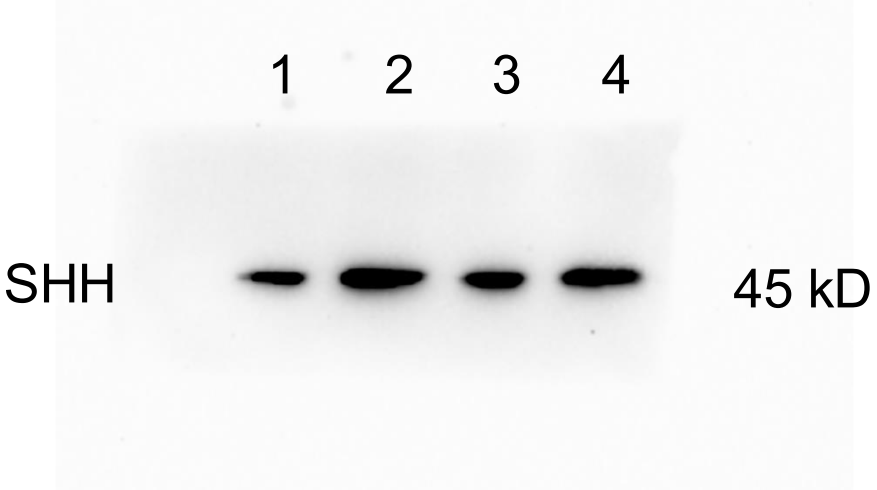

1. circc-EV + miR-NC
2. circ-OE + miR-NC
3. circ-EV + miR-OE
4. circ-OE + miR-OE

CaCO2

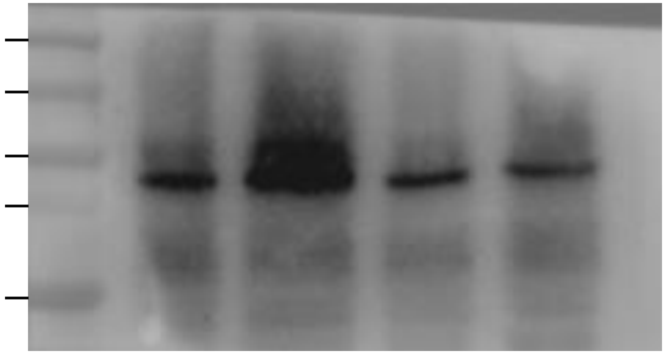

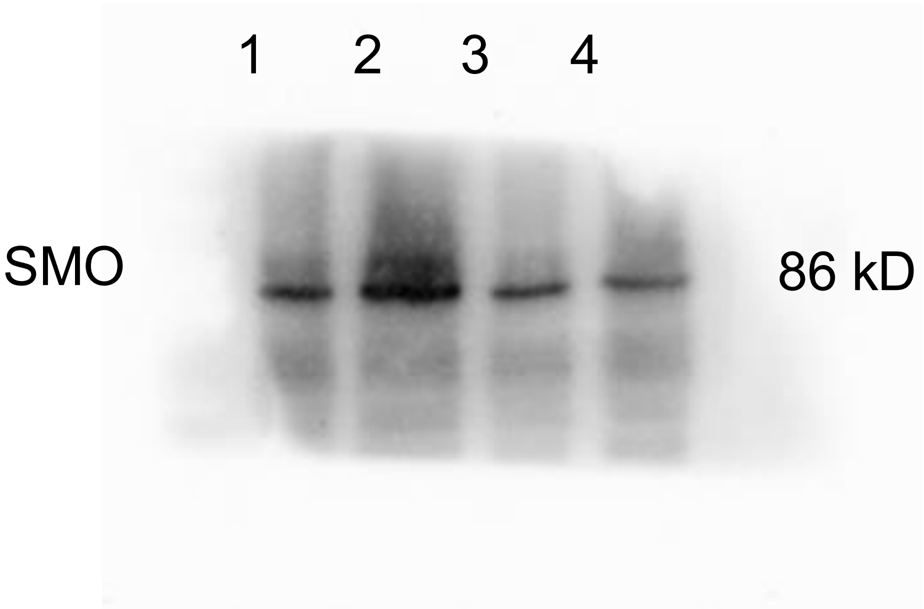

1. circ-EV + miR-NC
2. circ-OE + miR-NC
3. circ-EV + miR-OE
4. circ-OE + miR-OE

SNU-C1

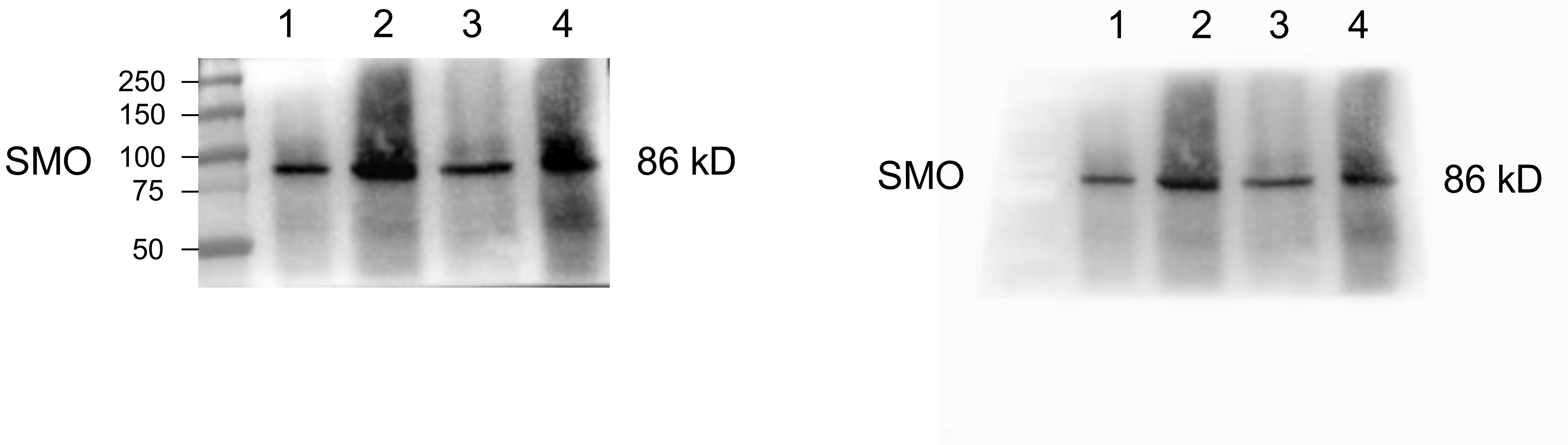

1. circc-EV + miR-NC
2. circ-OE + miR-NC
3. circ-EV + miR-OE
4. circ-OE + miR-OE

CaCO2

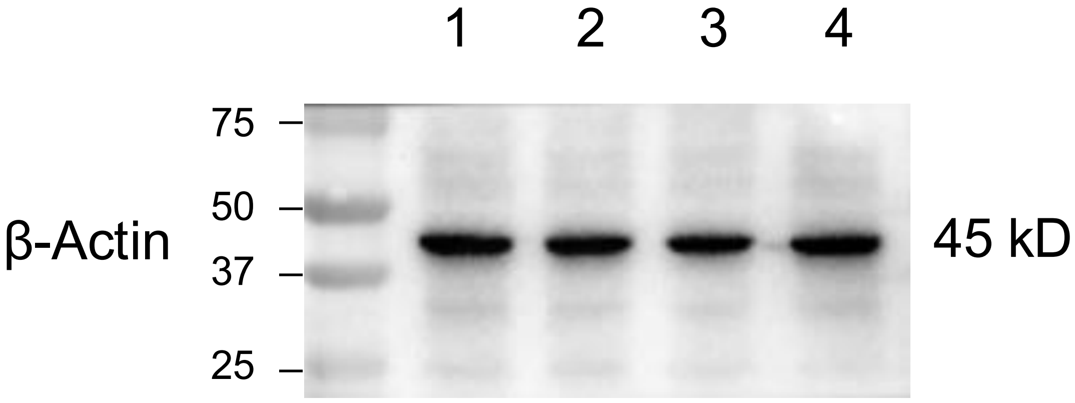

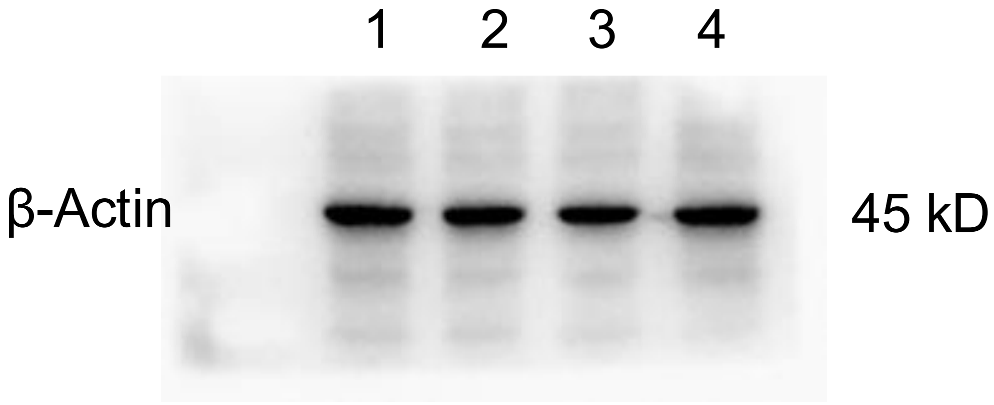

1. circ-EV + miR-NC
2. circ-OE + miR-NC
3. circ-EV + miR-OE
4. circ-OE + miR-OE

SNU-C1

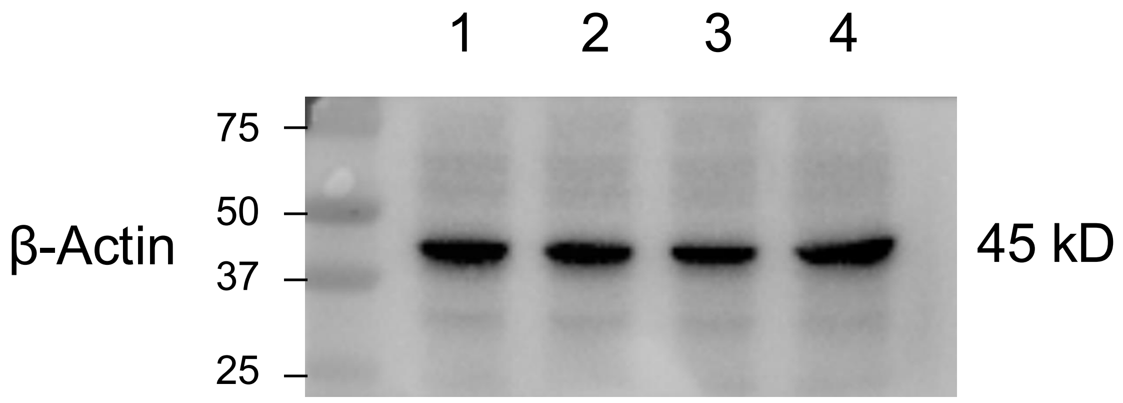

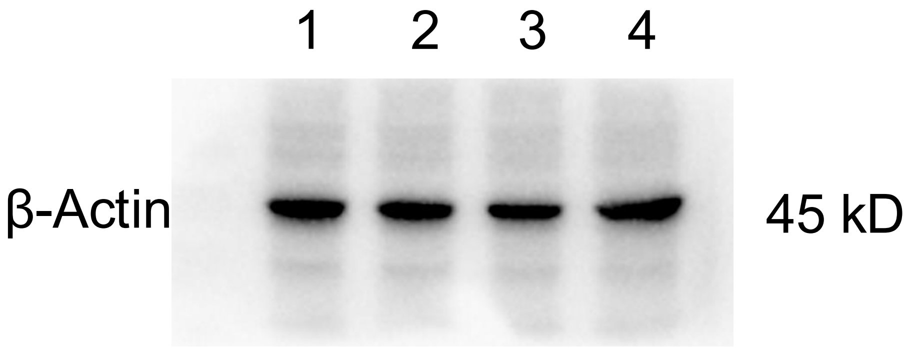

1. circc-EV + miR-NC
2. circ-OE + miR-NC
3. circ-EV + miR-OE
4. circ-OE + miR-OE
