# Supplementary material for: circ-EGFR is a predictor of response to Cetuximab and a potential target in colorectal cancer
Source: EMBO Mol Med. 2025 Nov 10;17(12):3525–54. doi: 10.1038/s44321-025-00333-0 (PMC12686431; doi:10.1038/s44321-025-00333-0)
Supplement: Supplementary file 11 — Source data Fig. 6 [file 44321_2025_333_MOESM11_ESM.zip › Figure 6/6F/Figure 6F_WB.pptx]

## Slide 1
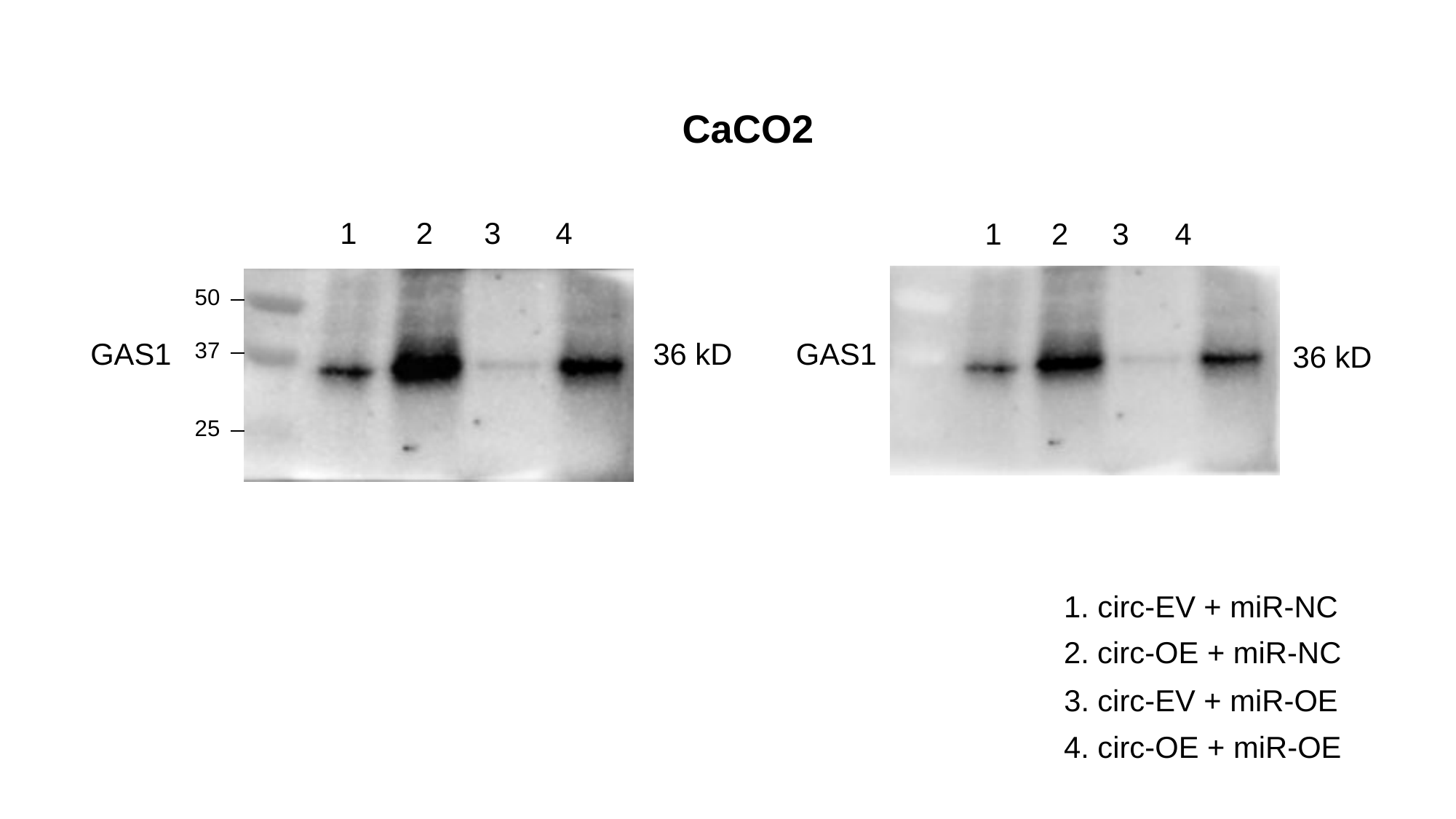

CaCO2
1
2
3
4
50
GAS1
36 kD
37
25
1
2
3
4
GAS1
36 kD
1. circ-EV + miR-NC
2. circ-OE + miR-NC
3. circ-EV + miR-OE
4. circ-OE + miR-OE

## Slide 2
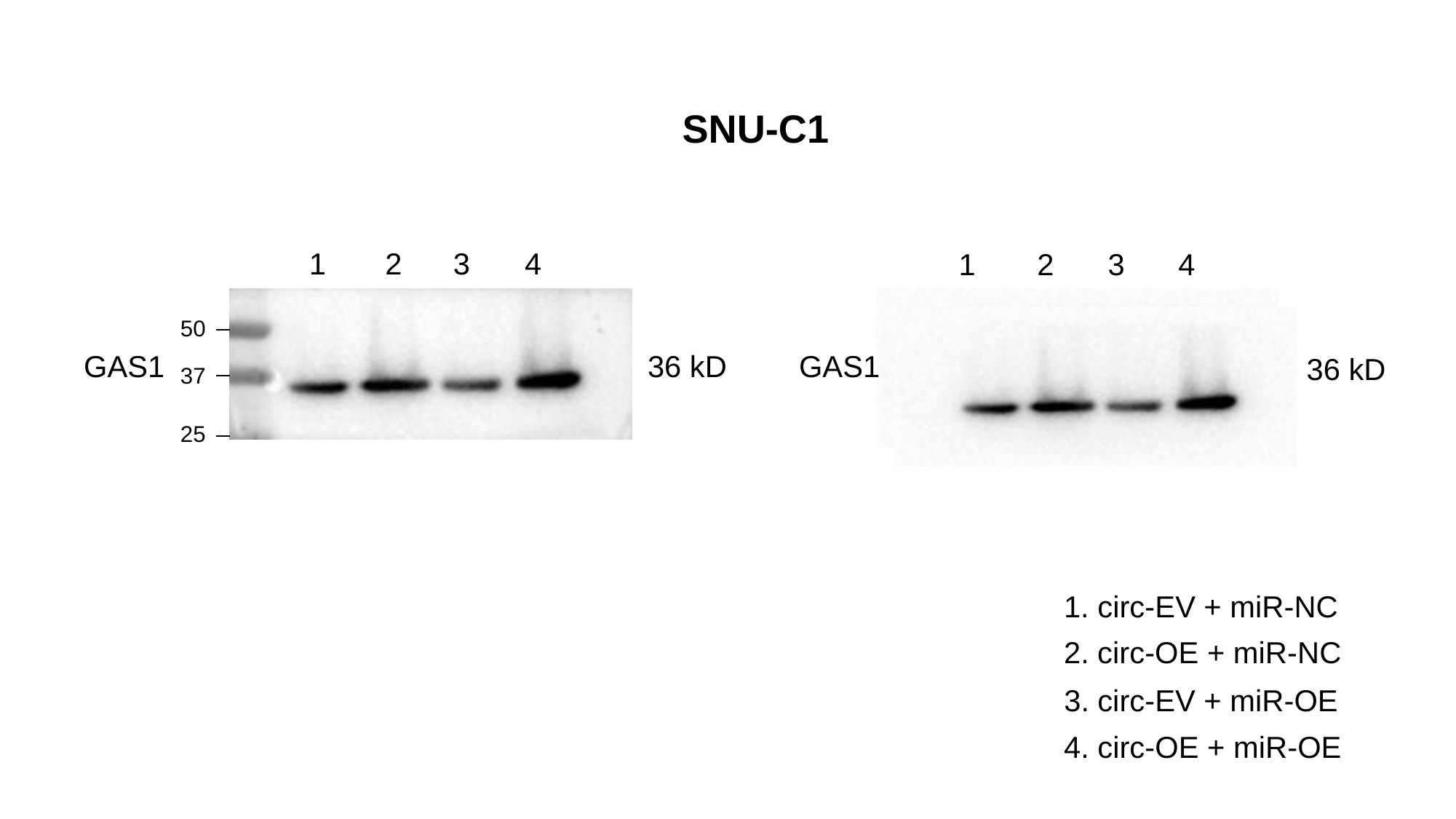

SNU-C1
1
2
3
4
50
GAS1
36 kD
37
25
1
2
3
4
GAS1
36 kD
1. circ-EV + miR-NC
2. circ-OE + miR-NC
3. circ-EV + miR-OE
4. circ-OE + miR-OE

## Slide 3
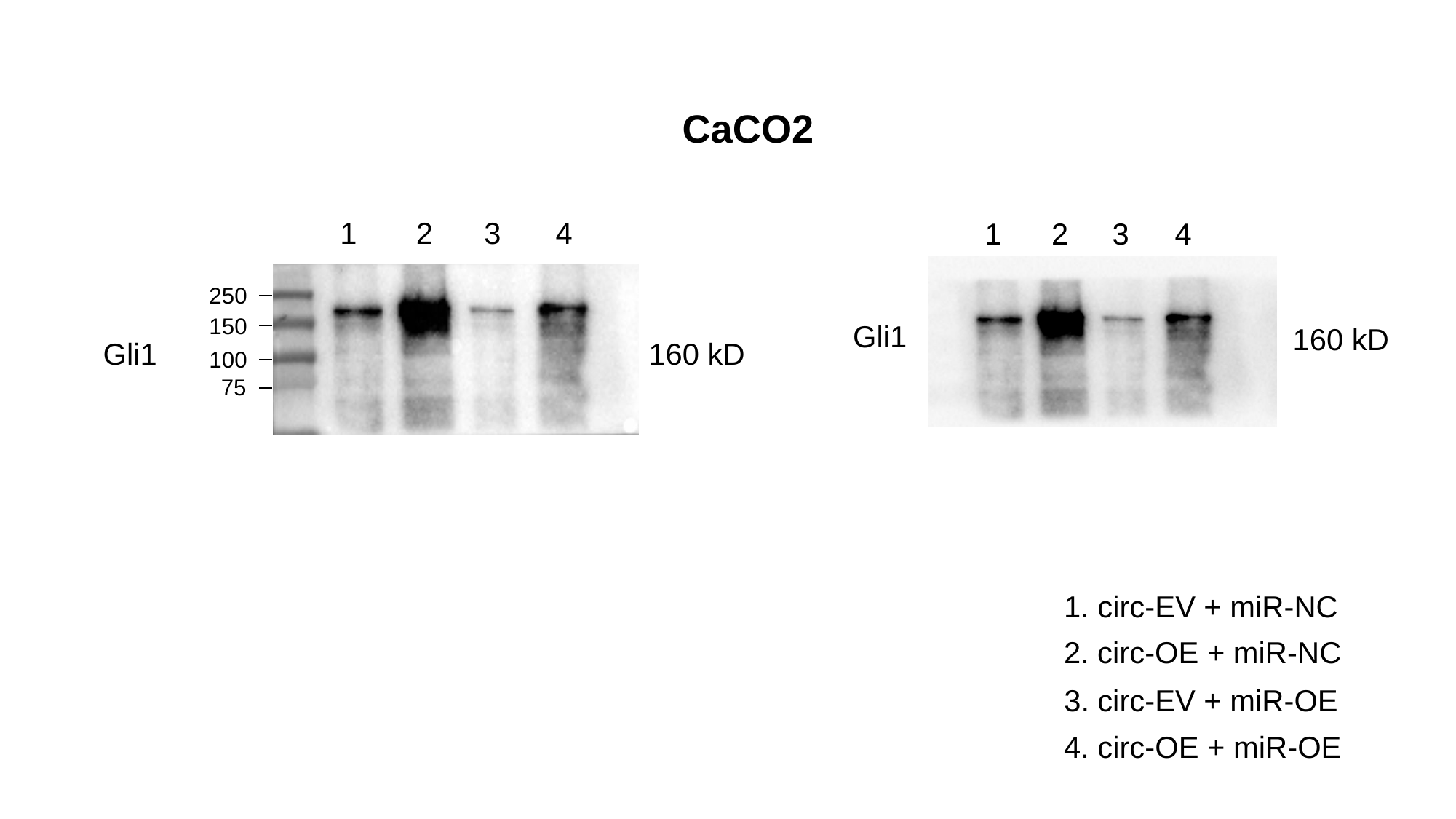

CaCO2
1
2
3
4
250
150
Gli1
160 kD
100
75
1
2
3
4
Gli1
160 kD
1. circ-EV + miR-NC
2. circ-OE + miR-NC
3. circ-EV + miR-OE
4. circ-OE + miR-OE

## Slide 4
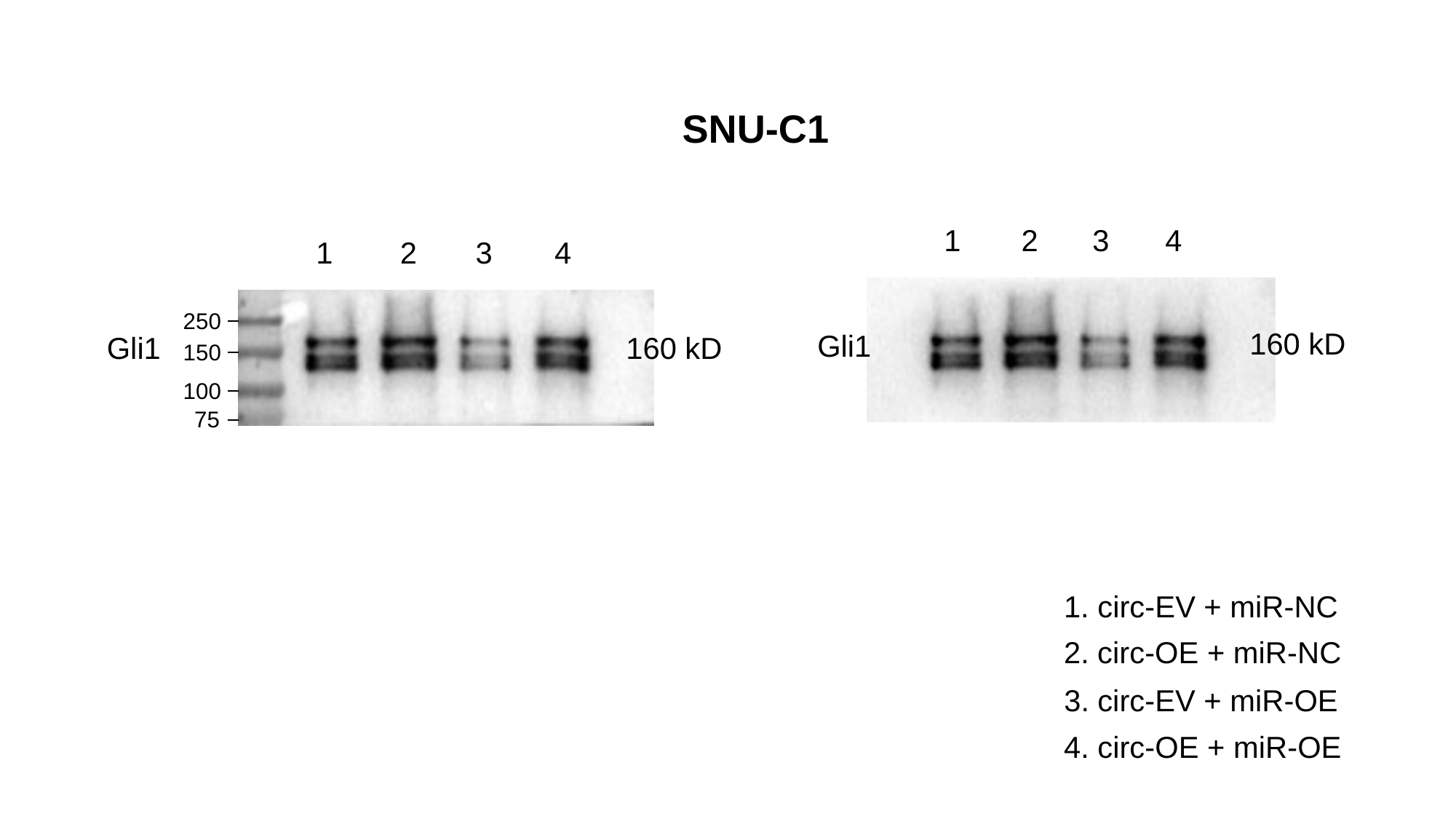

SNU-C1
1
2
3
4
160 kD
Gli1
1
2
3
4
250
Gli1
160 kD
150
100
75
1. circ-EV + miR-NC
2. circ-OE + miR-NC
3. circ-EV + miR-OE
4. circ-OE + miR-OE

## Slide 5
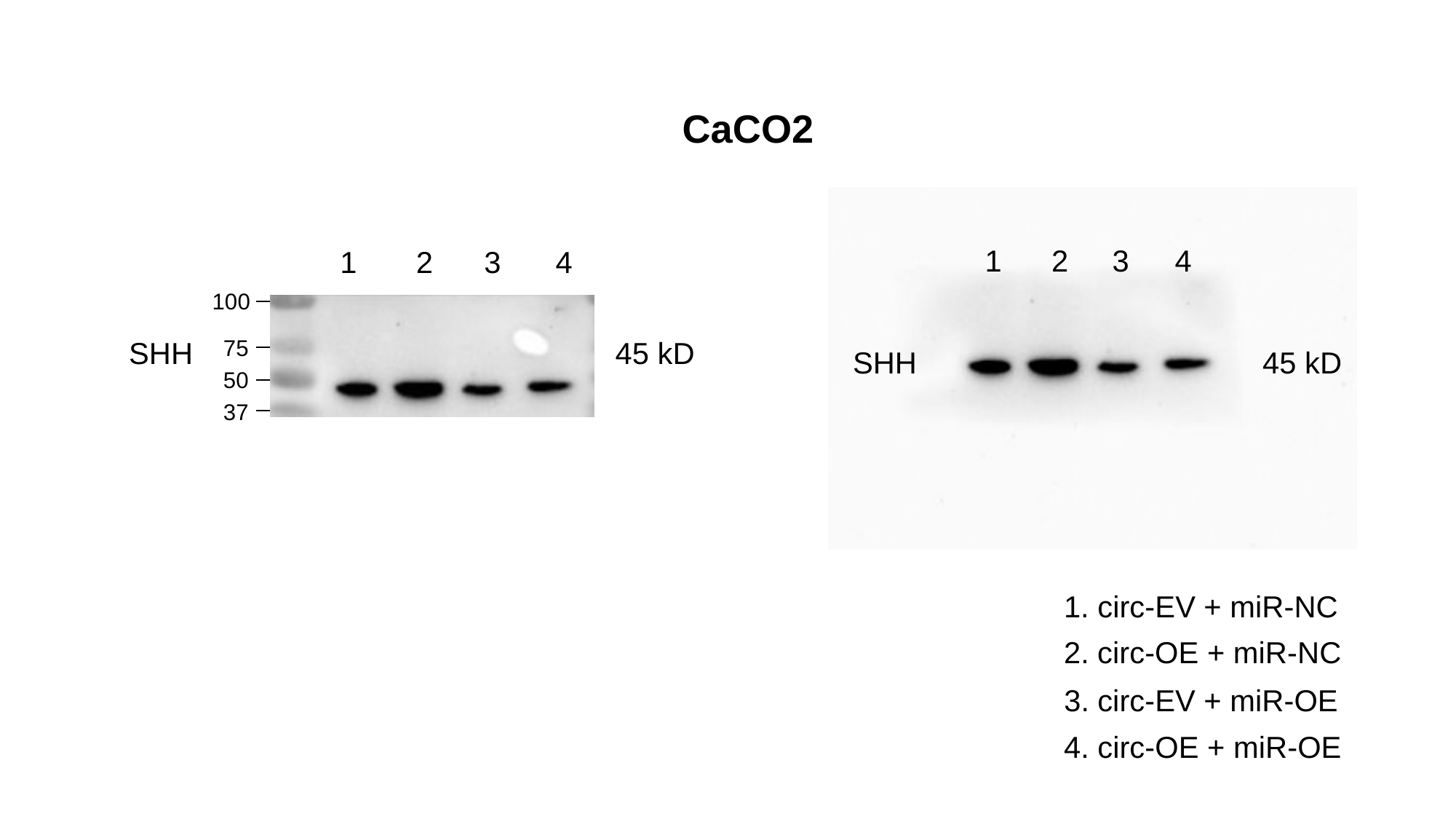

CaCO2
1
2
3
4
SHH
45 kD
1
2
3
4
100
75
SHH
45 kD
50
37
1. circ-EV + miR-NC
2. circ-OE + miR-NC
3. circ-EV + miR-OE
4. circ-OE + miR-OE

## Slide 6
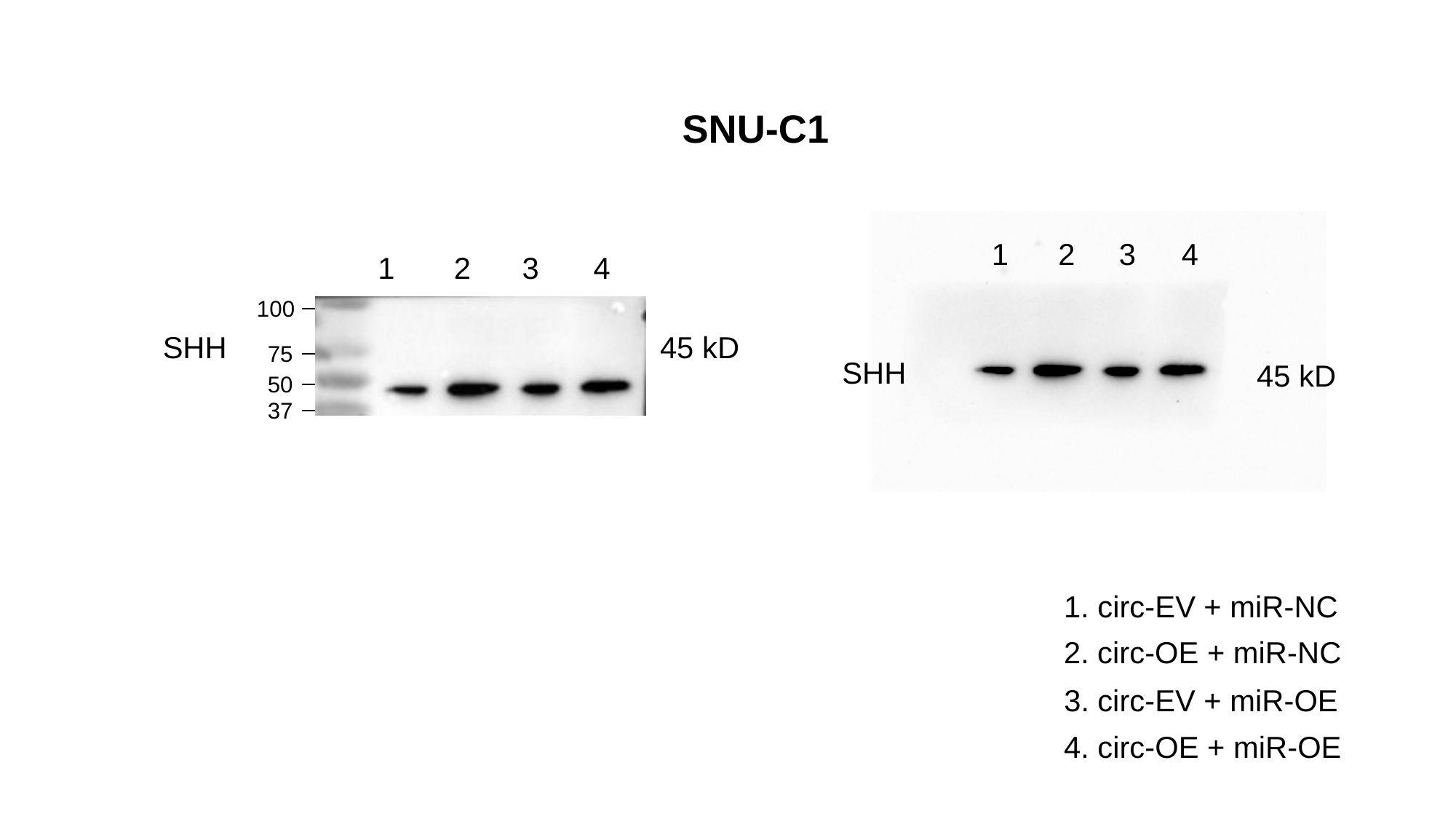

SNU-C1
1
2
3
4
SHH
45 kD
1
2
3
4
100
SHH
45 kD
75
50
37
1. circ-EV + miR-NC
2. circ-OE + miR-NC
3. circ-EV + miR-OE
4. circ-OE + miR-OE

## Slide 7
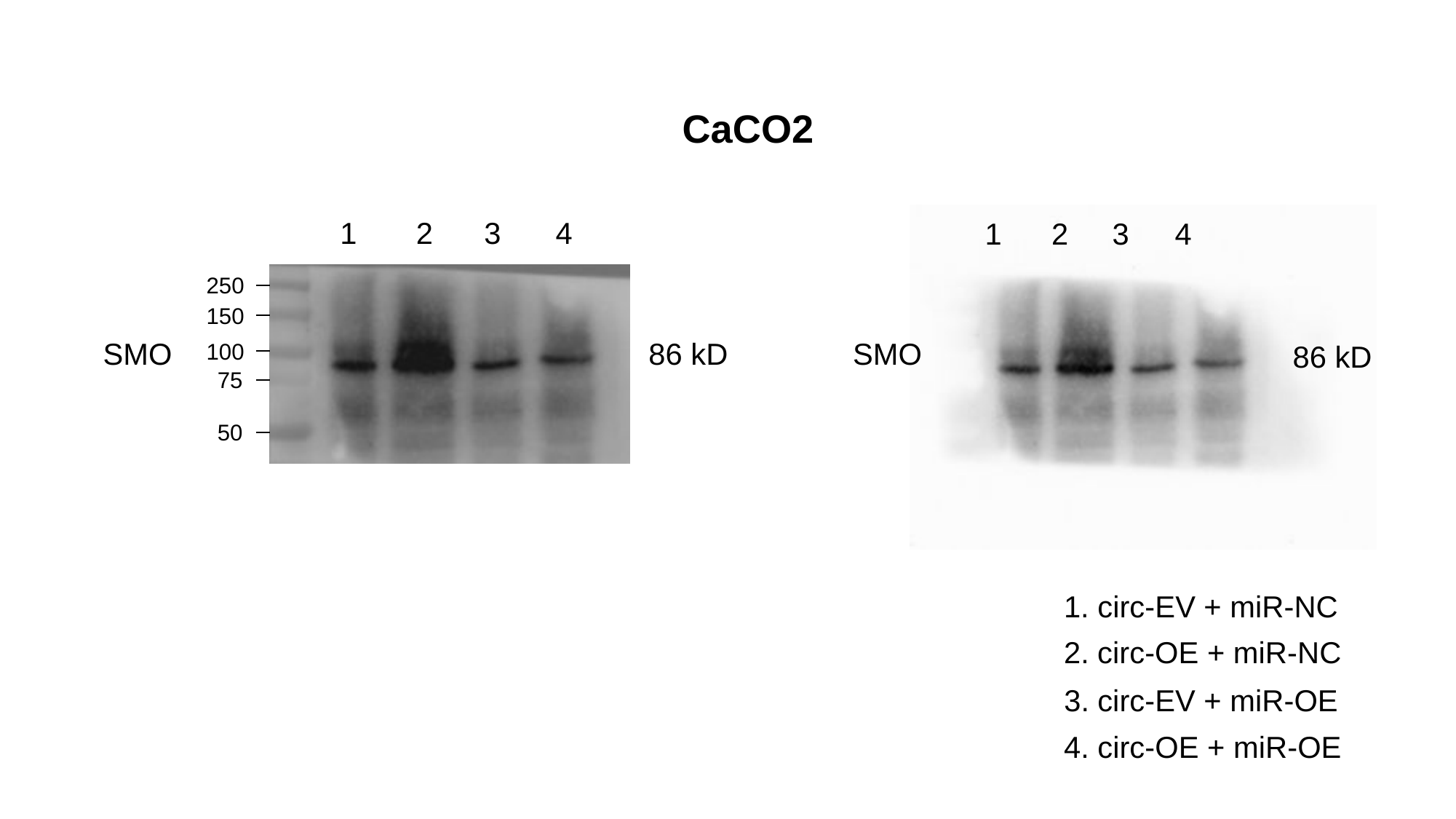

CaCO2
1
2
3
4
1
2
3
4
SMO
86 kD
250
150
SMO
86 kD
100
75
50
1. circ-EV + miR-NC
2. circ-OE + miR-NC
3. circ-EV + miR-OE
4. circ-OE + miR-OE

## Slide 8
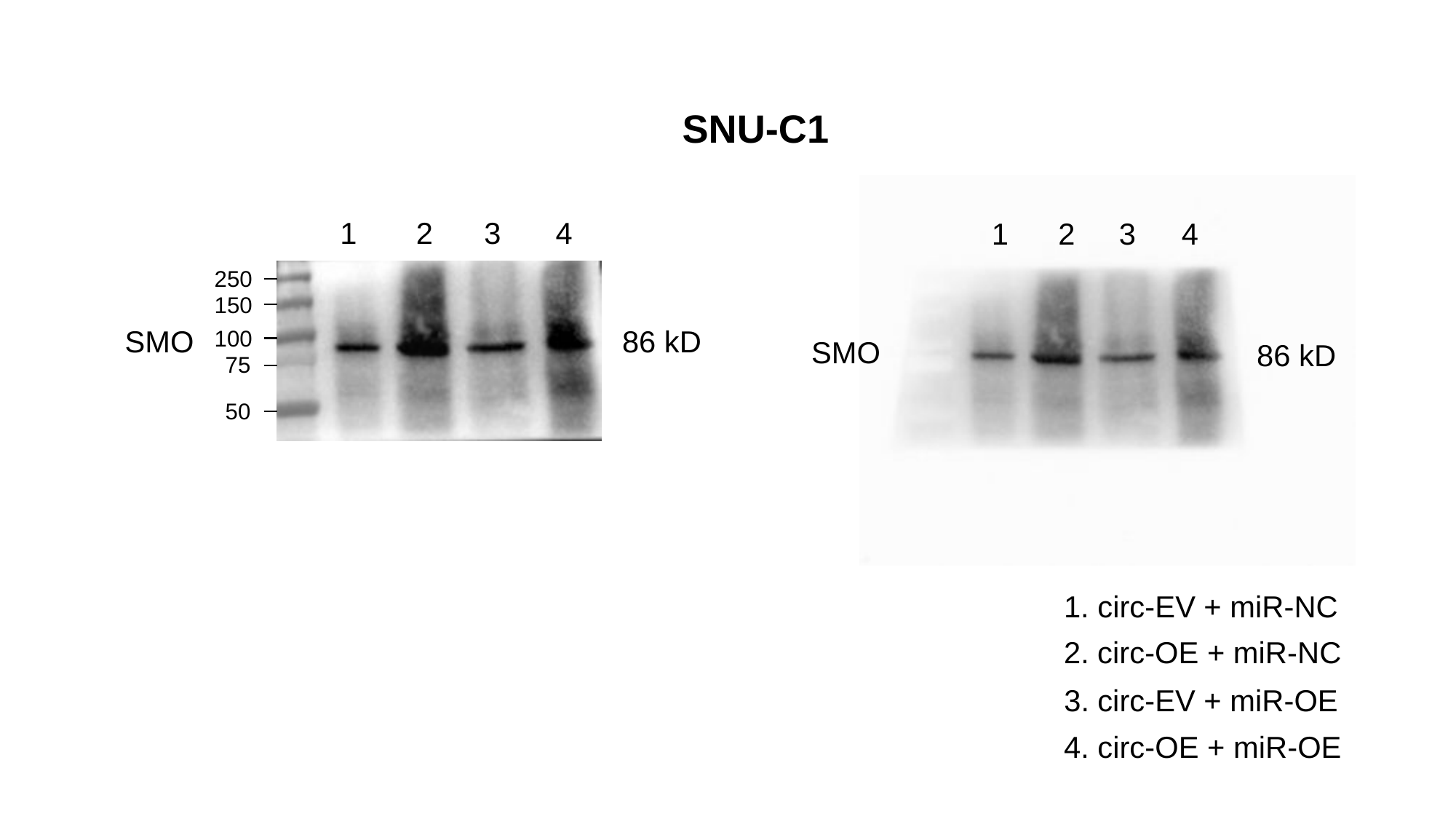

SNU-C1
1
2
3
4
1
2
3
4
SMO
86 kD
250
150
SMO
86 kD
100
75
50
1. circ-EV + miR-NC
2. circ-OE + miR-NC
3. circ-EV + miR-OE
4. circ-OE + miR-OE

## Slide 9
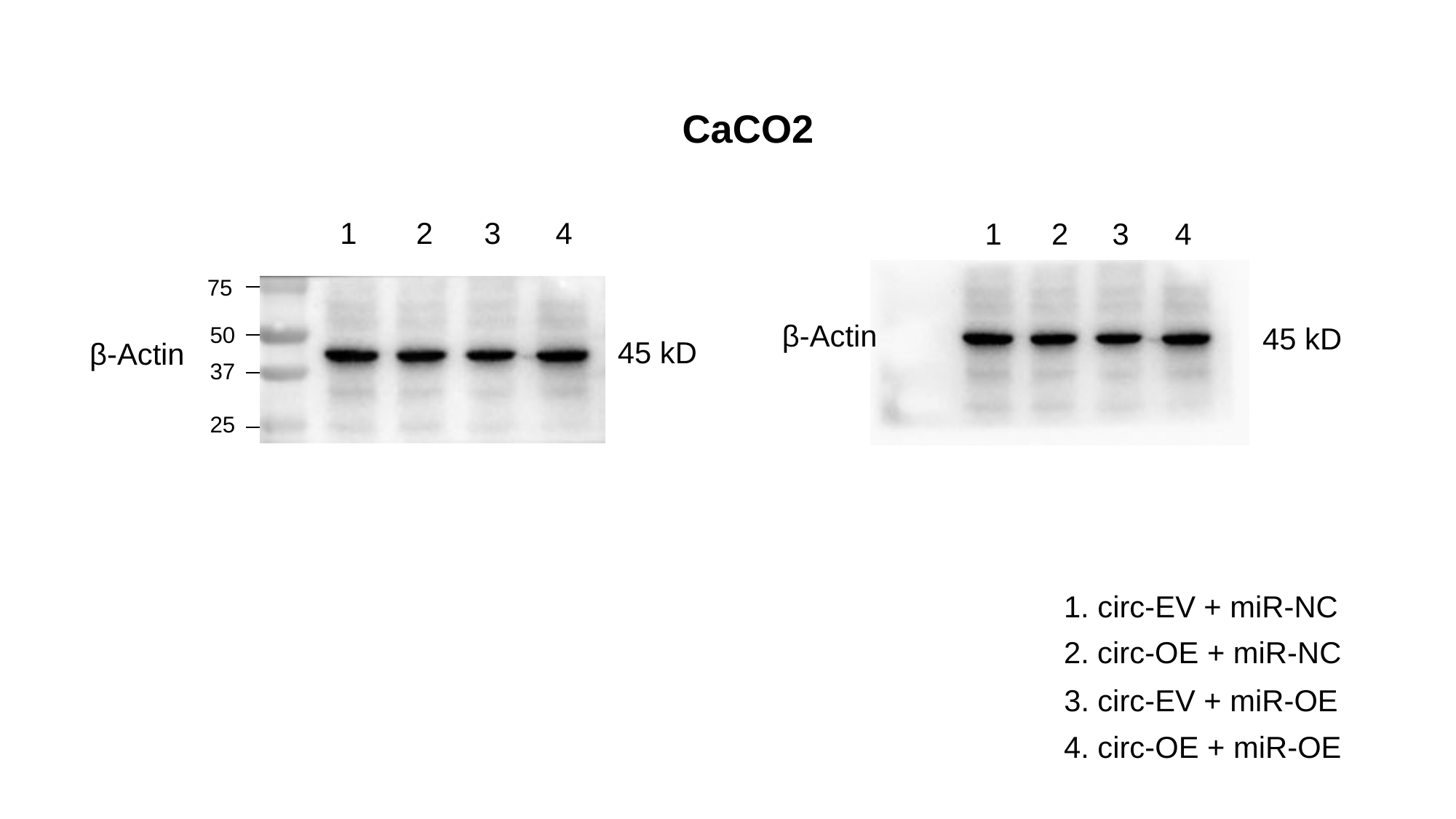

CaCO2
1
2
3
4
1
2
3
4
β-Actin
45 kD
75
50
45 kD
β-Actin
37
25
1. circ-EV + miR-NC
2. circ-OE + miR-NC
3. circ-EV + miR-OE
4. circ-OE + miR-OE

## Slide 10
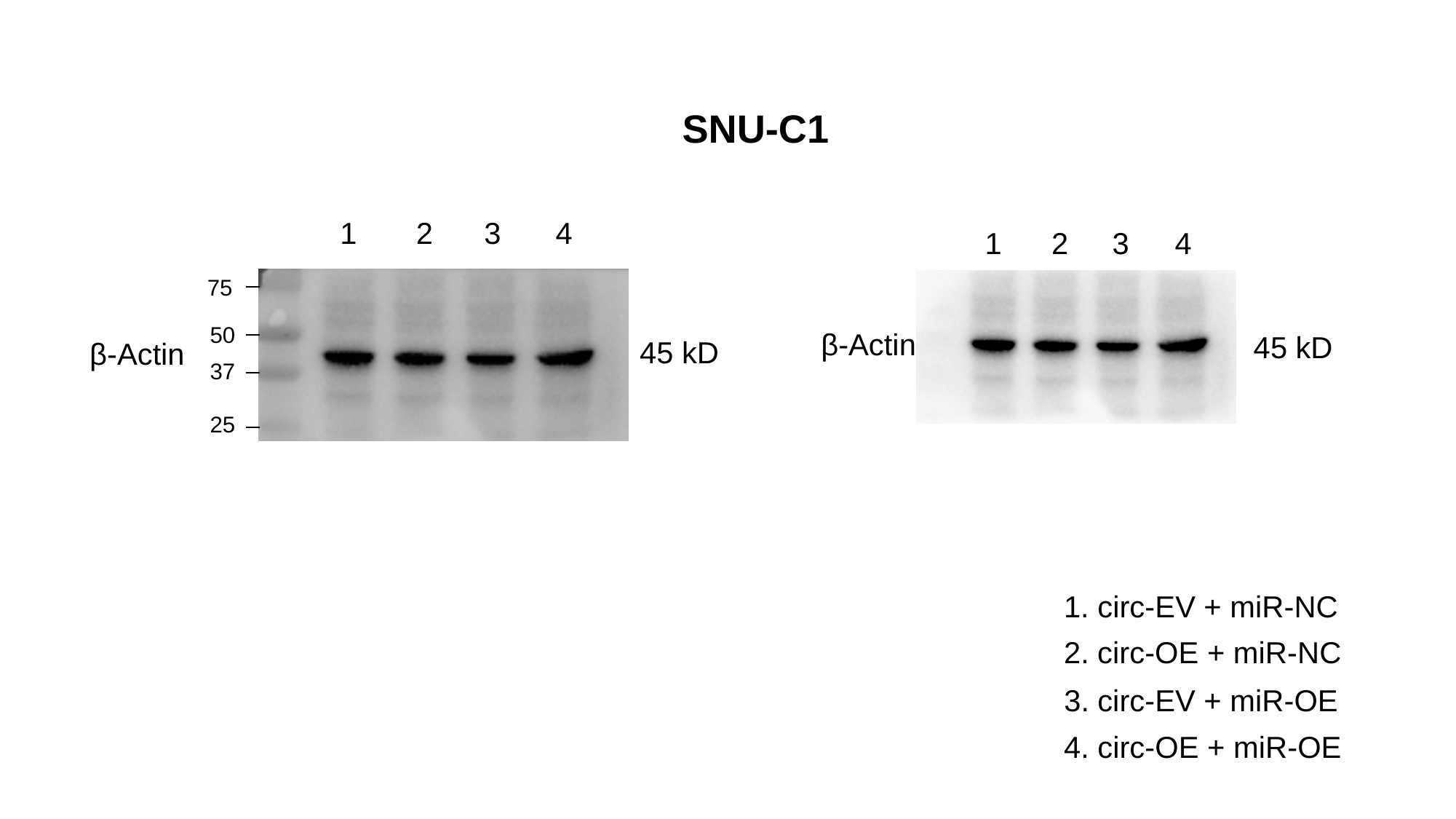

SNU-C1
1
2
3
4
1
2
3
4
β-Actin
45 kD
75
50
45 kD
β-Actin
37
25
1. circ-EV + miR-NC
2. circ-OE + miR-NC
3. circ-EV + miR-OE
4. circ-OE + miR-OE
