# Supplementary material for: circ-EGFR is a predictor of response to Cetuximab and a potential target in colorectal cancer
Source: EMBO Mol Med. 2025 Nov 10;17(12):3525–54. doi: 10.1038/s44321-025-00333-0 (PMC12686431; doi:10.1038/s44321-025-00333-0)
Supplement: Supplementary file 14 — Expanded View Figures [file 44321_2025_333_MOESM14_ESM.pdf]

Expanded View Figures

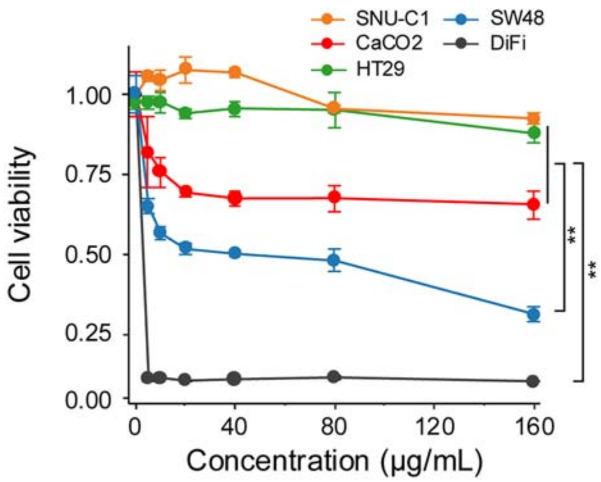

IC50 (μg/mL)	CaCO2	SNU-C1	HT29	SW48	DiFi
	N/A	N/A	N/A	34.77	< 5

Figure EV1. Cell viability of five colorectal cancer cell lines measured 48 h after initial exposure to cetuximab.

GEM-R vs. SW48: $p = 0.002$, GEM-R vs. DiFi: $p = 0.001$ (two-way ANOVA). Data were representative of three independent biological replicates. Bars represent the mean, and error bars indicate SD. ** $p < 0.01$, N/A not available.

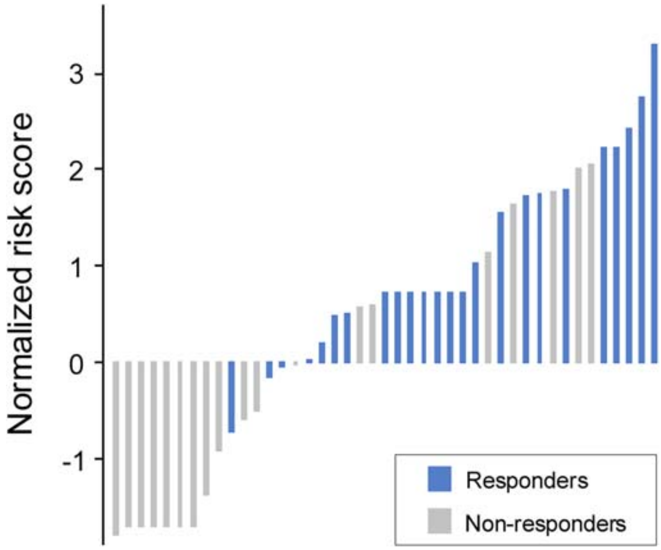

Figure EV2. The waterfall plot depicts the risk probability distribution of the normalized risk score between the responder and non-responder groups.

 © The Author(s)

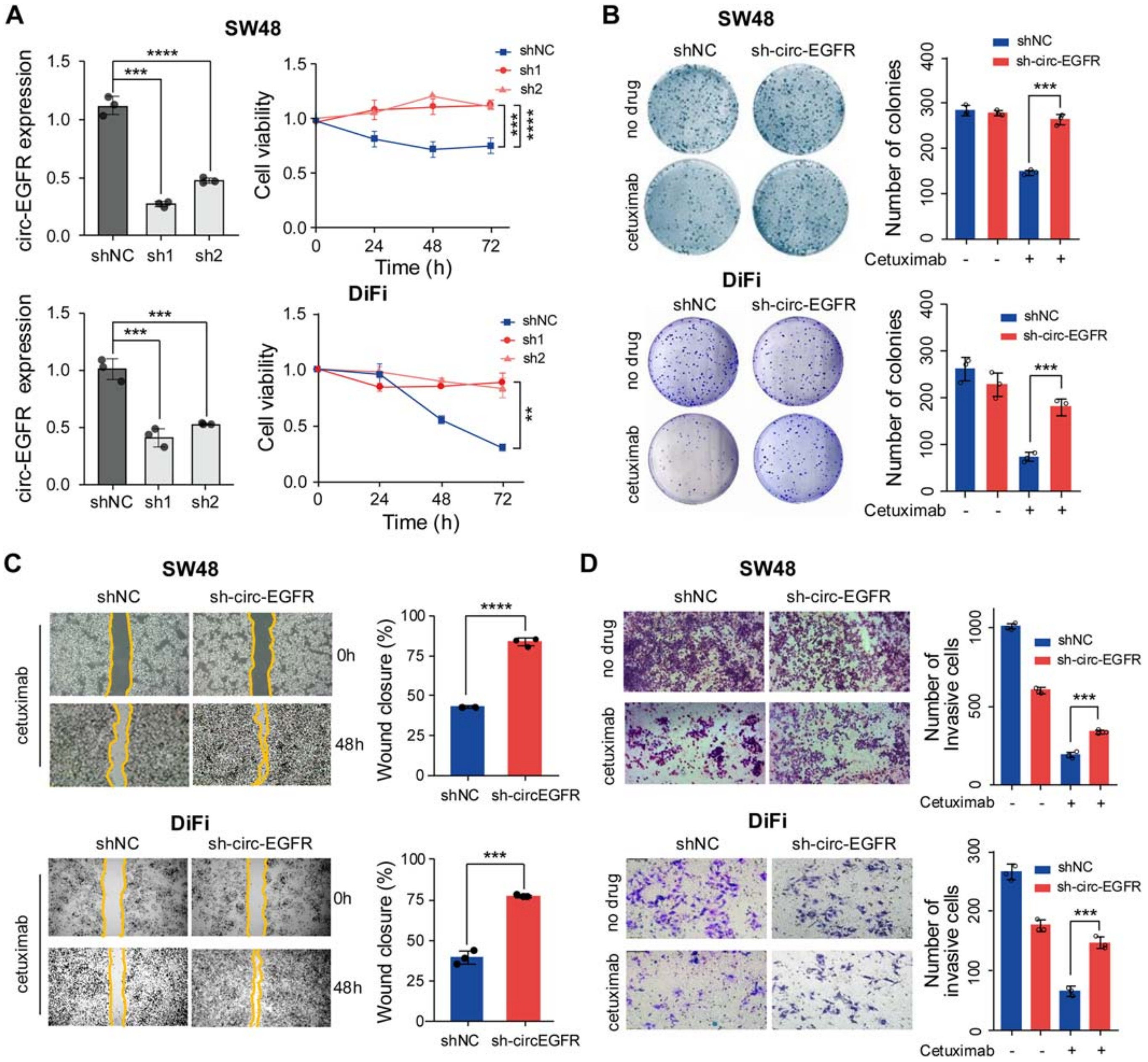

Figure EV3. Circ-EGFR depletion inhibits the response of CRC cells to cetuximab in vitro.

(**A**) The expression of circ-EGFR in stable circ-EGFR-silenced (sh1 and sh2) or negative control (shNC) from DiFi and SW48 cell lines (left). Assessment of cell proliferation capacity by MTT assay in circ-EGFR knockdown (sh1 and sh2) or shNC group from DiFi and SW48 cell lines after 48 h treatment with cetuximab (right). circ-EGFR expression of shNC vs. sh1 in SW48: $p < 0.0001$, shNC vs. sh2 in SW48: $p = 0.0002$, shNC vs. sh1 in DiFi: $p = 0.001$, shNC vs. sh2 in DiFi: $p = 0.0007$ (student's t-test). Cell viability of shNC vs. sh1 in SW48: $p = 0.0004$, shNC vs. sh2 in SW48: $p < 0.0001$, shNC vs. sh1 in DiFi: $p = 0.0004$, shNC vs. sh2 in DiFi: $p = 0.0004$ (two-way ANOVA). (**B**) Proliferation in stable circ-EGFR knockdown (sh-circ-EGFR) or shNC from DiFi and SW48 cells as determined by colony formation assay. SW48 $p = 0.0001$, DiFi $p = 0.0007$ (student's t-test). (**C**) Wound healing assay of sh-circ-EGFR or shNC in DiFi and SW48 cell lines after 48 h treatment with cetuximab. Scale bar = 50 µm. SW48 $p < 0.0001$, DiFi $p < 0.0001$ (Student's *t*-test). (**D**) Invasion assay of sh-circ-EGFR or shNC in DiFi and SW48 cell lines with or without cetuximab treatment. Scale bar = 100 µm. The number of invading cells was counted in randomly selected three fields. SW48 $p = 0.0002$, DiFi $p = 0.0007$ (student's *t*-test). Data were representative of at least three independent biological replicates. Bars represent the mean, and error bars indicate SD. *$p < 0.05$, **$p < 0.01$, ***$p < 0.001$, **** $p < 0.0001$, ns not significant.

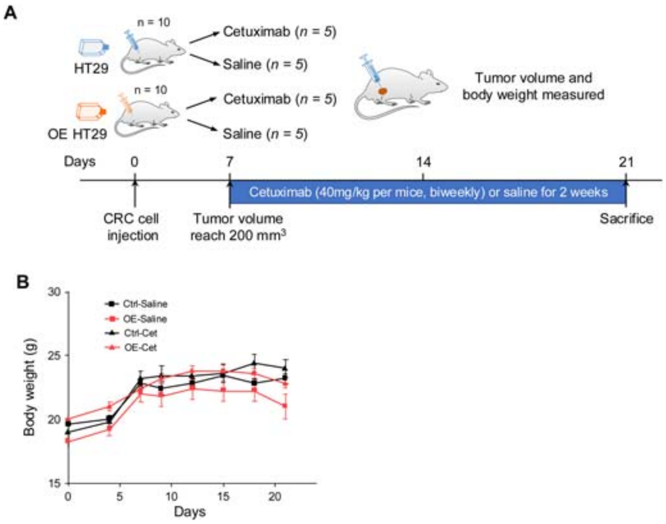

Figure EV4. Circ-EGFR enhances the efficacy of cetuximab in vivo.

(**A**) Schematic diagram of the HT29 cell (transfected with circ-EGFR and mock plasmids)-derived xenograft model in 20 nude mice and the treatment schedule of cetuximab. (**B**) The body weight of nude mice injected with circ-EGFR and mock plasmids in each treatment group was measured at different time points after inoculation. Ctrl Cet: mice inoculated with the HT29 cell line transfected with vector control and treated with cetuximab, Ctrl Saline: mice inoculated with the HT29 cell line transfected with vector control and treated with saline, OE Cet: mice inoculated with circ-EGFR overexpressing HT29 cells and treated with cetuximab, OE Saline: mice inoculated with circ-EGFR overexpressing HT29 cells and treated with saline. Data were representative of five biological replicates. Bars represent the mean, and error bars indicate SD.

 © The Author(s)

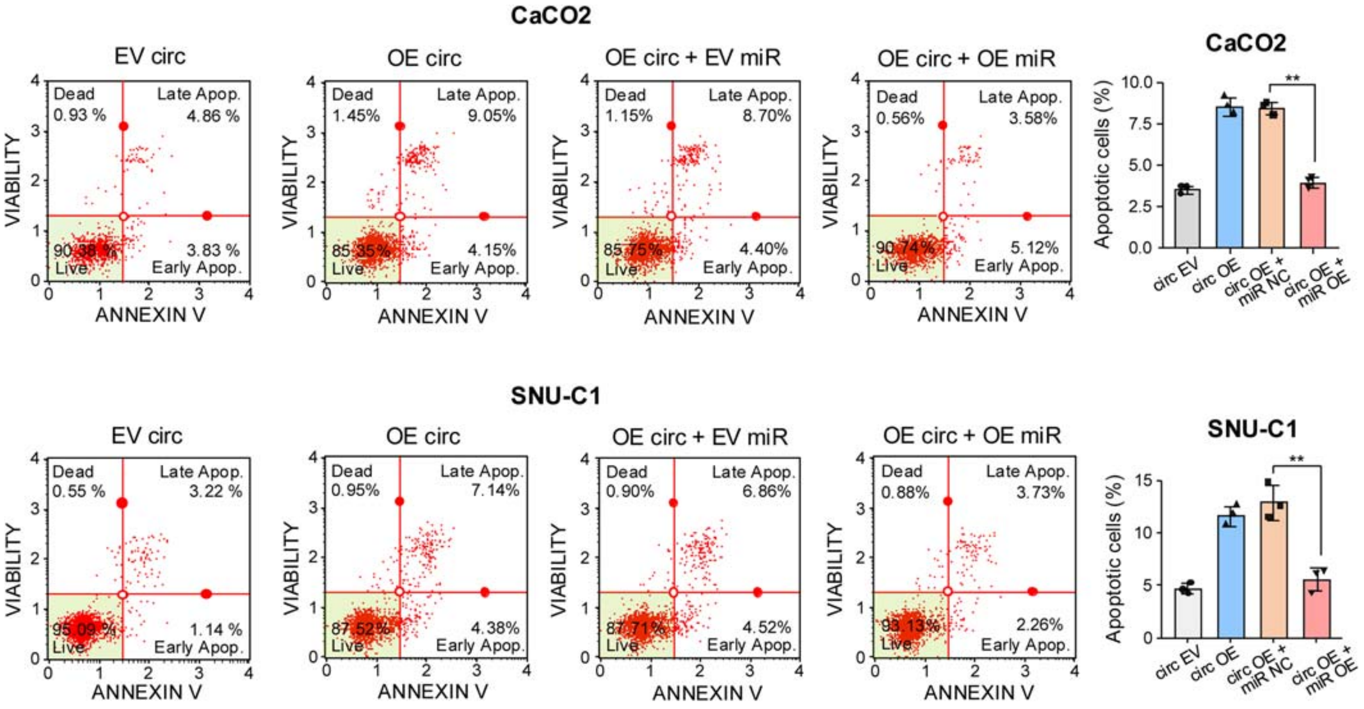

Figure EV5. Representative images of cells undergoing apoptosis that stained for the annexin V assay in CaCO2 and SNU-C1 transfected with circ-EGFR and/or miR-942-3p plasmids.

DiFi $p = 0.006$, SW48 $p = 0.005$ (student's *t*-test). Data were representative of three independent biological replicates. Bars represent the mean, and error bars indicate SD. **$p < 0.01$.

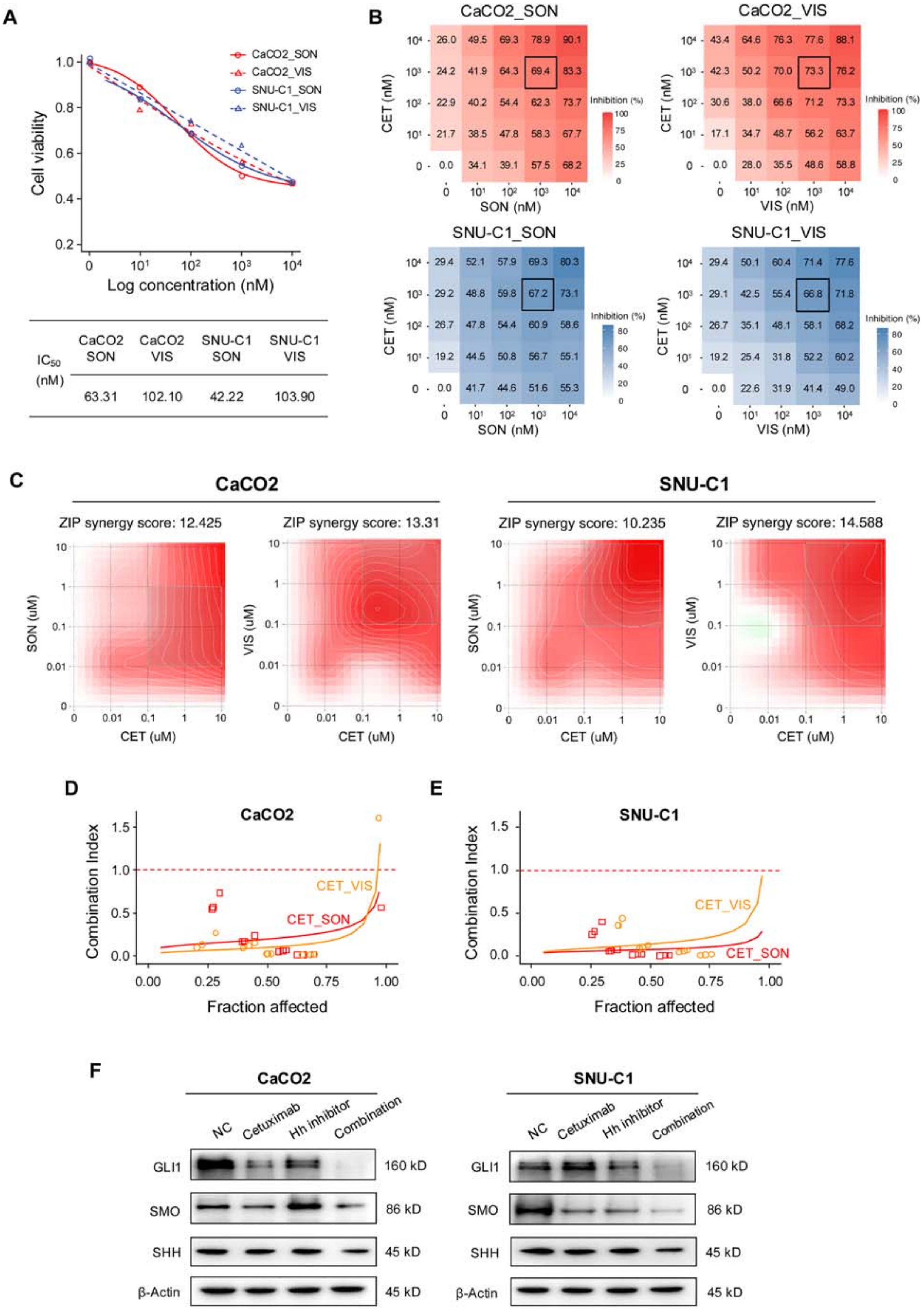
A
CaCO2_SON
CaCO2_VIS
SNU-C1_SON
SNU-C1_VIS
Cell viability
Log concentration (nM)
IC50 (nM)
CaCO2 SON 63.31
CaCO2 VIS 102.10
SNU-C1 SON 42.22
SNU-C1 VIS 103.90
B
CaCO2_SON
CaCO2_VIS
SNU-C1_SON
SNU-C1_VIS
CET (nM)
SON (nM)
VIS (nM)
Inhibition (%)
C
CaCO2
SNU-C1
ZIP synergy score: 12.425
ZIP synergy score: 13.31
ZIP synergy score: 10.235
ZIP synergy score: 14.588
SON (uM)
VIS (uM)
CET (uM)
D
CaCO2
E
SNU-C1
Combination Index
Fraction affected
CET_VIS
CET_SON
F
CaCO2
SNU-C1
NC
Cetuximab
Hh inhibitor
Combination
GLI1
SMO
SHH
β-Actin
160 kD
86 kD
45 kD
45 kD

 © The Author(s)

Figure EV6. Synergistic effects of cetuximab and hedgehog inhibitor combinations in CRC cells.

(**A**) Dose-response curves for Sonidegib or Vismodegib in CaCO2 and SNU-C1 cells. (**B**) Dose-response matrix (inhibition) for Cetuximab and Sonidegib, or Cetuximab and Vismodegib in CaCO2 and SNU-C1 cells. (**C**) Synergy distribution and scores for different drug combinations in CaCO2 and SNU-C1 cells were assessed using SynergyFinder with the ZIP model. (**D**, **E**) Combination index (CI-Fa) plots for CaCO2 and SNU-C1 cells treated with combinations of Cetuximab and Sonidegib or Cetuximab and Vismodegib. (**F**) Western blot analysis of Hh signaling pathway components in CaCO2 and SNU-C1 following Cetuximab, Sonidegib or Vismodegib, and the combination therapy. β-Actin served as an internal control.

© The Author(s)

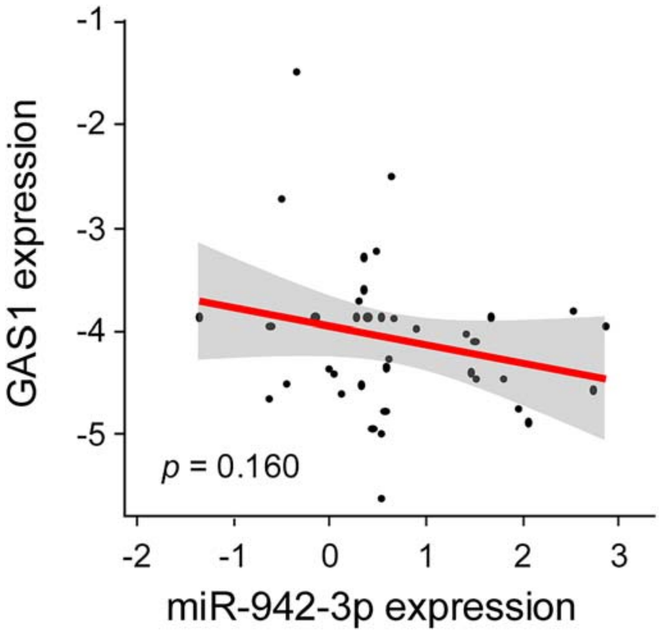

Figure EV7. The correlation between miR-942-3p expression and GAS1 expression in the clinical trial cohort.

$p = 0.160$ (Pearson correlation analysis).

 © The Author(s)
